# The evolution of the hypotetraploid *Catolobus pendulus* genome – the poorly known sister species of *Capsella*

**DOI:** 10.3389/fpls.2023.1165140

**Published:** 2023-05-08

**Authors:** Perla Farhat, Terezie Mandáková, Jan Divíšek, Hiroshi Kudoh, Dmitry A. German, Martin A. Lysak

**Affiliations:** ^1^ Central European Institute of Technology (CEITEC), Masaryk University, Brno, Czechia; ^2^ Department of Experimental Biology, Faculty of Science, Masaryk University, Brno, Czechia; ^3^ Department of Botany and Zoology, Faculty of Science, Masaryk University, Brno, Czechia; ^4^ Center for Ecological Research, Kyoto University, Otsu, Japan; ^5^ South-Siberian Botanical Garden, Altai State University, Barnaul, Russia; ^6^ National Centre for Biomolecular Research (NCBR), Faculty of Science, Masaryk University, Brno, Czechia

**Keywords:** chromosome painting, Hyb-Seq, Arabidopsis-related model systems, Brassicaceae, Cruciferae, polyploidy, diploidization, whole-genome duplication (WGD)

## Abstract

The establishment of *Arabidopsis* as the most important plant model has also brought other crucifer species into the spotlight of comparative research. While the genus *Capsella* has become a prominent crucifer model system, its closest relative has been overlooked. The unispecific genus *Catolobus* is native to temperate Eurasian woodlands, from eastern Europe to the Russian Far East. Here, we analyzed chromosome number, genome structure, intraspecific genetic variation, and habitat suitability of *Catolobus pendulus* throughout its range. Unexpectedly, all analyzed populations were hypotetraploid (2*n* = 30, ~330 Mb). Comparative cytogenomic analysis revealed that the *Catolobus* genome arose by a whole-genome duplication in a diploid genome resembling Ancestral Crucifer Karyotype (ACK, *n* = 8). In contrast to the much younger *Capsella* allotetraploid genomes, the presumably autotetraploid *Catolobus* genome (2*n* = 32) arose early after the *Catolobus*/*Capsella* divergence. Since its origin, the tetraploid *Catolobus* genome has undergone chromosomal rediploidization, including a reduction in chromosome number from 2*n* = 32 to 2*n* = 30. Diploidization occurred through end-to-end chromosome fusion and other chromosomal rearrangements affecting a total of six of 16 ancestral chromosomes. The hypotetraploid *Catolobus* cytotype expanded toward its present range, accompanied by some longitudinal genetic differentiation. The sister relationship between *Catolobus* and *Capsella* allows comparative studies of tetraploid genomes of contrasting ages and different degrees of genome diploidization.

## 1 Introduction

Eighty years ago, *Arabidopsis thaliana* (L.) Heynh. (Arabidopsis) was proposed as an ideal plant model system (Laibach, 1943). The genome sequence of Arabidopsis (Arabidopsis Genome Initiative, 2000) and the rich genomic resources stimulated analyzes of other members of the mustard family (crucifers, Brassicaceae), with different phylogenetic distance from Arabidopsis (e.g., Schranz et al., 2006; Koch et al., 2008; Koenig and Weigel, 2015). Species of *Arabidopsis*, *Arabis*, *Brassica*, *Capsella*, *Eutrema*, and *Schrenkiella* have become models in various fields of plant biology. However, family-wide comparative studies are hampered by the common morphological convergence, resulting in some species and genera being erroneously grouped together based on the similarity of their morphological traits (Al-Shehbaz, 1986; Mummenhoff et al., 1997; Koch and Mummenhoff, 2001; Al-Shehbaz, 2003; Al-Shehbaz et al., 2006; Huang et al., 2016). This is typically the case with *Arabis* L., which was considered one of the most taxonomically difficult genera in the family (Al-Shehbaz, 2003; Al-Shehbaz et al., 2006). Previously, this genus was delimited based on the combination of three main morphological characters (branched trichomes, latiseptate siliquae and accumbent cotyledons). However, this combination is not unique to *Arabis* and evolved independently multiple times in the Brassicaceae (Koch et al., 2003; Al-Shehbaz, 2005; Al-Shehbaz et al., 2006). Since several molecular studies have shown that *Arabis* is polyphyletic (O’Kane and Al-Shehbaz, 2003; Bailey et al., 2006; Beilstein et al., 2006; Warwick et al., 2006; Beilstein et al., 2008; Koch et al., 2008), efforts have been made to taxonomically assign the non-*Arabis* species to new genera (Al-Shehbaz, 2003; Al-Shehbaz, 2005). O’Kane and Al-Shehbaz (2003) were the first to demonstrate, based on phylogenetic analysis of nuclear ribosomal DNA sequences, that *Arabis pendula* L. is closely related to *Capsella* Medik. and other genera of the tribe Camelineae (supertribe Camelinodae, German et al., 2023). Based on this work, Al-Shehbaz (2005) transferred *A. pendula* to a newly erected unispecific genus *Catolobus* (C.A.Mey.) Al-Shehbaz.

*Catolobus pendulus* (L.) Al-Shehbaz is a biennial herb with an erect stem (30 - 200 cm tall), petiolate basal and sessile stem leaves, white petals, and pendulous siliques. Its natural range extends from Ukraine and Belarus through European Russia, Siberia, Kazakhstan, Kyrgyzstan and China to Korea, Japan, and the Russian Far East (about 6 700 km range). The species inhabits a wide range of mesic habitats at different altitudes from 0 to 4 300 m a. s. l., namely rocky slopes, roadsides, woodlands, forest edges, glades, riverbanks, and wastelands (ruderal areas).

Although the close phylogenetic relationship of *Catolobus* with *Arabidopsis* (DC.) Heynh., *Capsella*, and *Camelina* Crantz was established two decades ago (O’Kane and Al-Shehbaz, 2003) and has been recently confirmed by several other studies (e.g., Huang et al., 2016; Žerdoner Čalasan et al., 2019; Nikolov et al., 2019), the origin and evolution of the *Catolobus* genome remain poorly understood. The sparse karyological data make *Catolobus* one of the most intriguing Camelineae taxa. The currently known chromosomal reports are confusing and enigmatic. The only available reports from the Russian Far East report diploid (2*n* = 16) and near-triploid (2*n* = 21) chromosome numbers (Berkutenko and Gurzenkov, 1976; Berkutenko et al., 1984), whereas chromosome counts from most parts of Eurasia are lacking. These authors suggested that fertile, near-triploid plants might reproduce by apomixis. Because *Catolobus* and *Capsella* are sister genera (O’Kane and Al-Shehbaz, 2003; Huang et al., 2016; Žerdoner Čalasan et al., 2019) and *Capsella* (5 spp.) has become an increasingly popular model genus for studying polyploidization, selfing, and their association with diversification and speciation (e.g., Foxe et al., 2009; Guo et al., 2009; Douglas et al., 2015; Petrone Mendoza et al., 2018; Orsucci et al., 2022), understanding the origin, structure, and evolution of the *Catolobus* genome should be informative for comparative genomic analyses of model systems related to Arabidopsis (Koch et al., 2008; Koenig and Weigel, 2015).

Here, we aimed to analyze different populations of *C. pendulus* throughout the species’ range to decipher their genome architecture using comparative chromosome painting and to obtain a comprehensive picture of chromosome number variation. In addition, we used Hyb-Seq and plastome data to analyze intraspecific genetic diversity across the entire geographic range and phylogenetic relationships of the unispecific *Catolobus* within the tribe Camelineae. We also modeled the historical ecological niches and distributions of *Catolobus* and the sister *Capsella* species to clarify how the present-day distributions of these species formed. Interestingly, we found that the predominant or only extant *Catolobus* genome originated most likely as autotetraploid (2*n* = 4*x* = 32), followed by a moderate genome diploidization towards the present hypotetraploid genome (2*n* = 30) and its expansion throughout Eurasia.

## 2 Materials and methods

### 2.1 Plant material

A list of the investigated accessions and their origins are provided in **Supplementary Table S1** (**Supplementary Figure S1**). Plants were grown from seed and cultivated under standard conditions in growth chambers (150 µmol m^-2^ s^-1^; 21/18°C, day/night; 16/8 h light/dark) or in a greenhouse (150 µmol m^-2^ s^-1^; 22/19°C, day/night; 16/8 h light/dark). Whole young inflorescences from different individuals were fixed in freshly prepared ethanol: acetic acid (3: 1) fixative overnight, transferred to 70% ethanol and stored at -20°C until further use (see below). Fresh leaves or leaf samples from herbarium specimens were used to extract genomic DNA using the NucleoSpin Plant II kit (Macherey-Nagel). Homoploid genome size was estimated by flow cytometry using nuclei isolated from fresh leaves of populations 4, 23, 30, and 53 as described by Dogan et al. (2022).

### 2.2 Chromosome preparations

Mitotic and meiotic chromosome spreads from fixed young flower buds containing immature anthers were prepared as described previously (Mandáková and Lysak, 2016a). Briefly, selected flower buds were rinsed in distilled water (twice for 5 min) and citrate buffer (10 mM sodium citrate, pH 4.8; twice for 5 min) and digested in 0.3% cellulase, cytohelicase, and pectolyase (all Sigma-Aldrich) in citrate buffer at 37°C for 3 h. After digestion, individual anthers were dissected and spread on a microscope slide placed on a hot metal plate (50°C) (20 μl of 60% acetic acid, c. 30 s). The preparation was then fixed in freshly prepared fixative (ethanol: acetic acid, 3:1) by dropping the fixative around and into the spread. Chromosome spreads were dried with a hair dryer and checked under a phase contrast for suitable chromosome figures that were largely free of cytoplasm. Suitable slides were post-fixed in freshly prepared 4% formaldehyde in distilled water for 10 min and air-dried. The preparations were stored in a dust-free box at room temperature until use.

To remove the RNA and remaining cytoplasm, the preparations were treated with 100 µg/ml RNase (AppliChem) in 2× sodium saline citrate (SSC; 20× SSC: 3 M sodium chloride, 300 mM trisodium citrate, pH 7.0) for 60 min and 0.1 mg/ml pepsin (Sigma) in 0.01 M HCl at 37°C for 5 min. They were then post-fixed in 4% formaldehyde in 2× SSC for 10 min, washed in 2× SSC twice for 5 min, dehydrated in an ethanol series (70%, 90%, and 100%, 2 min each), and air-dried.

### 2.3 DNA probes

For comparative chromosome painting (CCP) in *Catolobus*, a total of 674 chromosome-specific BAC clones of *A. thaliana* grouped into contigs corresponding to eight chromosomes and 22 genomic blocks (GBs) of the Ancestral Crucifer Karyotype (Lysak et al., 2016) were used. See Mandáková et al. (2019) for delineation of the GB boundaries. To determine and characterize *Catolobus*-specific chromosome arrangements, some BAC contigs were split into smaller subcontigs after initial CCP experiments. The *A. thaliana* BAC clone T15P10 (AF167571), containing 35S rRNA genes, was used for *in situ* localization of nucleolar organizer regions (NORs), and the *A. thaliana* clone pCT4.2 (M65137), containing a 500-bp 5S rDNA repeat, was used for localization of 5S rDNA loci.

All DNA probes were labeled by nick translation with biotin-dUTP, digoxigenin-dUTP or Cy3-dUTP according to Mandáková and Lysak (2016b) as follows: 1 μg DNA diluted in distilled water to 29 µl, 5 μl nucleotide mix (2 mM dATP, dCTP, dGTP, 400 μM dTTP, all Roche), 5 μl 10× NT-buffer (0.5 M Tris-HCl, pH 7.5; 50 mM MgCl2, 0.05% bovine serum albumin), 4 μl 1 mM X-dUTP (where X was either biotin, digoxigenin, or Cy3), 5 μL 0.1 M β-mercaptoethanol, 1 μl DNase I (Roche), and 1 μl DNA polymerase I (Fermentas). The nick translation mixture was incubated at 15°C for 90 min (or longer) to obtain a fragment length of ~200 to 500 bp. The nick translation reaction was stopped by adding 1 μl 0.5 M EDTA, pH 8.0, and incubating at 65°C for 10 min. The individual labeled probes were stored at -20°C until use.

### 2.4 Fluorescence *in situ* hybridization and microscopy

Selected labeled probes were pooled according to the design of a particular experiment and precipitated by adding 1/10 volume of 3 M sodium acetate, pH 5.2, and 2.5 volumes of ice-cold 96% ethanol and kept at -20°C for 30 min. The pellet was then centrifuged at 13 000 g at 4°C for 30 min. The pellet was resuspended in 20 µl of the hybridization mix (50% formamide and 10% dextran sulfate in 2×SSC) per slide. 20 µl of the probe was pipetted onto a chromosome-containing slide. The cover slips were framed with rubber cement. The probe and chromosomes were denatured together on a hot plate at 80°C for 2 min and incubated in a moist chamber at 37°C overnight.

Post-hybridization washing was performed in 20% formamide in 2× SSC at 42°C. Immunodetection of hapten-labeled probes was performed as follows according to Mandáková and Lysak (2016b): biotin-dUTP was detected by avidin–Texas Red (Vector Laboratories) and amplified by goat anti-avidin–biotin (Vector Laboratories) and avidin–Texas Red; digoxigenin-dUTP was detected by mouse anti-digoxigenin (Jackson Immuno Research) and goat anti-mouse–Alexa Fluor 488 (Invitrogen). Cy3-dUTP-labeled probes were observed directly. After immunodetection, chromosomes were counterstained with 4’, 6-diamidino-2-phenylindole (DAPI, 2 µg/ml) in Vectashield (Vector Laboratories).

### 2.5 Hyb-Seq experimental design and data processing

For target enrichment, we used two bait sets, the Angiosperms-353 (Angio353, 353 single-copy genes) and the Brassicaceae-specific baits (1 827 exons comprising 761 single-copy genes) (Johnson et al., 2019; Nikolov et al., 2019). Both bait sets were provided by Arbor Biosciences (Arbor Biosciences, Ann Arbor, Michigan, USA). We combined both kits in one hybridization reaction using the same ratios and concentrations as elaborated by Hendriks et al. (2021). In total, we generated seven hybridization pools. Four pools contained *Catolobus* samples (maximum of eight samples/pool), two pools contained *Camelina* and *Neslia* samples, and one pool contained *Capsella* samples. Pools were created according to the best practices provided by the manufacturer (Arbor Biosciences), following three criteria for grouping samples: (i) taxonomic relatedness, (ii) DNA quality, and (iii) ploidy level. Libraries were sequenced at 150-bp paired-end on the Illumina Novaseq platform at the Genomics core facility, CEITEC MU. Raw sequence data were submitted to the NCBI Sequence Read Archive (SRA) under the BioProject PRJNA930157.

Reads were cleaned of adapters and low-quality bases with Trimmomatic v. 0.39 using the parameters ILLUMINACLIP: TruSeq3-PE.fa:2:30:10 LEADING:20 TRAILING:20 SLIDINGWINDOW:4:20 MINLEN:50. Data quality was inspected before and after cleaning using FastQC v0.11.9. Hybpiper v. 1.3.1 was used separately with Angio353 and Brassicaceae-specific bait targets to generate consensus sequences for each sample. Reads were mapped to references using BWA v. 0.7.13 and contigs were *de novo* assembled using SPADES and Exonerate v.2.2. Exons, introns, and supercontigs (exons + flanking regions) were retrieved using Hybpiper scripts. In addition, genes with paralogy warning were investigated using Hybpiper Python script paralogue_investigator.py. We identified 32 loci with overlap between Angio353 and Brassicaceae-specific baits using lastz V.1.04.15. To avoid analyzing the same markers twice, we kept one copy of these loci. In total, we analyzed 2 107 loci from both bait sets (**Supplementary Table S2**).

Two methods were used for plastome analysis based on the coverage of off-targets of each sample. In the first method, reads from each sample were mapped to the chloroplast reference genome of *A. thaliana* (RefSeq: NC_000932.1) using Geneious Mapper in Geneious Prime 2022.0.1 software. The consensus chloroplast sequences were extracted, with Ns inserted at sites without sequence coverage. The method described was performed because the coverage of off-target reads was insufficient to perform *de novo* assembly of the plastome for most of the samples. Sequence composition for each sample was examined using Seqtk (GitHub - lh3/seqtk: Toolkit for processing sequences in FASTA/Q formats). Samples with Ns frequency higher than 28% were excluded from this analysis. Data obtained by the first method were used for phylogenetic analysis. The second method concerned only the samples with high off-target coverage. In this case, plastome reads were extracted and assembled *de novo* (the method is detailed in section 2.9).

### 2.6 Ploidy level estimation of herbarium specimens

For most samples, the ploidy level was inferred from chromosome counts. For 11 herbarium samples (**Supplementary Table S1**), we applied the method based on allele frequency (Viruel et al., 2019). The frequency distribution of biallelic SNPs was examined using nQuire (Weiß et al., 2018), which uses NGS reads to elucidate intraspecific ploidy-level variation. The software requires NGS reads mapped to a reference as input. Because no reference is currently available for *Catolobus*, we used the supercontigs generated by Hybpiper as reference. Reads were mapped to the reference using BWA and sorted using Samtools v. 1.11. Bam alignments were cleaned using the denoise function of nQuire and analyzed using the lrdmodel, estmodel, and histotest models. We estimated the ploidy level of *Catolobus* populations based on four criteria: (i) comparison of allele frequency histograms between *Catolobus* samples with known and unknown ploidy levels along with the published histograms for diploid, triploid and tetraploid levels (Viruel et al., 2019), (ii) the lowest delta likelihood score, (iii) the best fit between the empirical and ideal histograms, characterized by a low standard error, a positive slope (y-y slope), a small sum of squared residuals (SSR) and a large R2, (iv) the median value of the allele ratios. For the last criterion, we calculated the distribution of allele ratios using the equation described by Viruel et al. (2019), which consisted of dividing the number of reads of the most frequent allele by the number of reads of the least frequent allele. The median allele ratios of samples with known chromosome number were compared to samples of unknown ploidy. Because allele ratios differ between samples, we set the highest and lowest median allele ratios calculated for *Catolobus* samples with known ploidy as limits. If the allele ratios of the herbarium specimens were within these limits, we assumed that they had the same ploidy.

### 2.7 STRUCTURE genetic cluster analysis

Genetic clusters of *Catolobus* samples were implemented using STRUCTURE 2.3.4 (Pritchard et al., 2000) by inferring a Bayesian clustering of SNP data. First, SNPs were generated by mapping read data to the reference target sequences using BWA mem. The alignments were sorted and indexed by Samtools. The HaplotypeCaller function of GATK v.4 was used to call variants considering ploidy level. The VariantFiltration function of GATK v.4 was used to implement hard filtering for SNPs to filter and retain high-quality SNPs. The filtration tagged vcf files were converted to STRUCTURE format using the SnipStrup pipeline (https://github.com/MarekSlenker/snipStrup) (Melichárková et al., 2020; Šlenker et al., 2021). 500 datasets were generated with a single random SNP site from each gene (1 800 exons concatenated into 730 genes) to ensure no linkage between sites. We ran K from 1 to 10 for each dataset, with a burn-in of 100 000 generations and data collection for additional 1 000 000 generations. All datasets were run with the software STRUCTURE using the admixture model and correlated allele frequencies. STRUCTURE results were averaged with CLUMPP (Jakobsson and Rosenberg, 2007) using the greedy model and visualized with Distruct (Rosenberg, 2004). Determination of the best-fit K value was based on the method of Evanno et al. (2005) using STRUCTURE HARVESTER (Earl and VonHoldt, 2012). Bayesian clustering analysis could generate a background of false genome admixture in some individuals. Here, we considered a genome to be admixed if the admixture pattern was above the 10% threshold.

To investigate the difference in SNPs (shared and unique) among samples belonging to the detected clusters in the STRUCTURE analysis, we used the Python script SnpCountCU (GitHub - JingfangSI/SnpCountCU: Count common and unique SNPs among several populations from a VCF format file). Because the number of SNPs could be affected by the number of individuals, we randomly selected six samples from each cluster. First, the Hyb-Seq reads of the samples were mapped to the merged unique Angiosperm and Brassicaceae targets (**Supplementary Table S2**) using BWA mem. Samtools was used to sort and index the alignment. GATK was used for variant identification considering ploidy level with HaplotypeCaller. Variants were filtered with GATK VariantFiltration and SelectVariants using hard filtering (‘QD < 2.0’, ‘DP < 8.0’, ‘MQ < 40.0’, and ‘FS > 60.0’). The vcf files were merged and indexed using BCFtools v.1.10.2. The merged vcf file was used for SNP counting. Singletons (one specific SNP per loci) were filtered out and common vs unique SNPs for each cluster were counted and plotted in a Venn plot using the R package VennDiagram 1.7.3. Genes that had SNPs specific to each cluster were localized to chromosomes based on the set of 22 genomic blocks in the *A. thaliana* genome (Lysak et al., 2016). For simplicity, SNPs were visualized on *Catolobus* chromosomes without species-specific rearrangements (chromosomes 1, 3, 5, 7, 9, 11, 13, and 14) using the R package chromoMap (Anand and Rodriguez Lopez, 2022). Gene positions on *Catolobus* chromosomes were estimated according to chromosomal homeology between genomes of *C. pendulus* and *A. thaliana*.

### 2.8 Phylogenomic analyses and divergence time estimation

Supercontigs generated by Hybpiper using Angiosperm and Brassicaceae targets were retrieved for *Catolobus* and accessions of *Camelina, Capsella*, and *Neslia* species and two outgroup species from tribes Arabideae (*Draba nuda*, NCBI accession no. SRR13271431) and Arabidopsideae (*A. thaliana*, target protein coding sequences extracted from the Tair-10 genome). Supercontigs were aligned using MAFFT v. 7.313 and cleaned using trimal v1.4 (-gt 0.7). Statistics for each alignment were generated using AMAS (Borowiec, 2016). Alignments less than 900 bp in length and not covered by at least 70% of samples were filtered out. In addition, overlapping genes previously detected between Angiosperm and Brassicaceae targets were removed. Supercontigs that passed filtering were concatenated and partitioned using FASconCAT-G_v1.05 (Kück and Longo, 2014). The maximum likelihood phylogenetic tree was constructed by IqTree v. 2.1.3 using the concatenated supercontigs for which the software estimated the best-fitting model for each partition (locus). Branch support was estimated using 1 000 fast bootstraps (Hoang et al., 2018) and 200 standard nonparametric bootstraps (Felsenstein, 1985). Phylogenetic trees were visualized using FigTree v1.4.4 (http://tree.bio.ed.ac.uk/software/figtree/).

Divergence times were estimated using MCMCtree software implemented in the PAML4.9e package (Yang, 2007). Baseml was first used to calculate the substitution rate using the alignment of the concatenated and filtered loci used in the phylogenetic analysis (1 176 loci covering 780 nuclear low-copy genes). We used the independent rate clock model with the gamma-Dirichlet prior including the calculated substitution rate as rgene_gamma = 1, 50, 1. The birth rate (λ), death rate (μ), and sampling fraction (ρ) were set to 1, 1, and 0, respectively. We applied two secondary calibration points. The first point was the diversification time between *Arabidopsis* and *Camelina*, which was set to ~14.6 million years ago (Mya) (Huang et al., 2016) and ~12.2 Mya (Hendriks et al., 2022). The second point was the diversification time between (*Camelina*/*Capsella*) and *Catolobus*, which was set at ~9.5 Mya and ~6.5 Mya by Huang et al. (2016) and Hendriks et al. (2022), respectively. Two separate MCMC runs were evaluated for each calibration, each for 5 million generations sampled every 50 generations after a burn-in of 500 000 iterations. Annotation and visualization of the trees was done using the R package MCMCtreeR (Puttick, 2019).

### 2.9 Chloroplast genome *de novo* assembly

GetOrganelle (Jin et al., 2020) was used to *de novo* assemble the whole chloroplast genome of *Catolobus*. Two *Catolobus* accessions (19 and 42) were assembled using GetOrganelle default parameters with K-mer values of 21, 45, 65, 85, and 105. *De novo* assembled genomes were aligned using minimap2 v.2.24, visualized in D-Genies (Cabanettes and Klopp, 2018), and inspected using Bandage (Wick et al., 2015) to verify their circularity. The two *de novo* assembled plastomes of *C. pendulus* showed no significant difference between their alignments (**Supplementary Figure S2A**). Therefore, we annotated the *de novo* assembled plastome of sample 42 (read coverage ~87.9×) as representative of the chloroplast genome of the species by GeSeq (Tillich et al., 2017). For this purpose, we selected the options to perform BLAT, HMMER, ARAGORN, tRNAscan-SE, and BLAST using the MPI-MP chloroplast database. Manual examination and blast were used to correct the annotation using the *A. thaliana* database as reference when necessary. To draw the genome, we employed EMBOSS seqret software (Madeira et al., 2022) and OGDRAW (Greiner et al., 2019). The genome size of the assembled chloroplast was 154 620 bp with a pair of inverted repeats (IR) of 26 467 bp separated by a large single-copy region (LSC) of 83 805 bp and a small single-copy region (SSC) of 17 878 bp (**Supplementary Figure S2C**, see **Supplementary Table S3** for a GFF description of the genome, NCBI accession number: OQ439752).

### 2.10 Plastome phylogenetic analysis

The plastome consensus sequences that passed filtering (<28% of ambiguity (Ns)) along with plastome sequence of *A. thaliana* (RefSeq: NC_000932.1) as outgroup were aligned using MAFFT v. 7.313 and cleaned of sites with gaps using trimal v1.4. The following samples were discarded in this analysis: *Camelina neglecta*, *C. laxa*, *D. nuda*, population samples 8, 16, 23, 25, 35, and 45. Samples kept for phylogenetic analysis of plastid data had an average coverage of off-target reads of approximately 24×. The bestfitting model of nucleotide substitution for each locus was estimated using the ModelFinder function in IqTree v. 2.1.3. Maximum likelihood (ML) phylogenetic tree was constructed using IqTree v. 2.1.3 and branch support was estimated by 1 000 fast bootstraps (Hoang et al., 2018). ML tree was annotated and visualized using Interactive Tree Of Life (iTOL) v5 (Letunic and Bork, 2021).

### 2.11 Inference of polyploidy mode

We inferred the presence and the mode of polyploidy (autopolyploidy vs allopolyploidy) in *Catolobus* using GRAMPA (Thomas et al., 2017). Briefly, GRAMPA uses an adapted least common ancestor (LCA) method that maps multi‐labeled (MUL) trees against a reference species tree. MUL trees have some identically labeled tips that will serve as representative for the polyploid genomes. Hence, in the case of allopolyploidy, GRAMPA will identify the parental lineages by supporting the non-monophyletic placement of paralogues in the MUL trees. For a better resolution in GRAMPA analysis, a simplified sampling was selected to construct the reference and gene trees. The sub-sampling included four *Catolobus* samples (38, 41, 11, and 25) along with the eight closely related species (*Camelina laxa*, *C. hispida*, *C. neglecta*, *Capsella grandiflora*, *C. orientalis*, *C. thracica*, *C. rubella*, and *Neslia paniculata*) and the outgroup *D. nuda.* Reference species tree for the selected samples was constructed using 1 183 supercontigs of low-copy genes recovered from target enrichment data of Angiosperm and Brassicaceae-specific baits. Supercontigs were aligned by MAFFT v.7.313, concatenated by FASconCAT-G_v1.05 and ML reference tree was built by IqTree v. 2.1.3. For gene trees construction, we retrieved 92 paralogous exon sequences (**Supplementary Table S4**) shared by the four *Catolobus* accessions and nine Brassicaceae accessions using the built-in Hybpiper script, paralog_retriever.py. Sequences were aligned by MAFFT v. 7.313 and cleaned using trimal v1.4 (-gt 0.7). Gene trees were constructed by IqTree v. 2.1.3 using the best fit model for each locus. GRAMPA with default parameters was run for reconciliation searches of the gene trees against the reference species tree. The most probable mode of polyploidization was inferred from the tree with the lowest parsimony score.

In addition, the ABBA-BABA test (Patterson’s D-statistic) was performed on *Capsella* and *Catolobus* samples to assess possible patterns of historical gene flow between these taxa (Green et al., 2010; Durand et al., 2011; Patterson et al., 2012). Briefly, the ABBA-BABA analysis simulates a four-taxon tree with the following relationship (((P1, P2), P3), O), where O is the output, P1 and P2 are closely related taxa, and P3 is the third in-group taxon. When the derived allele, denoted as “B”, is shared by P2 and P3, we obtain the pattern “ABBA”, while the pattern “BABA” is obtained when the derived allele is shared by P1 and P3. The D-statistic calculates the proportions of the “ABBA” and “BABA” patterns. In a scenario without introgression, this proportion must be similarly frequent. In the presence of gene flow between P2 and P3, however, the “ABBA” pattern will be more frequent than the “BABA” pattern, resulting in a D-statistic value significantly different from zero. The significance of the D-statistic value was identified using a p-value < 0.01 and a Z-score > 3 (obtained by the division of D-statistic value by the standard error) (Vargas et al., 2017). Supercontigs of low-copy genes recovered from Hyb-Seq data (**Supplementary Table S2**) for *Capsella* and *Catolobus* samples (subsample of GRAMPA analysis) were aligned with MAFFT v.7.313, cleaned with trimal v1.4 (-gt 0.7), and concatenated with FASconCAT-G_v1.05. SNPs were retrieved from the concatenated alignment using SNP-sites with the option -v to generate a vcf file (Page et al., 2016). The vcf file was assessed by the command Dtrios in D-suite v.0.5 r50 (Malinsky et al., 2021) using default parameters.

### 2.12 Habitat suitability modeling

We used habitat suitability modeling (Guisan et al., 2017) to estimate the climates suitable for *Catolobus* and its closely related genus *Capsella* during five periods: present (1979–2013 Common Era (CE)), mid-Holocene (8.3–4.2 kya), Last Glacial Maximum (LGM: ca. 21 kya), Last Interglacial (ca. 130 kya) and Pliocene (ca. 3.3 Mya). GBIF database (https://www.gbif.org) was used to retrieve data on the present-day distribution of *Catolobus pendulus* (GBIF.org, 2022a), *Capsella orientalis* (GBIF.org, 2022b), *Capsella rubella* (GBIF.org, 2022c), *Capsella grandiflora* (GBIF.org, 2022d), and *Capsella thracica* (GBIF.org, 2022e). For *C. thracica*, additional occurrence records were extracted from herbarium collections and the literature (**Supplementary Table S5**). Five bioclimatic variables were retrieved from the CHELSA (Karger et al., 2017) and PaleoClim (Brown et al., 2018) databases at 2.5 arc-minute resolution for the five periods studied. This set included bio_1 (Annual Mean Temperature), bio_4 (Temperature Seasonality), bio_15 (Precipitation Seasonality), bio_16 (Precipitation of Wettest Quarter), and bio_17 (Precipitation of Driest Quarter). Pearson pairwise correlation between variables was |*r*| < 0.8 but most variables were correlated less than 0.5. Prior to running the habitat suitability models, species occurrence records (except those for *C. thracica*) were pruned using the environmental filtering procedure (Varela et al., 2014) to avoid potential bias in the models due to uneven density of occurrence records. Models were calibrated using MaxEnt v3.4.4 (Phillips et al., 2006) with 20 000 background points distributed across Eurasia. Model settings (the combination of feature classes and regularization multiplier) were tuned using the ENMTools package (Warren et al., 2021). The best setting was selected based on the model with the lowest delta AIC value. The performance of the model for each species was assessed by the 10-fold cross-validation (4-fold for *C. thracica*) and the area under the curve (AUC; Fielding and Bell, 1997; Merow et al., 2013) value. In general, the model prediction is considered good and accurate when AUC scores are above 0.9 (Swets, 1988; Merow et al., 2013). The final models were then projected to the four historical periods using the above-mentioned paleoclimate data from the PaleoClim database (Brown et al., 2018).

## 3 Results

### 3.1 *C. pendulus* has hypotetraploid chromosome number across its distribution range

Chromosome counting in 32 populations of *C. pendulus* (nos. 1-30, 53, and 54; **Supplementary Table S1** and **Supplementary Figure S1**) revealed that all analyzed plants had the hypotetraploid chromosome number (2*n* = 30). In addition, detailed screening of chromosome numbers was performed in 60 individuals from two populations from the Russian Far East (populations 16 and 17), where diploid (2*n* = 16) and near-triploid (2*n* = 21) chromosome numbers have been previously reported (Berkutenko et al., 1984). All plants examined were hypotetraploid (2*n* = 30). The size of the holoploid genome of *C. pendulus* in populations 4, 23, 30, and 56 was estimated to be 326.8 Mb, 336.9 Mb, 331.3 Mb, and 327.0 Mb/1C, respectively. For 11 herbarium specimens (**Supplementary Table S1**), we estimated ploidy using allele frequency from Hyb-Seq data (Weiß et al., 2018; Viruel et al., 2019). For all specimens, nQuire statistical parameters (R2, delta logL, SSR, y-y slope) showed values consistent with either the triploid or tetraploid model, rejecting the diploid model (**Supplementary Table S6**). The most informative criteria for determining the ploidy level in *Catolobus* herbarium vouchers were the median allele ratio and the histograms of the allele frequency distribution. The median allele ratio in *Catolobus* specimens with counted chromosomes (2*n* = 30) ranged from 2.3 to 2.8, and correspondingly, the ratio in herbarium specimens ranged from 2.4 to 2.7 (**Supplementary Table S6**). Furthermore, the histograms of allele frequencies showed comparable patterns of distribution in all specimens examined, which, together with the median value of allele ratios, indicated that all analyzed accessions had the same ploidy level (**Supplementary Table S6**).

### 3.2 *Catolobus* genome originated through a WGD followed by descending dysploidy

**Figure 1:**
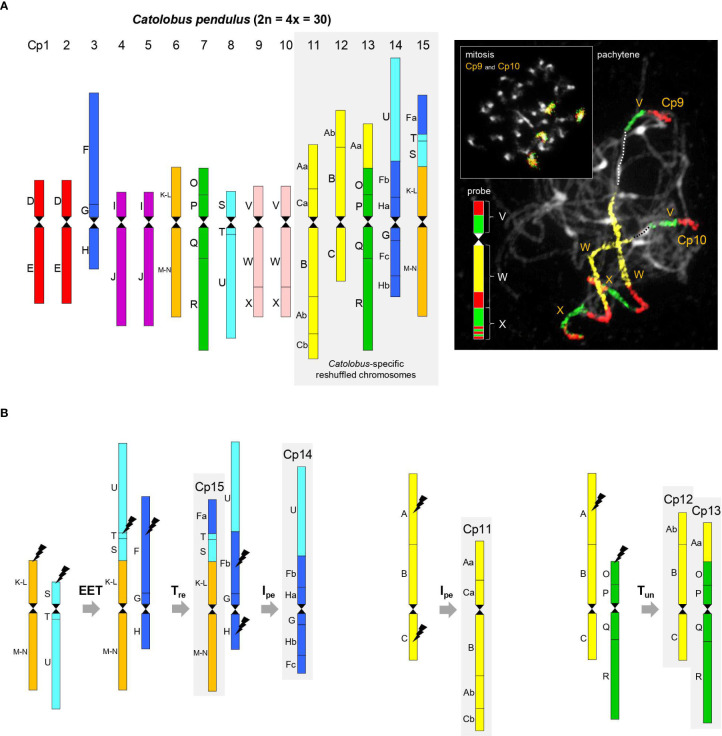
Comparative genome structure of *C. pendulus*. **(A)** The chromosome-level structure of the *Catolobus* genome based on comparative chromosome painting analysis showing the position of 44 genomic blocks on 15 *Catolobus* chromosomes (Cp1-Cp15). An example of comparative chromosome painting - chromosomes Cp9 and Cp10 in mitosis and meiosis were painted using *A. thaliana* BAC contigs representing ancestral genomic blocks V, W, and X, respectively. **(B)** A parsimonious reconstruction of the origin of the five rearranged chromosomes. Color coding and capital letters (A-X) correspond to the eight chromosomes and 22 genomic blocks of ACK. Hourglass symbols mark the centromeres. EET: end-to-end translocation, T_re_: reciprocal translocation, T_un_: unequal translocation, I_pe_: pericentric inversion.

To analyze the genome structure of *C. pendulus*, we used comparative chromosome painting (CCP) based on the localization of contigs of chromosome-specific BACs (Bacterial Artificial Clones) of *A. thaliana* on pachytene chromosomes. Painting probes were designed to reflect the system of 22 genomic blocks (GBs) of the Ancestral Crucifer Karyotype (ACK, *n* = 8, AK1-AK8; Schranz et al., 2006; Lysak et al., 2016), which is believed to be the ancestral genome of all Camelinodae tribes (Lysak et al., 2016). Genome structure was investigated in seven populations (4, 6, 7, 8, 20, 24, and 30; **Supplementary Table S1** and **Supplementary Figure S1**). In all analyzed individuals, all 22 GBs were clearly identified in two copies within the meiotic chromosome complement, clearly indicating the tetraploid origin of the *Catolobus* genome (**Figure 1A**).

Ten of 15 *Catolobus* chromosomes (Cp1 to Cp15) mirror the structure of the ancestral (AK) chromosomes: two homeologs of AK2 (Cp1 and Cp2), AK3 (Cp3), two homeologs of AK4 (Cp4 and Cp5), AK5 (Cp6), AK6 (Cp7), AK7 (Cp8), and two homeologs of AK8 (Cp9 and Cp10) (**Figures 1A, B**). Descending dysploidy in *Catolobus* was mediated by an end-to-end translocation between ancestral chromosomes AK5 and AK7. The resulting fusion chromosome consisted of the U+T+S+(K-L)+(M-N) blocks and the AK5 centromere, while the AK7 centromere was eliminated/inactivated (**Figure 1B**). Reciprocal translocation between the fusion chromosome and AK3 resulted in the formation of chromosomes Cp15 [GBs Fa+T+S+(K-L)+(M-N)] and the “U+Fb+G+H” product. Finally, an 8.21-Mb pericentric inversion of “U+Fb+G+H” with breakpoints within blocks Fb [between AT3G60970 (MRP15) and AT3G14220 (MLE3)] and H [between AT2G18900 (F19F24) and AT2G19000 (T20K24)] shaped the structure of Cp14 (U+Fb+Ha+G+Fc+Hb). Chromosome Cp11 (Aa+Ca+B+Ab+Cb) resembles ancestral chromosome AK1, which underwent an 15.2-Mb pericentric inversion with breakpoints within blocks A [between AT1G12180 (T28K15) and AT1G12660 (T12C24)] and C [between AT1G52240 (F9I5) and AT1G52450 (F6D8)]. Chromosomes Cp12 (Ab+B+C) and Cp13 (Aa+O+P+Q+R) originated by an unequal reciprocal translocation between chromosome AK1, with breakpoints within blocks A [between AT1G13500 (F13B4) and AT1G14220 (F7A19)], and subtelomeric region of the AK6 upper arm (**Figure 1B**).

With the exception of a single 3.54-Mb pericentric inversion on chromosome Cp13 in population 20 (**Supplementary Figure S3A**), all 15 chromosomes in the seven populations examined had the same structure. Limited variation among populations in the number and position of ribosomal RNA genes (rDNA) confirmed the stability of the *Catolobus* genome across its range: in 25 populations, the 35S rDNA was localized as terminal nucleolar organizer regions on the short arms of chromosomes Cp3 and Cp8, whereas the 5S rDNA loci were found in the pericentromeric regions of chromosomes Cp1 and Cp7. In plants representing six populations from Japan (24–29), an additional 35S rDNA locus was detected, whereas only one 5S rDNA locus was found. Only one 5S rDNA locus was also observed in population 4 from Russia (**Supplementary Figure S3B**).

In summary, our data show that the *Catolobus* genome originated by a WGD involving four, structurally identical, ACK-like genomes (*n* = 8). Post-polyploid descending dysploidy from *n* = 16 to *n* = 15 was mediated by an end-to-end translocation and followed by complex chromosome repatterning including one reciprocal translocation, two pericentric inversions and one unequal translocation (**Figure 1B**).

### 3.3 Two gene pools of *C. pendulus*

On average, target enrichment sequencing produced approximately 3.06 million raw reads per sample. After trimming adapters and removing low-quality reads, an average of ~2.58 million reads per sample remained. Clean reads mapped on average 20% to Angiosperm target sequences and 50% to Brassicaceae target sequences. Overall, 70% of reads were mapped to the target sequences, providing an average total coverage of the target sequences of ~148x. Statistics on the success of Brassicaceae and Angiosperm target enrichment can be found in **Supplementary Table S7**.

**Figure 2:**
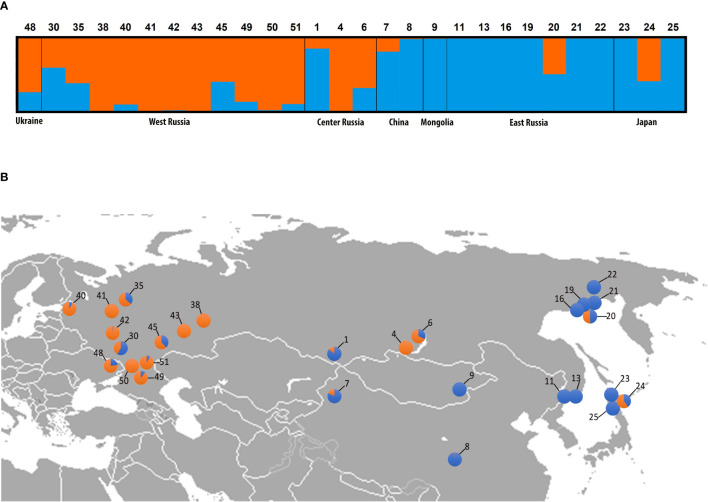
Genetic clustering of *Catolobus* populations based on 730 low-copy nuclear genes. **(A)** Bayesian clustering graph for the optimal genetic partition (K=2) resulting from the STRUCTURE analysis. Blue and orange colors assign samples to genetic cluster I or II, or both clusters, respectively; numbers refer to the populations analyzed (**Supplementary Table S1**). **(B)** Geographic distribution of analyzed populations and genetic composition of individuals analyzed in **(A)**. Population codes correspond to those in **(A)**.

The Bayesian clustering of 500 datasets harboring high-quality single-nucleotide polymorphism (SNP) data from Brassicaceae-specific targets (1 800 exons concatenated into 730 genes) successfully identified K=2 as the optimal genetic partition (**Supplementary Table S8**). STRUCTURE results clearly distinguished three classes of *Catolobus* samples (**Figure 2A**): (i) individuals belonging to pure genetic cluster I (accessions 8, 9, 11, 13, 16, 19, 21, 22, 23, and 25) (ii) individuals belonging to pure cluster II (4, 38, 41, 42, 43, and 50), and (iii) individuals with significant admixture between clusters I and II (1, 6, 7, 20, 24, 30, 35, 40, 45, 48, 49, and 51). Populations belonging to the two pure genetic clusters were geographically separated (**Figure 2B**). Individuals from cluster II were mainly restricted to the European part of Russia, except for the population in the center of the species’ range (population 4; Buryatia, Russia). Accessions from cluster I were found in central/eastern Asia and had a wider latitudinal distribution than cluster II. However, individuals with an admixed genetic pattern are scattered throughout the entire distribution range.

SNP count analysis of six randomly selected individuals from clusters I and II (**Supplementary Table S9**) showed that the two identified clusters shared 56 169 SNPs. Cluster I had more specific SNPs (9 331 unique SNPs) than cluster II (3 738 unique SNPs) (**Supplementary Figure S4**). The specific SNPs for both clusters were found to be randomly distributed throughout the studied part of the genome (**Supplementary Table S10** and **Supplementary Figure S4**).

### 3.4 Phylogenomic analysis corroborated the sister relationship of *Catolobus* and *Capsella*, and two intraspecific clades in *Catolobus*

**Figure 3:**
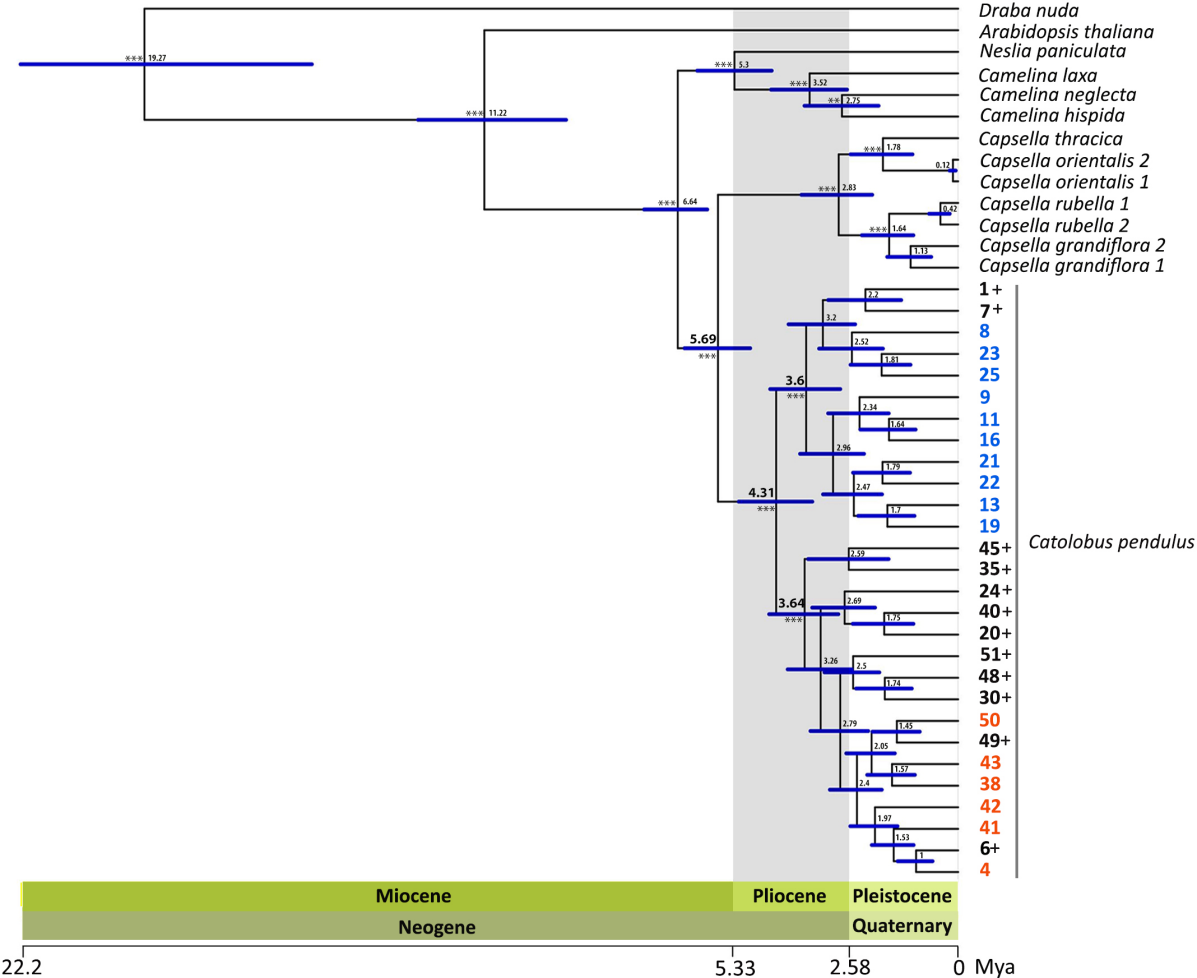
Inferred phylogenetic relationships between *C. pendulus* populations and within the tribe Camelineae. The dated ML tree was constructed using Hyb-Seq data for 780 nuclear low-copy genes and calibrated based on Hendriks et al. (2022). The numbers at the nodes represent median ages in millions of years, and the blue bars refer to the 95% confidence intervals for the divergence times. The high significance level (bootstrap > 99) of the major nodes is indicated by asterisks. The blue and orange accessions belonged to clusters I and II, respectively, in the genetic clustering analysis (STRUCTURE results). The black colored accessions followed by the sign “+” represent the accessions that had an admixed pattern between clusters I and II.

Phylogenetic analysis was based on 1 176 loci covering 780 nuclear low-copy genes and a concatenated alignment length of 1 822 965 bp. Maximum likelihood (ML) analysis based on the concatenated and partitioned alignments with 1 000 fast bootstraps (BS) and 200 nonparametric bootstraps resulted in a well-supported tree (**Supplementary Figures S5, S6**). Within *Catolobus*, the ML tree grouped the studied populations into two well-supported clades (**Figure 3** and **Supplementary Figures S5, S6**). One clade included all *Catolobus* populations assigned to genetic cluster I, as well as the accessions that showed an admixed pattern in the STRUCTURE analysis where cluster I was dominant. The other clade consisted of the populations assigned to cluster II (**Figure 3** and **Supplementary Figures S5, S6**) and the accessions that showed an admixed pattern with dominance of cluster II. Two accessions (20 and 30) in the latter clade differed by a slight dominance for cluster I in the STRUCTURE analysis. The closest relatives of *C. pendulus* based on our ML tree were *Capsella* species. Within *Capsella*, *C. orientalis* and *C. thracica* were sisters to *C. rubella* and *C. grandiflora.* The *Catolobus*/*Capsella* clade proved to be sister to the clade comprising *Camelina* and *Neslia* (**Figure 3** and **Supplementary Figures S5, S6**).

The structure and gene orientation of the *Catolobus* chloroplast genome were similar to those previously annotated in Camelineae (Wu, 2016; Wu and Ma, 2016; Omelchenko et al., 2020; Brock et al., 2022). Our analysis revealed three main features that support a closer relationship between the plastomes of *Catolobus* and *Capsella* compared to *Camelina* species. First, the size of the chloroplast genome in *Catolobus* and *Capsella* is comparable and slightly larger than in all *Camelina* plastomes examined. Second, 78 protein-coding genes were annotated in *Catolobus* and *Capsella*, in contrast to 79 protein-coding genes in *Camelina* plastomes. Third, all *Camelina* plastomes examined lack a functional copy of *rps16* gene (Brock et al., 2022), whereas this gene was present in the same position and orientation in all sequenced plastomes of *Catolobus* and *Capsella* (Wu, 2016; Wu and Ma, 2016; Omelchenko et al., 2020 and present results). Based on the extracted chloroplast consensus sequences from 22 *Catolobus* accessions, a ML tree was constructed based on a total of 56 670 bp. The topology of the plastome tree was congruent with the nuclear tree, with high supports (BS > 99) (**Supplementary Figure S2B**). The analysis revealed strong support (BS = 99) for the sister relationship between *Catolobus* and *Capsella*. However, no phylogenetic differentiation was found between *Catolobus* accessions, consistent with the expected uniformity of the maternal genome at the intraspecific level.

### 3.5 The most probable autopolyploid origin of the tetraploid *Catolobus* genome

We inferred the presence and type of polyploidy (autopolyploidy vs. allopolyploidy) in *Catolobus* using GRAMPA. In the case of allopolyploidy, GRAMPA identifies parental lineages by supporting non-monophyletic placement of paralogs in the multi‐labeled trees (Thomas et al., 2017). The best GRAMPA tree with the lowest parsimony value showed that the *Catolobus* accessions from cluster I (in blue in **Supplementary Figure S7B**) have both polyploid lineages within the species clade. The second best tree also showed the same scenario for the cluster II (in orange in **Supplementary Figure S7C**). Therefore, the GRAMPA reconciliation analyzes suggest that the hypotetraploid *C. pendulus* had an autopolyploid origin.

To assess historical introgressions between *Catolobus* and *Capsella*, we used the ABBA-BABA analysis to measure Patterson’s D‐statistic. All trios including *C. pendulus* had low and not supported values of D-statistic (D-statistic < 0.0878, Z-score < 2.117, p-value > 0.034; **Supplementary Table S11**). This result supports a scenario without gene flow between *Catolobus* and *Capsella* and, in agreement with results of GRAMPA, suggests a highly probable autopolyploid origin of *C. pendulus*. On the other hand, in the trios containing *Capsella grandiflora*, *C. rubella*, and *C. thracica* significantly high values of the D-statistic were estimated (D-statistic > 0.315, Z-score > 6.07, p-value < 1.24E-09; **Supplementary Table S11**), suggesting gene flow between these taxa. This is congruent with previous study identifying *C. grandiflora* and *C. rubella*, together with *C. bursa-pastoris*, as parental genomes of the allotetraploid *C. thracica* (Žerdoner Čalasan et al., 2021).

### 3.6 Populations of *C. pendulus* diverged earlier than the MRCA of *Capsella*

Molecular dating estimates yielded an overall slightly older age for all nodes based on the calibration of Huang et al. (2016) (hereafter referred to as Cal I) than estimates based on Hendriks et al. (2022) (hereafter referred to as Cal II) (**Figure 3** and **Supplementary Figure S8**). The time estimate based on both calibration references suggests that the most recent ancestor (MRCA) of *Catolobus* populations (mean age Cal I: 5.59 Mya, Cal II: 4.31 Mya) emerged earlier than the MRCA of *Capsella* (mean age Cal I: 3.67 Mya, Cal II: 2.83 Mya) and *Camelina* species (mean age Cal I: 4.79 Mya, Cal II: 3.52 Mya). Furthermore, this analysis revealed that the separation between *Catolobus* and *Capsella* occurred in the late Miocene (Cal I: 7.75 (6.57–8.85) Mya, Cal II: 5.69 (4.93–6.48) Mya). Diversification within the *Camelina* and *Catolobus* clades was estimated to have occurred in the Pliocene. Interestingly, during this period the two main clades, corresponding to the identified genetic clusters I and II in *Catolobus*, were separated and diversified at about the same time. On the basis of Cal I, clusters I and II emerged at around 4.63 (3.51–5.83) Mya and 4.68 (3.56–5.86) Mya, respectively (**Supplementary Figure S8**). Somewhat later, based on Cal II, the estimated age was set at approximately 3.6 (2.79–4.46) Mya for cluster I and 3.64 (2.84–4.48) Mya for cluster II (**Figure 3** and **Supplementary Figure S8**).

### 3.7 Historical shifts in climatic suitability for *C. pendulus* and *Capsella* species

**Figure 4:**
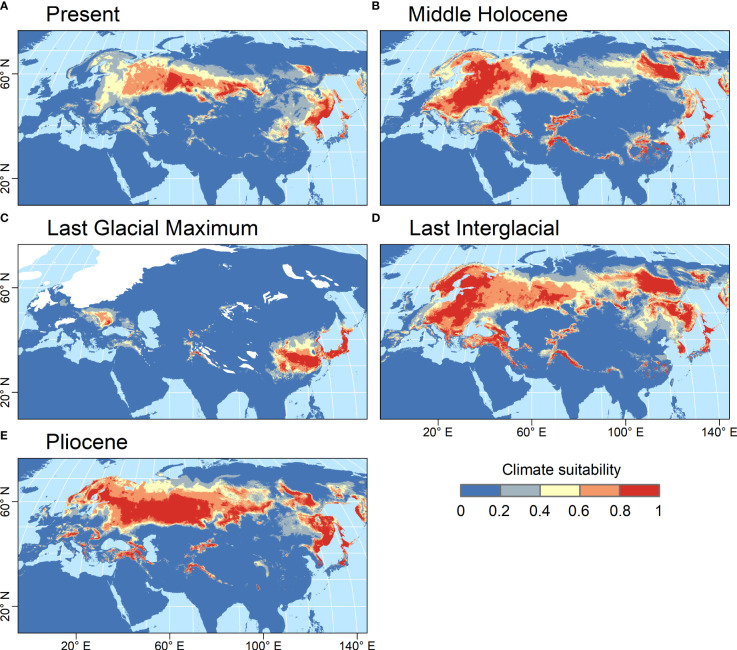
Climate suitability predicted by the MaxEnt model for *C. pendulus* for five time periods. **(A)** Present (1979 - 2013 CE), **(B)** Middle Holocene (4.2 - 8.3 kya), **(C)** Last Glacial Maximum (ca. 21 kya), **(D)** Last Interglacial (ca. 130 kya), and **(E)** Pliocene (ca. 3.3 Mya). Climate suitability is scaled between 0 (dark blue) and 1 (red), with higher values indicating more suitable conditions. The estimated extent of the LGM ice sheets is indicated by white color.

The habitat suitability model developed for *Catolobus* successfully captured its current distribution. The mean value (± std. dev.) of area under the curve resulting from 10-fold cross validation was high (AUC=0.933 ± 0.009), reflecting the high accuracy of the model (**Supplementary Table S12**). The variable that most contributed to the model was annual mean temperature (bio_1, 48.4%), followed by precipitation of the wettest quarter (bio_16, 20.6%). The areas with the most suitable climates, predicted by the best-fit model, extended from eastern Europe to eastern Asia roughly along the 55^th^ parallel (**Figure 4A**). In the warm periods (mid-Holocene, Last Interglacial and Pliocene), the climatically suitable areas extended northwards to northern Scandinavia and north-eastern Russia, westwards to central Europe, and to higher elevations (**Figures 4B, D, E**). In all these time periods, the models suggested a possible division of the *Catolobus* range into a western and an eastern part due to less suitable conditions around 100°E. In contrast, areas with suitable climates strongly contracted during the Last Glacial Maximum (LGM), suggesting an LGM bottleneck (**Figure 4C**). The model showed that suitable habitats during the LGM were mainly in eastern China, Korea, Japan and east of the Carpathians.

We applied the same strategy to *Capsella*, the sister genus of *Catolobus*, to investigate the suitable climates during past periods (**Supplementary Figure S9**). The AUC values for *Capsella* species, reflecting the accuracy of the model, were all higher than 0.9 (*C. grandiflora*= 0.985 ± 0.012; *C. orientalis* = 0.939 ± 0.028; *C. rubella*= 0.991 ± 0.001; *C. thracica* = 0.972 ± 0.029, **Supplementary Table S12**). The contribution of environmental variables to the Maxent model showed differences among *Capsella* species. Temperature seasonality (bio_4) contributed most to the models for *C. rubella* (29.8%) and *C. grandiflora* (37.9%), which avoid continental climates. Annual mean temperature (bio_1) contributed most to the model for *C. orientalis* (35.8%), while Precipitation Seasonality (bio_15) contributed largely to the model of *C. thracica* (67.9%). Based on our molecular dating (**Figure 3**) and published data, the divergence times of the studied *Capsella* species were assumed to be younger than the Pliocene (ca. 2 Mya). For this reason, we projected the habitat suitability models only to periods from the present to the Last Interglacial (**Supplementary Figure S9**). Our models suggested that the most suitable climates for both *C. rubell*a and *C. grandiflora* were located in oceanic western Europe during the warm periods. On the other hand, both species had suitable habitats in the Mediterranean during the LGM. For *C. orientalis*, the model suggested the most suitable areas in the dry continental climates of eastern Europe and western Asia during the warm periods. In the LGM, the suitable climates contracted to a narrow zone extending along the 45^th^ parallel from the Carpathian Basin to the west coast of the Caspian Sea. *C. thracica* is endemic to the southeastern part of the Balkan Peninsula, and the model suggests that the climate in this region has been relatively stable at least since the LGM.

## 4 Discussion

### 4.1 The unispecific *Catolobus* is closely related to *Capsella*, *Camelina*, and *Neslia*

Until 20 years ago, *C. pendulus* was included in the genus *Arabis* (*A. pendula*). However, phylogenetic analyzes have shown that the species is actually related to *Capsella* and the previously broadly defined Camelineae (O’Kane and Al-Shehbaz, 2003) and deserves generic status (Al-Shehbaz, 2005). Several other studies of Camelineae species rejected the monophyly of the tribe and mostly identified three subclades within the broadly defined Camelineae (Bailey et al., 2006; Koch et al., 2007; Lysak et al., 2009; Hohmann et al., 2015; Huang et al., 2016; Žerdoner Čalasan et al., 2019): (*Camelina*, *Capsella*, *Catolobus*, and *Neslia*), *Arabidopsis*, and (*Chrysochamela* and *Pseudoarabidopsis*). Here, using the Hyb-Seq data from two bait sets, we confirmed that genera *Camelina*, *Capsella*, *Catolobus*, and *Neslia* are closely related and formed a clade distant from *Arabidopsis*. This phylogenetic separation is formalized by German et al. (2023), in which *Camelina* (8 species), *Capsella* (5 spp.), *Catolobus* (1 sp.), and *Neslia* (2 spp.) formed the redefined Camelineae, while *Arabidopsis* is placed in the unigeneric Arabidopsideae. In Camelineae, *Catolobus* and *Capsella* have a sister relationship (**Figure 3**), but the species of the two genera differ in their ecological preferences, habit, and fruit and seed morphology. The fruits of *Catolobus* are linear siliques 4 to 10 cm long (at least 25 folds longer than wide), and latiseptate (flattened parallel to the septum), with 70–110 ovules per ovary and flat, winged (at least distally) seeds with accumbent cotyledons, whereas the silicles of *Capsella* are much smaller (subequal or up to two folds longer than wide), usually obcordate to obtriangular, and compressed at the right angle to the septum (e.g., angustiseptate), with 12–40 ovules per ovary, plump and wingless seeds with incumbent cotyledons (Zhou et al., 2001). While linear siliques found in many crucifer groups were maintained in *Catolobus*, the flat and obcordate fruit structure evolved in the MRCA of *Capsella*, and spherical-fruit structures evolved in *Camelina* and *Neslia* (Dong and Østergaard, 2019, and authors’ compilation).

### 4.2 The hypotetraploid chromosome number in *Catolobus* resulted from post-polyploid rediploidization

Since the ancestral base chromosome number in the Camelineae and other tribes of crucifer Lineage I (Camelinodae sensu German et al., 2023) was *x* = 8 (e.g., Lysak et al., 2016), the ancestral autotetraploid *Catolobus* genome combined four *n* = 8, ACK-like, genomes (2*n* = 4*x* = 32). The 30 chromosomes in the present *Catolobus* could be attributed to fixed aneuploidy (*n* - 1) or descending dysploidy by one fusion chromosome (*n* = 16 → *n* = 15). Interestingly, we have shown that post-polyploid diploidization in *C. pendulus* was associated with complex rearrangements involving six non-homologous chromosomes, although chromosome number was actually reduced only by one end-to end translocation associated with centromere elimination. The 15-chromosome genome was subsequently shaped by five rearrangements that included one reciprocal translocation, one unequal translocation and two pericentric inversions (**Figure 1**). This implies that the 15-chromosome hypotetraploid genome containing the fusion chromosome may have existed for some time and that later translocations and inversions shuffled the 15-chromosome genome before its successful spread over most of temperate Eurasia.

### 4.3 The tetraploid *Catolobus* genome is older and more diploidized than younger allotetraploid genomes in *Capsella*

End-to-end translocation (EET) is the most common mechanism of post-polyploid chromosome number reduction in tetraploid crucifer genomes (Mandáková and Lysak, 2018). An EET is the merger of entire two non-homologous chromosomes by recombination within the (sub)telomeric regions of the two chromosomes. Since the resulting fusion chromosome is dicentric, one of the two centromeres must become inactive to restore the functionality of the fusion chromosome in mitosis and meiosis. Similar to *Catolobus*, EET-based descending dysploidy has been documented as a diploidization mechanism in a number of polyploid crucifer taxa, e.g., in *Cardamine cordifolia* (Mandáková et al., 2016), Microlepidieae (Mandáková et al., 2010a; Mandáková et al., 2010b), or in *Pugionium* (Hu et al., 2021). Because the pace of chromosomal diploidization can vary even within a single polyploid clade (e.g., Mandáková et al., 2017; Mandáková and Lysak, 2018; Zuo et al., 2022), it is quite difficult to establish credible relationships between the age of polyploid genomes and the pace and extent of their diploidization. However, the dated phylogenetic trees and comparison of polyploid *Catolobus* and *Capsella* genomes allow us to draw some conclusions with relative confidence.

Earlier (Žerdoner Čalasan et al., 2019) and our divergence time estimates date the divergence of *Capsella* and *Catolobus* to the Miocene-Pliocene transition (5 to 4 Mya). During the Pliocene, *Capsella* split into an eastern and a western lineage (Žerdoner Čalasan et al., 2021), and the diploid genomes of the two lineages hybridized to form the allotetraploid genome of *Capsella bursa-pastoris* more than once, about 300 000 to 100 000 ya (Douglas et al., 2015) or 120 000 ya (Žerdoner Čalasan et al., 2021). More recently (several thousand years ago), the allotetraploid *C. thracica* arose from hybridization between *C. bursa-pastoris* and *C. grandiflora*/*rubella* (Žerdoner Čalasan et al., 2021). The eutetraploid chromosome number of *C. bursa-pastoris* (2*n* = 32) and subgenome phasing have not provided strong evidence for structural post-polyploid diploidization of this allotetraploid genome (Douglas et al., 2015; Kryvokhyzha et al., 2019). This is in stark contrast to the genome reshuffling including descending dysploidy in *Catolobus* described here. Given the extent of diploidization, involving one-third of the ancestral chromosomes, and the wide Eurasian range of *C. pendulus*, we conclude that the tetraploid genome of *Catolobus* arose earlier than the allotetraploid *C. bursa-pastoris* genome. Since the split between the western and eastern hypotetraploid *Catolobus* populations was dated to ~4 Mya, we conclude that the tetraploid *Catolobus* arose approximately between 5 and 4 Mya. Although the polyploid genomes in both genera arose from independent mergers of similar diploid (ACK-like) genomes, diploidization of the *Catolobus* genome is more advanced due to its older age (**Figure 3**).

### 4.4 Biogeographic dynamics of *Catolobus* and *Capsella*

We modeled potential habitats of *Catolobus* from the Pliocene to the present to understand the evolution-based distribution of its only species and to identify possible ecogeographic relationships with the divergence of the MRCA of *Catolobus* and *Capsella*. Based on Maxent simulations, we assume that *Catolobus* was widespread during all time periods studied. With the exception of the LGM (20 000 to 30 000 years ago), during which *Catolobus* appeared to retreat mainly to eastern China, Korea, and Japan (**Figure 4C**). The LGM shaped the distribution of numerous plants due to ice expansion and severe cooling (e.g., Douda et al., 2014; Delmas et al., 2022; Divíšek et al., 2022; Patton et al., 2022). Unfortunately, we could not estimate the timing of the polyploidization event due to the lack of a diploid cytotype. Nevertheless, we suspect that WGD occurred early during *Catolobus* diversification. We base our assumption on two criteria: (i) the wide distribution of the hypotetraploid *Catolobus* and (ii) chromosomal rearrangements shared by all hypotetraploid populations examined. Along these lines, we propose three hypothetical scenarios to explain the current distribution of the hypotetraploid *Catolobus* under the influence of the LGM (**Supplementary Figure S10**). In all scenarios described below, we assume that the polyploidization event occurred long before the LGM. The first two scenarios (**Supplementary Figures S10A, B**) assume that diploid and tetraploid cytotypes were comparably abundant before the LGM. The severe bottleneck during the LGM may have affected both cytotypes to the same extent (**Supplementary Figure S10A**) or may have been harder for diploids than for tetraploids, and recovery after the LGM was more successful for tetraploids (**Supplementary Figure S10B**). The third scenario (**Supplementary Figure S10C**) suggests that both the 2*x* and 4*x* cytotypes occurred unevenly before the LGM bottleneck with the dominance of tetraploids. The intraspecific genetic differentiation of the tetraploid genome during the Miocene-Pliocene transition suggests its wide Eurasian distribution prior to the LGM, which was then restored and likely enhanced during the post-LGM global warming. Moreover, the longitudinal genetic differentiation found in *Catolobus* may be due to climate changes during the Pliocene. During this time, Earth’s climate became cooler and drier and also more seasonal than in the Miocene. Increasing aridity in Central Asia, as documented in several studies (Shen et al., 2018; Su et al., 2019; Gallagher et al., 2021), may have led to a division of the range of *Catolobus* into western and eastern parts, and thus to genetic differentiation of the separated populations.

In addition, *Catolobus* showed a much wider distribution than *Capsella* species during all periods studied. In particular, *C. grandiflora* and *C. thracica* are currently geographically more confined than other *Capsella* species, to the southern Balkans and Bulgaria plus adjacent Turkey, respectively (Jalas et al., 1996; Güzel, 2022), in accord with published ecological niche simulations (Han et al., 2015). The wider distribution of *C. pendulus* may reflect the earlier emergence of the tetraploid *Catolobus* genome compared to more recent speciation events in *Capsella*. Despite the wide distribution of *Catolobus* during the time periods studied, Maxent simulations revealed at least partial geographic separation of *Catolobus* and *Capsella* species, with *Catolobus* having suitable habitats farther north and in more continental climates than *Capsella* species (**Figure 4** and **Supplementary Figure S9**). This is consistent with the current ecological optimum for *Catolobus* in the forests of continental Eurasia, while most *Capsella* species prefer sunnier, warmer, and drier treeless habitats.

### 4.5 Intraspecific genetic variation in *Catolobus* does not stem from the LGM bottleneck

In the last decade, target enrichment has been widely used in phylogenetic studies due to its cost-effectiveness (e.g., Weitemier et al., 2014; Herrando-Moraira et al., 2019; Straub et al., 2020; Šlenker et al., 2021). Merging universal and family-specific bait sets showed a great advantage over developing new baits, especially when combining datasets generated independently (Johnson et al., 2019; Larridon et al., 2020; Shah et al., 2021). Recently, Hendriks et al. (2021; 2022) successfully combined Angiosperm and Brassicaceae bait sets to resolve most problematic clades in the Brassicaceae. Here, we followed the same strategy at the population level in *C. pendulus*. Although custom genus-specific baits are recommended for population-level studies (Villaverde et al., 2018), several studies have successfully used universal baits (e.g., Van Andel et al., 2019; Slimp et al., 2021; Yardeni et al., 2022). Similarly, sufficient variations in *Catolobus* at the population level were successfully extracted from supercontigs (target exons + flanking intron regions) of the universal Angio353 and the Brassicaceae-specific baits. The Hyb-Seq data successfully revealed two main genetic clusters within *Catolobus* (**Figure 2**). Although the high number of shared SNPs (>56 000) between the two clusters was expected, cluster I had about 2.4 times more unique SNPs than cluster II (**Supplementary Figure S4**), which may be related to the broad latitudinal distribution of population samples from cluster I compared with those from cluster II. Most accessions (12) showed an admixed profile between the two gene pools (**Figure 2**), indicating hybridization between the two gene pools. The two identified genetic clusters were also recovered as two major clades in the nuclear phylogenetic tree (**Figure 3** and **Supplementary Figures S5, S6**). Because the divergence of the two intraspecific clades was estimated to be approximately 4 Mya, the genetic variation is not due to refugial isolation during the LGM.

### 4.6 The continuing quest for the diploid?

Surprisingly, the chromosome number of the widely distributed *C. pendulus* was known from only two published studies until our study. The chromosome number of 2*n* = 21 was found in two specimens from eastern Russia (Magadan city and Khabarovsk region; Berkutenko and Gurzenkov, 1976). Later, both diploid (2*n* = 16) and near triploid (2*n* = 21) chromosome numbers were reported from Magadan by the same authors (Berkutenko et al., 1984). The diploid chromosome number was counted in two collections from the towns of Ussurijsk and Novoshahtinskiy, north of Vladivostok (Probatova, 2014). As near-triploid plants showed normal fertility, Berkutenko and colleagues speculated on the apomictic mode of reproduction of these plants. Despite our search for *Catolobus* populations from the same regions where diploid and near-triploid plants were reported, all counts from the Russian Far East and the entire range of the species represented only the hypotetraploid chromosome number (2*n* = 30). Because the morphology of the species is characteristic, it is unlikely that the analyzed specimens were misidentified, and although some miscounts cannot be excluded, the existence of the diploid cytotype is a plausible option that requires further validation. The near-triploid chromosome numbers can be tentatively attributed to interploid hybrids with 2*n* = 23 (2*n* = 16 × 2*n* = 30), whose fertility is likely to be compromised if they do not reproduce apomictically. If diploid plants occur in eastern Russia, they represent a surviving minor (relict) cytotype within the dominant autotetraploid populations. Future targeted studies should verify the existence of the diploid *Catolobus* genome as a valuable complement for comparative analyzes with diploid *Capsella* species.

## Data availability statement

The original contributions presented in the study are publicly available. Hyb-Seq raw data are available on NCBI Sequence Read Archive (SRA) under the BioProject PRJNA930157. The GenBank accession number of the *de novo* assembled chloroplast genome of *Catolobus pendulus* is OQ439752. Data used for ecological niche modeling in this study were retrieved from the GBIF database for: *Catolobus pendulus* at https://doi.org/10.15468/dl.ha6uuu; *Capsella orientalis* at https://doi.org/10.15468/dl.akbser; *Capsella rubella* at https://doi.org/10.15468/dl.hjpcjd; *Capsella grandiflora* at https://doi.org/10.15468/dl.39538u; *Capsella thracica* at https://doi.org/10.15468/dl.j52scr. Herbarium vouchers of *Catolobus* accessions were deposited at the Herbarium of Masaryk University (BRNU); voucher numbers are listed in the **Supplementary Table S1**.

## Author contributions

ML conceived the project. PF conducted the phylogenomic analyzes. TM performed the cytogenomic analyzes. PF and JD performed ecological niche modeling. PF, TM, JD, and ML analyzed the data. HK and DG provided plant material and data on ecology and biogeography. PF, TM, JD, and ML drafted the manuscript. All authors contributed to the article and approved the submitted version.
