# Supplementary material for: The evolution of the hypotetraploid Catolobus pendulus genome – the poorly known sister species of Capsella
Source: Front Plant Sci. 2023 May 8;14:1165140. doi: 10.3389/fpls.2023.1165140 (PMC10200890; doi:10.3389/fpls.2023.1165140)
Supplement: Supplementary file 1 [file DataSheet_1.pdf]

Supplementary Material

1 Supplementary Figures and Tables

1.1 Supplementary Figures

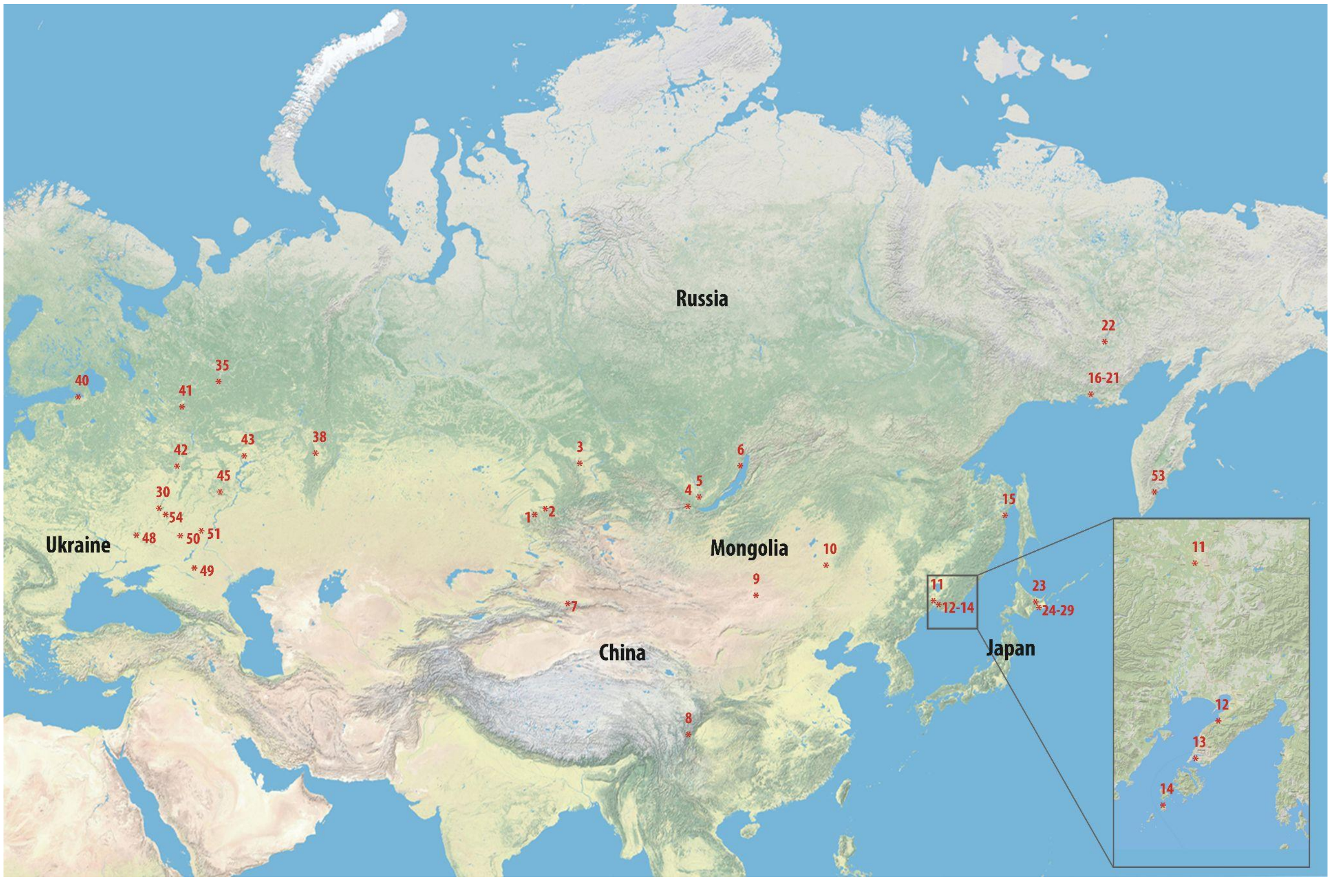

Figure S1. Distribution map of studied populations of *C. pendulus*.

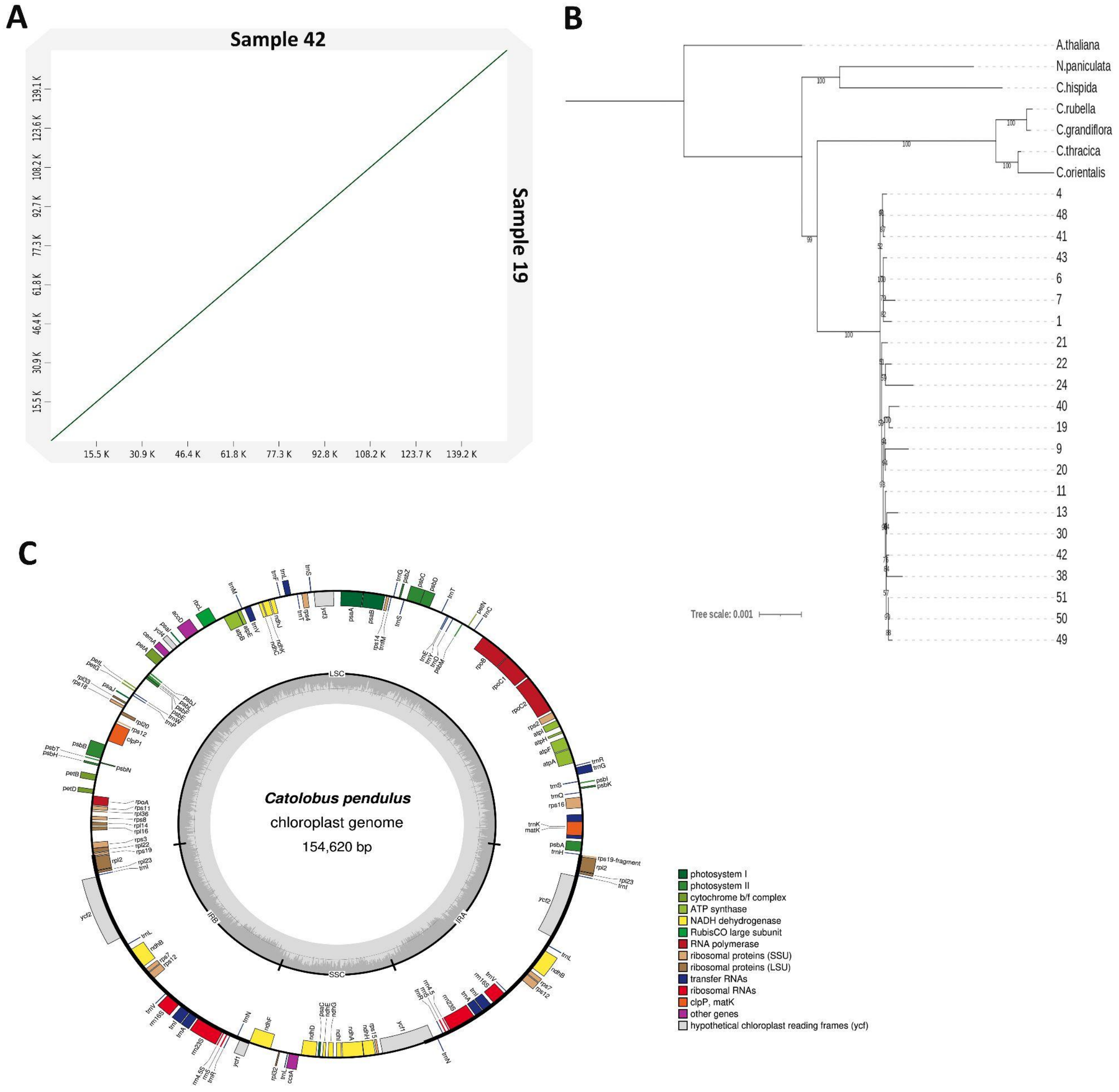

Figure S2. Plastome genome of *C. pendulus*. (A) Alignment between *de novo* assembled chloroplast genomes of accessions 19 (belongs to cluster I) and 42 (cluster II). (B) Maximum likelihood phylogeny of the chloroplast consensus sequence of *Catolobus* and related Camelineae species. Numbers at nodes correspond to bootstrap supports of branches. (C) Gene map of the *Catolobus* chloroplast genome. The color codes represent the functional groups of the genes. The circle in the center of the map illustrates the GC content in dark gray and the AT content in light gray.

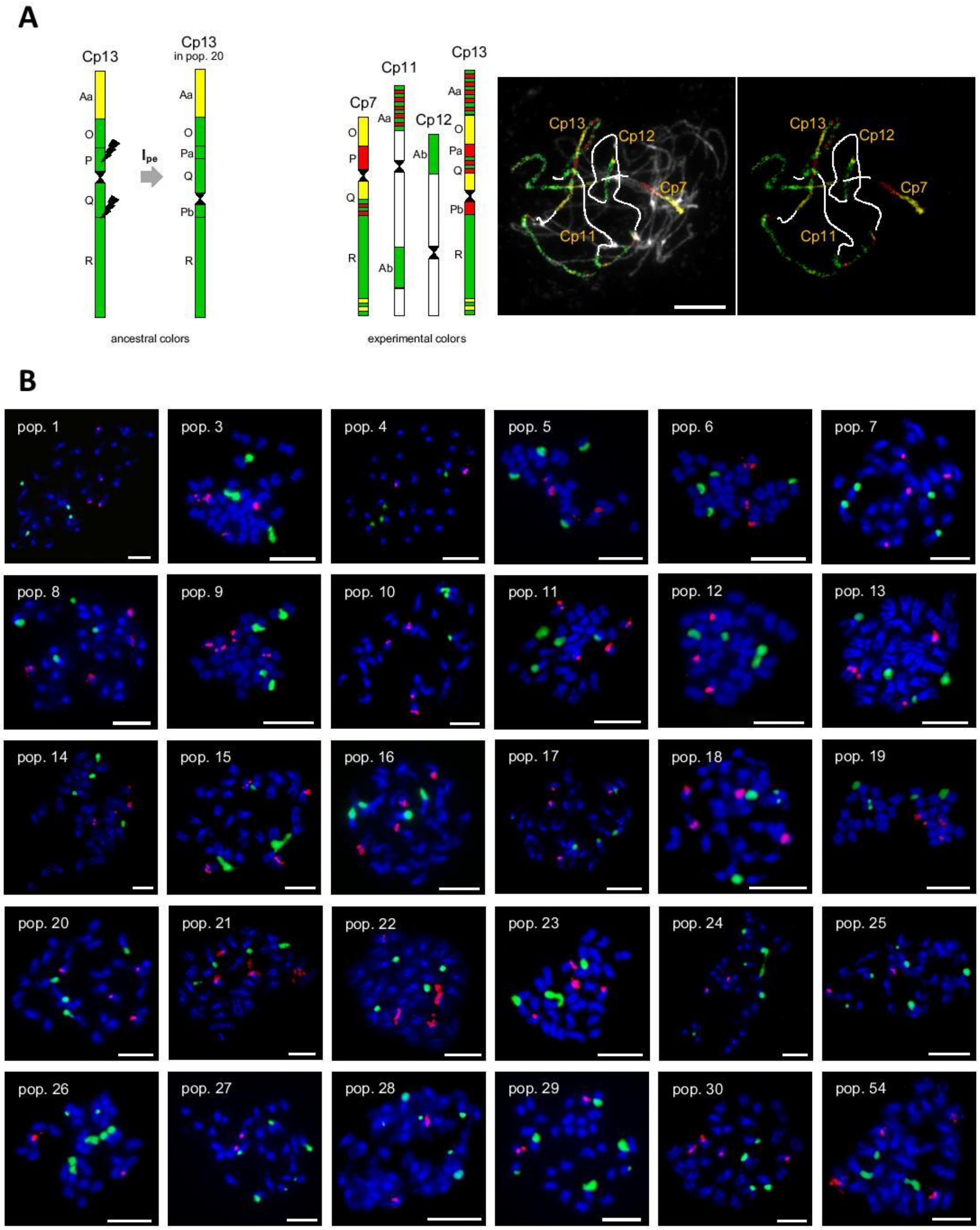

Figure S3. Chromosome analysis of *Catolobus pendulus*. (A) Pericentric inversion on chromosome Cp13 revealed by comparative chromosome painting in population 20. Chromosomes Cp7, Cp11, Cp12, and Cp13 in pachytene were painted using *A. thaliana* BAC contigs representing genomic blocks A, O, P, Q, and R, respectively. Color coding and capital letters correspond to chromosomes and genomic blocks of ACK. Hourglass symbols mark centromeres. I_{pe}: pericentric inversion. (B) Examples of *in situ* localization of 5S (red) and 35S rDNA (green) loci on mitotic chromosomes in thirty populations analyzed. Scale bars, 10 µm.

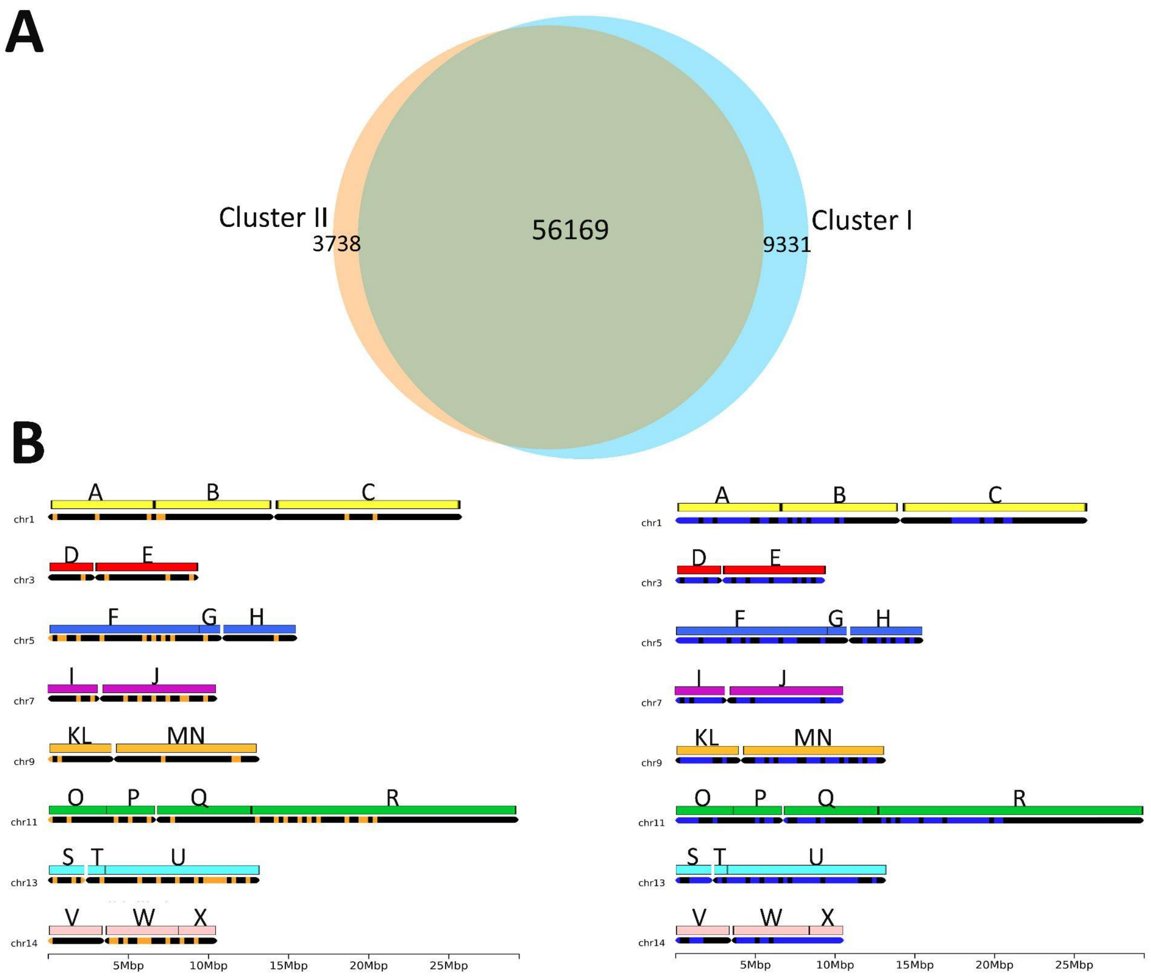

Figure S4. Single nucleotide polymorphism (SNP) between the two identified genetic clusters. (A) Venn diagram of shared and unique SNPs of cluster I and II. (B) ChromoMap-based mapping of genes with specific SNPs from cluster I and II on *Catolobus* chromosomes based on ancestral genomic blocks within the *A. thaliana* genome.

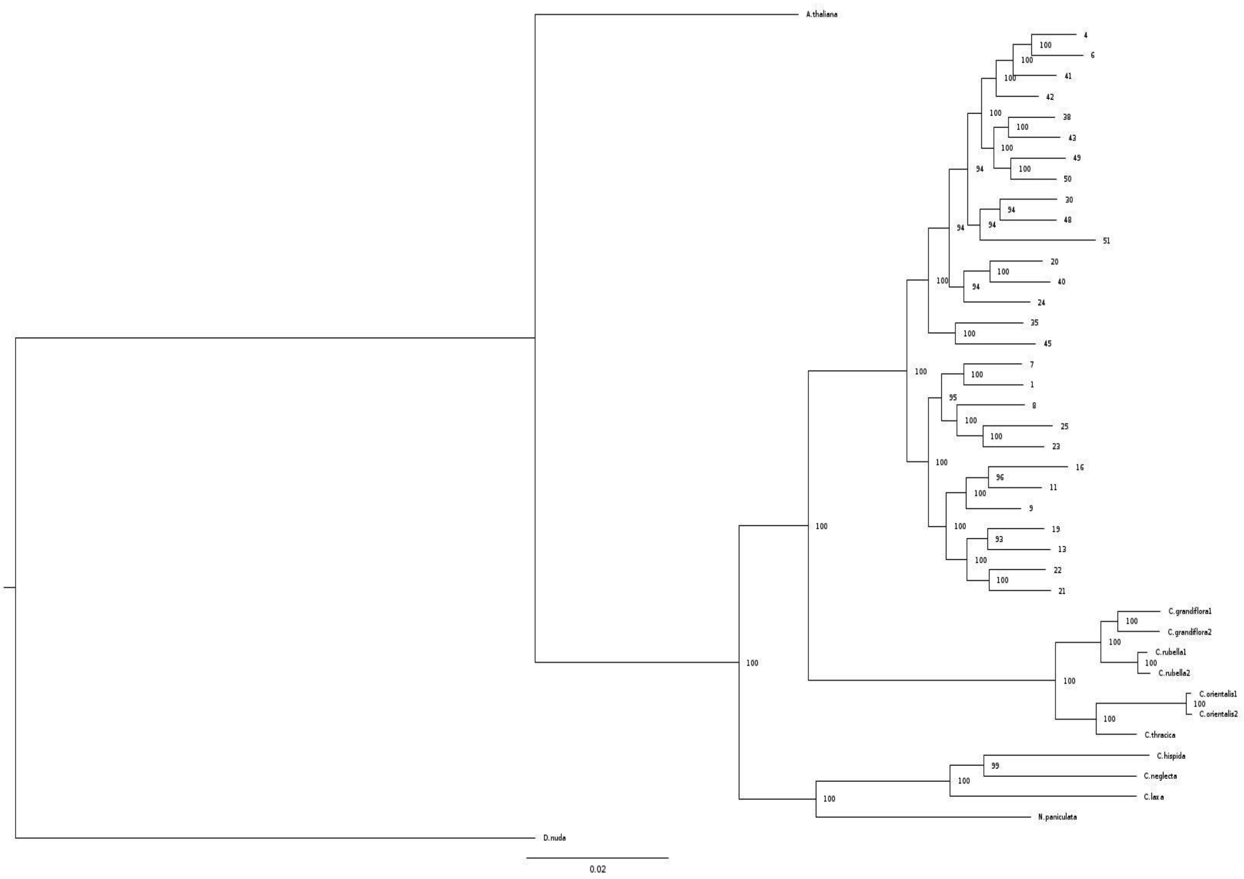

Figure S5. A ML tree of the Camelineae accessions using 1000 fast bootstraps. Numbers at nodes represent branch support. See Table S1 for more information on the accessions studied.

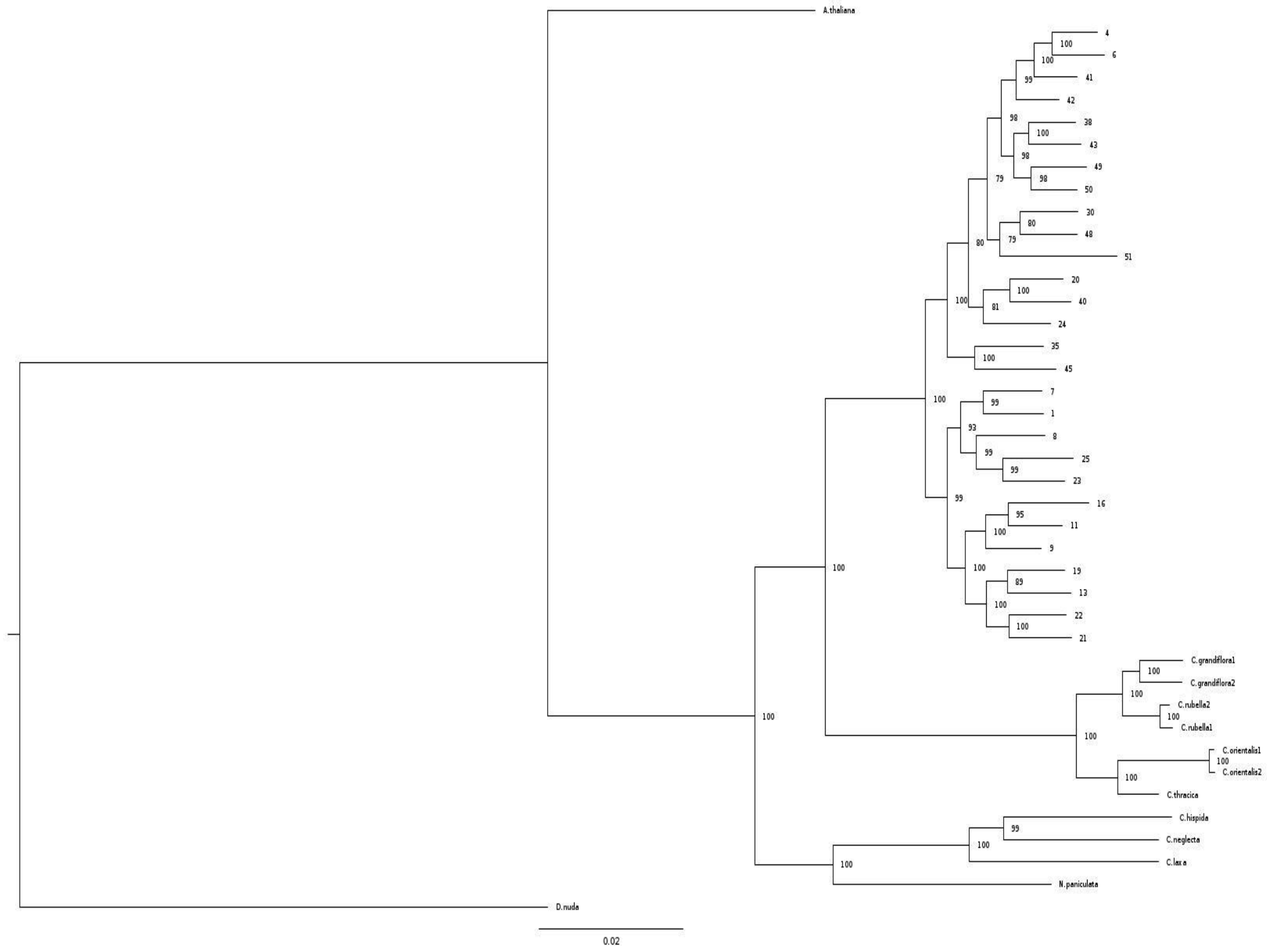

Figure S6. A ML tree of the Camelineae accessions using 200 standard nonparametric bootstraps.

Numbers at nodes represent branch support. See Table S1 for more information on the accessions studied.

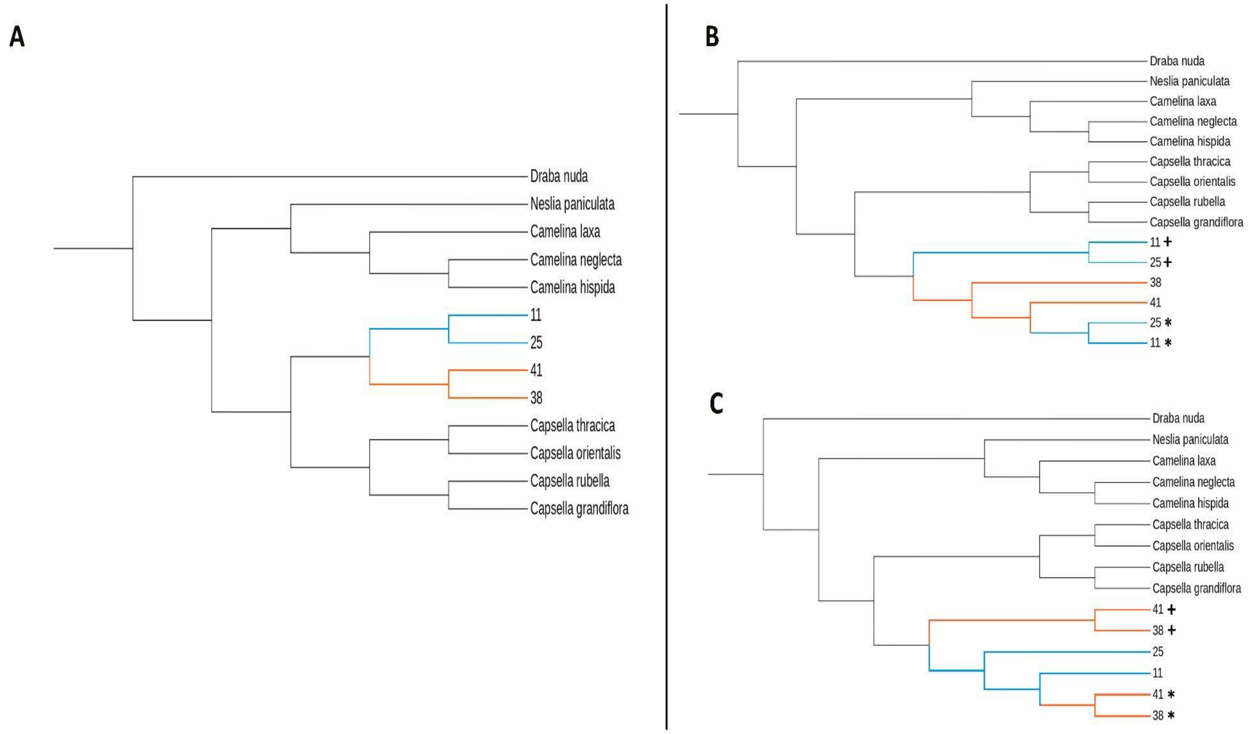

Figure S7. GRAMPA reconciliation in nine Camelineae species to infer the possible mode of polyploidization in *Catolobus*. (A) Simplified species phylogeny of the studied species comprising four *Catolobu*s accessions, two from cluster I (11 and 25, blue branches) and two from cluster II (41 and 38, orange branches). (B) Best GRAMPA multi-labeled tree with the lowest parsimony score. (C) Second best multi-labeled tree from GRAMPA. Tree topologies in (B) and (C) indicate that the hypotetraploid *Catolobus* arose as an autotetraploid.

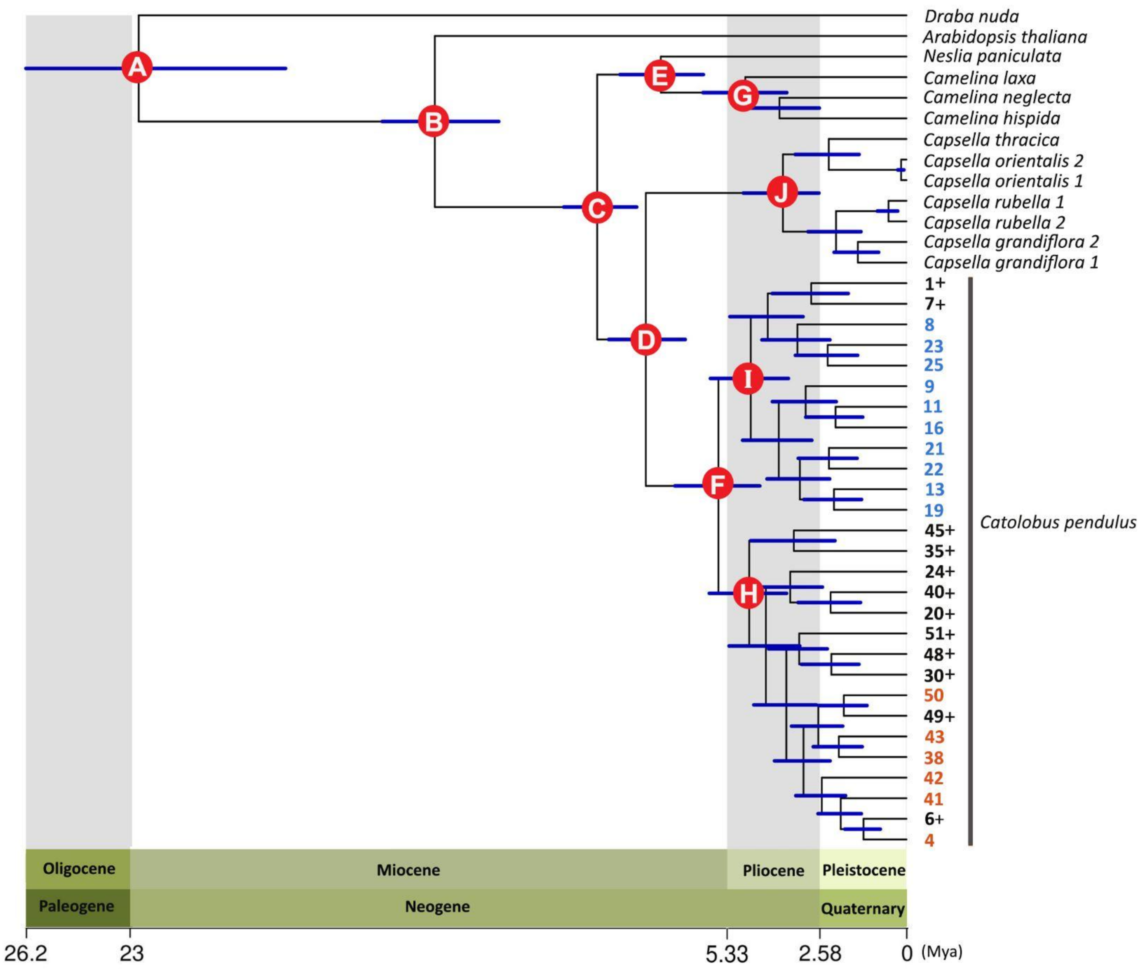

Node	Calibration I (Mya)	Calibration II (Mya)
A	22.83(18.46-26.19)	19.27(15.31-22.21)
B	14.02(12.12-15.59)	11.22(9.28-12.8)
C	9.197(8.02-10.19)	6.64(5.94-7.44)
D	7.75(6.57-8.85)	5.69(4.93-6.48)
E	7.3(6.04-8.52)	5.3(4.42-6.18)
F	5.59(4.36-6.89)	4.31(3.45-5.2)
G	4.79(3.55-6.05)	3.52(2.63-4.43)
H	4.676(3.56-5.86)	3.64(2.84-4.48)
I	4.629(3.51-5.83)	3.6(2.79-4.46)
J	3.67(2.6-4.85)	2.83(2.04-3.72)

Figure S8. Dated ML phylogeny of *Catolobus* and closely related species calibrated based on data in Huang et al. (2016). The letters at the nodes correspond to the letters in the table. The table shows median node ages with 95% confidence intervals using secondary calibrations from Huang et al. (2016; Calibration I) and Hendriks et al. (2023; Calibration II).

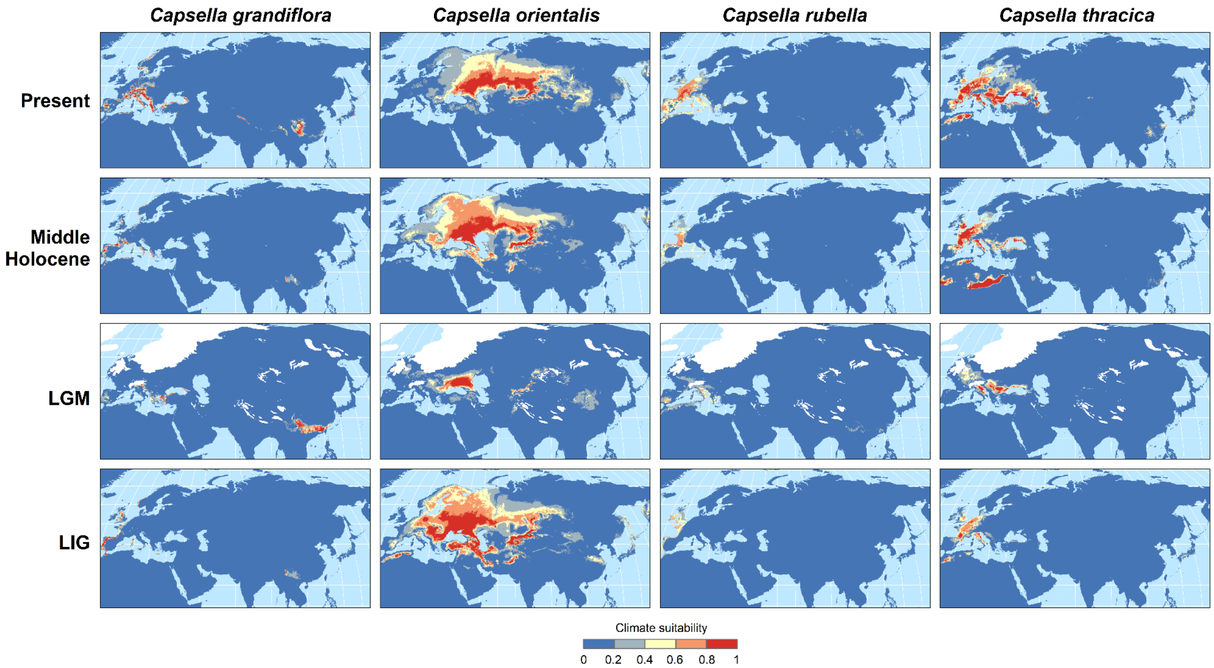

Figure S9. Habitat suitability modeling in four *Capsella* species using MaxEnt at four time periods: present, Middle Holocene, Last Glacial Maximum (LGM), and Last Interglacial (LIG). Habitat suitability is scaled between 0 and 1 and colored in blue and red, respectively. Values closer to 1 (red color) indicate more suitable habitats. Continental ice sheets and mountain glaciers in the LGM are white.

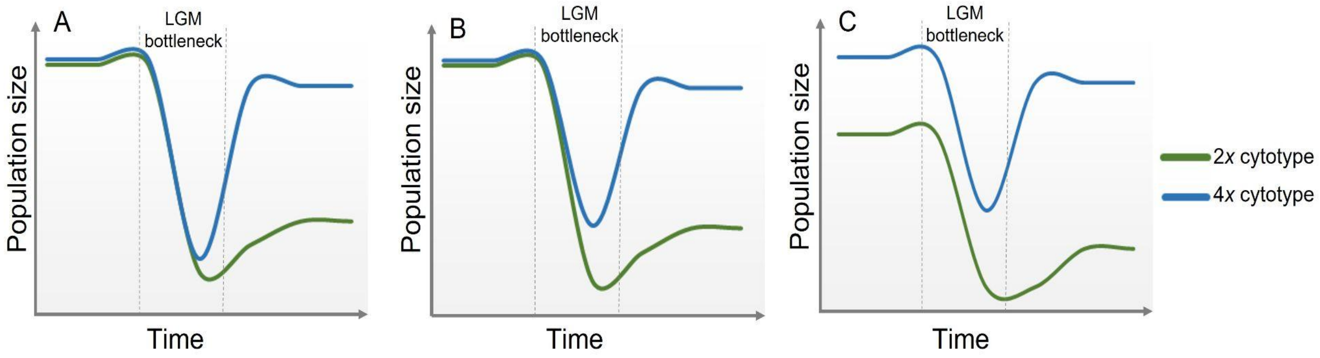

Figure S10. Hypothetical scenarios describing the possible influence of the Last Glaciation Maximum bottleneck on the population size of *Catolobus* 2*x* (green line) and 4*x* (blue line) cytotypes.

1.2 Supplementary Tables

Table S1. Summary data for Camelineae accessions analyzed.

Species	Accession-code	Seed Bank No./ Herbarium sheet number	Locality and collector	Material	Chromosome count	rDNA+telom	CCP	Hyb-Seq analyses	NCBI accession number of Hyb-Seq raw data	BRNU herbarium sheet number
Catolobus pendulus	1	4084	Russia, Altai Кepublic, valley of Talitsa upstream Sanarovka, N 51°04’ E 84°24', collector D. A. German	seeds	yes	yes	no	yes	SAMN32986178	
	2		Russia, Altai Кepublic, Izvestkovyi, N 51°45’ E 85°44', collector D. A. German	seeds	yes	yes	no	no	-	
	3	3895, 4159	Russia, Krasnoyarsk Krai, Sharypovsky District, N 55°12’ E 89°20', collector Barbara Neuffer	seeds	yes	yes	no	no	-	BRNU 681207
	4	3889	Russia, Buryatia, Tunkinsky District, N 51°55’ E 102°30', collector Barbara Neuffer	seeds	yes	yes	yes	yes	SAMN32986179	BRNU 681209
	5	3888	Russia, Irkutsk province, Irkutsk, N 52°18’ E 104°15’, collector Barbara Neuffer	seeds	yes	yes	no	no	-	
	6	3893	Russia, Buryatia, Severobaykal'skiy Rayon, N 55°03’ E 109°06', collector Barbara Neuffer	seeds	yes	yes	yes	yes	SAMN32986180	BRNU 681206
	7	524	China, Xinjiang, East Tian-Shan, N 43°54’ E 88°07', near Tianchi (Heaven Lake)	seeds	yes	yes	yes	yes	SAMN32986181	
	8	3798	China, Sichuan, N 30°17.1’ E 101°31.8’, collector Jan Stepanek	seeds	yes	yes	yes	yes	SAMN32986182	BRNU 681205
	9	3886	Mongolia, Erdene, approximately N 44°26' E 111°05’, collector Barbara Neuffer	seeds	yes	yes	no	yes	SAMN32986183	BRNU 681208
	10	3894, 4158	Mongolia, Numrug, N 46°56’ E 119°31', collector Barbara Neuffer	seeds	yes	yes	no	no	-	
	11	4091	Russia, Primorski Krai, Putsilovka, N 43°50’ E 131°37', collector E. V. Boltenkov	seeds	yes	yes	no	yes	SAMN32986184	
	12	4093	Russia, Primorski Krai, Vladivostok, collector E. V. Boltenkov	seeds	yes	yes	no	no	-	
	13	4092	Russia, Primorski Krai, Vladivostok, Cherkavskogo Peninsular, near the house at Gulbinovicha str., N 43°06’ E 131°53', collector E. V. Boltenkov	seeds	yes	yes	no	yes	SAMN32986185	
	14	4090	Russia, Primorski Krai, Vladivostok, Reinecke Island, N 42°55’ E 131°44', collector M. N. Koldaeva	seeds	yes	yes	no	no	-	
	15	4089, 4855	Russia, Khabarovsk Krai, near De-Kastri, N 51°28’ E 140°47', collector M. N. Koldaeva	seeds	yes	yes	no	no	-	
	16	1195	Russia, Magadan, Nagayev bay, N 59°33’ E 150°47', collector A. N. Berkutenko	seeds	yes	yes	no	yes	SAMN32986186	
	17	1196, 1746, 1961, 2961, 4484	Russia, Magadan, Portovaya, collector A. N. Berkutenko	seeds	yes	yes	no	no	-	BRNU 681203 (Flowers), BRNU 681202 (Fruits)
	18	4085, 4851, 4982	Russia, Magadan province, Magadan city, Portovaya str. 18, collector E. A. Andriyanova	seeds	yes	yes	no	no	-	BRNU 681210
	19	4086	Russia, Magadan province, Magadan city, Marchekansy str. 7a, collector E. A. Andriyanova	seeds	yes	yes	no	yes	SAMN32986187	BRNU 681211
	20	1191	Russia, Magadan, collector N. A. Sazonova	seeds	yes	yes	yes	yes	SAMN32986188	
	21	4088	Russia, Magadan province, former settlemet Atargan (vicinities of Ola), Roadside at sandy pebbly locality near the sea, N 59°34’ E 151°26', collector E. A. Andriyanova	seeds	yes	yes	no	yes	SAMN32986189	BRNU 681204
	22	4087	Russia, Magadan province, Seimchan, N 62°53’ E 152°26', collector E. A. Andriyanova	seeds	yes	yes	no	yes	SAMN32986190	BRNU 681212
	23	4006	Japan, Akan-cho, Kushiro-shi, Hokkaido, 43°25´54.8´´N 144°08´12.0´´E, Alt.440 m, collector Hiroshi Kudoh	seeds	yes	yes	no	yes	SAMN32986191	
	24	4000	Japan, Harutori-ko, Syunkodai, Kushiro-ski, Hokkaido, 42° 58´28.0´´N 144°24´19.2E, Alt. 2 m, collector Hiroshi Kudoh	seeds	yes	yes	yes	yes	SAMN32986192	
	25	4001	Japan, Harutori-ko, Syunkodai, Kushiro-ski, Hokkaido, 42° 58´28.0´´N 144°24´19.2E, Alt. 2 m, collector Hiroshi Kudoh	seeds	yes	yes	no	yes	SAMN32986193	
	26	4002, 4847	Japan, Harutori-ko, Syunkodai, Kushiro-ski, Hokkaido, 42° 58´28.0´´N 144°24´19.2E, Alt. 2 m, collector Hiroshi Kudoh	seeds	yes	yes	no	no	-	
	27	4003, 4848	Japan, Harutori-ko, Syunkodai, Kushiro-ski, Hokkaido, 42° 58´28.0´´N 144°24´19.2E, Alt. 2 m, collector Hiroshi Kudoh	seeds	yes	yes	no	no	-	
	28	4004, 4849	Japan, Harutori-ko, Syunkodai, Kushiro-ski, Hokkaido, 42° 58´28.0´´N 144°24´19.2E, Alt. 2 m, collector Hiroshi Kudoh	seeds	yes	yes	no	no	-	
	29	4005	Japan, Harutori-ko, Syunkodai, Kushiro-ski, Hokkaido, 42° 58´28.0´´N 144°24´19.2E, Alt. 2 m, collector Hiroshi Kudoh	seeds	yes	yes	no	no	-	

	30	4482	Russia, Voronezh province, Ramonsky district, deciduous forest near floodplain of Voronezh river; N 51.882024, E 39.32139, collector Aleksey Baushev	seeds	yes	yes	yes	yes	SAMN32986194	
	35	-	Russia, Vologda province, Velikoustyugskiy district, ca. N 60°41’ E 46°18', collectors Korchagin and Zubkov	herbarium-leaves	no	no	no	yes	SAMN32986195	
	38	223a	Russia, Bashrortostan, Duvansky district, north of Meteli, 56.049904, 57.926287, collectors Ya. Ya. Vasiliev and T. P. Lind	herbarium-leaves	no	no	no	yes	SAMN32986196	
	40	672	Russia, Leningrad province, Bolshaya Izhora, N 59°56’ E 29°36', collector N. N. Tzvelev	herbarium-leaves	no	no	no	yes	SAMN32986197	
	41	-	Russia, Kostroma province, near Ileykino, 58°58'47.0"N 42°11'14.0"E, collectors G. Yu. Konechnaya et al.	herbarium-leaves	no	no	no	yes	SAMN32986198	
	42	7284-2	Russia, Ryazan province, between Dankovo and Mimishkino, N 55°03’ E 41°17', collectors V. Tikhomirov and N. Oktiabriova	herbarium-leaves	no	no	no	yes	SAMN32986199	
	43	18	Russia, Tatarstan, Kazan, N 55°48’ E 49°11', collector Yu. Gusev	herbarium-leaves	no	no	no	yes	SAMN32986200	
	45	-	Russia, Penza province, 3-4 verst west of Yelyuzan station, N 53°09’ E 46°07', collector E. C. Stuckenberg	herbarium-leaves	no	no	no	yes	SAMN32986201	
	48	-	Ukraine, Charkov province, near Zmiev, N 49°41’ E 36°20', collector N. N. Tzvelev	herbarium-leaves	no	no	no	yes	SAMN32986202	
	49	948	Russia, Rostov province, near the khutor Fomin, N 46°57’ E 43°39', collector A. Voronov	herbarium-leaves	no	no	no	yes	SAMN32986203	
	50	-	Russia, Rostov province, 14 km east of Kazanskaya, near Solontsovsky, N 49°48’ E 41°19', collector A. N. Shmaraeva	herbarium-leaves	no	no	no	yes	SAMN32986204	
	51	171	Russia, Volgograd province, 2-3 km south of Slaschevskaya, N 49°50’ E 42°20', collectors V. D. Bochkin and M. Yu. Polonskaya	herbarium-leaves	no	no	no	yes	SAMN32986205	
	53	4811	Russia, Petropavlovsk-Kamchatsky, N 53°01’ E 158°39', colelctor Olga Chernyagina	seeds	yes	yes	no	no	-	
	54	4969	Russia, Voronezh, Bulvar Pobedy 8, 186, N 51°43’ E 39°10', collector Aleksei Baushev	seeds	yes	yes	no	no	-	
	C.grandiflora1	3902	Italy, approximately N 45°28' E 10°04’, collector Barbara Neuffer	Seeds	no	no	no	yes	SAMN32986206	
Capsella grandiflora	C.grandiflora2	3903	Greece, approximately N 39°29' E 19°53’, collector Barbara Neuffer	Seeds	no	no	no	yes	SAMN32986207	
	C.orientalis1	3905	Mongolia, approximately N 48°05' E 106°50’, collector Barbara Neuffer	Seeds	no	no	no	yes	SAMN32986208	
Capsella orientalis	C.orientalis2	3906	Kazakhstan, approximately N 49°51' E 82°25’, collector Barbara Neuffer	Seeds	no	no	no	yes	SAMN32986209	
	C.rubella1	3908	Portugal, approximately N 41°03' W 8°04’, collector Barbara Neuffer	Seeds	no	no	no	yes	SAMN32986210	
Capsella rubella	C.rubella2	3910	Italy, approximately N 43°46' E 11°14’, collector Barbara Neuffer	Seeds	no	no	no	yes	SAMN32986211	
Capsella thracica	C.thracica	3912	Bulgaria, approximately N 42°06' E 27°56’, collector Barbara Neuffer	Seeds	no	no	no	yes	SAMN32986212	
Camelina hispida	C.hispida	2760	Turkey, Ankara, collector Jordan Brock	Seeds	no	no	no	yes	SAMN32986213	
Camelina laxa	C.laxa	2765	Georgia, Samtskhe-Javakheti, collector Jordan Brock	Seeds	no	no	no	yes	SAMN32986214	
Camelina neglecta	C.neglecta	3270	France, Lozère, USDA/ PI 650135/ MO6869197/	Seeds	no	no	no	yes	SAMN32986215	
Neslia paniculata	N.paniculata	3123	08/2002, Lysák, code-Nr.28	Seeds	no	no	no	yes	SAMN32986216	

Table S2. List of merged unique loci from Angiosperm-353 and Brassicaceae-specific baits used in Hyb-Seq data analyses.

Angio-353 and Brassicaceae-specific baits merged loci					
Arath-4471	Athaliana-AT1G06950.1@14	Athaliana-AT2G26780.1@24	Athaliana-AT3G27870.1@2	Athaliana-AT5G05680.1@6	Athaliana-AT5G53920.1@6
Arath-4527	Athaliana-AT1G07010.1@1	Athaliana-AT2G26780.1@26	Athaliana-AT3G27870.1@4	Athaliana-AT5G05680.1@7	Athaliana-AT5G53970.1@3
Arath-4691	Athaliana-AT1G07590.1@3	Athaliana-AT2G26780.1@27	Athaliana-AT3G27870.1@11	Athaliana-AT5G05840.1@2	Athaliana-AT5G54080.2@2
Arath-4724	Athaliana-AT1G08460.1@2	Athaliana-AT2G26780.1@31	Athaliana-AT3G28040.1@1	Athaliana-AT5G05840.1@3	Athaliana-AT5G54080.2@9
Arath-4744	Athaliana-AT1G09010.1@6	Athaliana-AT2G26780.1@35	Athaliana-AT3G28040.1@2	Athaliana-AT5G06120.3@2	Athaliana-AT5G54180.1@2
Ambtr-4757	Athaliana-AT1G09010.1@8	Athaliana-AT2G26800.1@9	Athaliana-AT3G29320.1@6	Athaliana-AT5G06120.4@24	Athaliana-AT5G54260.1@20
AEPI-4793	Athaliana-AT1G09010.1@9	Athaliana-AT2G26800.3@4	Athaliana-AT3G29320.1@7	Athaliana-AT5G06940.1@1	Athaliana-AT5G54570.1@6
Arath-4796	Athaliana-AT1G09010.1@10	Athaliana-AT2G26930.1@6	Athaliana-AT3G29320.1@8	Athaliana-AT5G06970.1@10	Athaliana-AT5G54570.1@8
Arath-4802	Athaliana-AT1G09010.1@11	Athaliana-AT2G27090.1@2	Athaliana-AT3G29320.1@11	Athaliana-AT5G06970.1@12	Athaliana-AT5G54690.1@3
Arath-4806	Athaliana-AT1G09340.1@7	Athaliana-AT2G27090.1@4	Athaliana-AT3G43240.1@2	Athaliana-AT5G06970.1@15	Athaliana-AT5G54690.1@5
Arath-4848	Athaliana-AT1G09870.1@3	Athaliana-AT2G27090.1@6	Athaliana-AT3G43240.1@10	Athaliana-AT5G06970.1@27	Athaliana-AT5G54830.1@2
Ambtr-4889	Athaliana-AT1G09870.1@9	Athaliana-AT2G27500.3@1	Athaliana-AT3G43240.1@11	Athaliana-AT5G07590.1@2	Athaliana-AT5G55250.2@2
Arath-4890	Athaliana-AT1G09900.1@1	Athaliana-AT2G27590.1@3	Athaliana-AT3G43300.1@3	Athaliana-AT5G07590.1@3	Athaliana-AT5G55540.1@1
Arath-4893	Athaliana-AT1G10020.1@1	Athaliana-AT2G28070.1@1	Athaliana-AT3G43300.1@21	Athaliana-AT5G07590.1@6	Athaliana-AT5G55540.1@2
Arath-4932	Athaliana-AT1G10240.1@2	Athaliana-AT2G28070.1@3	Athaliana-AT3G43300.1@22	Athaliana-AT5G08030.1@5	Athaliana-AT5G55960.1@1
Arath-4942	Athaliana-AT1G10240.1@3	Athaliana-AT2G28070.1@7	Athaliana-AT3G43300.1@25	Athaliana-AT5G08280.1@2	Athaliana-AT5G56220.1@1
Arath-4951	Athaliana-AT1G10330.1@1	Athaliana-AT2G28070.1@9	Athaliana-AT3G43300.1@31	Athaliana-AT5G08280.1@3	Athaliana-AT5G56290.1@4
Arath-4954	Athaliana-AT1G10385.1@3	Athaliana-AT2G28780.1@3	Athaliana-AT3G43300.2@8	Athaliana-AT5G08310.1@1	Athaliana-AT5G56290.1@5
Arath-4989	Athaliana-AT1G10780.1@3	Athaliana-AT2G29050.1@1	Athaliana-AT3G43300.2@17	Athaliana-AT5G08415.1@1	Athaliana-AT5G56290.1@8
Arath-4992	Athaliana-AT1G10910.1@3	Athaliana-AT2G29050.1@4	Athaliana-AT3G43300.2@19	Athaliana-AT5G08490.1@1	Athaliana-AT5G56290.1@9
Arath-5018	Athaliana-AT1G10910.1@4	Athaliana-AT2G29560.1@12	Athaliana-AT3G43300.2@20	Athaliana-AT5G08530.1@2	Athaliana-AT5G56290.1@12
Arath-5032	Athaliana-AT1G10910.1@7	Athaliana-AT2G30520.1@3	Athaliana-AT3G44330.1@1	Athaliana-AT5G08580.1@1	Athaliana-AT5G56580.1@4
Arath-5034	Athaliana-AT1G10910.1@10	Athaliana-AT2G30520.1@4	Athaliana-AT3G44880.1@1	Athaliana-AT5G08580.1@2	Athaliana-AT5G56730.1@21
Arath-5038	Athaliana-AT1G11000.1@3	Athaliana-AT2G31530.1@2	Athaliana-AT3G44880.1@2	Athaliana-AT5G08660.1@13	Athaliana-AT5G57800.1@4
Arath-5064	Athaliana-AT1G11915.1@1	Athaliana-AT2G31740.1@1	Athaliana-AT3G44880.1@6	Athaliana-AT5G10900.1@4	Athaliana-AT5G58020.1@1
Arath-5090	Athaliana-AT1G12150.1@3	Athaliana-AT2G31740.1@5	Athaliana-AT3G44880.1@7	Athaliana-AT5G10900.1@5	Athaliana-AT5G58230.1@1
ABSS-5104	Athaliana-AT1G12330.1@3	Athaliana-AT2G31970.1@6	Athaliana-AT3G46220.1@3	Athaliana-AT5G11040.1@1	Athaliana-AT5G58230.1@2
Arath-5116	Athaliana-AT1G12470.1@3	Athaliana-AT2G31970.1@11	Athaliana-AT3G46220.3@2	Athaliana-AT5G11040.1@2	Athaliana-AT5G58230.1@6
Arath-5123	Athaliana-AT1G12470.1@4	Athaliana-AT2G31970.1@17	Athaliana-AT3G47400.1@1	Athaliana-AT5G11040.1@3	Athaliana-AT5G58330.3@4
Arath-5131	Athaliana-AT1G12470.1@19	Athaliana-AT2G31970.1@18	Athaliana-AT3G47400.1@2	Athaliana-AT5G11040.1@6	Athaliana-AT5G58410.1@7
Arath-5138	Athaliana-AT1G12470.1@20	Athaliana-AT2G31970.1@20	Athaliana-AT3G47700.1@4	Athaliana-AT5G11040.1@7	Athaliana-AT5G58410.1@8
Arath-5162	Athaliana-AT1G12470.1@24	Athaliana-AT2G32040.1@2	Athaliana-AT3G48110.1@24	Athaliana-AT5G11040.1@8	Athaliana-AT5G58410.1@11
Arath-5163	Athaliana-AT1G13970.1@8	Athaliana-AT2G32040.1@6	Athaliana-AT3G48150.1@3	Athaliana-AT5G11040.1@9	Athaliana-AT5G58410.1@12
Arath-5168	Athaliana-AT1G14300.2@3	Athaliana-AT2G32040.1@9	Athaliana-AT3G48380.1@3	Athaliana-AT5G11040.1@10	Athaliana-AT5G58410.1@13
Arath-5177	Athaliana-AT1G15440.1@1	Athaliana-AT2G32290.1@2	Athaliana-AT3G48380.2@2	Athaliana-AT5G11330.1@4	Athaliana-AT5G58410.1@14
Arath-5188	Athaliana-AT1G15440.2@3	Athaliana-AT2G32590.1@6	Athaliana-AT3G48380.2@13	Athaliana-AT5G11330.2@1	Athaliana-AT5G58430.1@1
Arath-5200	Athaliana-AT1G15440.2@5	Athaliana-AT2G32590.1@9	Athaliana-AT3G48425.1@1	Athaliana-AT5G11330.2@3	Athaliana-AT5G58470.1@1
Arath-5206	Athaliana-AT1G15440.2@6	Athaliana-AT2G32900.1@1	Athaliana-AT3G48425.1@4	Athaliana-AT5G11380.1@5	Athaliana-AT5G58470.1@3

Arath-5220	Athaliana-AT1G15740.1@2	Athaliana-AT2G32900.1@5	Athaliana-AT3G48500.1@12	Athaliana-AT5G11380.1@8	Athaliana-AT5G58480.1@1
Arath-5257	Athaliana-AT1G15740.1@4	Athaliana-AT2G33770.1@3	Athaliana-AT3G48610.1@1	Athaliana-AT5G11380.1@9	Athaliana-AT5G58480.1@2
ARYD-5260	Athaliana-AT1G15740.1@16	Athaliana-AT2G33770.1@5	Athaliana-AT3G48610.1@2	Athaliana-AT5G11380.2@6	Athaliana-AT5G58510.1@8
Arath-5264	Athaliana-AT1G15980.1@1	Athaliana-AT2G33770.1@8	Athaliana-AT3G48820.2@6	Athaliana-AT5G11380.2@7	Athaliana-AT5G58510.1@19
Arath-5271	Athaliana-AT1G15980.1@3	Athaliana-AT2G34640.1@3	Athaliana-AT3G49400.1@15	Athaliana-AT5G11390.1@3	Athaliana-AT5G58510.1@22
Arath-5273	Athaliana-AT1G15980.1@4	Athaliana-AT2G34640.1@5	Athaliana-AT3G49400.1@16	Athaliana-AT5G11560.1@6	Athaliana-AT5G58600.1@1
Arath-5280	Athaliana-AT1G16070.1@1	Athaliana-AT2G34960.1@1	Athaliana-AT3G49400.2@8	Athaliana-AT5G11560.1@7	Athaliana-AT5G58750.1@1
Ambtr-5296	Athaliana-AT1G16570.1@15	Athaliana-AT2G35040.1@9	Athaliana-AT3G50620.1@4	Athaliana-AT5G11560.1@10	Athaliana-AT5G58940.1@2
Arath-5299	Athaliana-AT1G16720.1@1	Athaliana-AT2G35040.2@10	Athaliana-AT3G50660.1@8	Athaliana-AT5G11960.1@8	Athaliana-AT5G58940.1@5
Arath-5304	Athaliana-AT1G16720.1@3	Athaliana-AT2G35150.1@1	Athaliana-AT3G51470.1@3	Athaliana-AT5G11960.1@9	Athaliana-AT5G59900.1@1
AUIP-5318	Athaliana-AT1G16770.1@2	Athaliana-AT2G35500.1@3	Athaliana-AT3G51490.2@5	Athaliana-AT5G12040.1@3	Athaliana-AT5G60020.1@3
Ambtr-5326	Athaliana-AT1G17760.1@21	Athaliana-AT2G35610.1@2	Athaliana-AT3G51930.1@1	Athaliana-AT5G12290.1@3	Athaliana-AT5G60020.1@5
Arath-5328	Athaliana-AT1G18270.2@12	Athaliana-AT2G35610.1@3	Athaliana-AT3G52200.1@9	Athaliana-AT5G12290.1@6	Athaliana-AT5G60450.1@1
Arath-5333	Athaliana-AT1G18270.2@23	Athaliana-AT2G35610.1@5	Athaliana-AT3G52200.1@13	Athaliana-AT5G12470.1@1	Athaliana-AT5G60570.1@2
Arath-5335	Athaliana-AT1G18270.2@42	Athaliana-AT2G35720.1@2	Athaliana-AT3G52940.2@2	Athaliana-AT5G12900.1@2	Athaliana-AT5G60600.2@2
Arath-5339	Athaliana-AT1G18270.3@39	Athaliana-AT2G35720.1@3	Athaliana-AT3G52970.1@3	Athaliana-AT5G13020.1@5	Athaliana-AT5G60600.3@8
Arath-5343	Athaliana-AT1G19025.1@3	Athaliana-AT2G35920.1@12	Athaliana-AT3G53100.1@4	Athaliana-AT5G13020.1@6	Athaliana-AT5G60750.1@4
Arath-5347	Athaliana-AT1G19025.1@4	Athaliana-AT2G35920.1@13	Athaliana-AT3G53150.1@1	Athaliana-AT5G13020.1@8	Athaliana-AT5G60790.1@4
Arath-5348	Athaliana-AT1G19860.1@2	Athaliana-AT2G35920.1@14	Athaliana-AT3G53180.1@2	Athaliana-AT5G13030.1@4	Athaliana-AT5G60980.1@3
Arath-5354	Athaliana-AT1G19860.1@4	Athaliana-AT2G35920.1@15	Athaliana-AT3G53180.1@14	Athaliana-AT5G13030.1@5	Athaliana-AT5G60980.2@4
Arath-5355	Athaliana-AT1G20080.1@10	Athaliana-AT2G36840.1@4	Athaliana-AT3G53180.1@15	Athaliana-AT5G13030.1@6	Athaliana-AT5G61400.1@1
Arath-5357	Athaliana-AT1G20560.1@2	Athaliana-AT2G36840.1@5	Athaliana-AT3G54690.1@1	Athaliana-AT5G13030.1@7	Athaliana-AT5G61560.1@9
Arath-5366	Athaliana-AT1G21640.2@2	Athaliana-AT2G37230.1@1	Athaliana-AT3G54690.1@2	Athaliana-AT5G13030.1@8	Athaliana-AT5G61560.1@10
Arath-5398	Athaliana-AT1G21640.2@5	Athaliana-AT2G37310.1@1	Athaliana-AT3G54790.1@2	Athaliana-AT5G13230.1@1	Athaliana-AT5G61865.1@4
Arath-5404	Athaliana-AT1G21640.2@8	Athaliana-AT2G37320.1@1	Athaliana-AT3G54790.1@4	Athaliana-AT5G13420.1@4	Athaliana-AT5G62130.1@2
Arath-5406	Athaliana-AT1G22620.1@2	Athaliana-AT2G37370.1@18	Athaliana-AT3G54860.2@4	Athaliana-AT5G13420.1@5	Athaliana-AT5G62130.1@3
Arath-5421	Athaliana-AT1G22620.1@4	Athaliana-AT2G38000.1@4	Athaliana-AT3G55070.1@6	Athaliana-AT5G13420.1@6	Athaliana-AT5G62310.1@4
Arath-5422	Athaliana-AT1G22620.1@6	Athaliana-AT2G38000.1@7	Athaliana-AT3G55070.2@2	Athaliana-AT5G13420.1@7	Athaliana-AT5G62310.1@6
Arath-5426	Athaliana-AT1G22620.1@10	Athaliana-AT2G38280.2@7	Athaliana-AT3G55070.2@4	Athaliana-AT5G13530.1@2	Athaliana-AT5G62310.1@10
Arath-5427	Athaliana-AT1G22620.1@13	Athaliana-AT2G38500.1@1	Athaliana-AT3G55160.1@1	Athaliana-AT5G13530.1@3	Athaliana-AT5G62370.1@1
Arath-5428	Athaliana-AT1G22620.1@16	Athaliana-AT2G38500.1@2	Athaliana-AT3G55160.1@2	Athaliana-AT5G13530.1@5	Athaliana-AT5G63010.1@1
Arath-5430	Athaliana-AT1G22770.1@5	Athaliana-AT2G38510.1@1	Athaliana-AT3G55160.1@3	Athaliana-AT5G13530.1@12	Athaliana-AT5G63010.1@2
Arath-5434	Athaliana-AT1G22770.1@7	Athaliana-AT2G38770.1@1	Athaliana-AT3G55160.1@4	Athaliana-AT5G13530.1@13	Athaliana-AT5G63050.1@4
Arath-5449	Athaliana-AT1G22770.1@9	Athaliana-AT2G38770.1@7	Athaliana-AT3G55580.1@6	Athaliana-AT5G13530.1@16	Athaliana-AT5G63050.1@5
Arath-5454	Athaliana-AT1G22770.1@10	Athaliana-AT2G38770.1@9	Athaliana-AT3G55580.1@8	Athaliana-AT5G13530.2@1	Athaliana-AT5G63410.1@2
Arath-5460	Athaliana-AT1G22770.1@12	Athaliana-AT2G38770.1@10	Athaliana-AT3G56120.1@6	Athaliana-AT5G13530.2@14	Athaliana-AT5G63410.1@4
AFQQ-5463	Athaliana-AT1G24610.1@3	Athaliana-AT2G38770.1@11	Athaliana-AT3G56310.1@1	Athaliana-AT5G13530.2@15	Athaliana-AT5G63410.1@7
Ambtr-5464	Athaliana-AT1G24610.1@4	Athaliana-AT2G38770.1@12	Athaliana-AT3G56370.1@2	Athaliana-AT5G13560.1@2	Athaliana-AT5G63420.1@2
Arath-5477	Athaliana-AT1G24706.1@13	Athaliana-AT2G38770.1@13	Athaliana-AT3G56370.1@3	Athaliana-AT5G13560.1@18	Athaliana-AT5G63420.1@5
Arath-5489	Athaliana-AT1G24706.1@15	Athaliana-AT2G39260.1@3	Athaliana-AT3G56940.1@1	Athaliana-AT5G13630.1@1	Athaliana-AT5G63420.1@7
Arath-5502	Athaliana-AT1G24706.1@16	Athaliana-AT2G39260.1@4	Athaliana-AT3G56940.2@4	Athaliana-AT5G13630.1@3	Athaliana-AT5G63640.1@11

Arath-5513	Athaliana-AT1G24706.2@24	Athaliana-AT2G39260.1@10	Athaliana-AT3G57180.1@2	Athaliana-AT5G13640.1@1	Athaliana-AT5G63770.1@2
Arath-5528	Athaliana-AT1G24706.2@26	Athaliana-AT2G39260.1@12	Athaliana-AT3G57300.1@3	Athaliana-AT5G13640.1@2	Athaliana-AT5G63840.1@1
Arath-5531	Athaliana-AT1G24706.2@32	Athaliana-AT2G39260.1@13	Athaliana-AT3G57300.1@4	Athaliana-AT5G13640.1@3	Athaliana-AT5G63840.1@2
Arath-5536	Athaliana-AT1G25500.1@2	Athaliana-AT2G39260.1@15	Athaliana-AT3G57300.1@5	Athaliana-AT5G13640.1@4	Athaliana-AT5G63840.1@4
Arath-5551	Athaliana-AT1G25500.3@7	Athaliana-AT2G39260.1@16	Athaliana-AT3G57300.1@7	Athaliana-AT5G13640.1@5	Athaliana-AT5G63840.1@5
Arath-5554	Athaliana-AT1G25570.1@2	Athaliana-AT2G39260.1@17	Athaliana-AT3G57300.1@14	Athaliana-AT5G13640.1@6	Athaliana-AT5G63860.1@3
Arath-5562	Athaliana-AT1G25570.1@8	Athaliana-AT2G39450.1@3	Athaliana-AT3G57300.1@16	Athaliana-AT5G13680.1@2	Athaliana-AT5G63920.1@1
Arath-5578	Athaliana-AT1G27460.1@4	Athaliana-AT2G39620.1@1	Athaliana-AT3G57300.2@18	Athaliana-AT5G13690.1@3	Athaliana-AT5G63920.1@9
Arath-5594	Athaliana-AT1G27460.1@6	Athaliana-AT2G39730.1@5	Athaliana-AT3G57300.2@22	Athaliana-AT5G13690.1@16	Athaliana-AT5G63920.1@10
Arath-5596	Athaliana-AT1G27752.1@8	Athaliana-AT2G39730.1@6	Athaliana-AT3G57610.1@1	Athaliana-AT5G13690.1@17	Athaliana-AT5G64270.1@2
Arath-5599	Athaliana-AT1G27752.2@9	Athaliana-AT2G39830.1@8	Athaliana-AT3G57610.1@2	Athaliana-AT5G13690.1@19	Athaliana-AT5G64370.1@3
Arath-5614	Athaliana-AT1G27760.3@8	Athaliana-AT2G39830.1@12	Athaliana-AT3G57610.1@3	Athaliana-AT5G13770.1@1	Athaliana-AT5G64370.1@4
Arath-5620	Athaliana-AT1G29690.1@1	Athaliana-AT2G39970.1@11	Athaliana-AT3G58520.1@1	Athaliana-AT5G14210.1@1	Athaliana-AT5G64370.1@5
Arath-5634	Athaliana-AT1G29690.1@2	Athaliana-AT2G40070.1@1	Athaliana-AT3G58520.2@3	Athaliana-AT5G14210.1@4	Athaliana-AT5G65500.1@4
Arath-5639	Athaliana-AT1G29690.1@3	Athaliana-AT2G40070.1@4	Athaliana-AT3G60830.1@1	Athaliana-AT5G14230.1@2	Athaliana-AT5G65500.1@6
Arath-5642	Athaliana-AT1G29690.1@6	Athaliana-AT2G40070.2@6	Athaliana-AT3G60830.1@3	Athaliana-AT5G14230.1@4	Athaliana-AT5G65950.1@1
Arath-5644	Athaliana-AT1G29900.1@1	Athaliana-AT2G40190.1@1	Athaliana-AT3G60830.1@4	Athaliana-AT5G14450.1@2	Athaliana-AT5G65950.1@6
Arath-5656	Athaliana-AT1G29900.1@2	Athaliana-AT2G40190.1@2	Athaliana-AT3G60830.1@5	Athaliana-AT5G14450.1@3	Athaliana-AT5G65950.1@8
Arath-5660	Athaliana-AT1G29900.1@3	Athaliana-AT2G40730.1@21	Athaliana-AT3G61960.2@1	Athaliana-AT5G14450.1@4	Athaliana-AT5G65950.1@9
Arath-5664	Athaliana-AT1G30000.1@2	Athaliana-AT2G40760.1@2	Athaliana-AT3G62130.1@1	Athaliana-AT5G14450.1@5	Athaliana-AT5G65990.1@1
Arath-5670	Athaliana-AT1G30000.1@4	Athaliana-AT2G40760.1@4	Athaliana-AT4G00450.1@11	Athaliana-AT5G14510.1@2	Athaliana-AT5G65990.1@2
Arath-5699	Athaliana-AT1G30000.1@6	Athaliana-AT2G40760.1@5	Athaliana-AT4G00450.1@12	Athaliana-AT5G14700.1@3	Athaliana-AT5G66680.1@1
Arath-5702	Athaliana-AT1G30010.1@1	Athaliana-AT2G40770.1@1	Athaliana-AT4G00550.1@2	Athaliana-AT5G14950.1@4	Athaliana-AT5G66680.1@3
Arath-5703	Athaliana-AT1G30440.1@3	Athaliana-AT2G40770.1@4	Athaliana-AT4G00550.1@3	Athaliana-AT5G15300.1@1	Athaliana-AT5G66960.1@3
Arath-5716	Athaliana-AT1G31360.1@2	Athaliana-AT2G40770.1@5	Athaliana-AT4G00550.1@4	Athaliana-AT5G15400.1@1	Athaliana-AT5G66960.1@5
Arath-5721	Athaliana-AT1G31360.2@4	Athaliana-AT2G40770.1@6	Athaliana-AT4G00550.1@5	Athaliana-AT5G15400.1@2	Athaliana-AT5G66960.1@8
Arath-5733	Athaliana-AT1G31800.1@5	Athaliana-AT2G40770.1@7	Athaliana-AT4G00740.1@1	Athaliana-AT5G15400.1@4	Athaliana-AT5G66960.1@9
Arath-5744	Athaliana-AT1G33410.1@13	Athaliana-AT2G40770.1@9	Athaliana-AT4G00740.1@2	Athaliana-AT5G15400.1@6	Athaliana-AT5G67430.1@3
Arath-5770	Athaliana-AT1G33410.1@14	Athaliana-AT2G40770.1@11	Athaliana-AT4G00740.1@3	Athaliana-AT5G15540.1@5	
Arath-5772	Athaliana-AT1G33410.1@18	Athaliana-AT2G40770.1@14	Athaliana-AT4G00740.1@4	Athaliana-AT5G15540.1@9	
Arath-5791	Athaliana-AT1G33410.2@9	Athaliana-AT2G40770.1@16	Athaliana-AT4G00740.1@5	Athaliana-AT5G15540.1@10	
Arath-5802	Athaliana-AT1G33410.2@10	Athaliana-AT2G40840.1@2	Athaliana-AT4G00740.1@7	Athaliana-AT5G15540.1@11	
Arath-5815	Athaliana-AT1G33410.2@11	Athaliana-AT2G40840.1@7	Athaliana-AT4G00910.1@1	Athaliana-AT5G15540.2@12	
Arath-5816	Athaliana-AT1G33410.2@16	Athaliana-AT2G40840.1@13	Athaliana-AT4G00910.1@3	Athaliana-AT5G15540.2@16	
Arath-5821	Athaliana-AT1G33410.2@25	Athaliana-AT2G40840.1@21	Athaliana-AT4G00910.1@5	Athaliana-AT5G15540.2@25	
Arath-5822	Athaliana-AT1G47380.1@8	Athaliana-AT2G40860.1@5	Athaliana-AT4G00910.1@6	Athaliana-AT5G15710.1@1	
Arath-5840	Athaliana-AT1G47380.1@9	Athaliana-AT2G40860.1@7	Athaliana-AT4G01037.1@2	Athaliana-AT5G15880.1@1	
AZBL-5841	Athaliana-AT1G47670.1@1	Athaliana-AT2G40860.1@9	Athaliana-AT4G01320.1@7	Athaliana-AT5G15880.1@6	
Arath-5842	Athaliana-AT1G47670.1@5	Athaliana-AT2G40860.1@12	Athaliana-AT4G01570.1@1	Athaliana-AT5G16210.1@10	
Arath-5843	Athaliana-AT1G48090.1@13	Athaliana-AT2G40890.1@2	Athaliana-AT4G01730.1@3	Athaliana-AT5G16210.1@12	
Arath-5849	Athaliana-AT1G48090.1@17	Athaliana-AT2G41190.1@3	Athaliana-AT4G01730.1@9	Athaliana-AT5G16210.1@15	

Arath-5853	Athaliana-AT1G48090.1@22	Athaliana-AT2G41190.1@5	Athaliana-AT4G01880.1@3	Athaliana-AT5G16280.1@5
Arath-5857	Athaliana-AT1G48090.1@23	Athaliana-AT2G41190.1@6	Athaliana-AT4G01880.1@4	Athaliana-AT5G16280.1@25
Arath-5858	Athaliana-AT1G48090.1@29	Athaliana-AT2G41670.1@7	Athaliana-AT4G02030.1@14	Athaliana-AT5G16280.2@20
Arath-5859	Athaliana-AT1G48090.1@30	Athaliana-AT2G41700.1@9	Athaliana-AT4G02030.1@18	Athaliana-AT5G16280.2@22
Arath-5865	Athaliana-AT1G48090.1@33	Athaliana-AT2G41700.1@12	Athaliana-AT4G02030.1@19	Athaliana-AT5G16280.2@27
Arath-5866	Athaliana-AT1G48090.1@58	Athaliana-AT2G41700.1@21	Athaliana-AT4G02260.1@21	Athaliana-AT5G16300.1@1
Arath-5870	Athaliana-AT1G48090.1@62	Athaliana-AT2G41700.1@23	Athaliana-AT4G02260.2@2	Athaliana-AT5G16300.1@3
Arath-5893	Athaliana-AT1G48090.1@63	Athaliana-AT2G41700.1@37	Athaliana-AT4G02460.1@2	Athaliana-AT5G16300.2@4
Arath-5894	Athaliana-AT1G48880.1@1	Athaliana-AT2G41700.2@4	Athaliana-AT4G02460.1@3	Athaliana-AT5G16310.1@4
Arath-5899	Athaliana-AT1G49040.1@8	Athaliana-AT2G41700.2@5	Athaliana-AT4G02460.1@4	Athaliana-AT5G16420.1@1
Arath-5910	Athaliana-AT1G49040.1@26	Athaliana-AT2G41700.2@11	Athaliana-AT4G02680.1@2	Athaliana-AT5G17230.1@2
Arath-5913	Athaliana-AT1G49040.1@30	Athaliana-AT2G41700.2@15	Athaliana-AT4G02730.1@1	Athaliana-AT5G17230.1@6
Arath-5918	Athaliana-AT1G49040.2@11	Athaliana-AT2G42490.1@3	Athaliana-AT4G02730.1@2	Athaliana-AT5G17250.1@13
Arath-5919	Athaliana-AT1G49040.3@22	Athaliana-AT2G42490.1@5	Athaliana-AT4G02780.1@4	Athaliana-AT5G17290.1@8
Arath-5921	Athaliana-AT1G49820.1@2	Athaliana-AT2G42490.1@6	Athaliana-AT4G02780.1@9	Athaliana-AT5G17530.2@5
Arath-5922	Athaliana-AT1G49820.1@4	Athaliana-AT2G42490.1@7	Athaliana-AT4G02990.1@2	Athaliana-AT5G17530.3@7
Arath-5926	Athaliana-AT1G49820.1@5	Athaliana-AT2G42490.1@8	Athaliana-AT4G03020.2@2	Athaliana-AT5G17620.1@9
Arath-5933	Athaliana-AT1G49970.1@1	Athaliana-AT2G42490.1@10	Athaliana-AT4G03220.1@1	Athaliana-AT5G17860.1@1
Arath-5936	Athaliana-AT1G49970.1@3	Athaliana-AT2G42700.2@4	Athaliana-AT4G03220.1@3	Athaliana-AT5G18525.1@3
Arath-5940	Athaliana-AT1G49970.1@8	Athaliana-AT2G42750.1@1	Athaliana-AT4G03260.1@3	Athaliana-AT5G18525.1@4
Arath-5941	Athaliana-AT1G50120.1@1	Athaliana-AT2G42850.1@1	Athaliana-AT4G03260.1@5	Athaliana-AT5G18525.1@7
Arath-5942	Athaliana-AT1G50120.1@6	Athaliana-AT2G42850.1@2	Athaliana-AT4G04350.1@6	Athaliana-AT5G18525.1@9
Arath-5943	Athaliana-AT1G50200.1@19	Athaliana-AT2G42850.1@3	Athaliana-AT4G04350.1@9	Athaliana-AT5G18525.1@11
Arath-5944	Athaliana-AT1G50200.2@2	Athaliana-AT2G43235.1@6	Athaliana-AT4G04350.1@10	Athaliana-AT5G18525.1@12
Arath-5945	Athaliana-AT1G50200.2@21	Athaliana-AT2G43420.1@7	Athaliana-AT4G04350.1@19	Athaliana-AT5G18525.1@13
Arath-5949	Athaliana-AT1G50200.2@22	Athaliana-AT2G43770.1@2	Athaliana-AT4G04350.1@20	Athaliana-AT5G18580.1@12
Arath-5950	Athaliana-AT1G50940.1@2	Athaliana-AT2G43890.1@1	Athaliana-AT4G04640.1@1	Athaliana-AT5G18670.1@2
Arath-5958	Athaliana-AT1G50940.1@3	Athaliana-AT2G43890.1@2	Athaliana-AT4G04970.1@1	Athaliana-AT5G18670.1@3
Arath-5960	Athaliana-AT1G51550.1@1	Athaliana-AT2G43890.1@3	Athaliana-AT4G04970.1@2	Athaliana-AT5G18700.1@8
Arath-5968	Athaliana-AT1G51550.1@2	Athaliana-AT2G45500.2@2	Athaliana-AT4G05090.1@4	Athaliana-AT5G18700.1@9
Arath-5974	Athaliana-AT1G51720.1@2	Athaliana-AT2G45500.2@5	Athaliana-AT4G05090.1@6	Athaliana-AT5G18700.1@10
Arath-5977	Athaliana-AT1G51720.1@4	Athaliana-AT2G46060.1@6	Athaliana-AT4G08150.1@1	Athaliana-AT5G18830.1@3
Arath-5980	Athaliana-AT1G52520.1@2	Athaliana-AT2G46060.1@7	Athaliana-AT4G08150.1@5	Athaliana-AT5G18830.2@1
Arath-5981	Athaliana-AT1G52760.1@1	Athaliana-AT2G46370.3@6	Athaliana-AT4G08920.1@1	Athaliana-AT5G18830.3@9
Arath-5990	Athaliana-AT1G52760.1@2	Athaliana-AT2G46890.1@1	Athaliana-AT4G08920.1@2	Athaliana-AT5G19130.1@4
Arath-6000	Athaliana-AT1G53270.1@1	Athaliana-AT2G46890.1@2	Athaliana-AT4G08920.1@3	Athaliana-AT5G19130.1@6
Arath-6003	Athaliana-AT1G55150.1@8	Athaliana-AT2G46890.1@3	Athaliana-AT4G09140.1@5	Athaliana-AT5G19180.1@3
Arath-6004	Athaliana-AT1G55150.1@9	Athaliana-AT2G47020.1@4	Athaliana-AT4G09140.1@15	Athaliana-AT5G19180.1@4
Arath-6016	Athaliana-AT1G55250.1@6	Athaliana-AT2G47020.1@11	Athaliana-AT4G09750.1@10	Athaliana-AT5G19180.1@9
Arath-6026	Athaliana-AT1G55250.1@9	Athaliana-AT2G47600.1@2	Athaliana-AT4G10020.1@6	Athaliana-AT5G19350.1@2
Arath-6029	Athaliana-AT1G55250.3@4	Athaliana-AT2G47600.1@7	Athaliana-AT4G10950.1@1	Athaliana-AT5G19610.1@2

Arath-6034	Athaliana-AT1G55250.3@14	Athaliana-AT2G47790.1@8	Athaliana-AT4G10950.1@3	Athaliana-AT5G19610.1@3
Arath-6036	Athaliana-AT1G55250.4@7	Athaliana-AT3G01060.1@1	Athaliana-AT4G10950.1@4	Athaliana-AT5G19640.1@3
Arath-6038	Athaliana-AT1G55270.1@2	Athaliana-AT3G01060.1@6	Athaliana-AT4G10950.1@5	Athaliana-AT5G19680.1@2
Arath-6041	Athaliana-AT1G55480.1@1	Athaliana-AT3G01060.2@3	Athaliana-AT4G11120.1@6	Athaliana-AT5G19690.1@16
Arath-6048	Athaliana-AT1G55590.1@4	Athaliana-AT3G01100.1@2	Athaliana-AT4G14940.1@3	Athaliana-AT5G19730.1@1
Arath-6050	Athaliana-AT1G55760.1@4	Athaliana-AT3G01100.1@8	Athaliana-AT4G15420.1@1	Athaliana-AT5G19730.1@4
Arath-6051	Athaliana-AT1G55910.1@1	Athaliana-AT3G01100.2@6	Athaliana-AT4G15420.1@2	Athaliana-AT5G20170.1@2
Arath-6056	Athaliana-AT1G55910.1@2	Athaliana-AT3G01100.2@7	Athaliana-AT4G16144.1@8	Athaliana-AT5G20170.1@7
Arath-6064	Athaliana-AT1G57770.1@2	Athaliana-AT3G01150.1@1	Athaliana-AT4G16144.1@13	Athaliana-AT5G20270.1@3
Arath-6068	Athaliana-AT1G57770.1@7	Athaliana-AT3G01150.1@2	Athaliana-AT4G16340.1@8	Athaliana-AT5G20350.1@1
Arath-6072	Athaliana-AT1G57770.1@9	Athaliana-AT3G01460.1@2	Athaliana-AT4G16340.1@9	Athaliana-AT5G20890.1@9
Arath-6098	Athaliana-AT1G57770.1@10	Athaliana-AT3G01460.1@3	Athaliana-AT4G16340.1@11	Athaliana-AT5G20890.1@10
Arath-6110	Athaliana-AT1G58122.1@1	Athaliana-AT3G01460.1@4	Athaliana-AT4G16340.1@19	Athaliana-AT5G20990.1@6
Arath-6114	Athaliana-AT1G59980.1@9	Athaliana-AT3G01460.1@5	Athaliana-AT4G16340.1@23	Athaliana-AT5G20990.1@7
Arath-6119	Athaliana-AT1G60060.1@3	Athaliana-AT3G01460.1@6	Athaliana-AT4G16340.1@26	Athaliana-AT5G20990.1@9
Arath-6128	Athaliana-AT1G60070.1@5	Athaliana-AT3G01460.1@8	Athaliana-AT4G16340.1@27	Athaliana-AT5G21930.1@1
Arath-6130	Athaliana-AT1G60070.2@14	Athaliana-AT3G01460.1@9	Athaliana-AT4G16340.1@28	Athaliana-AT5G21970.1@2
Arath-6139	Athaliana-AT1G60490.1@5	Athaliana-AT3G01460.1@10	Athaliana-AT4G16340.1@29	Athaliana-AT5G21990.1@2
Arath-6148	Athaliana-AT1G60490.1@11	Athaliana-AT3G01670.1@3	Athaliana-AT4G16340.1@30	Athaliana-AT5G21990.1@9
Arath-6150	Athaliana-AT1G60560.1@2	Athaliana-AT3G01670.1@6	Athaliana-AT4G16420.1@3	Athaliana-AT5G22030.1@2
Arath-6164	Athaliana-AT1G60560.1@3	Athaliana-AT3G02130.1@1	Athaliana-AT4G16420.3@9	Athaliana-AT5G22030.1@8
Arath-6175	Athaliana-AT1G60560.1@5	Athaliana-AT3G02260.1@4	Athaliana-AT4G16570.1@2	Athaliana-AT5G22030.1@11
Arath-6176	Athaliana-AT1G60560.2@4	Athaliana-AT3G02260.1@5	Athaliana-AT4G16570.1@8	Athaliana-AT5G22030.1@13
Arath-6198	Athaliana-AT1G60995.1@1	Athaliana-AT3G02260.1@8	Athaliana-AT4G16660.1@2	Athaliana-AT5G22030.1@14
Arath-6216	Athaliana-AT1G60995.1@4	Athaliana-AT3G02260.1@9	Athaliana-AT4G16660.1@3	Athaliana-AT5G22260.1@3
Arath-6221	Athaliana-AT1G60995.1@15	Athaliana-AT3G02260.1@10	Athaliana-AT4G16660.1@5	Athaliana-AT5G22330.1@2
Arath-6226	Athaliana-AT1G61600.1@1	Athaliana-AT3G02260.1@12	Athaliana-AT4G16660.1@7	Athaliana-AT5G22350.1@1
Arath-6227	Athaliana-AT1G61850.2@1	Athaliana-AT3G02260.1@13	Athaliana-AT4G17090.1@1	Athaliana-AT5G22350.1@2
Arath-6238	Athaliana-AT1G63160.1@6	Athaliana-AT3G02260.1@14	Athaliana-AT4G17090.1@2	Athaliana-AT5G22450.1@2
Arath-6258	Athaliana-AT1G63660.1@1	Athaliana-AT3G02570.1@1	Athaliana-AT4G17090.1@4	Athaliana-AT5G22450.1@3
Arath-6265	Athaliana-AT1G63700.1@11	Athaliana-AT3G02570.1@3	Athaliana-AT4G17140.1@12	Athaliana-AT5G22450.1@4
Arath-6270	Athaliana-AT1G65320.1@1	Athaliana-AT3G02570.1@4	Athaliana-AT4G17140.1@34	Athaliana-AT5G22450.1@7
Arath-6274	Athaliana-AT1G65320.1@2	Athaliana-AT3G02570.1@5	Athaliana-AT4G17140.1@44	Athaliana-AT5G22450.1@12
Arath-6282	Athaliana-AT1G65380.1@1	Athaliana-AT3G03210.1@1	Athaliana-AT4G17140.1@48	Athaliana-AT5G22450.1@16
Arath-6284	Athaliana-AT1G65840.1@8	Athaliana-AT3G03380.1@1	Athaliana-AT4G17140.1@50	Athaliana-AT5G22510.1@2
Arath-6295	Athaliana-AT1G66830.1@1	Athaliana-AT3G03380.1@15	Athaliana-AT4G17140.2@17	Athaliana-AT5G22510.1@7
Arath-6298	Athaliana-AT1G66830.1@2	Athaliana-AT3G03380.1@16	Athaliana-AT4G17140.2@36	Athaliana-AT5G22750.1@1
Arath-6299	Athaliana-AT1G67120.1@4	Athaliana-AT3G03380.1@20	Athaliana-AT4G17140.2@38	Athaliana-AT5G22750.1@6
Ambtr-6303	Athaliana-AT1G67120.1@17	Athaliana-AT3G03380.1@21	Athaliana-AT4G17140.2@39	Athaliana-AT5G22750.1@8
Arath-6318	Athaliana-AT1G67120.1@34	Athaliana-AT3G03710.1@7	Athaliana-AT4G17140.2@53	Athaliana-AT5G22750.1@15
Arath-6320	Athaliana-AT1G67120.1@38	Athaliana-AT3G03710.1@10	Athaliana-AT4G17140.2@54	Athaliana-AT5G22750.1@16

Arath-6363	Athaliana-AT1G67120.1@51	Athaliana-AT3G03710.1@23	Athaliana-AT4G17140.2@58	Athaliana-AT5G22800.1@3
Arath-6366	Athaliana-AT1G67120.1@64	Athaliana-AT3G04340.1@1	Athaliana-AT4G17140.2@62	Athaliana-AT5G22800.1@4
Arath-6373	Athaliana-AT1G67120.1@65	Athaliana-AT3G04340.1@2	Athaliana-AT4G17140.3@8	Athaliana-AT5G22800.1@5
Arath-6376	Athaliana-AT1G67120.1@66	Athaliana-AT3G04340.1@3	Athaliana-AT4G17140.3@28	Athaliana-AT5G22800.1@6
Arath-6378	Athaliana-AT1G67120.1@69	Athaliana-AT3G04340.1@4	Athaliana-AT4G17140.3@31	Athaliana-AT5G22800.1@8
Arath-6379	Athaliana-AT1G67120.1@71	Athaliana-AT3G04340.1@5	Athaliana-AT4G17140.3@35	Athaliana-AT5G22850.1@3
Arath-6383	Athaliana-AT1G67120.1@72	Athaliana-AT3G04340.1@6	Athaliana-AT4G17140.3@41	Athaliana-AT5G22850.1@4
Arath-6384	Athaliana-AT1G67550.1@8	Athaliana-AT3G04340.1@9	Athaliana-AT4G17140.3@45	Athaliana-AT5G23050.1@8
Arath-6387	Athaliana-AT1G67560.1@2	Athaliana-AT3G04340.1@11	Athaliana-AT4G17140.3@59	Athaliana-AT5G23050.1@10
Ambtr-6389	Athaliana-AT1G67560.1@3	Athaliana-AT3G04480.1@4	Athaliana-AT4G17140.3@61	Athaliana-AT5G23060.1@2
Arath-6393	Athaliana-AT1G67560.1@4	Athaliana-AT3G04480.1@11	Athaliana-AT4G17140.3@65	Athaliana-AT5G23110.1@1
Arath-6398	Athaliana-AT1G67560.1@7	Athaliana-AT3G04480.1@12	Athaliana-AT4G17230.1@2	Athaliana-AT5G23110.1@2
Arath-6401	Athaliana-AT1G67560.1@8	Athaliana-AT3G04740.1@1	Athaliana-AT4G17370.1@7	Athaliana-AT5G23110.1@3
Arath-6404	Athaliana-AT1G67560.1@9	Athaliana-AT3G04740.1@2	Athaliana-AT4G17380.1@2	Athaliana-AT5G23110.1@4
Arath-6405	Athaliana-AT1G67840.1@3	Athaliana-AT3G04740.1@6	Athaliana-AT4G17830.2@2	Athaliana-AT5G23110.1@5
Arath-6406	Athaliana-AT1G67840.1@9	Athaliana-AT3G04740.1@7	Athaliana-AT4G18010.2@8	Athaliana-AT5G23110.1@6
Arath-6407	Athaliana-AT1G68050.1@2	Athaliana-AT3G04740.1@8	Athaliana-AT4G18060.1@6	Athaliana-AT5G23110.1@7
Arath-6412	Athaliana-AT1G68570.1@2	Athaliana-AT3G05350.1@4	Athaliana-AT4G18340.1@2	Athaliana-AT5G23110.1@8
Arath-6420	Athaliana-AT1G68570.1@3	Athaliana-AT3G05350.1@8	Athaliana-AT4G18340.1@3	Athaliana-AT5G23110.1@10
Arath-6432	Athaliana-AT1G68740.1@3	Athaliana-AT3G06270.1@1	Athaliana-AT4G18830.1@1	Athaliana-AT5G23110.1@11
Arath-6439	Athaliana-AT1G68740.1@7	Athaliana-AT3G06483.1@2	Athaliana-AT4G18950.1@1	Athaliana-AT5G23300.1@3
Arath-6447	Athaliana-AT1G68830.1@9	Athaliana-AT3G06483.1@3	Athaliana-AT4G19180.1@2	Athaliana-AT5G23300.1@4
Arath-6448	Athaliana-AT1G68930.1@1	Athaliana-AT3G06530.2@31	Athaliana-AT4G19460.1@1	Athaliana-AT5G23300.1@5
Arath-6449	Athaliana-AT1G69860.1@2	Athaliana-AT3G06530.2@36	Athaliana-AT4G20050.1@3	Athaliana-AT5G23340.1@1
Arath-6450	Athaliana-AT1G70260.1@4	Athaliana-AT3G06530.3@29	Athaliana-AT4G20050.2@3	Athaliana-AT5G23340.1@2
Arath-6454	Athaliana-AT1G70570.2@2	Athaliana-AT3G06530.3@30	Athaliana-AT4G20060.1@2	Athaliana-AT5G23880.1@10
Arath-6457	Athaliana-AT1G70570.2@10	Athaliana-AT3G06530.3@34	Athaliana-AT4G20060.1@3	Athaliana-AT5G23880.1@11
Arath-6458	Athaliana-AT1G70820.1@2	Athaliana-AT3G06530.3@37	Athaliana-AT4G20060.1@6	Athaliana-AT5G24240.1@2
Arath-6459	Athaliana-AT1G70820.1@3	Athaliana-AT3G06810.1@5	Athaliana-AT4G20070.1@9	Athaliana-AT5G24810.1@17
Arath-6460	Athaliana-AT1G70820.1@4	Athaliana-AT3G06810.1@10	Athaliana-AT4G20090.1@1	Athaliana-AT5G24810.1@19
Arath-6462	Athaliana-AT1G71220.1@10	Athaliana-AT3G06810.1@11	Athaliana-AT4G20850.1@1	Athaliana-AT5G25230.1@1
Arath-6483	Athaliana-AT1G71220.1@24	Athaliana-AT3G06810.1@14	Athaliana-AT4G20850.1@11	Athaliana-AT5G25230.1@3
Arath-6487	Athaliana-AT1G71220.2@2	Athaliana-AT3G06810.1@17	Athaliana-AT4G20850.1@16	Athaliana-AT5G25230.1@7
Arath-6488	Athaliana-AT1G71220.2@28	Athaliana-AT3G06860.1@8	Athaliana-AT4G20850.1@17	Athaliana-AT5G26570.1@10
Arath-6492	Athaliana-AT1G71220.2@36	Athaliana-AT3G06860.1@13	Athaliana-AT4G21350.1@1	Athaliana-AT5G26570.1@19
Arath-6494	Athaliana-AT1G71220.2@37	Athaliana-AT3G06880.2@6	Athaliana-AT4G21680.1@3	Athaliana-AT5G26820.1@4
Arath-6496	Athaliana-AT1G72280.1@3	Athaliana-AT3G06880.2@7	Athaliana-AT4G21680.1@4	Athaliana-AT5G27970.1@23
Arath-6498	Athaliana-AT1G72280.1@7	Athaliana-AT3G06920.1@2	Athaliana-AT4G21770.1@3	Athaliana-AT5G27970.1@40
Arath-6500	Athaliana-AT1G72500.1@3	Athaliana-AT3G07080.1@1	Athaliana-AT4G21770.1@9	Athaliana-AT5G27970.2@2
Arath-6506	Athaliana-AT1G72500.1@10	Athaliana-AT3G07080.1@2	Athaliana-AT4G22720.1@2	Athaliana-AT5G30510.1@1
Arath-6507	Athaliana-AT1G72970.1@2	Athaliana-AT3G07080.1@3	Athaliana-AT4G23540.1@1	Athaliana-AT5G30510.1@2

Arath-6514	Athaliana-AT1G72970.1@4	Athaliana-AT3G07140.2@2	Athaliana-AT4G23540.1@2	Athaliana-AT5G30510.1@4
Arath-6526	Athaliana-AT1G72970.1@5	Athaliana-AT3G07140.2@4	Athaliana-AT4G23540.1@5	Athaliana-AT5G35410.1@7
Arath-6527	Athaliana-AT1G73180.1@3	Athaliana-AT3G07420.1@1	Athaliana-AT4G23540.1@7	Athaliana-AT5G35460.1@7
Arath-6528	Athaliana-AT1G73180.2@5	Athaliana-AT3G07420.1@2	Athaliana-AT4G23540.1@8	Athaliana-AT5G35460.1@8
Arath-6531	Athaliana-AT1G73930.2@2	Athaliana-AT3G07420.1@3	Athaliana-AT4G23940.1@1	Athaliana-AT5G35560.1@3
Arath-6532	Athaliana-AT1G73930.2@3	Athaliana-AT3G08670.1@4	Athaliana-AT4G23940.1@3	Athaliana-AT5G35560.1@7
Arath-6533	Athaliana-AT1G73960.1@5	Athaliana-AT3G08760.1@2	Athaliana-AT4G23940.1@9	Athaliana-AT5G35560.1@10
Arath-6538	Athaliana-AT1G73960.2@2	Athaliana-AT3G08760.1@3	Athaliana-AT4G23940.1@11	Athaliana-AT5G36880.2@20
Arath-6544	Athaliana-AT1G73960.2@13	Athaliana-AT3G08760.1@4	Athaliana-AT4G24190.1@10	Athaliana-AT5G36890.1@13
Arath-6550	Athaliana-AT1G73960.2@24	Athaliana-AT3G08950.1@4	Athaliana-AT4G24190.1@14	Athaliana-AT5G36890.2@7
Arath-6552	Athaliana-AT1G73960.2@25	Athaliana-AT3G08960.1@6	Athaliana-AT4G24190.2@9	Athaliana-AT5G36890.2@9
Arath-6557	Athaliana-AT1G73990.1@2	Athaliana-AT3G08960.1@8	Athaliana-AT4G24220.1@2	Athaliana-AT5G37830.1@2
Ambtr-6559	Athaliana-AT1G73990.1@13	Athaliana-AT3G08960.1@19	Athaliana-AT4G24610.2@6	Athaliana-AT5G38530.1@2
Arath-6563	Athaliana-AT1G74460.1@3	Athaliana-AT3G09090.1@6	Athaliana-AT4G24610.2@13	Athaliana-AT5G38530.1@3
Arath-6565	Athaliana-AT1G74680.1@1	Athaliana-AT3G09090.3@8	Athaliana-AT4G24610.2@18	Athaliana-AT5G38530.1@4
Arath-6570	Athaliana-AT1G75200.1@1	Athaliana-AT3G09090.3@9	Athaliana-AT4G24610.2@19	Athaliana-AT5G38530.1@6
Arath-6572	Athaliana-AT1G75200.1@3	Athaliana-AT3G09090.3@10	Athaliana-AT4G24620.2@1	Athaliana-AT5G39040.1@16
Arath-6601	Athaliana-AT1G75200.1@4	Athaliana-AT3G09090.3@12	Athaliana-AT4G24740.1@2	Athaliana-AT5G39500.1@1
Arath-6620	Athaliana-AT1G75200.1@7	Athaliana-AT3G09650.1@1	Athaliana-AT4G24790.1@3	Athaliana-AT5G39500.1@2
Arath-6631	Athaliana-AT1G76400.1@3	Athaliana-AT3G10030.2@1	Athaliana-AT4G24790.1@4	Athaliana-AT5G39710.1@1
Arath-6636	Athaliana-AT1G76400.1@4	Athaliana-AT3G10030.2@5	Athaliana-AT4G24790.1@6	Athaliana-AT5G39830.1@8
Arath-6639	Athaliana-AT1G76400.1@9	Athaliana-AT3G10230.1@1	Athaliana-AT4G24790.2@2	Athaliana-AT5G40405.1@1
Arath-6641	Athaliana-AT1G76400.1@10	Athaliana-AT3G10380.1@3	Athaliana-AT4G24830.1@6	Athaliana-AT5G40440.1@3
Arath-6649	Athaliana-AT1G76730.1@3	Athaliana-AT3G10380.1@7	Athaliana-AT4G24830.1@10	Athaliana-AT5G40440.1@4
Arath-6652	Athaliana-AT1G77405.1@1	Athaliana-AT3G10380.1@10	Athaliana-AT4G24830.2@8	Athaliana-AT5G40440.1@9
Arath-6660	Athaliana-AT1G78280.1@5	Athaliana-AT3G10380.1@19	Athaliana-AT4G26090.1@1	Athaliana-AT5G40480.1@2
Arath-6667	Athaliana-AT1G78280.1@6	Athaliana-AT3G10700.1@14	Athaliana-AT4G26750.1@6	Athaliana-AT5G40480.1@3
Arath-6679	Athaliana-AT1G78280.1@8	Athaliana-AT3G11220.2@3	Athaliana-AT4G27640.1@1	Athaliana-AT5G40480.1@4
Arath-6685	Athaliana-AT1G78280.1@11	Athaliana-AT3G11460.1@1	Athaliana-AT4G27640.1@5	Athaliana-AT5G40480.1@5
Arath-6689	Athaliana-AT1G79080.1@1	Athaliana-AT3G11540.1@12	Athaliana-AT4G27640.1@6	Athaliana-AT5G40480.1@6
Arath-6705	Athaliana-AT1G79150.1@3	Athaliana-AT3G11540.1@13	Athaliana-AT4G27640.1@8	Athaliana-AT5G40480.1@10
Arath-6713	Athaliana-AT1G79150.1@4	Athaliana-AT3G11540.1@16	Athaliana-AT4G27640.1@10	Athaliana-AT5G40480.1@12
Arath-6717	Athaliana-AT1G79150.1@10	Athaliana-AT3G11540.2@2	Athaliana-AT4G27790.1@1	Athaliana-AT5G40480.1@15
Arath-6732	Athaliana-AT1G79150.1@13	Athaliana-AT3G11540.2@3	Athaliana-AT4G28080.1@9	Athaliana-AT5G40480.1@24
Arath-6733	Athaliana-AT1G79560.1@1	Athaliana-AT3G11960.1@7	Athaliana-AT4G28080.1@11	Athaliana-AT5G40480.1@32
Arath-6738	Athaliana-AT1G79560.1@2	Athaliana-AT3G11960.1@9	Athaliana-AT4G28080.1@14	Athaliana-AT5G40740.1@1
Arath-6746	Athaliana-AT1G79560.1@3	Athaliana-AT3G11960.2@6	Athaliana-AT4G28080.1@19	Athaliana-AT5G40740.1@8
Arath-6779	Athaliana-AT1G79560.1@4	Athaliana-AT3G11964.1@12	Athaliana-AT4G28080.1@23	Athaliana-AT5G40740.1@11
Arath-6780	Athaliana-AT1G79560.1@6	Athaliana-AT3G11964.1@34	Athaliana-AT4G28220.1@3	Athaliana-AT5G40740.1@12
Arath-6782	Athaliana-AT1G79560.1@10	Athaliana-AT3G12280.2@10	Athaliana-AT4G28220.1@4	Athaliana-AT5G40820.1@4
Arath-6785	Athaliana-AT1G80350.1@2	Athaliana-AT3G12590.1@1	Athaliana-AT4G28220.1@5	Athaliana-AT5G40820.1@7

Arath-6791	Athaliana-AT1G80350.1@3	Athaliana-AT3G12590.1@4	Athaliana-AT4G28220.1@6	Athaliana-AT5G40820.1@10
Arath-6792	Athaliana-AT1G80350.1@4	Athaliana-AT3G12590.1@5	Athaliana-AT4G29010.1@8	Athaliana-AT5G41800.1@2
Arath-6797	Athaliana-AT1G80350.1@7	Athaliana-AT3G12590.1@7	Athaliana-AT4G29310.1@1	Athaliana-AT5G41800.1@7
Arath-6825	Athaliana-AT1G80360.1@9	Athaliana-AT3G12590.1@9	Athaliana-AT4G29310.1@2	Athaliana-AT5G43920.1@1
Arath-6848	Athaliana-AT1G80410.1@17	Athaliana-AT3G12590.1@11	Athaliana-AT4G29380.1@1	Athaliana-AT5G43920.1@3
Arath-6854	Athaliana-AT1G80460.1@3	Athaliana-AT3G12590.1@12	Athaliana-AT4G29380.1@9	Athaliana-AT5G43920.1@4
Ambtr-6859	Athaliana-AT1G80460.1@4	Athaliana-AT3G12590.1@13	Athaliana-AT4G29810.1@4	Athaliana-AT5G44000.1@1
Arath-6860	Athaliana-AT2G01070.2@2	Athaliana-AT3G12590.1@14	Athaliana-AT4G29810.2@6	Athaliana-AT5G44000.1@2
Arath-6864	Athaliana-AT2G02560.1@4	Athaliana-AT3G12610.1@1	Athaliana-AT4G30490.1@13	Athaliana-AT5G44240.2@12
Arath-6865	Athaliana-AT2G02560.1@21	Athaliana-AT3G12810.1@15	Athaliana-AT4G30600.1@2	Athaliana-AT5G44240.2@25
Arath-6875	Athaliana-AT2G02560.2@22	Athaliana-AT3G12810.1@18	Athaliana-AT4G30720.1@13	Athaliana-AT5G44370.1@2
Arath-6882	Athaliana-AT2G04270.1@6	Athaliana-AT3G12810.1@19	Athaliana-AT4G30790.1@1	Athaliana-AT5G45780.1@9
Arath-6883	Athaliana-AT2G04270.3@2	Athaliana-AT3G13490.1@10	Athaliana-AT4G30790.1@2	Athaliana-AT5G45780.1@11
Arath-6886	Athaliana-AT2G04270.5@3	Athaliana-AT3G14120.1@17	Athaliana-AT4G30790.1@3	Athaliana-AT5G45900.1@2
Arath-6893	Athaliana-AT2G04270.5@4	Athaliana-AT3G14120.1@20	Athaliana-AT4G30790.1@4	Athaliana-AT5G46070.1@7
Arath-6909	Athaliana-AT2G04660.1@3	Athaliana-AT3G14120.3@12	Athaliana-AT4G30790.1@5	Athaliana-AT5G46070.1@12
Arath-6913	Athaliana-AT2G04660.1@4	Athaliana-AT3G14470.1@1	Athaliana-AT4G31200.1@3	Athaliana-AT5G46070.1@14
Arath-6914	Athaliana-AT2G04660.1@6	Athaliana-AT3G14910.1@1	Athaliana-AT4G31200.2@2	Athaliana-AT5G46100.1@1
Arath-6924	Athaliana-AT2G04660.1@7	Athaliana-AT3G14910.1@3	Athaliana-AT4G31200.3@3	Athaliana-AT5G46220.1@2
Arath-6933	Athaliana-AT2G04842.1@6	Athaliana-AT3G14910.1@8	Athaliana-AT4G31540.1@1	Athaliana-AT5G46220.1@3
Arath-6946	Athaliana-AT2G04842.1@7	Athaliana-AT3G15380.1@1	Athaliana-AT4G31770.1@4	Athaliana-AT5G46580.1@1
Arath-6947	Athaliana-AT2G07360.1@5	Athaliana-AT3G15380.1@2	Athaliana-AT4G31770.1@5	Athaliana-AT5G47010.1@1
Arath-6954	Athaliana-AT2G07360.1@15	Athaliana-AT3G15380.1@4	Athaliana-AT4G31850.1@1	Athaliana-AT5G47010.1@2
Arath-6955	Athaliana-AT2G07360.1@17	Athaliana-AT3G15380.1@8	Athaliana-AT4G32430.1@1	Athaliana-AT5G47010.1@24
Arath-6958	Athaliana-AT2G07360.1@19	Athaliana-AT3G15380.1@10	Athaliana-AT4G32910.1@18	Athaliana-AT5G47040.1@6
Arath-6961	Athaliana-AT2G07360.2@13	Athaliana-AT3G15550.1@7	Athaliana-AT4G33080.2@5	Athaliana-AT5G47040.1@9
Ambtr-6962	Athaliana-AT2G07360.2@14	Athaliana-AT3G16150.1@2	Athaliana-AT4G33210.1@2	Athaliana-AT5G47040.1@10
Arath-6968	Athaliana-AT2G07360.2@18	Athaliana-AT3G16150.1@4	Athaliana-AT4G33210.1@5	Athaliana-AT5G47040.1@12
Arath-6969	Athaliana-AT2G07360.2@21	Athaliana-AT3G16270.1@2	Athaliana-AT4G33210.1@6	Athaliana-AT5G47040.1@13
Arath-6977	Athaliana-AT2G07360.2@25	Athaliana-AT3G16270.1@3	Athaliana-AT4G33210.1@13	Athaliana-AT5G47040.1@14
Arath-6978	Athaliana-AT2G13370.1@2	Athaliana-AT3G16270.1@5	Athaliana-AT4G33210.1@14	Athaliana-AT5G47040.1@15
Arath-6979	Athaliana-AT2G13370.1@4	Athaliana-AT3G16290.1@1	Athaliana-AT4G33330.2@2	Athaliana-AT5G47690.1@2
Arath-6992	Athaliana-AT2G13370.1@5	Athaliana-AT3G16910.1@2	Athaliana-AT4G33410.1@2	Athaliana-AT5G47690.1@3
Arath-6995	Athaliana-AT2G13370.1@14	Athaliana-AT3G16910.1@3	Athaliana-AT4G33410.1@3	Athaliana-AT5G47690.1@6
Arath-7013	Athaliana-AT2G13370.1@19	Athaliana-AT3G17640.1@1	Athaliana-AT4G33440.1@3	Athaliana-AT5G47690.1@12
Arath-7021	Athaliana-AT2G13370.1@20	Athaliana-AT3G17810.1@1	Athaliana-AT4G33440.1@5	Athaliana-AT5G47690.2@11
Arath-7024	Athaliana-AT2G13370.1@23	Athaliana-AT3G17810.1@2	Athaliana-AT4G33760.1@9	Athaliana-AT5G47690.3@5
Arath-7028	Athaliana-AT2G13370.1@25	Athaliana-AT3G17810.1@5	Athaliana-AT4G33760.1@10	Athaliana-AT5G47780.1@6
Arath-7029	Athaliana-AT2G13370.1@28	Athaliana-AT3G17830.1@5	Athaliana-AT4G33945.1@3	Athaliana-AT5G47780.1@7
Arath-7067	Athaliana-AT2G13370.1@29	Athaliana-AT3G17880.1@10	Athaliana-AT4G34260.1@3	Athaliana-AT5G47780.1@8
Arath-7111	Athaliana-AT2G13370.1@31	Athaliana-AT3G17900.1@4	Athaliana-AT4G34260.1@6	Athaliana-AT5G48270.1@1

Arath-7128	Athaliana-AT2G13540.1@9	Athaliana-AT3G17900.1@5	Athaliana-AT4G34260.1@9	Athaliana-AT5G48385.1@3
Arath-7135	Athaliana-AT2G13540.1@15	Athaliana-AT3G17900.1@9	Athaliana-AT4G34310.1@1	Athaliana-AT5G48470.1@1
Arath-7136	Athaliana-AT2G13540.1@16	Athaliana-AT3G18290.1@1	Athaliana-AT4G34310.1@2	Athaliana-AT5G48520.1@4
Arath-7141	Athaliana-AT2G13610.1@1	Athaliana-AT3G18290.1@2	Athaliana-AT4G34310.1@3	Athaliana-AT5G48800.1@3
Arath-7174	Athaliana-AT2G15620.1@2	Athaliana-AT3G18290.1@3	Athaliana-AT4G34350.1@1	Athaliana-AT5G48800.1@4
Arath-7194	Athaliana-AT2G15620.1@3	Athaliana-AT3G18290.1@4	Athaliana-AT4G34350.1@2	Athaliana-AT5G49030.2@2
Arath-7241	Athaliana-AT2G15620.1@4	Athaliana-AT3G18290.1@5	Athaliana-AT4G34350.1@3	Athaliana-AT5G49030.2@10
Arath-7273	Athaliana-AT2G15695.1@5	Athaliana-AT3G18290.1@7	Athaliana-AT4G34350.1@8	Athaliana-AT5G49030.2@11
Arath-7279	Athaliana-AT2G15695.1@7	Athaliana-AT3G18290.1@9	Athaliana-AT4G34850.1@2	Athaliana-AT5G49030.2@20
Arath-7296	Athaliana-AT2G16440.1@2	Athaliana-AT3G18290.1@11	Athaliana-AT4G34850.1@3	Athaliana-AT5G49030.3@22
Arath-7313	Athaliana-AT2G16880.1@1	Athaliana-AT3G18524.1@4	Athaliana-AT4G35560.2@13	Athaliana-AT5G49070.1@1
Arath-7324	Athaliana-AT2G17020.1@4	Athaliana-AT3G18524.1@5	Athaliana-AT4G35760.1@3	Athaliana-AT5G49430.1@1
Arath-7325	Athaliana-AT2G17510.1@9	Athaliana-AT3G18730.1@1	Athaliana-AT4G35850.1@7	Athaliana-AT5G49430.1@3
Arath-7331	Athaliana-AT2G17510.2@8	Athaliana-AT3G18730.1@2	Athaliana-AT4G35870.1@1	Athaliana-AT5G49430.1@19
Arath-7333	Athaliana-AT2G17510.2@13	Athaliana-AT3G18730.1@3	Athaliana-AT4G35880.1@3	Athaliana-AT5G49430.1@20
Arath-7336	Athaliana-AT2G17760.1@3	Athaliana-AT3G18730.1@6	Athaliana-AT4G35880.1@4	Athaliana-AT5G49430.1@21
Arath-7361	Athaliana-AT2G18710.1@3	Athaliana-AT3G18730.1@9	Athaliana-AT4G36180.1@1	Athaliana-AT5G49810.1@2
Arath-7363	Athaliana-AT2G18760.1@2	Athaliana-AT3G19180.1@10	Athaliana-AT4G36180.1@2	Athaliana-AT5G49810.1@3
Arath-7367	Athaliana-AT2G18760.1@3	Athaliana-AT3G19180.2@6	Athaliana-AT4G36390.1@2	Athaliana-AT5G49810.1@5
Arath-7572	Athaliana-AT2G18760.1@4	Athaliana-AT3G19210.1@4	Athaliana-AT4G36390.1@3	Athaliana-AT5G49810.1@6
Arath-7577	Athaliana-AT2G18760.1@5	Athaliana-AT3G19210.1@19	Athaliana-AT4G36630.1@1	Athaliana-AT5G49810.1@7
Arath-7583	Athaliana-AT2G20050.2@1	Athaliana-AT3G19210.2@2	Athaliana-AT4G36630.1@2	Athaliana-AT5G49810.1@9
Arath-7602	Athaliana-AT2G20190.1@13	Athaliana-AT3G19553.1@2	Athaliana-AT4G36630.1@6	Athaliana-AT5G49810.1@10
Arath-7628	Athaliana-AT2G20190.1@14	Athaliana-AT3G19553.1@3	Athaliana-AT4G36630.1@7	Athaliana-AT5G49810.1@11
Athaliana-AT1G01220.1@2	Athaliana-AT2G21470.1@4	Athaliana-AT3G19630.1@1	Athaliana-AT4G36630.1@8	Athaliana-AT5G49830.1@4
Athaliana-AT1G01220.1@6	Athaliana-AT2G21470.1@6	Athaliana-AT3G19810.1@2	Athaliana-AT4G36630.1@9	Athaliana-AT5G49830.1@5
Athaliana-AT1G01220.1@7	Athaliana-AT2G21470.2@7	Athaliana-AT3G19810.1@3	Athaliana-AT4G36630.1@10	Athaliana-AT5G49830.3@4
Athaliana-AT1G01770.1@8	Athaliana-AT2G21470.2@9	Athaliana-AT3G19970.1@3	Athaliana-AT4G36630.1@12	Athaliana-AT5G49890.1@5
Athaliana-AT1G01770.1@11	Athaliana-AT2G21610.1@4	Athaliana-AT3G19970.1@6	Athaliana-AT4G36790.1@2	Athaliana-AT5G49890.1@6
Athaliana-AT1G01770.1@14	Athaliana-AT2G21610.1@5	Athaliana-AT3G19990.1@2	Athaliana-AT4G36790.1@5	Athaliana-AT5G50160.1@3
Athaliana-AT1G01910.5@1	Athaliana-AT2G22120.1@6	Athaliana-AT3G20050.1@9	Athaliana-AT4G37030.1@3	Athaliana-AT5G50170.1@2
Athaliana-AT1G02400.1@2	Athaliana-AT2G23140.1@2	Athaliana-AT3G20240.1@3	Athaliana-AT4G38010.1@1	Athaliana-AT5G50170.1@4
Athaliana-AT1G02420.1@1	Athaliana-AT2G23140.2@4	Athaliana-AT3G20240.1@4	Athaliana-AT4G39470.1@4	Athaliana-AT5G50170.1@6
Athaliana-AT1G02860.1@3	Athaliana-AT2G24230.1@1	Athaliana-AT3G20260.1@1	Athaliana-AT4G39850.1@3	Athaliana-AT5G50210.1@1
Athaliana-AT1G02860.2@2	Athaliana-AT2G25660.1@1	Athaliana-AT3G20260.1@2	Athaliana-AT4G39850.1@22	Athaliana-AT5G50210.1@2
Athaliana-AT1G02970.1@4	Athaliana-AT2G25660.1@2	Athaliana-AT3G20260.1@3	Athaliana-AT4G39850.2@4	Athaliana-AT5G50210.1@3
Athaliana-AT1G02970.1@9	Athaliana-AT2G25660.1@7	Athaliana-AT3G20260.1@4	Athaliana-AT4G39850.2@6	Athaliana-AT5G50210.1@4
Athaliana-AT1G03090.2@1	Athaliana-AT2G25660.1@10	Athaliana-AT3G20630.1@4	Athaliana-AT4G39850.2@10	Athaliana-AT5G50210.1@5
Athaliana-AT1G03090.2@11	Athaliana-AT2G25660.1@15	Athaliana-AT3G20630.1@12	Athaliana-AT4G39850.3@5	Athaliana-AT5G50320.1@5
Athaliana-AT1G03090.2@14	Athaliana-AT2G25660.1@18	Athaliana-AT3G20920.2@5	Athaliana-AT4G39850.3@9	Athaliana-AT5G50320.1@6
Athaliana-AT1G03100.1@1	Athaliana-AT2G25660.1@19	Athaliana-AT3G21420.1@2	Athaliana-AT5G01360.1@4	Athaliana-AT5G51070.1@1

Athaliana-AT1G03190.1@2	Athaliana-AT2G25660.1@20	Athaliana-AT3G21420.1@3	Athaliana-AT5G01380.1@2	Athaliana-AT5G51070.1@2
Athaliana-AT1G03190.1@3	Athaliana-AT2G25660.1@21	Athaliana-AT3G21420.1@4	Athaliana-AT5G02270.1@6	Athaliana-AT5G51070.1@3
Athaliana-AT1G03390.1@1	Athaliana-AT2G25660.1@22	Athaliana-AT3G21720.1@2	Athaliana-AT5G02480.1@1	Athaliana-AT5G51070.1@7
Athaliana-AT1G04110.1@1	Athaliana-AT2G25660.1@23	Athaliana-AT3G22170.1@2	Athaliana-AT5G02810.1@7	Athaliana-AT5G51070.1@9
Athaliana-AT1G04690.1@1	Athaliana-AT2G25730.1@1	Athaliana-AT3G22980.1@1	Athaliana-AT5G02820.1@1	Athaliana-AT5G51070.1@10
Athaliana-AT1G04690.1@2	Athaliana-AT2G25730.1@3	Athaliana-AT3G23020.1@1	Athaliana-AT5G02820.1@2	Athaliana-AT5G51070.1@11
Athaliana-AT1G04730.1@4	Athaliana-AT2G25730.1@5	Athaliana-AT3G23430.1@4	Athaliana-AT5G03070.1@2	Athaliana-AT5G51070.1@12
Athaliana-AT1G04730.1@18	Athaliana-AT2G25730.1@13	Athaliana-AT3G23430.1@9	Athaliana-AT5G03070.1@5	Athaliana-AT5G51150.1@5
Athaliana-AT1G04910.1@7	Athaliana-AT2G25730.1@14	Athaliana-AT3G24170.3@6	Athaliana-AT5G03070.1@10	Athaliana-AT5G51150.1@8
Athaliana-AT1G04910.1@9	Athaliana-AT2G25730.1@15	Athaliana-AT3G25430.1@7	Athaliana-AT5G03280.1@2	Athaliana-AT5G51200.1@9
Athaliana-AT1G04970.1@1	Athaliana-AT2G25730.1@16	Athaliana-AT3G25430.1@8	Athaliana-AT5G03280.1@5	Athaliana-AT5G51200.1@19
Athaliana-AT1G04970.1@6	Athaliana-AT2G25730.1@17	Athaliana-AT3G25660.1@2	Athaliana-AT5G03280.1@6	Athaliana-AT5G51200.1@26
Athaliana-AT1G04970.2@2	Athaliana-AT2G25730.1@18	Athaliana-AT3G26090.1@6	Athaliana-AT5G03280.1@7	Athaliana-AT5G51200.1@29
Athaliana-AT1G05850.1@2	Athaliana-AT2G25730.1@19	Athaliana-AT3G26090.1@7	Athaliana-AT5G03280.1@8	Athaliana-AT5G51200.1@36
Athaliana-AT1G05850.1@4	Athaliana-AT2G25730.1@27	Athaliana-AT3G26410.1@2	Athaliana-AT5G03430.1@8	Athaliana-AT5G51340.1@2
Athaliana-AT1G05910.1@2	Athaliana-AT2G25730.1@32	Athaliana-AT3G26410.1@5	Athaliana-AT5G04360.1@6	Athaliana-AT5G51340.1@4
Athaliana-AT1G05910.1@4	Athaliana-AT2G25730.2@2	Athaliana-AT3G26670.2@13	Athaliana-AT5G04480.1@7	Athaliana-AT5G51340.1@10
Athaliana-AT1G05910.1@5	Athaliana-AT2G25730.2@8	Athaliana-AT3G26670.3@8	Athaliana-AT5G04480.1@8	Athaliana-AT5G51540.1@7
Athaliana-AT1G05910.1@7	Athaliana-AT2G25730.2@24	Athaliana-AT3G26700.1@3	Athaliana-AT5G04480.1@14	Athaliana-AT5G51690.1@1
Athaliana-AT1G05910.1@8	Athaliana-AT2G25730.2@27	Athaliana-AT3G26700.1@6	Athaliana-AT5G04480.2@1	Athaliana-AT5G51690.1@4
Athaliana-AT1G05910.1@10	Athaliana-AT2G25730.2@28	Athaliana-AT3G26700.1@8	Athaliana-AT5G04480.2@6	Athaliana-AT5G51750.1@1
Athaliana-AT1G05960.2@14	Athaliana-AT2G25800.1@1	Athaliana-AT3G27670.1@1	Athaliana-AT5G04480.2@9	Athaliana-AT5G51830.1@4
Athaliana-AT1G06260.1@2	Athaliana-AT2G25800.1@2	Athaliana-AT3G27670.1@3	Athaliana-AT5G04480.2@10	Athaliana-AT5G51970.2@6
Athaliana-AT1G06270.1@2	Athaliana-AT2G25800.1@5	Athaliana-AT3G27670.1@16	Athaliana-AT5G04550.1@2	Athaliana-AT5G52280.1@2
Athaliana-AT1G06440.1@1	Athaliana-AT2G26170.1@3	Athaliana-AT3G27670.1@17	Athaliana-AT5G04710.1@3	Athaliana-AT5G52280.1@6
Athaliana-AT1G06560.1@2	Athaliana-AT2G26180.1@2	Athaliana-AT3G27730.1@2	Athaliana-AT5G04710.1@5	Athaliana-AT5G52580.2@7
Athaliana-AT1G06560.1@3	Athaliana-AT2G26180.1@5	Athaliana-AT3G27730.1@4	Athaliana-AT5G04710.1@7	Athaliana-AT5G52580.2@16
Athaliana-AT1G06560.1@8	Athaliana-AT2G26200.1@11	Athaliana-AT3G27730.1@9	Athaliana-AT5G04930.1@1	Athaliana-AT5G52850.1@1
Athaliana-AT1G06590.1@4	Athaliana-AT2G26200.1@12	Athaliana-AT3G27730.1@10	Athaliana-AT5G04930.1@5	Athaliana-AT5G53000.1@8
Athaliana-AT1G06590.1@8	Athaliana-AT2G26690.1@5	Athaliana-AT3G27730.1@18	Athaliana-AT5G04930.1@6	Athaliana-AT5G53000.1@9
Athaliana-AT1G06590.1@10	Athaliana-AT2G26690.1@6	Athaliana-AT3G27730.1@19	Athaliana-AT5G05200.1@5	Athaliana-AT5G53320.1@2
Athaliana-AT1G06590.1@12	Athaliana-AT2G26780.1@19	Athaliana-AT3G27730.1@20	Athaliana-AT5G05200.1@7	Athaliana-AT5G53480.1@2
Athaliana-AT1G06590.1@18	Athaliana-AT2G26780.1@20	Athaliana-AT3G27820.1@5	Athaliana-AT5G05200.1@9	Athaliana-AT5G53480.1@3
Athaliana-AT1G06710.1@1	Athaliana-AT2G26780.1@22	Athaliana-AT3G27820.1@7	Athaliana-AT5G05200.1@11	Athaliana-AT5G53580.1@2

Table S3. Description of the features of the *C. pendulus* chloroplast genome.

##gff-version	3							
##source-version	GeSeq	2.03						
##sequence-region	42	1	154620					
42	GeSeq	region	1	154620	.	+	0	ID=Catolobus_pendulus;Dbxref=taxon:42;Is_circular=true;Name=Pltd;genome=chloroplast;mol_type=genomic-DNA
42	OGDRAW	sequence_feature	1	83805	.	+	1	ID=misc_feature-ogdraw_lsc;gbkey=misc_feature
42	blatN	gene	3	75	100	-	1	ID=gene-blatn_trnH_1;gbkey=gene;gene=trnH;gene_biotype=tRNA
42	blatN	tRNA	3	75	.	-	1	ID=trna-blatn_trnH_1;gbkey=tRNA;gene=trnH
42	blatX	gene	344	1405	99.2	-	1	ID=gene-blatx_psbA_1;gbkey=gene;gene=psbA;gene_biotype=protein_coding
42	blatX	CDS	344	1405	.	-	1	ID=cds-blatx_psbA_1;gbkey=CDS;gene=psbA
42	blatN	gene	1675	4312	65.3	-	1	ID=gene-blatn_trnK_1;gbkey=gene;gene=trnK;gene_biotype=tRNA
42	blatN	tRNA	1675	1709	.	-	1	ID=trna-blatn_trnK_1;gbkey=tRNA;gene=trnK
42	blatN	exon	1675	1709	.	-	1	ID=blatn_trnK_1_exon_2;Parent=gene-blatn_trnK_1;gbkey=exon;gene=trnK
42	blatN	intron	1710	4275	.	-	1	ID=blatn_trnK_1_intron_1;Parent=gene-blatn_trnK_1;gbkey=intron;gene=trnK
42	blatN	tRNA	4276	4312	.	-	1	ID=trna-blatn_trnK_1;gbkey=tRNA;gene=trnK
42	blatN	exon	4276	4312	.	-	1	ID=blatn_trnK_1_exon_1;Parent=gene-blatn_trnK_1;gbkey=exon;gene=trnK
42	blatX	gene	2030	3544	98.2	-	1	ID=gene-blatx_matK_1;gbkey=gene;gene=matK;gene_biotype=protein_coding
42	blatX	CDS	2030	3544	.	-	1	ID=cds-blatx_matK_1;gbkey=CDS;gene=matK
42	Chloe	gene	4974	6141	.	-	1	ID=gene-chloe_rps16_1_1;Parent=gene-chloe_rps16_1_1;gbkey=gene;gene=rps16
42	Chloe	CDS	4974	5200	.	-	1	ID=cds-chloe_rps16_1_2;Parent=gene-chloe_rps16_1_2;gbkey=CDS;gene=rps16
42	Chloe	exon	4974	5200	.	-	1	ID=chloe_rps16_1_5;Parent=gene-chloe_rps16_1_2;gbkey=exon;gene=rps16
42	Chloe	intron	5201	6101	.	-	1	ID=chloe_rps16_1_4;Parent=gene-chloe_rps16_1_2;gbkey=intron;gene=rps16
42	Chloe	CDS	6102	6141	.	-	1	ID=cds-chloe_rps16_1_2;Parent=gene-chloe_rps16_1_2;gbkey=CDS;gene=rps16
42	Chloe	exon	6102	6141	.	-	1	ID=chloe_rps16_1_3;Parent=gene-chloe_rps16_1_2;gbkey=exon;gene=rps16
42	blatN	gene	6572	6643	100	-	1	ID=gene-blatn_trnQ_1;gbkey=gene;gene=trnQ;gene_biotype=tRNA
42	blatN	tRNA	6572	6643	.	-	1	ID=trna-blatn_trnQ_1;gbkey=tRNA;gene=trnQ
42	blatX	gene	6973	7158	97.9	+	1	ID=gene-blatx_psbK_1;gbkey=gene;gene=psbK;gene_biotype=protein_coding
42	blatX	CDS	6973	7158	.	+	1	ID=cds-blatx_psbK_1;gbkey=CDS;gene=psbK
42	blatX	gene	7545	7655	100	+	1	ID=gene-blatx_psbI_1;gbkey=gene;gene=psbI;gene_biotype=protein_coding
42	blatX	CDS	7545	7655	.	+	1	ID=cds-blatx_psbI_1;gbkey=CDS;gene=psbI
42	blatN	gene	7747	7834	100	-	1	ID=gene-blatn_trnS_3;gbkey=gene;gene=trnS;gene_biotype=tRNA
42	blatN	tRNA	7747	7834	.	-	1	ID=trna-blatn_trnS_3;gbkey=tRNA;gene=trnS
42	blatN	gene	8485	9278	70.9	+	1	ID=gene-blatn_trnG_2;gbkey=gene;gene=trnG;gene_biotype=tRNA
42	blatN	tRNA	8485	8502	.	+	1	ID=trna-blatn_trnG_2;gbkey=tRNA;gene=trnG
42	blatN	exon	8485	8502	.	+	1	ID=blatn_trnG_2_exon_1;Parent=gene-blatn_trnG_2;gbkey=exon;gene=trnG
42	blatN	intron	8503	9224	.	+	1	ID=blatn_trnG_2_intron_1;Parent=gene-blatn_trnG_2;gbkey=intron;gene=trnG
42	blatN	tRNA	9225	9278	.	+	1	ID=trna-blatn_trnG_2;gbkey=tRNA;gene=trnG
42	blatN	exon	9225	9278	.	+	1	ID=blatn_trnG_2_exon_2;Parent=gene-blatn_trnG_2;gbkey=exon;gene=trnG
42	blatN	gene	9440	9511	100	+	1	ID=gene-blatn_trnR_3;gbkey=gene;gene=trnR;gene_biotype=tRNA
42	blatN	tRNA	9440	9511	.	+	1	ID=trna-blatn_trnR_3;gbkey=tRNA;gene=trnR
42	blatX	gene	9802	11325	99.1	-	1	ID=gene-blatx_atpA_1;gbkey=gene;gene=atpA;gene_biotype=protein_coding

42	blatX	CDS	9802	11325	.	-	1	ID=cds-blatx_atpA_1;gbkey=CDS;gene=atpA
42	blatX	gene	11391	12672	94.5	-	1	ID=gene-blatx_atpF_1;gbkey=gene;gene=atpF;gene_biotype=protein_coding
42	blatX	CDS	11391	11801	.	-	1	ID=cds-blatx_atpF_1;gbkey=CDS;gene=atpF
42	blatX	exon	11391	11801	.	-	1	ID=blatx_atpF_1_exon_2;Parent=gene-blatx_atpF_1;gbkey=exon;gene=atpF
42	blatX	intron	11802	12528	.	-	1	ID=blatx_atpF_1_intron_1;Parent=gene-blatx_atpF_1;gbkey=intron;gene=atpF
42	blatX	CDS	12529	12672	.	-	1	ID=cds-blatx_atpF_1;gbkey=CDS;gene=atpF
42	blatX	exon	12529	12672	.	-	1	ID=blatx_atpF_1_exon_1;Parent=gene-blatx_atpF_1;gbkey=exon;gene=atpF
42	blatX	gene	13137	13382	99.2	-	1	ID=gene-blatx_atpH_1;gbkey=gene;gene=atpH;gene_biotype=protein_coding
42	blatX	CDS	13137	13382	.	-	1	ID=cds-blatx_atpH_1;gbkey=CDS;gene=atpH
42	blatX	gene	13889	14638	99.4	-	1	ID=gene-blatx_atpI_1;gbkey=gene;gene=atpI;gene_biotype=protein_coding
42	blatX	CDS	13889	14638	.	-	1	ID=cds-blatx_atpI_1;gbkey=CDS;gene=atpI
42	blatX	gene	14879	15589	99.3	-	1	ID=gene-blatx_rps2_1;gbkey=gene;gene=rps2;gene_biotype=protein_coding
42	blatX	CDS	14879	15589	.	-	1	ID=cds-blatx_rps2_1;gbkey=CDS;gene=rps2
42	Chloe	CDS	15801	19943	.	-	1	ID=cds-chloe_rpoC2_1
42	Chloe	gene	15801	19943	.	-	1	ID=gene-chloe_rpoC2_1
42	blatX	gene	20125	22974	98.2	-	1	ID=gene-blatx_rpoC1_1;gbkey=gene;gene=rpoC1;gene_biotype=protein_coding
42	blatX	CDS	20125	21737	.	-	1	ID=cds-blatx_rpoC1_1;gbkey=CDS;gene=rpoC1
42	blatX	exon	20125	21737	.	-	1	ID=blatx_rpoC1_1_exon_2;Parent=gene-blatx_rpoC1_1;gbkey=exon;gene=rpoC1
42	blatX	intron	21738	22544	.	-	1	ID=blatx_rpoC1_1_intron_1;Parent=gene-blatx_rpoC1_1;gbkey=intron;gene=rpoC1
42	blatX	CDS	22545	22974	.	-	1	ID=cds-blatx_rpoC1_1;gbkey=CDS;gene=rpoC1
42	blatX	exon	22545	22974	.	-	1	ID=blatx_rpoC1_1_exon_1;Parent=gene-blatx_rpoC1_1;gbkey=exon;gene=rpoC1
42	blatX	gene	23001	26219	99.1	-	1	ID=gene-blatx_rpoB_1;gbkey=gene;gene=rpoB;gene_biotype=protein_coding
42	blatX	CDS	23001	26219	.	-	1	ID=cds-blatx_rpoB_1;gbkey=CDS;gene=rpoB
42	blatN	gene	27319	27389	100	+	1	ID=gene-blatn_trnC_1;gbkey=gene;gene=trnC;gene_biotype=tRNA
42	blatN	tRNA	27319	27389	.	+	1	ID=trna-blatn_trnC_1;gbkey=tRNA;gene=trnC
42	blatX	gene	28033	28122	98.9	+	1	ID=gene-blatx_petN_1;gbkey=gene;gene=petN;gene_biotype=protein_coding
42	blatX	CDS	28033	28122	.	+	1	ID=cds-blatx_petN_1;gbkey=CDS;gene=petN
42	blatX	gene	28654	28758	99.1	-	1	ID=gene-blatx_psbM_1;gbkey=gene;gene=psbM;gene_biotype=protein_coding
42	blatX	CDS	28654	28758	.	-	1	ID=cds-blatx_psbM_1;gbkey=CDS;gene=psbM
42	blatN	gene	29749	29822	100	-	1	ID=gene-blatn_trnD_1;gbkey=gene;gene=trnD;gene_biotype=tRNA
42	blatN	tRNA	29749	29822	.	-	1	ID=trna-blatn_trnD_1;gbkey=tRNA;gene=trnD
42	blatN	gene	30262	30345	100	-	1	ID=gene-blatn_trnY_1;gbkey=gene;gene=trnY;gene_biotype=tRNA
42	blatN	tRNA	30262	30345	.	-	1	ID=trna-blatn_trnY_1;gbkey=tRNA;gene=trnY
42	blatN	gene	30405	30477	100	-	1	ID=gene-blatn_trnE_1;gbkey=gene;gene=trnE;gene_biotype=tRNA
42	blatN	tRNA	30405	30477	.	-	1	ID=trna-blatn_trnE_1;gbkey=tRNA;gene=trnE
42	blatN	gene	31086	31157	100	+	1	ID=gene-blatn_trnT_2;gbkey=gene;gene=trnT;gene_biotype=tRNA
42	blatN	tRNA	31086	31157	.	+	1	ID=trna-blatn_trnT_2;gbkey=tRNA;gene=trnT
42	blatX	gene	32428	33489	99.6	+	1	ID=gene-blatx_psbD_1;gbkey=gene;gene=psbD;gene_biotype=protein_coding
42	blatX	CDS	32428	33489	.	+	1	ID=cds-blatx_psbD_1;gbkey=CDS;gene=psbD
42	blatX	gene	33437	34858	99.6	+	1	ID=gene-blatx_psbC_1;gbkey=gene;gene=psbC;gene_biotype=protein_coding
42	blatX	CDS	33437	34858	.	+	1	ID=cds-blatx_psbC_1;gbkey=CDS;gene=psbC
42	blatN	gene	35040	35131	100	-	1	ID=gene-blatn_trnS_2;gbkey=gene;gene=trnS;gene_biotype=tRNA
42	blatN	tRNA	35040	35131	.	-	1	ID=trna-blatn_trnS_2;gbkey=tRNA;gene=trnS

42	blatX	gene	35479	35667	99.5	+	1	ID=gene-blatx_psbZ_1;gbkey=gene;gene=psbZ;gene_biotype=protein_coding
42	blatX	CDS	35479	35667	.	+	1	ID=cds-blatx_psbZ_1;gbkey=CDS;gene=psbZ
42	blatN	gene	36159	36229	100	+	1	ID=gene-blatn_trnG_1;gbkey=gene;gene=trnG;gene_biotype=tRNA
42	blatN	tRNA	36159	36229	.	+	1	ID=trna-blatn_trnG_1;gbkey=tRNA;gene=trnG
42	blatN	gene	36374	36447	100	-	1	ID=gene-blatn_trnfM_1;gbkey=gene;gene=trnfM;gene_biotype=tRNA
42	blatN	tRNA	36374	36447	.	-	1	ID=trna-blatn_trnfM_1;gbkey=tRNA;gene=trnfM
42	blatX	gene	36608	36910	99.7	-	1	ID=gene-blatx_rps14_1;gbkey=gene;gene=rps14;gene_biotype=protein_coding
42	blatX	CDS	36608	36910	.	-	1	ID=cds-blatx_rps14_1;gbkey=CDS;gene=rps14
42	blatX	gene	37045	39249	99.6	-	1	ID=gene-blatx_psaB_1;gbkey=gene;gene=psaB;gene_biotype=protein_coding
42	blatX	CDS	37045	39249	.	-	1	ID=cds-blatx_psaB_1;gbkey=CDS;gene=psaB
42	blatX	gene	39275	41527	99.7	-	1	ID=gene-blatx_psaA_1;gbkey=gene;gene=psaA;gene_biotype=protein_coding
42	blatX	CDS	39275	41527	.	-	1	ID=cds-blatx_psaA_1;gbkey=CDS;gene=psaA
42	blatX	gene	42233	44232	94.1	-	1	ID=gene-ycf3;Name=ycf3;gbkey=Gene;gene=ycf3;gene_biotype=protein_coding
42	blatX	CDS	42233	42387	.	-	1	ID=cds-ycf3;gbkey=CDS;gene=ycf3
42	blatX	exon	42233	42387	.	-	1	ID=blatx_ycf3_exon_3;Parent=gene-ycf3;gbkey=exon;gene=ycf3
42	blatX	intron	42388	43160	.	-	1	ID=blatx_ycf3_intron_2;Parent=gene-ycf3;gbkey=intron;gene=ycf3
42	blatX	CDS	43161	43386	.	-	1	ID=cds-ycf3;gbkey=CDS;gene=ycf3
42	blatX	exon	43161	43386	.	-	1	ID=blatx_ycf3_exon_2;Parent=gene-ycf3;gbkey=exon;gene=ycf3
42	blatX	intron	43387	44106	.	-	1	ID=blatx_ycf3_intron_1;Parent=gene-ycf3;gbkey=intron;gene=ycf3
42	blatX	CDS	44107	44232	.	-	1	ID=cds-ycf3;gbkey=CDS;gene=ycf3
42	blatX	exon	44107	44232	.	-	1	ID=blatx_ycf3_exon_1;Parent=gene-ycf3;gbkey=exon;gene=ycf3
42	blatN	gene	44468	44554	100	+	1	ID=gene-blatn_trnS_1;gbkey=gene;gene=trnS;gene_biotype=tRNA
42	blatN	tRNA	44468	44554	.	+	1	ID=trna-blatn_trnS_1;gbkey=tRNA;gene=trnS
42	blatX	gene	44865	45470	99.4	-	1	ID=gene-blatx_rps4_1;gbkey=gene;gene=rps4;gene_biotype=protein_coding
42	blatX	CDS	44865	45470	.	-	1	ID=cds-blatx_rps4_1;gbkey=CDS;gene=rps4
42	blatN	gene	45873	45945	100	-	1	ID=gene-blatn_trnT_1;gbkey=gene;gene=trnT;gene_biotype=tRNA
42	blatN	tRNA	45873	45945	.	-	1	ID=trna-blatn_trnT_1;gbkey=tRNA;gene=trnT
42	blatN	gene	46513	47101	76.5	+	1	ID=gene-blatn_trnL_4;gbkey=gene;gene=trnL;gene_biotype=tRNA
42	blatN	tRNA	46513	46547	.	+	1	ID=trna-blatn_trnL_4;gbkey=tRNA;gene=trnL
42	blatN	exon	46513	46547	.	+	1	ID=blatn_trnL_4_exon_1;Parent=gene-blatn_trnL_4;gbkey=exon;gene=trnL
42	blatN	intron	46548	47051	.	+	1	ID=blatn_trnL_4_intron_1;Parent=gene-blatn_trnL_4;gbkey=intron;gene=trnL
42	blatN	tRNA	47052	47101	.	+	1	ID=trna-blatn_trnL_4;gbkey=tRNA;gene=trnL
42	blatN	exon	47052	47101	.	+	1	ID=blatn_trnL_4_exon_2;Parent=gene-blatn_trnL_4;gbkey=exon;gene=trnL
42	blatN	gene	47584	47656	100	+	1	ID=gene-blatn_trnF_1;gbkey=gene;gene=trnF;gene_biotype=tRNA
42	blatN	tRNA	47584	47656	.	+	1	ID=trna-blatn_trnF_1;gbkey=tRNA;gene=trnF
42	blatX	gene	48247	48723	99.6	-	1	ID=gene-blatx_ndhJ_1;gbkey=gene;gene=ndhJ;gene_biotype=protein_coding
42	blatX	CDS	48247	48723	.	-	1	ID=cds-blatx_ndhJ_1;gbkey=CDS;gene=ndhJ
42	blatX	gene	48826	49503	99.2	-	1	ID=gene-blatx_ndhK_1;gbkey=gene;gene=ndhK;gene_biotype=protein_coding
42	blatX	CDS	48826	49503	.	-	1	ID=cds-blatx_ndhK_1;gbkey=CDS;gene=ndhK
42	blatX	gene	49567	49929	99.5	-	1	ID=gene-blatx_ndhC_1;gbkey=gene;gene=ndhC;gene_biotype=protein_coding
42	blatX	CDS	49567	49929	.	-	1	ID=cds-blatx_ndhC_1;gbkey=CDS;gene=ndhC
42	blatN	gene	50762	51426	73	-	1	ID=gene-blatn_trnV_3;gbkey=gene;gene=trnV;gene_biotype=tRNA
42	blatN	tRNA	50762	50798	.	-	1	ID=trna-blatn_trnV_3;gbkey=tRNA;gene=trnV

42	blatN	exon	50762	50798	.	-	1	ID=blatn_trnV_3_exon_2;Parent=gene-blatn_trnV_3;gbkey=exon;gene=trnV
42	blatN	intron	50799	51389	.	-	1	ID=blatn_trnV_3_intron_1;Parent=gene-blatn_trnV_3;gbkey=intron;gene=trnV
42	blatN	tRNA	51390	51426	.	-	1	ID=trna-blatn_trnV_3;gbkey=tRNA;gene=trnV
42	blatN	exon	51390	51426	.	-	1	ID=blatn_trnV_3_exon_1;Parent=gene-blatn_trnV_3;gbkey=exon;gene=trnV
42	blatN	gene	51611	51683	100	+	1	ID=gene-blatn_trnM_1;gbkey=gene;gene=trnM;gene_biotype=tRNA
42	blatN	tRNA	51611	51683	.	+	1	ID=trna-blatn_trnM_1;gbkey=tRNA;gene=trnM
42	blatX	gene	51824	52222	99	-	1	ID=gene-blatx_atpE_1;gbkey=gene;gene=atpE;gene_biotype=protein_coding
42	blatX	CDS	51824	52222	.	-	1	ID=cds-blatx_atpE_1;gbkey=CDS;gene=atpE
42	blatX	gene	52219	53715	98.6	-	1	ID=gene-blatx_atpB_1;gbkey=gene;gene=atpB;gene_biotype=protein_coding
42	blatX	CDS	52219	53715	.	-	1	ID=cds-blatx_atpB_1;gbkey=CDS;gene=atpB
42	blatX	gene	54504	55943	98.8	+	1	ID=gene-blatx_rbcL_1;gbkey=gene;gene=rbcL;gene_biotype=protein_coding
42	blatX	CDS	54504	55943	.	+	1	ID=cds-blatx_rbcL_1;gbkey=CDS;gene=rbcL
42	blatX	gene	56636	58090	98.6	+	1	ID=gene-blatx_accD_1;gbkey=gene;gene=accD;gene_biotype=protein_coding
42	blatX	CDS	56636	58090	.	+	1	ID=cds-blatx_accD_1;gbkey=CDS;gene=accD
42	blatX	gene	58769	58882	100	+	1	ID=gene-blatx_psaI_1;gbkey=gene;gene=psaI;gene_biotype=protein_coding
42	blatX	CDS	58769	58882	.	+	1	ID=cds-blatx_psaI_1;gbkey=CDS;gene=psaI
42	blatX	gene	59290	59844	99.9	+	1	ID=gene-ycf4;Name=ycf4;gbkey=Gene;gene=ycf4;gene_biotype=protein_coding
42	blatX	CDS	59290	59844	.	+	1	ID=cds-blatx_ycf4;gbkey=CDS;gene=ycf4
42	blatX	gene	60267	60956	99.2	+	1	ID=gene-blatx_cemA_1;gbkey=gene;gene=cemA;gene_biotype=protein_coding
42	blatX	CDS	60267	60956	.	+	1	ID=cds-blatx_cemA_1;gbkey=CDS;gene=cemA
42	blatX	gene	61185	62147	98.5	+	1	ID=gene-blatx_petA_1;gbkey=gene;gene=petA;gene_biotype=protein_coding
42	blatX	CDS	61185	62147	.	+	1	ID=cds-blatx_petA_1;gbkey=CDS;gene=petA
42	blatX	gene	63140	63262	99.2	-	1	ID=gene-blatx_psbJ_1;gbkey=gene;gene=psbJ;gene_biotype=protein_coding
42	blatX	CDS	63140	63262	.	-	1	ID=cds-blatx_psbJ_1;gbkey=CDS;gene=psbJ
42	blatX	gene	63406	63522	100	-	1	ID=gene-blatx_psbL_1;gbkey=gene;gene=psbL;gene_biotype=protein_coding
42	blatX	CDS	63406	63522	.	-	1	ID=cds-blatx_psbL_1;gbkey=CDS;gene=psbL
42	blatX	gene	63544	63663	100	-	1	ID=gene-blatx_psbF_1;gbkey=gene;gene=psbF;gene_biotype=protein_coding
42	blatX	CDS	63544	63663	.	-	1	ID=cds-blatx_psbF_1;gbkey=CDS;gene=psbF
42	blatX	gene	63673	63924	99.3	-	1	ID=gene-blatx_psbE_1;gbkey=gene;gene=psbE;gene_biotype=protein_coding
42	blatX	CDS	63673	63924	.	-	1	ID=cds-blatx_psbE_1;gbkey=CDS;gene=psbE
42	blatX	gene	65297	65392	100	+	1	ID=gene-blatx_petL_1;gbkey=gene;gene=petL;gene_biotype=protein_coding
42	blatX	CDS	65297	65392	.	+	1	ID=cds-blatx_petL_1;gbkey=CDS;gene=petL
42	blatX	gene	65580	65693	100	+	1	ID=gene-blatx_petG_1;gbkey=gene;gene=petG;gene_biotype=protein_coding
42	blatX	CDS	65580	65693	.	+	1	ID=cds-blatx_petG_1;gbkey=CDS;gene=petG
42	blatN	gene	65823	65896	100	-	1	ID=gene-blatn_trnW_1;gbkey=gene;gene=trnW;gene_biotype=tRNA
42	blatN	tRNA	65823	65896	.	-	1	ID=trna-blatn_trnW_1;gbkey=tRNA;gene=trnW
42	blatN	gene	66080	66153	100	-	1	ID=gene-blatn_trnP_1;gbkey=gene;gene=trnP;gene_biotype=tRNA
42	blatN	tRNA	66080	66153	.	-	1	ID=trna-blatn_trnP_1;gbkey=tRNA;gene=trnP
42	blatX	gene	66532	66660	99.3	+	1	ID=gene-blatx_psaJ_1;gbkey=gene;gene=psaJ;gene_biotype=protein_coding
42	blatX	CDS	66532	66660	.	+	1	ID=cds-blatx_psaJ_1;gbkey=CDS;gene=psaJ
42	blatX	gene	67098	67298	99.1	+	1	ID=gene-blatx_rpl33_1;gbkey=gene;gene=rpl33;gene_biotype=protein_coding
42	blatX	CDS	67098	67298	.	+	1	ID=cds-blatx_rpl33_1;gbkey=CDS;gene=rpl33
42	blatX	gene	67513	67818	99.4	+	1	ID=gene-blatx_rps18_1;gbkey=gene;gene=rps18;gene_biotype=protein_coding

42	blatX	CDS	67513	67818	.	+	1	ID=cds-blatx_rps18_1;gbkey=CDS;gene=rps18
42	blatX	gene	68115	68468	98.4	-	1	ID=gene-blatx_rpl20_1;gbkey=gene;gene=rpl20;gene_biotype=protein_coding
42	blatX	CDS	68115	68468	.	-	1	ID=cds-blatx_rpl20_1;gbkey=CDS;gene=rpl20
42	blatX	gene	69224	69337	100	-	1	ID=gene-trsp_rps12_1;gbkey=gene;gene=rps12;gene_biotype=protein_coding
42	blatX	CDS	69224	69337	.	-	1	ID=cds-trsp_rps12_1;gbkey=CDS;gene=rps12
42	blatX	gene	69224	69337	100	-	1	ID=gene-trsp_rps12_1;gbkey=gene;gene=rps12;gene_biotype=protein_coding
42	blatX	CDS	69224	69337	.	-	1	ID=cds-trsp_rps12_1;gbkey=CDS;gene=rps12
42	blatX	exon	69224	69337	.	-	1	ID=trsp_rps12_1_exon_1;Parent=gene-trsp_rps12_1;gbkey=exon;gene=rps12
42	blatX	gene	69517	71549	95	-	1	ID=gene-blatx_clpP1_1;gbkey=gene;gene=clpP1;gene_biotype=protein_coding
42	blatX	CDS	69517	69744	.	-	1	ID=cds-blatx_clpP1_1;gbkey=CDS;gene=clpP1
42	blatX	exon	69517	69744	.	-	1	ID=blatx_clpP1_1_exon_3;Parent=gene-blatx_clpP1_1;gbkey=exon;gene=clpP1
42	blatX	intron	69745	70314	.	-	1	ID=blatx_clpP1_1_intron_2;Parent=gene-blatx_clpP1_1;gbkey=intron;gene=clpP1
42	blatX	CDS	70315	70606	.	-	1	ID=cds-blatx_clpP1_1;gbkey=CDS;gene=clpP1
42	blatX	exon	70315	70606	.	-	1	ID=blatx_clpP1_1_exon_2;Parent=gene-blatx_clpP1_1;gbkey=exon;gene=clpP1
42	blatX	intron	70607	71478	.	-	1	ID=blatx_clpP1_1_intron_1;Parent=gene-blatx_clpP1_1;gbkey=intron;gene=clpP1
42	blatX	CDS	71479	71549	.	-	1	ID=cds-blatx_clpP1_1;gbkey=CDS;gene=clpP1
42	blatX	exon	71479	71549	.	-	1	ID=blatx_clpP1_1_exon_1;Parent=gene-blatx_clpP1_1;gbkey=exon;gene=clpP1
42	blatX	gene	72030	73556	98.5	+	1	ID=gene-blatx_psbB_1;gbkey=gene;gene=psbB;gene_biotype=protein_coding
42	blatX	CDS	72030	73556	.	+	1	ID=cds-blatx_psbB_1;gbkey=CDS;gene=psbB
42	blatX	gene	73739	73840	100	+	1	ID=gene-blatx_psbT_1;gbkey=gene;gene=psbT;gene_biotype=protein_coding
42	blatX	CDS	73739	73840	.	+	1	ID=cds-blatx_psbT_1;gbkey=CDS;gene=psbT
42	blatX	gene	73906	74037	100	-	1	ID=gene-psbN;Name=psbN;gbkey=Gene;gene=psbN;gene_biotype=protein_coding
42	blatX	CDS	73906	74037	.	-	1	ID=cds-blatx_psbN;gbkey=CDS;gene=psbN
42	blatX	gene	74142	74363	99.1	+	1	ID=gene-blatx_psbH_1;gbkey=gene;gene=psbH;gene_biotype=protein_coding
42	blatX	CDS	74142	74363	.	+	1	ID=cds-blatx_psbH_1;gbkey=CDS;gene=psbH
42	Chloe	gene	74498	75937	.	+	1	ID=gene-chloe_petB_1_1
42	Chloe	CDS	74498	74503	.	+	1	ID=cds-chloe_petB_1_2
42	Chloe	exon	74498	74503	.	+	1	ID=chloe_petB_1_3
42	Chloe	intron	74504	75295	.	+	1	ID=chloe_petB_1_4
42	Chloe	CDS	75296	75937	.	+	1	ID=cds-chloe_petB_1_2
42	Chloe	exon	75296	75937	.	+	1	ID=chloe_petB_1_5
42	Chloe	gene	76126	77331	.	+	1	ID=gene-chloe_petD_1_1
42	Chloe	CDS	76126	76133	.	+	1	ID=cds-chloe_petD_1_2
42	Chloe	exon	76126	76133	.	+	1	ID=chloe_petD_1_3
42	Chloe	intron	76134	76856	.	+	1	ID=chloe_petD_1_4
42	Chloe	CDS	76857	77331	.	+	1	ID=cds-chloe_petD_1_2
42	Chloe	exon	76857	77331	.	+	1	ID=chloe_petD_1_5
42	blatX	gene	77537	78520	98.5	-	1	ID=gene-blatx_rpoA_1;gbkey=gene;gene=rpoA;gene_biotype=protein_coding
42	blatX	CDS	77537	78520	.	-	1	ID=cds-blatx_rpoA_1;gbkey=CDS;gene=rpoA
42	blatX	gene	78590	79006	99.1	-	1	ID=gene-blatx_rps11_1;gbkey=gene;gene=rps11;gene_biotype=protein_coding
42	blatX	CDS	78590	79006	.	-	1	ID=cds-blatx_rps11_1;gbkey=CDS;gene=rps11
42	blatX	gene	79121	79234	100	-	1	ID=gene-blatx_rpl36_1;gbkey=gene;gene=rpl36;gene_biotype=protein_coding
42	blatX	CDS	79121	79234	.	-	1	ID=cds-blatx_rpl36_1;gbkey=CDS;gene=rpl36

42	blatX	gene	79699	80103	97.8	-	1	ID=gene-blatx_rps8_1;gbkey=gene;gene=rps8;gene_biotype=protein_coding
42	blatX	CDS	79699	80103	.	-	1	ID=cds-blatx_rps8_1;gbkey=CDS;gene=rps8
42	blatX	gene	80316	80684	99.5	-	1	ID=gene-blatx_rpl14_1;gbkey=gene;gene=rpl14;gene_biotype=protein_coding
42	blatX	CDS	80316	80684	.	-	1	ID=cds-blatx_rpl14_1;gbkey=CDS;gene=rpl14
42	Chloe	gene	80809	82278	.	-	1	ID=gene-chloe_rpl16_1_1
42	Chloe	CDS	80809	81207	.	-	1	ID=cds-chloe_rpl16_1_2
42	Chloe	exon	80809	81207	.	-	1	ID=chloe_rpl16_1_5
42	Chloe	intron	81208	82269	.	-	1	ID=chloe_rpl16_1_4
42	Chloe	CDS	82270	82278	.	-	1	ID=cds-chloe_rpl16_1_2
42	Chloe	exon	82270	82278	.	-	1	ID=chloe_rpl16_1_3
42	blatX	gene	82462	83118	99.4	-	1	ID=gene-blatx_rps3_1;gbkey=gene;gene=rps3;gene_biotype=protein_coding
42	blatX	CDS	82462	83118	.	-	1	ID=cds-blatx_rps3_1;gbkey=CDS;gene=rps3
42	blatX	gene	83103	83585	98.4	-	1	ID=gene-blatx_rpl22_1;gbkey=gene;gene=rpl22;gene_biotype=protein_coding
42	blatX	CDS	83103	83585	.	-	1	ID=cds-blatx_rpl22_1;gbkey=CDS;gene=rpl22
42	blatX	gene	83641	83919	98.3	-	1	ID=gene-blatx_rps19_1;gbkey=gene;gene=rps19;gene_biotype=protein_coding
42	blatX	CDS	83641	83919	.	-	1	ID=cds-blatx_rps19_1;gbkey=CDS;gene=rps19
42	OGDRAW	inverted_repeat	83806	110273	.	+	.	ID=repeat_region-ogdraw_repreg2;gbkey=repeat_region
42	blatX	gene	83974	85480	97.1	-	1	ID=gene-blatx_rpl2_2;gbkey=gene;gene=rpl2;gene_biotype=protein_coding
42	blatX	CDS	83974	84407	.	-	1	ID=cds-blatx_rpl2_2;gbkey=CDS;gene=rpl2
42	blatX	exon	83974	84407	.	-	1	ID=blatx_rpl2_2_exon_2;Parent=gene-blatx_rpl2_2;gbkey=exon;gene=rpl2
42	blatX	intron	84408	85089	.	-	1	ID=blatx_rpl2_2_intron_1;Parent=gene-blatx_rpl2_2;gbkey=intron;gene=rpl2
42	blatX	CDS	85090	85480	.	-	1	ID=cds-blatx_rpl2_2;gbkey=CDS;gene=rpl2
42	blatX	exon	85090	85480	.	-	1	ID=blatx_rpl2_2_exon_1;Parent=gene-blatx_rpl2_2;gbkey=exon;gene=rpl2
42	blatX	gene	85499	85780	100	-	1	ID=gene-blatx_rpl23_2;gbkey=gene;gene=rpl23;gene_biotype=protein_coding
42	blatX	CDS	85499	85780	.	-	1	ID=cds-blatx_rpl23_2;gbkey=CDS;gene=rpl23
42	blatN	gene	85949	86022	100	-	1	ID=gene-blatn_trnI_2;gbkey=gene;gene=trnI;gene_biotype=tRNA
42	blatN	tRNA	85949	86022	.	-	1	ID=trna-blatn_trnI_2;gbkey=tRNA;gene=trnI
42	blatX	gene	86111	92983	99.7	+	1	ID=gene-blatx_ycf2_2;gbkey=gene;gene=ycf2;gene_biotype=protein_coding
42	blatX	CDS	86111	92983	.	+	1	ID=cds-blatx_ycf2_2;gbkey=CDS;gene=ycf2
42	blatN	gene	93901	93981	100	-	1	ID=gene-blatn_trnL_3;gbkey=gene;gene=trnL;gene_biotype=tRNA
42	blatN	tRNA	93901	93981	.	-	1	ID=trna-blatn_trnL_3;gbkey=tRNA;gene=trnL
42	blatX	gene	94562	96785	98.5	-	1	ID=gene-blatx_ndhB_2;gbkey=gene;gene=ndhB;gene_biotype=protein_coding
42	blatX	CDS	94562	95323	.	-	1	ID=cds-blatx_ndhB_2;gbkey=CDS;gene=ndhB
42	blatX	exon	94562	95323	.	-	1	ID=blatx_ndhB_2_exon_2;Parent=gene-blatx_ndhB_2;gbkey=exon;gene=ndhB
42	blatX	intron	95324	96008	.	-	1	ID=blatx_ndhB_2_intron_1;Parent=gene-blatx_ndhB_2;gbkey=intron;gene=ndhB
42	blatX	CDS	96009	96785	.	-	1	ID=cds-blatx_ndhB_2;gbkey=CDS;gene=ndhB
42	blatX	exon	96009	96785	.	-	1	ID=blatx_ndhB_2_exon_1;Parent=gene-blatx_ndhB_2;gbkey=exon;gene=ndhB
42	blatX	gene	97099	97566	100	-	1	ID=gene-blatx_rps7_2;gbkey=gene;gene=rps7;gene_biotype=protein_coding
42	blatX	CDS	97099	97566	.	-	1	ID=cds-blatx_rps7_2;gbkey=CDS;gene=rps7
42	blatX	gene	97621	98414	100	-	1	ID=gene-trsp_rps12_1;gbkey=gene;gene=rps12;gene_biotype=protein_coding
42	blatX	CDS	97621	97645	.	-	1	ID=cds-trsp_rps12_1;gbkey=CDS;gene=rps12
42	blatX	exon	97621	97645	.	-	1	ID=trsp_rps12_1_exon_3;Parent=gene-trsp_rps12_1;gbkey=exon;gene=rps12
42	blatX	intron	97646	98182	.	-	1	ID=blatx_rps12_2_intron_1;Parent=gene-blatx_rps12_2;gbkey=intron;gene=rps12

42	blatX	CDS	98183	98414	.	-	1	ID=cds-trsp_rps12_1;gbkey=CDS;gene=rps12
42	blatX	exon	98183	98414	.	-	1	ID=trsp_rps12_1_exon_2;Parent=gene-trsp_rps12_1;gbkey=exon;gene=rps12
42	blatN	gene	100337	100408	100	+	1	ID=gene-blatn_trnV_2;gbkey=gene;gene=trnV;gene_biotype=tRNA
42	blatN	tRNA	100337	100408	.	+	1	ID=trna-blatn_trnV_2;gbkey=tRNA;gene=trnV
42	blatN	gene	100640	102130	100	+	1	ID=gene-blatn_rrn16S_2;gbkey=gene;gene=rrn16S;gene_biotype=rRNA
42	blatN	rRNA	100640	102130	.	+	1	ID=rrna-blatn_rrn16S_2;gbkey=rRNA;gene=rrn16S
42	blatN	gene	102429	103447	69.5	+	1	ID=gene-blatn_trnI_4;gbkey=gene;gene=trnI;gene_biotype=tRNA
42	blatN	tRNA	102429	102465	.	+	1	ID=trna-blatn_trnI_4;gbkey=tRNA;gene=trnI
42	blatN	exon	102429	102465	.	+	1	ID=blatn_trnI_4_exon_1;Parent=gene-blatn_trnI_4;gbkey=exon;gene=trnI
42	blatN	intron	102466	103412	.	+	1	ID=blatn_trnI_4_intron_1;Parent=gene-blatn_trnI_4;gbkey=intron;gene=trnI
42	blatN	tRNA	103413	103447	.	+	1	ID=trna-blatn_trnI_4;gbkey=tRNA;gene=trnI
42	blatN	exon	103413	103447	.	+	1	ID=blatn_trnI_4_exon_2;Parent=gene-blatn_trnI_4;gbkey=exon;gene=trnI
42	blatN	gene	103512	104385	71.3	+	1	ID=gene-blatn_trnA_2;gbkey=gene;gene=trnA;gene_biotype=tRNA
42	blatN	tRNA	103512	103549	.	+	1	ID=trna-blatn_trnA_2;gbkey=tRNA;gene=trnA
42	blatN	exon	103512	103549	.	+	1	ID=blatn_trnA_2_exon_1;Parent=gene-blatn_trnA_2;gbkey=exon;gene=trnA
42	blatN	intron	103550	104350	.	+	1	ID=blatn_trnA_2_intron_1;Parent=gene-blatn_trnA_2;gbkey=intron;gene=trnA
42	blatN	tRNA	104351	104385	.	+	1	ID=trna-blatn_trnA_2;gbkey=tRNA;gene=trnA
42	blatN	exon	104351	104385	.	+	1	ID=blatn_trnA_2_exon_2;Parent=gene-blatn_trnA_2;gbkey=exon;gene=trnA
42	blatN	gene	104538	107347	100	+	1	ID=gene-blatn_rrn23S_2;gbkey=gene;gene=rrn23S;gene_biotype=rRNA
42	blatN	rRNA	104538	107347	.	+	1	ID=rrna-blatn_rrn23S_2;gbkey=rRNA;gene=rrn23S
42	blatN	gene	107446	107548	100	+	1	ID=gene-blatn_rrn4.5S_2;gbkey=gene;gene=rrn4.5S;gene_biotype=rRNA
42	blatN	rRNA	107446	107548	.	+	1	ID=rrna-blatn_rrn4.5S_2;gbkey=rRNA;gene=rrn4.5S
42	blatN	gene	107796	107916	100	+	1	ID=gene-blatn_rrn5S_2;gbkey=gene;gene=rrn5;gene_biotype=rRNA
42	blatN	rRNA	107796	107916	.	+	1	ID=rrna-blatn_rrn5S_2;gbkey=rRNA;gene=rrn5
42	blatN	gene	108156	108229	100	+	1	ID=gene-blatn_trnR_2;gbkey=gene;gene=trnR;gene_biotype=tRNA
42	blatN	tRNA	108156	108229	.	+	1	ID=trna-blatn_trnR_2;gbkey=tRNA;gene=trnR
42	blatN	gene	108857	108928	100	-	1	ID=gene-blatn_trnN_2;gbkey=gene;gene=trnN;gene_biotype=tRNA
42	blatN	tRNA	108857	108928	.	-	1	ID=trna-blatn_trnN_2;gbkey=tRNA;gene=trnN
42	blatX	gene	109249	110274	99.6	+	1	ID=gene-blatx_ycf1_1;gbkey=gene;gene=ycf1;gene_biotype=protein_coding
42	blatX	CDS	109249	110274	.	+	1	ID=cds-blatx_ycf1_1;gbkey=CDS;gene=ycf1
42	blatX	gene	110236	112476	98.6	-	1	ID=gene-blatx_ndhF_1;gbkey=gene;gene=ndhF;gene_biotype=protein_coding
42	blatX	CDS	110236	112476	.	-	1	ID=cds-blatx_ndhF_1;gbkey=CDS;gene=ndhF
42	OGDRAW	sequence_feature	110274	128152	.	+	1	ID=misc_feature-ogdraw_ssc;gbkey=misc_feature
42	blatX	gene	113320	113478	98.8	+	1	ID=gene-blatx_rpl32_1;gbkey=gene;gene=rpl32;gene_biotype=protein_coding
42	blatX	CDS	113320	113478	.	+	1	ID=cds-blatx_rpl32_1;gbkey=CDS;gene=rpl32
42	blatN	gene	114205	114284	100	+	1	ID=gene-blatn_trnL_2;gbkey=gene;gene=trnL;gene_biotype=tRNA
42	blatN	tRNA	114205	114284	.	+	1	ID=trna-blatn_trnL_2;gbkey=tRNA;gene=trnL
42	blatX	gene	114399	115385	98.6	+	1	ID=gene-blatx_ccsA_1;gbkey=gene;gene=ccsA;gene_biotype=protein_coding
42	blatX	CDS	114399	115385	.	+	1	ID=cds-blatx_ccsA_1;gbkey=CDS;gene=ccsA
42	blatX	gene	115599	117101	98.6	-	1	ID=gene-blatx_ndhD_1;gbkey=gene;gene=ndhD;gene_biotype=protein_coding
42	blatX	CDS	115599	117101	.	-	1	ID=cds-blatx_ndhD_1;gbkey=CDS;gene=ndhD
42	blatX	gene	117261	117506	99.6	-	1	ID=gene-blatx_psaC_1;gbkey=gene;gene=psaC;gene_biotype=protein_coding
42	blatX	CDS	117261	117506	.	-	1	ID=cds-blatx_psaC_1;gbkey=CDS;gene=psaC

42	blatX	gene	117747	118052	99.1	-	1	ID=gene-blatx_ndhE_1;gbkey=gene;gene=ndhE;gene_biotype=protein_coding
42	blatX	CDS	117747	118052	.	-	1	ID=cds-blatx_ndhE_1;gbkey=CDS;gene=ndhE
42	blatX	gene	118302	118832	99.1	-	1	ID=gene-blatx_ndhG_1;gbkey=gene;gene=ndhG;gene_biotype=protein_coding
42	blatX	CDS	118302	118832	.	-	1	ID=cds-blatx_ndhG_1;gbkey=CDS;gene=ndhG
42	blatX	gene	119150	119668	99.7	-	1	ID=gene-blatx_ndhI_1;gbkey=gene;gene=ndhI;gene_biotype=protein_coding
42	blatX	CDS	119150	119668	.	-	1	ID=cds-blatx_ndhI_1;gbkey=CDS;gene=ndhI
42	blatX	gene	119754	121912	96.1	-	1	ID=gene-blatx_ndhA_1;gbkey=gene;gene=ndhA;gene_biotype=protein_coding
42	blatX	CDS	119754	120284	.	-	1	ID=cds-blatx_ndhA_1;gbkey=CDS;gene=ndhA
42	blatX	exon	119754	120284	.	-	1	ID=blatx_ndhA_1_exon_2;Parent=gene-blatx_ndhA_1;gbkey=exon;gene=ndhA
42	blatX	intron	120285	121360	.	-	1	ID=blatx_ndhA_1_intron_1;Parent=gene-blatx_ndhA_1;gbkey=intron;gene=ndhA
42	blatX	CDS	121361	121912	.	-	1	ID=cds-blatx_ndhA_1;gbkey=CDS;gene=ndhA
42	blatX	exon	121361	121912	.	-	1	ID=blatx_ndhA_1_exon_1;Parent=gene-blatx_ndhA_1;gbkey=exon;gene=ndhA
42	blatX	gene	121914	123095	99.4	-	1	ID=gene-blatx_ndhH_1;gbkey=gene;gene=ndhH;gene_biotype=protein_coding
42	blatX	CDS	121914	123095	.	-	1	ID=cds-blatx_ndhH_1;gbkey=CDS;gene=ndhH
42	blatX	gene	123199	123465	98.6	-	1	ID=gene-blatx_rps15_1;gbkey=gene;gene=rps15;gene_biotype=protein_coding
42	blatX	CDS	123199	123465	.	-	1	ID=cds-blatx_rps15_1;gbkey=CDS;gene=rps15
42	Chloe	CDS	123808	129174	.	-	1	ID=cds-chloe_ycf1_1
42	Chloe	gene	123808	129174	.	-	1	ID=gene-chloe_ycf1_1
42	OGDRAW	inverted_repeat	128153	154620	.	+	.	ID=repeat_region-ogdraw_repreg1;gbkey=repeat_region
42	blatN	gene	129498	129569	100	+	1	ID=gene-blatn_trnN_1;gbkey=gene;gene=trnN;gene_biotype=tRNA
42	blatN	tRNA	129498	129569	.	+	1	ID=trna-blatn_trnN_1;gbkey=tRNA;gene=trnN
42	blatN	gene	130197	130270	100	-	1	ID=gene-blatn_trnR_1;gbkey=gene;gene=trnR;gene_biotype=tRNA
42	blatN	tRNA	130197	130270	.	-	1	ID=trna-blatn_trnR_1;gbkey=tRNA;gene=trnR
42	blatN	gene	130510	130630	100	-	1	ID=gene-blatn_rrn5S_1;gbkey=gene;gene=rrn5;gene_biotype=rRNA
42	blatN	rRNA	130510	130630	.	-	1	ID=rrna-blatn_rrn5S_1;gbkey=rRNA;gene=rrn5
42	blatN	gene	130878	130980	100	-	1	ID=gene-blatn_rrn4.5S_1;gbkey=gene;gene=rrn4.5;gene_biotype=rRNA
42	blatN	rRNA	130878	130980	.	-	1	ID=rrna-blatn_rrn4.5S_1;gbkey=rRNA;gene=rrn4.5
42	blatN	gene	131079	133888	100	-	1	ID=gene-blatn_rrn23S_1;gbkey=gene;gene=rrn23S;gene_biotype=rRNA
42	blatN	rRNA	131079	133888	.	-	1	ID=rrna-blatn_rrn23S_1;gbkey=rRNA;gene=rrn23S
42	blatN	gene	134041	134914	71.3	-	1	ID=gene-blatn_trnA_1;gbkey=gene;gene=trnA;gene_biotype=tRNA
42	blatN	tRNA	134041	134075	.	-	1	ID=trna-blatn_trnA_1;gbkey=tRNA;gene=trnA
42	blatN	exon	134041	134075	.	-	1	ID=blatn_trnA_1_exon_2;Parent=gene-blatn_trnA_1;gbkey=exon;gene=trnA
42	blatN	intron	134076	134876	.	-	1	ID=blatn_trnA_1_intron_1;Parent=gene-blatn_trnA_1;gbkey=intron;gene=trnA
42	blatN	tRNA	134877	134914	.	-	1	ID=trna-blatn_trnA_1;gbkey=tRNA;gene=trnA
42	blatN	exon	134877	134914	.	-	1	ID=blatn_trnA_1_exon_1;Parent=gene-blatn_trnA_1;gbkey=exon;gene=trnA
42	blatN	gene	134979	135997	69.5	-	1	ID=gene-blatn_trnI_3;gbkey=gene;gene=trnI;gene_biotype=tRNA
42	blatN	tRNA	134979	135013	.	-	1	ID=trna-blatn_trnI_3;gbkey=tRNA;gene=trnI
42	blatN	exon	134979	135013	.	-	1	ID=blatn_trnI_3_exon_2;Parent=gene-blatn_trnI_3;gbkey=exon;gene=trnI
42	blatN	intron	135014	135960	.	-	1	ID=blatn_trnI_3_intron_1;Parent=gene-blatn_trnI_3;gbkey=intron;gene=trnI
42	blatN	tRNA	135961	135997	.	-	1	ID=trna-blatn_trnI_3;gbkey=tRNA;gene=trnI
42	blatN	exon	135961	135997	.	-	1	ID=blatn_trnI_3_exon_1;Parent=gene-blatn_trnI_3;gbkey=exon;gene=trnI
42	blatN	gene	136296	137786	100	-	1	ID=gene-blatn_rrn16S_1;gbkey=gene;gene=rrn16S;gene_biotype=rRNA
42	blatN	rRNA	136296	137786	.	-	1	ID=rrna-blatn_rrn16S_1;gbkey=rRNA;gene=rrn16S

42	blatN	gene	138018	138089	100	-	1	ID=gene-blatn_trnV_1;gbkey=gene;gene=trnV;gene_biotype=tRNA
42	blatN	tRNA	138018	138089	.	-	1	ID=trna-blatn_trnV_1;gbkey=tRNA;gene=trnV
42	blatX	gene	140012	140805	100	+	1	ID=gene-trsp_rps12_1;gbkey=gene;gene=rps12;gene_biotype=protein_coding
42	blatX	CDS	140012	140243	.	+	1	ID=cds-trsp_rps12_1;gbkey=CDS;gene=rps12
42	blatX	exon	140012	140243	.	+	1	ID=trsp_rps12_1_exon_2;Parent=gene-trsp_rps12_1;gbkey=exon;gene=rps12
42	blatX	intron	140244	140780	.	+	1	ID=blatx_rps12_1_intron_1;Parent=gene-blatx_rps12_1;gbkey=intron;gene=rps12
42	blatX	CDS	140781	140805	.	+	1	ID=cds-trsp_rps12_1;gbkey=CDS;gene=rps12
42	blatX	exon	140781	140805	.	+	1	ID=trsp_rps12_1_exon_3;Parent=gene-trsp_rps12_1;gbkey=exon;gene=rps12
42	blatX	gene	140860	141327	100	+	1	ID=gene-blatx_rps7_1;gbkey=gene;gene=rps7;gene_biotype=protein_coding
42	blatX	CDS	140860	141327	.	+	1	ID=cds-blatx_rps7_1;gbkey=CDS;gene=rps7
42	blatX	gene	141641	143864	98.5	+	1	ID=gene-blatx_ndhB_1;gbkey=gene;gene=ndhB;gene_biotype=protein_coding
42	blatX	CDS	141641	142417	.	+	1	ID=cds-blatx_ndhB_1;gbkey=CDS;gene=ndhB
42	blatX	exon	141641	142417	.	+	1	ID=blatx_ndhB_1_exon_1;Parent=gene-blatx_ndhB_1;gbkey=exon;gene=ndhB
42	blatX	intron	142418	143102	.	+	1	ID=blatx_ndhB_1_intron_1;Parent=gene-blatx_ndhB_1;gbkey=intron;gene=ndhB
42	blatX	CDS	143103	143864	.	+	1	ID=cds-blatx_ndhB_1;gbkey=CDS;gene=ndhB
42	blatX	exon	143103	143864	.	+	1	ID=blatx_ndhB_1_exon_2;Parent=gene-blatx_ndhB_1;gbkey=exon;gene=ndhB
42	blatN	gene	144445	144525	100	+	1	ID=gene-blatn_trnL_1;gbkey=gene;gene=trnL;gene_biotype=tRNA
42	blatN	tRNA	144445	144525	.	+	1	ID=trna-blatn_trnL_1;gbkey=tRNA;gene=trnL
42	blatX	gene	145443	152315	99.7	-	1	ID=gene-blatx_ycf2_1;gbkey=gene;gene=ycf2;gene_biotype=protein_coding
42	blatX	CDS	145443	152315	.	-	1	ID=cds-blatx_ycf2_1;gbkey=CDS;gene=ycf2
42	blatN	gene	152404	152477	100	+	1	ID=gene-blatn_trnI_1;gbkey=gene;gene=trnI;gene_biotype=tRNA
42	blatN	tRNA	152404	152477	.	+	1	ID=trna-blatn_trnI_1;gbkey=tRNA;gene=trnI
42	blatX	gene	152646	152927	100	+	1	ID=gene-blatx_rpl23_1;gbkey=gene;gene=rpl23;gene_biotype=protein_coding
42	blatX	CDS	152646	152927	.	+	1	ID=cds-blatx_rpl23_1;gbkey=CDS;gene=rpl23
42	blatX	gene	152946	154452	97.1	+	1	ID=gene-blatx_rpl2_1;gbkey=gene;gene=rpl2;gene_biotype=protein_coding
42	blatX	CDS	152946	153336	.	+	1	ID=cds-blatx_rpl2_1;gbkey=CDS;gene=rpl2
42	blatX	exon	152946	153336	.	+	1	ID=blatx_rpl2_1_exon_1;Parent=gene-blatx_rpl2_1;gbkey=exon;gene=rpl2
42	blatX	intron	153337	154018	.	+	1	ID=blatx_rpl2_1_intron_1;Parent=gene-blatx_rpl2_1;gbkey=intron;gene=rpl2
42	blatX	CDS	154019	154452	.	+	1	ID=cds-blatx_rpl2_1;gbkey=CDS;gene=rpl2
42	blatX	exon	154019	154452	.	+	1	ID=blatx_rpl2_1_exon_2;Parent=gene-blatx_rpl2_1;gbkey=exon;gene=rpl2
42	blatX	gene	154507	154620	100	+	1	ID=gene-blatx_rps19-fragment_1;gbkey=gene;gene=rps19-fragment;gene_biotype=protein_coding
42	blatX	CDS	154507	154620	.	+	1	ID=cds-blatx_rps19-fragment_1;gbkey=CDS;gene=rps19-fragment

Table S4. List of paralogous loci retrieved for GRAMPA reconciliation analysis.

Paralog-loci-GRAMPA-analysis			
5328	AT1G72280.1@7	AT4G05090.1@4	AT5G51200.1@9
5347	AT1G75330.1@2	AT4G17140.3@61	AT5G55250.2@2
5502	AT1G76400.1@10	AT4G20070.1@9	
6198	AT1G79150.1@10	AT4G20090.1@1	
6459	AT2G13370.1@5	AT4G22720.1@2	
6460	AT2G27590.1@3	AT4G24190.2@9	
6532	AT2G28070.1@9	AT4G24790.1@3	
6886	AT2G31970.1@11	AT4G24830.1@6	
AT1G03090.2@14	AT2G32040.1@2	AT4G33440.1@3	
AT1G03190.1@3	AT2G32040.1@6	AT5G01360.1@4	
AT1G05910.1@7	AT2G32590.1@9	AT5G03070.1@10	
AT1G08490.1@3	AT2G33770.1@5	AT5G03070.1@2	
AT1G12470.1@4	AT2G39090.1@16	AT5G04480.2@6	
AT1G14300.2@3	AT2G39260.1@3	AT5G08280.1@3	
AT1G15440.2@6	AT2G39260.1@4	AT5G10900.1@5	
AT1G16070.1@1	AT2G41700.1@9	AT5G13530.1@16	
AT1G19025.1@3	AT2G42850.1@2	AT5G13690.1@16	
AT1G27752.1@8	AT3G04340.1@3	AT5G13690.1@3	
AT1G33410.2@9	AT3G04340.1@4	AT5G14760.1@4	
AT1G43860.1@3	AT3G06810.1@10	AT5G15880.1@6	
AT1G48090.1@62	AT3G17900.1@5	AT5G18525.1@12	
AT1G49820.1@4	AT3G46220.3@2	AT5G20990.1@9	
AT1G50200.2@2	AT3G47400.1@2	AT5G22800.1@5	
AT1G55880.1@5	AT3G48380.1@3	AT5G26820.1@4	
AT1G55910.1@1	AT3G49400.1@16	AT5G40480.1@2	
AT1G60560.1@3	AT3G51050.1@3	AT5G40480.1@3	
AT1G61850.2@1	AT3G54690.1@2	AT5G40480.1@5	
AT1G68570.1@3	AT4G00740.1@4	AT5G45780.1@9	
AT1G69860.1@2	AT4G01730.1@3	AT5G46180.1@3	
AT1G70570.2@2	AT4G01880.1@3	AT5G49430.1@21	

Table S5. Occurrence records of *Catolobus* and *Capsella* species included in habitat suitability modeling.

Species	Longitude	Latitude	bio_1	bio_4	bio_15	bio_16	bio_17
Catolobus_pendulus	38.863889	56.302778	48	9757	34	246	84
Catolobus_pendulus	55.479199	57.893009	27	11238	31	190	72
Catolobus_pendulus	38.351047	54.861344	55	9809	35	224	75
Catolobus_pendulus	129.37722	41.55333	61	10954	86	427	31
Catolobus_pendulus	87.11544	53.754907	28	12021	36	184	55
Catolobus_pendulus	60.631887	56.841913	23	11259	49	244	58
Catolobus_pendulus	109.006562	55.715263	-59	14282	57	210	35
Catolobus_pendulus	106.666098	52.224142	6	13583	82	230	19
Catolobus_pendulus	85.1318	55.4934	8	12433	37	201	58
Catolobus_pendulus	34.333333	54.783333	54	9235	33	267	104
Catolobus_pendulus	107.618538	51.812827	9	13834	102	183	8
Catolobus_pendulus	36.892407	55.740259	52	9550	31	230	88
Catolobus_pendulus	60.561041	56.84385	24	11262	50	231	53
Catolobus_pendulus	86.4217	49.918	-27	10727	59	221	29
Catolobus_pendulus	85.573831	51.566926	29	11174	63	229	34
Catolobus_pendulus	52.448999	55.789526	39	11450	30	191	75
Catolobus_pendulus	135.0557	44.2718	39	11873	65	322	35
Catolobus_pendulus	135.990418	45.53711	18	12782	66	351	37
Catolobus_pendulus	51.2995	54.3607	40	11503	30	182	73
Catolobus_pendulus	103.199131	52.427491	12	12759	88	237	23
Catolobus_pendulus	104.27592	52.239402	9	13117	88	313	27
Catolobus_pendulus	53.2143	56.8827	33	11354	31	195	76
Catolobus_pendulus	138.885556	36.161944	106	8358	63	667	83
Catolobus_pendulus	83.869057	53.129738	30	12716	30	176	66
Catolobus_pendulus	139.004444	36.016944	138	8353	58	507	73
Catolobus_pendulus	46.417547	55.480241	48	10752	28	197	82
Catolobus_pendulus	128.521683	37.295142	107	9190	82	783	77
Catolobus_pendulus	37.600972	54.840278	56	9742	33	223	80
Catolobus_pendulus	86.235239	55.367498	19	12282	36	191	61
Catolobus_pendulus	134.91107	62.628245	-102	22722	67	149	20
Catolobus_pendulus	37.20939	55.711859	51	9591	33	252	95
Catolobus_pendulus	104.343515	52.314796	8	13232	85	283	28

Catolobus_pendulus	107.447319	49.412537	-4	13472	109	295	12
Catolobus_pendulus	138.08	35.52	76	8093	33	593	198
Catolobus_pendulus	128.495833	38.044761	92	8979	76	791	106
Catolobus_pendulus	141.6109	39.974	76	8011	37	563	192
Catolobus_pendulus	37.734986	55.612931	52	9667	31	254	102
Catolobus_pendulus	83.764403	53.299233	27	12752	35	168	54
Catolobus_pendulus	102.425974	51.933383	-30	11946	90	281	23
Catolobus_pendulus	98.8667	28.5	52	5764	68	414	32
Catolobus_pendulus	130.7488	62.7953	-95	21561	61	140	22
Catolobus_pendulus	92.81809	51.318682	-1	14046	85	178	16
Catolobus_pendulus	82.841872	55.0549	15	12852	40	192	50
Catolobus_pendulus	86.3367	54.9512	21	12279	43	186	48
Catolobus_pendulus	90.956988	54.017851	2	12065	85	184	14
Catolobus_pendulus	38.527375	53.134076	59	9994	29	211	84
Catolobus_pendulus	138.698889	36.191111	107	8455	64	566	69
Catolobus_pendulus	104.3303	52.2687	11	13201	87	293	26
Catolobus_pendulus	89.0132	62.2984	-28	14435	34	207	72
Catolobus_pendulus	86.16981	51.11256	29	10875	75	237	22
Catolobus_pendulus	103.870758	52.520408	13	13238	86	265	25
Catolobus_pendulus	103.535994	52.809502	11	13337	87	260	23
Catolobus_pendulus	144.366667	42.983333	79	7995	39	426	121
Catolobus_pendulus	83.872561	53.46981	28	12788	31	172	61
Catolobus_pendulus	138.125	35.35375	61	7835	48	803	180
Catolobus_pendulus	60.540244	56.8322	24	11257	53	240	50
Catolobus_pendulus	41.9167	55.5417	53	10231	31	209	77
Catolobus_pendulus	36.860284	55.914287	52	9528	33	225	80
Catolobus_pendulus	86.7911	53.2902	30	12080	34	217	70
Catolobus_pendulus	83.050026	51.135655	27	11664	33	200	76
Catolobus_pendulus	117.486069	40.565164	40	10854	127	725	7
Catolobus_pendulus	102.7395	57.9845	-16	14522	46	182	43
Catolobus_pendulus	64.2171	54.8021	28	12706	48	184	40
Catolobus_pendulus	85.833333	51	-15	10622	73	242	24
Catolobus_pendulus	40.2515	51.1968	72	10295	24	180	79
Catolobus_pendulus	105.1133	51.556	11	13186	78	443	42
Catolobus_pendulus	131.8048	43.0031	64	10785	67	425	48

Catolobus_pendulus	137.75	36.102083	54	8360	43	620	177
Catolobus_pendulus	138.5625	36.06125	85	8372	48	515	106
Catolobus_pendulus	138.7225	36.011667	85	8295	53	547	93
Catolobus_pendulus	135.2858	44.3522	30	11783	69	365	34
Catolobus_pendulus	134.2522	58.7743	-62	19131	55	144	26
Catolobus_pendulus	37.077819	54.499894	56	9695	31	213	82
Catolobus_pendulus	128.335889	36.956528	117	9411	77	692	73
Catolobus_pendulus	139.08423	36.0085	140	8316	49	459	90
Catolobus_pendulus	39.538688	51.84264	67	10181	24	200	94
Catolobus_pendulus	86.109555	55.38232	15	12265	36	219	70
Catolobus_pendulus	126.466915	63.582619	-85	20243	55	120	24
Catolobus_pendulus	65.067489	57.059114	20	12328	48	210	46
Catolobus_pendulus	83.069356	54.883762	18	12881	41	189	50
Catolobus_pendulus	53.2508	56.8388	31	11346	28	200	85
Catolobus_pendulus	138.0625	36.26875	76	8471	40	583	168
Catolobus_pendulus	102.593223	53.56405	3	13514	72	225	27
Catolobus_pendulus	85.1779	56.1853	9	12475	35	212	66
Catolobus_pendulus	37.383561	55.757991	52	9614	32	251	98
Catolobus_pendulus	55.6968	58.5601	22	11085	37	211	68
Catolobus_pendulus	103.88946	52.530665	13	13258	86	259	25
Catolobus_pendulus	132.8221	42.7611	65	9840	61	407	50
Catolobus_pendulus	85.0043	56.7794	7	12660	39	203	55
Catolobus_pendulus	105.4232	51.236	-19	13370	82	332	29
Catolobus_pendulus	99.0263	54.9012	10	12430	92	245	19
Catolobus_pendulus	85.605143	51.611127	28	11171	60	251	41
Catolobus_pendulus	104.312095	51.485471	7	12846	89	520	35
Catolobus_pendulus	87.125755	53.746147	25	11957	37	216	64
Catolobus_pendulus	139.1336	48.1572	12	12847	53	300	47
Catolobus_pendulus	48.5699	54.6746	45	11092	34	206	71
Catolobus_pendulus	113.05793	51.791322	1	14336	113	236	6
Catolobus_pendulus	87.819435	55.755212	18	12034	42	214	55
Catolobus_pendulus	128.386806	35.189167	124	8486	69	951	109
Catolobus_pendulus	83.003567	54.99352	16	12861	39	188	51
Catolobus_pendulus	132.8817	53.0663	-34	16614	84	373	17
Catolobus_pendulus	49.386425	53.47837	52	11526	20	174	89

Catolobus_pendulus	37.686634	56.534254	50	9628	31	241	90
Catolobus_pendulus	94.933	55.8196	7	12399	61	210	32
Catolobus_pendulus	94.52306	58.16919	-7	13808	40	170	45
Catolobus_pendulus	43.766849	55.417372	49	10413	33	201	68
Catolobus_pendulus	114.8393	50.8228	1	14807	113	289	9
Catolobus_pendulus	107.780347	51.224014	4	13795	106	191	9
Catolobus_pendulus	129.143639	37.074306	90	8404	61	715	130
Catolobus_pendulus	40.25	56.125	48	9954	28	221	91
Catolobus_pendulus	105.511464	53.064355	-9	14000	99	222	13
Catolobus_pendulus	128.528644	37.276192	110	9210	84	793	73
Catolobus_pendulus	105.2761	51.184	-17	13305	86	319	26
Catolobus_pendulus	85.86108	50.83839	11	10867	75	199	18
Catolobus_pendulus	65.627777	57.123596	19	12417	53	228	43
Catolobus_pendulus	56.231087	57.983034	24	11176	31	223	88
Catolobus_pendulus	60.229013	55.151881	25	11622	50	231	52
Catolobus_pendulus	51.963144	55.726498	40	11419	25	189	84
Catolobus_pendulus	85.683005	50.19765	9	11039	61	160	21
Catolobus_pendulus	83.093901	54.898975	15	12842	40	207	56
Catolobus_pendulus	132.9284	42.8654	65	10016	68	397	37
Catolobus_pendulus	127.26667	53.75	-23	16640	106	401	10
Catolobus_pendulus	82.895442	54.98485	16	12865	44	196	47
Catolobus_pendulus	92.739788	55.964745	16	12383	52	201	39
Catolobus_pendulus	52.099665	55.07486	43	11536	31	170	63
Catolobus_pendulus	166.845525	60.95571	-29	11353	36	167	61
Catolobus_pendulus	91.5825	54.1917	27	12527	77	186	17
Catolobus_pendulus	82.968735	51.154359	25	11674	30	208	86
Catolobus_pendulus	63.6297	56.087	28	12319	46	179	43
Catolobus_pendulus	83.765945	53.23851	31	12763	31	155	56
Catolobus_pendulus	72.7237	58.6937	3	12421	50	227	47
Catolobus_pendulus	37.638511	56.506931	50	9618	31	249	93
Catolobus_pendulus	128.293528	37.822333	80	9217	76	930	108
Catolobus_pendulus	142.9429	49.8508	10	11312	50	291	54
Catolobus_pendulus	65.438056	57.135553	18	12356	53	236	45
Catolobus_pendulus	35.911896	56.859611	51	9379	33	246	87
Catolobus_pendulus	127.372422	53.936519	-35	16505	103	406	11

Catolobus_pendulus	137.75	36.06125	65	8398	52	654	143
Catolobus_pendulus	132.6861	44.8573	50	13265	69	339	37
Catolobus_pendulus	85.12778	50.15278	0	11068	54	166	28
Catolobus_pendulus	64.1371	57.1325	20	12129	47	202	47
Catolobus_pendulus	37.295034	55.760098	54	9620	31	228	91
Catolobus_pendulus	116.57222	51.97861	-5	15983	109	219	9
Catolobus_pendulus	138.1	35.5	77	8090	34	596	192
Catolobus_pendulus	105.2234	51.18	-27	13178	84	333	29
Catolobus_pendulus	100.71667	31.52	30	6337	86	321	9
Catolobus_pendulus	103.048611	48.948056	-20	12494	124	281	3
Catolobus_pendulus	81.54676	53.93524	16	12985	43	163	41
Catolobus_pendulus	136.1642	57.6601	-59	17814	64	202	27
Catolobus_pendulus	66.561103	56.505192	19	12526	51	210	42
Catolobus_pendulus	41.4167	55.7917	50	10126	29	223	87
Catolobus_pendulus	87.4547	52.9392	26	11579	29	263	106
Catolobus_pendulus	96.605278	31.184167	20	6745	94	229	4
Catolobus_pendulus	150.921062	59.631363	-33	11940	59	180	23
Catolobus_pendulus	137.875	36.102083	108	8603	48	395	93
Catolobus_pendulus	43.75	51.29167	64	10956	22	168	79
Catolobus_pendulus	109.368777	55.657214	-30	14697	51	156	30
Catolobus_pendulus	102.137905	51.681459	9	12490	95	275	21
Catolobus_pendulus	56.276344	53.188082	40	11985	26	168	73
Catolobus_pendulus	97.3569	58.0536	-7	14141	40	151	41
Catolobus_pendulus	65.567787	57.141472	19	12396	55	233	42
Catolobus_pendulus	41.4167	55.625	51	10142	29	209	82
Catolobus_pendulus	55.9643	53.6528	45	11919	25	179	80
Catolobus_pendulus	138.9	36.2	119	8420	52	546	97
Catolobus_pendulus	54.087279	55.466607	35	11498	35	184	61
Catolobus_pendulus	89.5837	56.1744	12	12163	42	197	48
Catolobus_pendulus	128.09425	36.822833	102	9435	80	834	85
Catolobus_pendulus	92.735434	55.926608	3	12236	57	243	42
Catolobus_pendulus	62.159657	57.373444	26	11535	52	218	45
Catolobus_pendulus	103.8911	51.9526	-2	12614	86	389	33
Catolobus_pendulus	60.648204	56.91061	22	11251	52	252	55
Catolobus_pendulus	40.5833	55.375	53	10064	28	223	87

Catolobus_pendulus	138.4375	36.309583	113	8575	48	474	108
Catolobus_pendulus	94.3034	55.6347	7	12247	55	204	36
Catolobus_pendulus	50.028842	58.546737	27	10792	32	241	90
Catolobus_pendulus	56.0418	54.4192	42	11706	27	213	90
Catolobus_pendulus	38.559824	54.854087	56	9853	31	208	75
Catolobus_pendulus	138.344509	36.455582	84	8465	40	611	183
Catolobus_pendulus	102.815278	49.144444	7	12760	126	199	3
Catolobus_pendulus	103.727214	52.456358	11	13075	86	301	29
Catolobus_pendulus	130.9899	63.232	-93	21494	62	155	21
Catolobus_pendulus	55.9628	58.0127	26	11196	29	202	85
Catolobus_pendulus	132.3947	44.5292	53	12957	71	331	32
Catolobus_pendulus	131.913488	43.179407	65	11124	67	385	40
Catolobus_pendulus	38.35	52.57	61	9980	29	225	89
Catolobus_pendulus	128.600067	37.276225	108	9109	77	744	85
Catolobus_pendulus	138.6875	35.97625	68	8228	54	599	97
Catolobus_pendulus	103.212482	52.893603	10	13261	81	223	22
Catolobus_pendulus	62.7222	56.4288	27	11979	54	197	37
Catolobus_pendulus	40.752708	52.843443	61	10356	25	200	93
Catolobus_pendulus	41.677385	55.802201	51	10169	30	212	80
Catolobus_pendulus	43.3924	52.3786	58	10759	20	183	92
Catolobus_pendulus	144.4948	43.1471	76	8153	37	389	115
Catolobus_pendulus	128.512226	50.917349	0	16336	93	377	13
Catolobus_pendulus	100.9097	31.9208	22	6491	93	431	10
Catolobus_pendulus	146.327	43.64278	61	6731	33	442	140
Catolobus_pendulus	142.0115	48.7821	2	9799	28	286	125
Catolobus_pendulus	77.6353	55.1782	15	13108	50	161	35
Catolobus_pendulus	86.136908	55.402999	14	12247	36	213	69
Catolobus_pendulus	83.706492	55.038183	13	12741	41	180	46
Catolobus_pendulus	89.446376	54.457284	3	11820	30	214	76
Catolobus_pendulus	38.366705	54.902097	55	9803	30	220	83
Catolobus_pendulus	67.9954	57.6089	14	12549	47	209	47
Catolobus_pendulus	133.9572	43.0994	39	10215	58	361	53
Catolobus_pendulus	87.5891	54.8516	19	11924	25	341	149
Catolobus_pendulus	137.6875	35.935417	80	8426	54	753	153
Catolobus_pendulus	69.457	56.1091	19	12757	56	188	36

Catolobus_pendulus	61.002951	56.900545	26	11336	52	224	49
Catolobus_pendulus	138.09328	35.719147	83	8298	40	577	150
Catolobus_pendulus	103.730898	51.659523	12	12666	84	488	42
Catolobus_pendulus	104.32579	52.225104	11	13154	90	304	24
Catolobus_pendulus	34.801809	56.246857	52	9229	34	243	86
Catolobus_pendulus	56.9226	57.4756	26	11298	35	203	65
Catolobus_pendulus	133.5926	62.6328	-103	22403	68	134	16
Catolobus_pendulus	138.45236	35.97814	70	8312	67	631	71
Catolobus_pendulus	101.1367	32.6397	26	6772	81	270	11
Catolobus_pendulus	123.592656	54.107072	-26	16502	93	278	11
Catolobus_pendulus	89.467656	54.445561	0	11796	29	262	95
Catolobus_pendulus	111.0527	59.4711	-38	16005	42	159	37
Catolobus_pendulus	93.0076	56.2333	10	12397	56	192	32
Catolobus_pendulus	150.799949	59.560158	-29	11502	55	201	30
Catolobus_pendulus	44.502934	55.443394	50	10514	28	197	79
Catolobus_pendulus	97.270556	32.143056	19	6834	99	202	4
Catolobus_pendulus	85.027401	56.466629	10	12590	35	209	63
Catolobus_pendulus	127.266917	53.730731	-16	16746	102	335	9
Catolobus_pendulus	134.120513	43.003832	55	9976	70	379	39
Catolobus_pendulus	51.6058	57.1091	34	11181	33	221	84
Catolobus_pendulus	132.4405	61.9966	-94	21815	69	129	16
Catolobus_pendulus	107.425061	47.93432	-32	13670	117	251	6
Catolobus_pendulus	137.9979	35.495	80	8081	38	773	216
Catolobus_pendulus	42.0833	55.5417	54	10263	32	204	73
Catolobus_pendulus	103.3815	52.2636	5	12672	91	330	28
Catolobus_pendulus	87.829685	51.348735	33	11039	75	242	22
Catolobus_pendulus	83.9288	51.367	1	11234	48	284	64
Catolobus_pendulus	102.6909	58.0237	-12	14585	49	146	31
Catolobus_pendulus	38.543353	53.200932	58	9982	28	224	91
Catolobus_pendulus	137.98255	36.226315	119	8662	42	440	122
Catolobus_pendulus	138.4975	36.488333	87	8457	46	420	116
Catolobus_pendulus	59.896862	56.808637	19	11175	48	227	54
Catolobus_pendulus	144.5	43.666667	59	8483	30	312	122
Catolobus_pendulus	108.0065	61.2749	-50	16384	47	149	32
Catolobus_pendulus	100.6451	54.6573	1	13182	75	238	24

Catolobus_pendulus	135.103848	44.345218	23	11869	68	416	40
Catolobus_pendulus	91.46687	62.15161	-33	14896	32	208	74
Catolobus_pendulus	43.291236	51.761602	62	10815	21	186	92
Catolobus_pendulus	128.502359	41.303216	16	11321	84	530	37
Catolobus_pendulus	104.2474	51.8663	1	12727	83	337	33
Catolobus_pendulus	126.03928	38.47079	108	10169	112	860	34
Catolobus_pendulus	58.709882	55.145522	26	11522	44	240	63
Catolobus_pendulus	103.064363	52.859132	13	13132	79	219	24
Catolobus_pendulus	83.610931	53.220436	25	12719	33	176	61
Catolobus_pendulus	56.942249	57.434599	28	11325	39	192	55
Catolobus_pendulus	89.0427	62.3603	-30	14448	34	213	72
Catolobus_pendulus	128.345375	41.19054	18	11342	87	565	37
Catolobus_pendulus	92.7313	55.8673	1	12199	46	229	50
Catolobus_pendulus	142.3298	43.7526	76	9416	31	360	149
Catolobus_pendulus	128.067037	38.612792	82	9249	67	929	157
Catolobus_pendulus	100.941	31.9744	37	6429	94	299	6
Catolobus_pendulus	37.97347	51.340535	68	9925	23	205	97
Catolobus_pendulus	95.67	56.0048	7	12685	63	182	26
Catolobus_pendulus	128.311472	37.366606	97	9341	93	1012	74
Catolobus_pendulus	60.594803	56.79324	25	11278	53	227	49
Catolobus_pendulus	103.636244	52.739074	11	13315	88	231	20
Catolobus_pendulus	144.130154	43.409366	51	8689	39	396	124
Catolobus_pendulus	98.911543	28.48611	57	5745	73	414	29
Catolobus_pendulus	85.59233	51.45839	16	11014	65	292	39
Catolobus_pendulus	83.1061	54.82625	15	12842	40	210	57
Catolobus_pendulus	121.08611	56.05556	-60	16250	89	278	15
Catolobus_pendulus	82.814375	54.971316	17	12884	45	186	43
Catolobus_pendulus	59.0562	54.7925	0	11307	42	289	80
Catolobus_pendulus	86.093416	51.221136	22	10848	73	269	27
Catolobus_pendulus	90.867782	54.501058	11	12210	90	240	18
Catolobus_pendulus	138.25	35.809583	86	8333	55	520	84
Catolobus_pendulus	127.445655	39.147502	108	10227	86	1011	111
Catolobus_pendulus	60.0669	55.1591	27	11627	48	231	54
Catolobus_pendulus	57.860721	54.858015	25	11507	40	228	67
Catolobus_pendulus	142.0601	46.8047	34	8903	31	318	114

Catolobus_pendulus	138.16342	36.47575	124	8647	38	356	132
Catolobus_pendulus	143.1467	59.3681	-23	12149	65	225	28
Catolobus_pendulus	144.1117	43.3351	49	8681	49	456	107
Catolobus_pendulus	137.9375	36.394583	116	8632	39	498	171
Catolobus_pendulus	131.987	60.8969	-79	20872	66	133	17
Catolobus_pendulus	106.494128	52.804375	-2	13558	98	187	10
Catolobus_pendulus	132.035065	43.193015	55	11025	67	423	46
Catolobus_pendulus	36.277071	51.531642	66	9661	21	229	117
Catolobus_pendulus	108.489132	50.936418	-4	13383	108	223	9
Catolobus_pendulus	104.070435	52.208779	13	13057	84	258	26
Catolobus_pendulus	42.1511	49.8017	76	10551	16	156	97
Catolobus_pendulus	39.54612	51.841184	66	10179	24	204	97
Catolobus_pendulus	88.0288	52.8795	7	11267	36	310	100
Catolobus_pendulus	43.669762	51.24114	67	10961	21	151	77
Catolobus_pendulus	48.8482	54.3575	51	11257	33	191	67
Catolobus_pendulus	46.6332	61.253	23	10553	32	207	76
Catolobus_pendulus	128.331342	37.386597	83	9270	89	1034	86
Catolobus_pendulus	85.766936	51.658583	34	11197	61	267	40
Catolobus_pendulus	57.063298	53.042061	30	11936	24	179	88
Catolobus_pendulus	81.65105	54.133248	20	13038	43	152	38
Catolobus_pendulus	103.702332	51.719667	14	12648	77	320	38
Catolobus_pendulus	86.002742	51.397817	34	10999	69	247	29
Catolobus_pendulus	104.433844	52.241275	9	13188	84	287	28
Catolobus_pendulus	62.5935	55.9128	30	12153	55	203	37
Catolobus_pendulus	60.646041	56.812252	25	11284	50	228	52
Catolobus_pendulus	128.761111	37.683333	91	8691	76	920	120
Catolobus_pendulus	138.139184	35.783521	81	8313	38	518	145
Catolobus_pendulus	159.755835	55.537908	3	10663	33	209	64
Catolobus_pendulus	94.1612	55.4011	3	12120	53	253	46
Catolobus_pendulus	48.6615	54.4178	51	11204	37	209	66
Catolobus_pendulus	104.241898	52.293589	13	13217	88	254	23
Catolobus_pendulus	86.366667	50.116667	-2	10882	63	243	30
Catolobus_pendulus	105.754711	50.512242	12	14326	110	217	9
Catolobus_pendulus	45.810885	53.997771	53	10773	33	197	69
Catolobus_pendulus	88.515556	52.293889	18	11239	47	279	60

Catolobus_pendulus	97.3038	57.44097	0	13776	35	162	53
Catolobus_pendulus	88.742252	52.54219	21	11319	47	269	57
Catolobus_pendulus	133.124647	43.020438	59	10401	68	393	38
Catolobus_pendulus	166.059534	60.427737	-19	9802	38	183	67
Catolobus_pendulus	45.586051	53.107427	54	10899	20	171	88
Catolobus_pendulus	113.510123	52.033445	-3	14628	108	283	10
Catolobus_pendulus	132.8609	42.7826	64	9824	65	419	44
Catolobus_pendulus	132.2159	57.6448	-91	17204	71	223	27
Catolobus_pendulus	81.917879	54.359192	20	13005	41	153	40
Catolobus_pendulus	87.0643	54.1626	23	12035	36	182	56
Catolobus_pendulus	126.719743	54.124783	-32	16382	97	342	12
Catolobus_pendulus	108.8568	52.8685	-34	12865	73	267	19
Catolobus_pendulus	158.287725	57.725549	-9	10339	49	186	42
Catolobus_pendulus	103.508326	52.394973	12	12918	89	304	26
Catolobus_pendulus	37.63124	55.620533	52	9659	30	237	97
Catolobus_pendulus	63.762	56.3542	24	12251	49	191	43
Catolobus_pendulus	103.256723	52.192759	13	12618	95	268	20
Catolobus_pendulus	104.88719	52.778852	-3	13782	98	231	14
Catolobus_pendulus	60.126959	56.683827	20	11200	53	230	48
Catolobus_pendulus	87.2191	54.2065	23	11971	30	194	72
Catolobus_pendulus	104.83301	51.867968	11	13041	78	297	32
Catolobus_pendulus	102.049	53.9207	4	13545	73	184	20
Catolobus_pendulus	89.911416	54.324468	1	11857	59	191	31
Catolobus_pendulus	128.02186	37.30127	97	9530	83	954	98
Catolobus_pendulus	94.4938	55.4972	9	12201	59	228	37
Catolobus_pendulus	102.5203	30.8553	23	6191	81	365	7
Catolobus_pendulus	58.373557	55.121887	21	11454	43	254	69
Catolobus_pendulus	106.4656	54.2391	-17	13887	97	212	12
Catolobus_pendulus	141.6815	39.26833	82	7825	38	662	178
Catolobus_pendulus	38.9167	56.2083	49	9775	35	255	86
Catolobus_pendulus	41.798449	54.825946	56	10302	27	200	84
Catolobus_pendulus	86.1775	55.461	14	12235	38	196	60
Catolobus_pendulus	55.9228	53.3796	45	11987	27	170	71
Catolobus_pendulus	128.52377	37.463625	76	9016	76	987	119
Catolobus_pendulus	141.38	40.12	87	8152	29	490	224

Catolobus_pendulus	128.56081	36.213609	133	9273	75	539	56
Catolobus_pendulus	69.45	56.15	18	12744	55	189	37
Catolobus_pendulus	47.1358	52.691	51	11274	16	168	100
Catolobus_pendulus	138.0625	35.394583	91	8007	41	673	177
Catolobus_pendulus	101.0064	32.1428	33	6502	85	257	8
Catolobus_pendulus	148.278082	62.832418	-103	17666	75	164	18
Catolobus_pendulus	69.1874	58.7968	7	12327	49	219	46
Catolobus_pendulus	103.435763	51.748373	6	12521	86	356	31
Catolobus_pendulus	83.02	51.1298	28	11686	32	197	74
Catolobus_pendulus	99.626944	29.101667	52	5919	123	257	1
Catolobus_pendulus	60.149246	55.140915	27	11629	48	254	60
Catolobus_pendulus	104.714705	52.994002	-4	13833	98	216	13
Catolobus_pendulus	95.5521	57.832	-6	13681	42	165	42
Catolobus_pendulus	62.5836	56.2832	31	12035	54	188	36
Catolobus_pendulus	129.819946	61.874605	-80	20802	58	132	23
Catolobus_pendulus	138.039044	35.457809	72	7997	40	797	213
Catolobus_pendulus	83.63609	54.47027	13	12731	45	196	46
Catolobus_pendulus	132.8292	42.8002	63	9940	59	401	52
Catolobus_pendulus	97.4451	58.3799	-7	14402	47	146	32
Catolobus_pendulus	49.2213	53.8523	53	11439	24	182	84
Catolobus_pendulus	100.174167	50.46	-34	13405	118	185	3
Catolobus_pendulus	55.540009	55.420051	42	11605	31	190	72
Catolobus_pendulus	142.817778	43.655833	14	9335	32	591	245
Catolobus_pendulus	41.633	55.3346	51	10190	29	222	87
Catolobus_pendulus	138.182611	35.720861	29	8066	43	626	155
Catolobus_pendulus	93.3078	56.8785	12	12791	51	172	32
Catolobus_pendulus	42.975757	52.42853	57	10685	23	195	93
Catolobus_pendulus	140.886003	51.501936	0	12360	48	299	66
Catolobus_pendulus	123.701955	54.092924	-28	16451	90	292	13
Catolobus_pendulus	86.3166	55.3916	17	12240	37	184	58
Catolobus_pendulus	138.1025	35.559722	68	8095	37	686	197
Catolobus_pendulus	89.503883	54.454249	-11	11675	31	312	109
Catolobus_pendulus	38.05402	53.072432	60	9927	29	208	84
Catolobus_pendulus	89.4651	54.483	0	11803	30	288	101
Catolobus_pendulus	103.0755	53.1398	1	13356	80	236	25

Catolobus_pendulus	39.525481	52.247308	65	10194	26	208	89
Catolobus_pendulus	143.7752	50.0378	-7	10563	42	351	88
Catolobus_pendulus	141.954722	39.461389	115	7707	44	558	136
Catolobus_pendulus	111.22	55.8558	-28	15297	85	189	15
Catolobus_pendulus	103.877678	52.543175	12	13298	86	262	25
Catolobus_pendulus	80.8827	54.0069	17	13082	47	154	35
Catolobus_pendulus	93.2521	56.3054	12	12474	58	185	29
Catolobus_pendulus	135.0864	48.4645	18	14877	79	478	36
Catolobus_pendulus	104.319285	52.347587	9	13263	83	253	27
Catolobus_pendulus	107.601597	51.839777	2	13737	96	171	8
Catolobus_pendulus	135.060864	44.247329	37	11747	62	312	41
Catolobus_pendulus	54.168998	56.130204	38	11516	32	194	69
Catolobus_pendulus	87.00833	50.06111	-63	10457	72	303	31
Catolobus_pendulus	106.972778	47.914444	-5	13854	114	179	4
Catolobus_pendulus	37.398482	55.74261	51	9612	30	247	101
Catolobus_pendulus	128.078622	38.164697	100	9525	83	838	80
Catolobus_pendulus	64.6059	56.7781	20	12288	51	202	41
Catolobus_pendulus	131.206216	42.579968	67	10244	80	527	35
Catolobus_pendulus	85.821379	57.18553	3	12725	45	206	46
Catolobus_pendulus	136.136	45.2544	0	11811	68	451	45
Catolobus_pendulus	101.41	47.5	-11	12190	105	256	7
Catolobus_pendulus	134.2514	62.1961	-102	22891	68	138	17
Catolobus_pendulus	132.869	42.8163	62	9915	64	410	46
Catolobus_pendulus	52.7354	58.1493	27	11042	30	203	84
Catolobus_pendulus	67.9199	58.2558	12	12383	47	219	50
Catolobus_pendulus	104.972324	52.874803	-5	13845	99	272	16
Catolobus_pendulus	57.670633	54.973876	32	11568	37	218	69
Catolobus_pendulus	46.410592	55.471472	49	10755	29	196	79
Catolobus_pendulus	104.215197	52.207039	12	13078	86	283	26
Catolobus_pendulus	47.225569	56.135563	45	10791	35	198	67
Catolobus_pendulus	88.0597	54.1341	15	11712	23	430	200
Catolobus_pendulus	60.049013	57.433284	19	11193	55	226	47
Catolobus_pendulus	13.374934	52.474986	94	6683	21	189	106
Catolobus_pendulus	86.5	50.1	-10	10820	70	263	26
Catolobus_pendulus	125.753471	39.032011	109	10493	106	751	35

Catolobus_pendulus	52.2872	55.6823	42	11473	30	182	69
Catolobus_pendulus	86.073495	55.327767	18	12319	42	191	53
Catolobus_pendulus	127.536617	50.268988	10	15962	97	363	12
Catolobus_pendulus	132.803467	43.198678	43	10842	68	395	35
Catolobus_pendulus	49.447758	53.50528	52	11529	24	174	79
Catolobus_pendulus	105.708105	54.005885	-10	14028	86	194	15
Catolobus_pendulus	132.5189	43.6412	49	11999	76	370	27
Catolobus_pendulus	144.404967	42.976226	78	7957	37	432	126
Catolobus_pendulus	37.473469	55.806563	52	9624	29	257	108
Catolobus_pendulus	76.5	56.5	10	12810	51	186	36
Catolobus_pendulus	138	36.687083	99	8443	25	547	278
Catolobus_pendulus	84.7842	50.9892	2	11104	59	229	36
Catolobus_pendulus	36.522305	49.735606	82	9736	18	176	96
Catolobus_pendulus	103.795505	51.629735	20	12758	81	414	38
Catolobus_pendulus	49.662525	53.435437	51	11562	27	168	69
Catolobus_pendulus	82.918961	51.134937	22	11660	28	217	95
Catolobus_pendulus	101.53333	30.28333	34	5602	80	362	10
Catolobus_pendulus	85.725854	50.224664	20	11118	61	150	20
Catolobus_pendulus	61.35912	55.194409	31	11999	54	219	44
Catolobus_pendulus	143.3311	46.4955	43	8585	33	302	112
Catolobus_pendulus	37.543345	51.453447	69	9881	26	199	87
Catolobus_pendulus	141.652222	45.445278	64	8255	30	408	176
Catolobus_pendulus	135.725313	44.334271	48	11300	67	336	36
Catolobus_pendulus	39.067286	51.980426	68	10128	26	193	85
Catolobus_pendulus	60.528026	56.83468	24	11262	51	227	51
Catolobus_pendulus	167.972734	63.171063	-52	13967	38	161	48
Catolobus_pendulus	129.183333	49.983333	4	16067	90	370	16
Catolobus_pendulus	61.358193	55.121354	29	12011	52	238	50
Catolobus_pendulus	96.9703	57.533	0	13789	38	153	46
Catolobus_pendulus	122.9902	54.4666	-42	16492	93	290	14
Catolobus_pendulus	127.982972	37.085333	117	9612	89	943	84
Catolobus_pendulus	92.7586	56.0292	15	12386	56	194	34
Catolobus_pendulus	128.2075	36.775	104	9412	82	874	86
Catolobus_pendulus	84.858769	50.443644	1	11154	47	197	44
Catolobus_pendulus	58.736315	54.599213	15	11510	46	201	51

Catolobus_pendulus	138.738611	36.0775	99	8366	53	486	84
Catolobus_pendulus	138.125	36.227917	62	8398	50	552	124
Catolobus_pendulus	158.715783	55.939517	-23	10836	55	183	30
Catolobus_pendulus	83.674538	53.265533	25	12725	34	179	60
Catolobus_pendulus	38.9167	56.2917	47	9761	34	256	87
Catolobus_pendulus	102.6936	30.9972	42	6245	81	312	7
Catolobus_pendulus	87.985037	51.085116	26	11061	73	190	21
Catolobus_pendulus	138.46014	35.99622	82	8352	60	516	69
Catolobus_pendulus	92.8802	56.0002	19	12438	51	189	36
Catolobus_pendulus	37.361862	55.782981	52	9612	32	255	99
Catolobus_pendulus	127.465966	35.620755	103	8962	80	951	104
Catolobus_pendulus	52.13	57.318	31	11201	30	213	86
Catolobus_pendulus	90.107233	54.484511	11	12040	66	191	26
Catolobus_pendulus	128.636022	37.362061	98	8995	74	868	108
Catolobus_pendulus	87.179654	53.753058	27	11964	32	215	75
Catolobus_pendulus	125.714647	37.614294	118	9120	93	841	61
Catolobus_pendulus	105.282893	52.118122	-7	13224	95	247	16
Catolobus_pendulus	128.592381	37.731301	86	8888	77	863	104
Catolobus_pendulus	41.99026	55.40759	54	10256	30	205	78
Catolobus_pendulus	39.485235	51.856378	68	10180	24	200	94
Catolobus_pendulus	56.503254	53.092339	37	11973	27	191	83
Catolobus_pendulus	95.87321	56.09773	12	12833	63	177	26
Catolobus_pendulus	66.3122	56.6546	21	12532	55	210	38
Catolobus_pendulus	103.88882	51.734074	14	12711	74	318	42
Catolobus_pendulus	135.153522	48.454981	20	14857	76	417	35
Catolobus_pendulus	104.8367	54.6979	-6	13936	78	205	19
Catolobus_pendulus	86.3265	54.8347	19	12272	41	192	54
Catolobus_pendulus	67.8667	61.3572	-8	12868	41	225	64
Catolobus_pendulus	128.64	37.535833	74	8849	73	1013	135
Catolobus_pendulus	41.75	55.125	56	10264	27	197	81
Catolobus_pendulus	138.51784	35.95033	74	8288	53	576	98
Catolobus_pendulus	64.683824	69.095715	-62	10735	38	109	38
Catolobus_pendulus	41.503088	57.454889	40	10142	32	227	84
Catolobus_pendulus	41.4167	55.7083	51	10141	29	205	79
Catolobus_pendulus	137.7188	54.9319	-23	11220	41	205	54

Catolobus_pendulus	132.227403	43.621184	52	11912	68	314	29
Catolobus_pendulus	102.317359	56.125658	-8	13760	57	194	32
Catolobus_pendulus	87.4237	53.4064	24	11732	30	256	104
Catolobus_pendulus	127.764816	41.416985	42	12174	99	485	21
Catolobus_pendulus	92.887653	55.977086	19	12425	59	197	31
Catolobus_pendulus	105.3792	50.970867	-27	13479	91	315	23
Catolobus_pendulus	61.314793	55.240108	30	11964	56	231	44
Catolobus_pendulus	41.187637	55.056004	54	10180	28	227	92
Catolobus_pendulus	128.909058	36.991139	104	8827	81	710	73
Catolobus_pendulus	137.6875	36.142917	83	8483	46	537	152
Catolobus_pendulus	84.15833	51.06667	17	11298	53	226	43
Catolobus_pendulus	138.4375	36.227917	112	8565	50	383	79
Catolobus_pendulus	54.151515	53.907208	33	11734	26	166	75
Catolobus_pendulus	90.787411	53.246728	33	12055	78	196	20
Catolobus_pendulus	40.63	55.4	53	10072	28	219	86
Catolobus_pendulus	59.941547	60.152892	6	11879	52	221	53
Catolobus_pendulus	84.055206	53.093349	29	12668	29	173	66
Catolobus_pendulus	85.4335	55.5184	14	12438	38	190	55
Catolobus_pendulus	142.7246	47.054	39	9210	32	329	127
Catolobus_pendulus	37.450693	55.795308	54	9633	29	234	97
Catolobus_pendulus	91.7601	54.2939	19	12494	80	198	17
Catolobus_pendulus	109.606454	64.104226	-75	17487	56	154	28
Catolobus_pendulus	78.5671	60.2073	-9	13179	49	229	48
Catolobus_pendulus	127.871071	36.543569	98	9402	81	939	101
Catolobus_pendulus	113.6409	59.7507	-43	15912	39	196	55
Catolobus_pendulus	162.305195	58.56693	-9	10144	30	260	106
Catolobus_pendulus	143.205329	42.872246	80	9005	38	429	135
Catolobus_pendulus	56.200509	55.24951	37	11544	26	206	96
Catolobus_pendulus	82.990067	54.998131	18	12884	42	175	45
Catolobus_pendulus	84.349517	51.386572	5	11214	55	243	45
Catolobus_pendulus	73.3682	54.9893	20	13183	48	162	37
Catolobus_pendulus	129.75083	42.44278	64	11459	87	376	20
Catolobus_pendulus	138.168	35.7701	61	8220	39	568	155
Catolobus_pendulus	118.274604	56.798395	-49	15577	95	214	9
Catolobus_pendulus	141.2321	46.2546	51	8351	27	334	148

Catolobus_pendulus	95.606	50.5454	-43	13126	91	161	15
Catolobus_pendulus	63.7333	57.0142	24	12108	52	210	42
Catolobus_pendulus	66.0192	55.9357	24	12580	51	182	36
Catolobus_pendulus	102.012177	47.771176	10	12301	108	171	5
Catolobus_pendulus	138.704722	36.054167	100	8388	59	473	65
Catolobus_pendulus	113.992812	51.660751	1	14619	112	245	7
Catolobus_pendulus	57.577015	53.229139	32	11950	32	185	69
Catolobus_pendulus	128.141121	51.366999	0	16265	91	348	13
Catolobus_pendulus	142.074271	47.771866	36	9279	25	301	151
Catolobus_pendulus	46.2978	60.7611	25	10450	29	186	74
Catolobus_pendulus	56.566996	58.174558	21	11126	27	220	96
Catolobus_pendulus	93.00556	51.09444	-47	13811	70	159	23
Catolobus_pendulus	93.711098	56.508949	16	12664	52	170	31
Catolobus_pendulus	84.7341	51.1766	-1	11073	65	270	35
Catolobus_pendulus	155.374027	62.572585	-116	16652	55	164	30
Catolobus_pendulus	61.363731	55.131695	29	11996	54	245	49
Catolobus_pendulus	119.961044	57.636967	-75	15355	85	261	22
Catolobus_pendulus	129.6107	62.0207	-84	20829	61	115	19
Catolobus_pendulus	95.383	55.692	10	12396	59	217	34
Catolobus_pendulus	87.01944	51.13889	-46	10252	67	325	38
Catolobus_pendulus	89.35	43.89	73	12799	56	75	11
Catolobus_pendulus	138.416667	35.716667	133	8408	58	509	76
Catolobus_pendulus	80.853002	59.065599	-5	13041	53	208	38
Catolobus_pendulus	128.63694	41.65806	1	11434	83	661	51
Catolobus_pendulus	67.1497	57.6122	15	12528	45	218	53
Catolobus_pendulus	83.171524	51.677422	32	11975	33	202	73
Catolobus_pendulus	52.4973	57.7022	25	11118	26	205	96
Catolobus_pendulus	138.174721	35.814408	63	8255	46	581	131
Catolobus_pendulus	141.79012	39.8548	97	7922	42	553	154
Catolobus_pendulus	42.1033	61.0659	25	10046	33	227	83
Catolobus_pendulus	109.5948	55.7949	-31	14980	55	152	28
Catolobus_pendulus	86.8433	54.0925	23	12117	38	185	53
Catolobus_pendulus	57.118371	53.029903	32	11964	29	195	80
Catolobus_pendulus	122.876306	54.04656	-30	16521	95	307	13
Catolobus_pendulus	39.733302	53.912193	56	10111	32	215	77

Catolobus_pendulus	48.393439	54.608593	47	11081	37	194	60
Catolobus_pendulus	41.75	55.7917	51	10185	32	215	77
Catolobus_pendulus	104.886475	51.889524	0	12994	78	367	38
Catolobus_pendulus	37.621722	52.428876	64	9874	29	202	79
Catolobus_pendulus	84.977379	56.447754	10	12586	34	223	69
Catolobus_pendulus	138.756111	36.104722	104	8385	61	578	77
Catolobus_pendulus	105.133333	51.15	-24	13192	88	370	28
Catolobus_pendulus	98.3768	58.4316	-9	14527	48	148	31
Catolobus_pendulus	131.8431	43.0196	63	10753	69	407	45
Catolobus_pendulus	54.776041	58.095014	22	11123	32	217	77
Catolobus_pendulus	138.125	35.935417	80	8403	45	562	125
Catolobus_pendulus	114.886117	60.712441	-42	16563	47	144	33
Catolobus_pendulus	105.316667	51.2	-20	13311	85	378	31
Catolobus_pendulus	131.0848	62.8053	-96	21633	63	136	21
Catolobus_pendulus	10.6302	59.9728	50	7446	27	330	158
Catolobus_pendulus	83.715208	53.672691	23	12799	33	183	61
Catolobus_pendulus	41.935857	55.287174	55	10270	29	202	78
Catolobus_pendulus	86.7756	53.82	24	12133	43	167	42
Catolobus_pendulus	138.4636	36.05	96	8443	55	422	68
Catolobus_pendulus	61.4025	55.1599	30	12019	56	228	43
Catolobus_pendulus	85.6195	55.6684	14	12409	35	199	63
Catolobus_pendulus	98.6619	55.5678	10	12790	67	214	28
Catolobus_pendulus	150.8124	59.6236	-33	11684	55	216	32
Catolobus_pendulus	84.972531	56.489526	10	12595	36	224	67
Catolobus_pendulus	104.759673	52.801979	2	13827	99	207	12
Catolobus_pendulus	49.1889	53.8858	53	11427	25	182	81
Catolobus_pendulus	36.506236	55.849696	50	9458	33	236	85
Catolobus_pendulus	92.8783	55.7608	-7	12091	51	260	51
Catolobus_pendulus	84.7339	56.0741	10	12568	39	206	56
Catolobus_pendulus	138.1	35.4	72	7934	40	719	201
Catolobus_pendulus	131.2123	42.6398	72	10452	85	433	26
Catolobus_pendulus	89.4074	54.4456	-5	11716	29	237	85
Catolobus_pendulus	109.99577	55.936579	-30	15204	46	156	36
Catolobus_pendulus	138.773889	36.177778	125	8496	53	438	74
Catolobus_pendulus	36.459031	55.617213	51	9484	32	232	86

Catolobus_pendulus	94.5527	55.994	13	12493	57	179	31
Catolobus_pendulus	150.713047	59.597346	-38	11631	55	235	35
Catolobus_pendulus	46.107676	54.139746	53	10801	39	205	62
Catolobus_pendulus	49.0086	53.4958	55	11501	24	167	74
Catolobus_pendulus	127.52778	40.66861	25	11466	97	429	21
Catolobus_pendulus	141.39375	40.1375	82	8121	28	564	259
Catolobus_pendulus	83.314772	54.642144	19	12872	41	173	45
Catolobus_pendulus	56.933226	54.251656	29	11612	30	270	109
Catolobus_pendulus	65.3236	56.4846	24	12454	49	199	41
Catolobus_pendulus	87.8915	55.6221	5	11849	38	267	77
Catolobus_pendulus	130.7538	61.8333	-88	20987	60	137	22
Catolobus_pendulus	65.5412	57.1529	19	12385	56	232	41
Catolobus_pendulus	132.8698	42.8341	57	9992	65	443	49
Catolobus_pendulus	85.8285	55.5622	16	12372	39	191	57
Catolobus_pendulus	103.358651	52.982886	7	13386	86	237	21
Catolobus_pendulus	135.401627	44.323882	37	11611	71	376	34
Catolobus_pendulus	41.0833	55.2917	54	10146	28	218	88
Catolobus_pendulus	59.093872	53.459096	28	12031	52	206	47
Catolobus_pendulus	113.33	39.17	86	10752	82	305	16
Catolobus_pendulus	65.3411	55.441	29	12669	47	166	38
Catolobus_pendulus	87.139625	53.751909	29	12003	34	193	62
Catolobus_pendulus	87.60332	51.76909	33	11041	57	308	49
Catolobus_pendulus	100.241944	56.124849	0	13419	63	187	27
Catolobus_pendulus	99.3644	56.0611	4	13224	63	206	30
Catolobus_pendulus	143.158611	42.874722	79	9029	39	439	136
Catolobus_pendulus	101.944199	47.244979	-27	11951	99	236	10
Catolobus_pendulus	40.292482	51.148567	71	10285	25	184	80
Catolobus_pendulus	150.786443	59.568947	-33	11476	56	225	33
Catolobus_pendulus	143.4953	50.6254	-5	10887	40	304	92
Catolobus_pendulus	142.8839	47.1166	14	9079	35	373	125
Catolobus_pendulus	138.125	36.187083	72	8439	52	526	111
Catolobus_pendulus	137.81122	36.19975	96	8557	46	464	126
Catolobus_pendulus	121.625947	63.755758	-80	19240	57	131	26
Catolobus_pendulus	138.5	36.06125	97	8431	49	433	87
Catolobus_pendulus	136.935421	49.504328	18	14782	64	315	42

Catolobus_pendulus	85.77951	51.683464	34	11229	57	256	47
Catolobus_pendulus	64.5704	56.1621	27	12432	48	179	41
Catolobus_pendulus	128.991165	42.085739	43	11835	90	473	28
Catolobus_pendulus	128.927047	36.788428	108	8835	75	720	81
Catolobus_pendulus	130.1323	62.4453	-89	21382	58	129	22
Catolobus_pendulus	48.369346	54.398121	47	11095	38	212	65
Catolobus_pendulus	92.2379	54.3668	17	12525	64	193	24
Catolobus_pendulus	131.4558	42.6575	72	10276	74	433	38
Catolobus_pendulus	105.745827	56.779095	-17	14547	44	221	55
Catolobus_pendulus	127.386424	53.971068	-48	16311	98	469	15
Catolobus_pendulus	108.2052	59.2613	-34	15795	59	162	26
Catolobus_pendulus	128.22	45.04	46	13787	96	408	18
Catolobus_pendulus	107.7055	56.0213	-15	14515	69	229	27
Catolobus_pendulus	127.703291	49.873203	14	15829	95	368	15
Catolobus_pendulus	138.25	36.309583	115	8618	43	378	106
Catolobus_pendulus	128.450861	37.824889	87	9094	69	735	109
Catolobus_pendulus	106.903889	47.9225	-7	13815	118	169	3
Catolobus_pendulus	59.3291	53.9381	21	11841	53	234	52
Catolobus_pendulus	65.5886	55.8238	27	12599	49	180	39
Catolobus_pendulus	127.35948	53.854331	-27	16619	103	386	10
Catolobus_pendulus	138.625	36.06125	79	8320	55	561	89
Catolobus_pendulus	166.008795	60.364921	-13	9559	36	170	67
Catolobus_pendulus	60.270168	57.423939	20	11221	54	234	50
Catolobus_pendulus	89.816667	52.2	-9	11268	64	251	30
Catolobus_pendulus	85.9606	51.9578	36	11416	51	278	61
Catolobus_pendulus	107.657935	51.84483	4	13749	102	152	6
Catolobus_pendulus	96.77639	31.47917	27	6728	97	236	4
Catolobus_pendulus	47.1359	59.6943	23	10532	35	225	73
Catolobus_pendulus	59.976261	55.165939	17	11492	45	267	70
Catolobus_pendulus	81.12	43.15	33	8963	69	242	25
Catolobus_pendulus	48.3472	54.2729	51	11152	38	199	60
Catolobus_pendulus	48.978099	55.769013	43	11091	35	192	69
Catolobus_pendulus	102.497	53.5534	6	13458	72	187	22
Catolobus_pendulus	100.2139	54.1275	8	12578	89	304	24
Catolobus_pendulus	144.469444	42.994167	75	7891	37	451	132

Catolobus_pendulus	138.090668	35.774024	92	8363	39	531	143
Catolobus_pendulus	98.56	28.1	116	5585	51	318	42
Catolobus_pendulus	106.323881	52.150818	6	13615	76	247	25
Catolobus_pendulus	131.9591	62.7548	-98	21769	67	138	19
Catolobus_pendulus	69.131783	61.022838	-7	12832	42	222	62
Catolobus_pendulus	87.256797	53.286122	27	11786	30	255	98
Catolobus_pendulus	142.3873	49.7298	-20	10677	49	368	76
Catolobus_pendulus	120.0293	52.7797	-18	16580	110	255	9
Catolobus_pendulus	128	43	28	12423	97	581	23
Catolobus_pendulus	138.9	36.1	118	8383	59	545	75
Catolobus_pendulus	100.572762	54.557506	3	13083	84	248	21
Catolobus_pendulus	85.963042	55.382573	19	12363	39	178	53
Catolobus_pendulus	129.81937	62.072674	-84	20999	60	121	20
Catolobus_pendulus	115.8877	50.1362	3	15341	116	221	6
Catolobus_pendulus	84.6456	55.7267	12	12597	38	188	52
Catolobus_pendulus	114.637871	45.430744	-6	14040	118	207	6
Catolobus_pendulus	139.126667	35.871389	117	8128	62	709	95
Catolobus_pendulus	41.975503	55.508451	53	10233	30	220	83
Catolobus_pendulus	83.6209	51.7042	19	11687	38	227	70
Catolobus_pendulus	114.533333	51.1	0	14644	113	266	8
Catolobus_pendulus	73.3088	55.0215	18	13158	45	164	42
Catolobus_pendulus	100.989062	51.67751	-31	12606	110	201	5
Catolobus_pendulus	140.91884	40.12647	94	8407	28	433	201
Catolobus_pendulus	83.983385	54.618952	15	12692	47	191	41
Catolobus_pendulus	131.904768	43.162423	65	11034	67	394	44
Catolobus_pendulus	99.009269	54.920164	11	12471	86	238	20
Catolobus_pendulus	150.980402	59.64477	-38	11905	63	204	24
Catolobus_pendulus	138.4375	36.56125	51	8302	41	625	222
Catolobus_pendulus	106.580278	47.878333	-9	13742	115	184	4
Catolobus_pendulus	91.43794	52.79936	8	12194	78	279	23
Catolobus_pendulus	48.8758	54.0311	50	11310	28	199	83
Catolobus_pendulus	134.753333	47.5775	27	14543	76	435	34
Catolobus_pendulus	85.570823	51.587362	23	11144	58	247	43
Catolobus_pendulus	128.594169	37.079217	102	9124	75	692	76
Catolobus_pendulus	137.946389	35.975278	91	8491	48	508	111

Catolobus_pendulus	85.726423	51.348376	-1	10789	69	321	37
Catolobus_pendulus	138.23022	35.86139	85	8364	49	478	96
Catolobus_pendulus	101.2258	53.6469	7	12776	81	255	24
Catolobus_pendulus	56.3896	58.3844	20	11100	32	215	81
Catolobus_pendulus	104.626631	51.472336	16	13042	82	435	39
Catolobus_pendulus	41.75	55.2917	52	10215	29	211	82
Catolobus_pendulus	138.375	36.435417	57	8347	42	649	190
Catolobus_pendulus	94.7729	55.571	11	12263	61	216	33
Catolobus_pendulus	98.974694	55.017922	6	12464	85	238	21
Catolobus_pendulus	90.2827	56.2558	15	12246	48	191	39
Catolobus_pendulus	137.925748	35.294556	123	8043	36	572	175
Catolobus_pendulus	43.6932	51.1909	67	10962	20	151	77
Catolobus_pendulus	62.1342	56.2175	31	11887	52	206	41
Catolobus_pendulus	101.7175	54.225	-2	13523	72	219	23
Catolobus_pendulus	43.367	49.783	79	10906	17	130	80
Catolobus_pendulus	52.2206	54.1201	41	11628	26	184	83
Catolobus_pendulus	110.295	53.7147	-29	14076	88	274	18
Catolobus_pendulus	103.970163	53.33642	3	13821	89	221	17
Catolobus_pendulus	38.9167	56.125	49	9778	34	259	89
Catolobus_pendulus	99.830278	28.626944	42	5715	121	326	3
Catolobus_pendulus	126.878236	54.086228	-50	16138	98	379	12
Catolobus_pendulus	73.323968	55.042836	17	13127	47	178	42
Catolobus_pendulus	138.25	35.85375	100	8416	55	481	78
Catolobus_pendulus	100.998285	51.721082	-76	12176	109	285	8
Catolobus_pendulus	110.01294	55.936035	-28	15245	48	160	36
Catolobus_pendulus	45.4584	59.5304	27	10356	32	244	90
Catolobus_pendulus	85.582526	51.972852	22	11461	52	309	65
Catolobus_pendulus	59.515788	56.791779	19	11174	45	228	58
Catolobus_pendulus	60.351258	56.832358	22	11228	54	240	49
Catolobus_pendulus	141.6673	39.9455	73	7944	34	625	222
Catolobus_pendulus	95.9665	56.5661	4	13111	53	179	34
Catolobus_pendulus	135.5781	45.7988	21	13501	67	374	38
Catolobus_pendulus	138.125	35.435417	26	7808	43	760	191
Catolobus_pendulus	57.734971	55.013558	27	11497	37	269	88
Catolobus_pendulus	98.0029	55.9405	8	12898	54	225	42

Catolobus_pendulus	94.9939	56.1104	4	12575	54	219	39
Catolobus_pendulus	130.351	52.797	-20	17174	90	419	16
Catolobus_pendulus	56.8578	52.5766	33	12097	27	191	84
Catolobus_pendulus	86.190903	55.365717	20	12304	40	178	51
Catolobus_pendulus	41.5123	57.4606	41	10148	30	219	87
Catolobus_pendulus	98.98749	56.90923	-1	13447	55	193	33
Catolobus_pendulus	145.9471	44.1728	50	7044	41	529	127
Catolobus_pendulus	85.03316	56.53915	8	12578	36	230	68
Catolobus_pendulus	107.811224	51.054054	3	13734	104	267	13
Catolobus_pendulus	138.98224	36.02926	134	8362	61	526	70
Catolobus_pendulus	83.064255	51.315147	33	11797	31	190	76
Catolobus_pendulus	59.3199	59.4684	-18	11390	44	320	89
Catolobus_pendulus	140.99425	40.01297	88	8422	33	443	188
Catolobus_pendulus	45.018316	53.195063	55	10817	18	173	97
Catolobus_pendulus	40.661992	52.857239	60	10334	24	201	96
Catolobus_pendulus	88.8103	53.7649	8	11575	25	324	136
Catolobus_pendulus	85.2411	55.9646	12	12478	35	199	61
Catolobus_pendulus	101.5091	54.3378	-2	13495	77	227	21
Catolobus_pendulus	61.0579	56.6014	28	11407	50	230	52
Catolobus_pendulus	138.125	36.687083	102	8469	36	565	231
Catolobus_pendulus	108.2648	57.7261	-18	15206	57	194	32
Catolobus_pendulus	85.526046	51.574306	17	11086	64	271	38
Catolobus_pendulus	127.015	60.8157	-69	18542	65	147	23
Catolobus_pendulus	128.370731	37.1985	115	9400	82	769	74
Catolobus_pendulus	146.8267	64.7436	-130	17663	88	141	14
Catolobus_pendulus	87.4125	56.0917	15	12152	46	205	46
Catolobus_pendulus	143.8214	49.3088	12	10423	47	334	71
Catolobus_pendulus	103.7	51.75	-6	12469	80	407	45
Catolobus_pendulus	113.1998	55.1594	-54	15006	113	302	6
Catolobus_pendulus	63.9143	55.3373	26	12499	45	183	44
Catolobus_pendulus	43.75	51.20833	65	10960	21	166	83
Catolobus_pendulus	90.0679	50.2479	-95	11352	104	133	6
Catolobus_pendulus	53.1578	57.187	30	11289	30	180	72
Catolobus_pendulus	104.371478	52.45166	9	13370	90	274	23
Catolobus_pendulus	137.6279	50.7559	6	15157	69	325	39

Catolobus_pendulus	46.7534	53.9394	42	10795	41	246	72
Catolobus_pendulus	144.166672	43.099998	74	8327	45	480	116
Catolobus_pendulus	64.6026	55.563	25	12520	49	182	40
Catolobus_pendulus	43.318893	54.932109	52	10408	31	205	78
Catolobus_pendulus	85.952543	53.877392	15	12418	44	181	47
Catolobus_pendulus	60.315267	55.016139	28	11713	52	226	48
Catolobus_pendulus	62.579305	56.284685	31	12021	56	194	35
Catolobus_pendulus	138.5	36.642917	44	8247	33	704	302
Catolobus_pendulus	92.9511	55.8603	-5	12124	54	241	44
Catolobus_pendulus	49.5007	53.8094	51	11466	22	177	88
Catolobus_pendulus	69.0254	57.7795	13	12496	50	209	44
Catolobus_pendulus	104.17157	52.34407	12	13230	90	276	22
Catolobus_pendulus	127.316667	40.25	33	11115	100	641	35
Catolobus_pendulus	108.397	62.9524	-64	17307	50	143	30
Catolobus_pendulus	68.4514	58.2986	11	12367	51	228	46
Catolobus_pendulus	57.1277	57.3763	22	11255	36	225	70
Catolobus_pendulus	102.574677	53.568549	5	13506	73	221	26
Catolobus_pendulus	37.643329	55.71741	54	9668	29	236	100
Catolobus_pendulus	43.9476	56.3081	49	10400	27	213	86
Catolobus_pendulus	56.7514	53.0172	31	11932	25	186	89
Catolobus_pendulus	84.2359	51.2046	-42	10759	54	337	61
Catolobus_pendulus	128.301806	37.771278	84	9239	78	887	99
Catolobus_pendulus	64.98729	56.49064	23	12404	48	192	43
Catolobus_pendulus	89.4393	54.5753	10	11913	31	232	80
Catolobus_pendulus	137.8125	35.894583	82	8420	38	581	164
Catolobus_pendulus	137.76477	36.19811	89	8523	50	512	124
Catolobus_pendulus	143.283333	43.216667	67	9331	48	488	116
Catolobus_pendulus	126.961098	40.97917	19	11791	95	589	28
Catolobus_pendulus	86.376617	53.293369	24	12252	36	209	67
Catolobus_pendulus	47.533333	57.766667	36	10709	37	218	68
Catolobus_pendulus	87.9761	55.8317	19	12050	44	217	52
Catolobus_pendulus	127.3769	60.8376	-67	18769	62	141	24
Catolobus_pendulus	138.657778	36.0975	81	8343	56	594	94
Catolobus_pendulus	127.508378	63.923754	-89	20357	61	139	22
Catolobus_pendulus	138.2412	36.76669	112	8461	30	506	218

Catolobus_pendulus	135.5979	44.5579	35	11852	67	332	33
Catolobus_pendulus	92.9231	55.9914	19	12430	59	199	32
Catolobus_pendulus	131.20416	42.59805	68	10342	78	463	32
Catolobus_pendulus	73.391	61.2479	-14	12972	40	224	67
Catolobus_pendulus	135.3894	44.4081	23	11678	65	386	41
Catolobus_pendulus	129.063556	37.102444	91	8516	66	714	114
Catolobus_pendulus	121.619575	63.749865	-80	19238	55	128	26
Catolobus_pendulus	138.751111	36.077222	103	8364	53	501	87
Catolobus_pendulus	132.149317	43.373078	62	11472	67	342	33
Catolobus_pendulus	111.7819	55.9326	-30	15259	92	231	14
Catolobus_pendulus	59.991656	59.776937	9	11748	55	226	52
Catolobus_pendulus	129.016	37.062556	94	8597	74	667	84
Catolobus_pendulus	103.686904	51.706411	18	12697	83	353	33
Catolobus_pendulus	115.581192	39.837842	74	10824	105	348	7
Catolobus_pendulus	128.338662	36.991146	122	9435	82	662	61
Catolobus_pendulus	105.861568	53.979316	-8	14065	92	194	13
Catolobus_pendulus	99.918889	29.075833	48	5848	117	297	2
Catolobus_pendulus	82.951858	51.252171	18	11671	34	237	85
Catolobus_pendulus	102.429983	51.914615	5	12245	90	217	18
Catolobus_pendulus	86.6142	55.5133	13	12129	34	211	72
Catolobus_pendulus	131.4909	43.1025	52	11076	75	444	38
Catolobus_pendulus	62.3696	55.3292	32	12291	55	193	37
Catolobus_pendulus	36.903023	56.039337	49	9505	33	251	89
Catolobus_pendulus	95.9105	56.2268	0	12816	61	211	33
Catolobus_pendulus	139.349167	36.501667	112	8268	50	741	134
Catolobus_pendulus	101.674793	51.694073	-5	12518	96	254	17
Catolobus_pendulus	88.8457	52.8571	18	11351	42	301	74
Catolobus_pendulus	114.529896	45.439899	-10	14012	114	252	9
Catolobus_pendulus	52.192935	55.768693	41	11444	25	174	77
Catolobus_pendulus	35.590845	53.522025	58	9483	30	221	89
Catolobus_pendulus	128.919444	37.158333	77	8617	81	838	87
Catolobus_pendulus	138.625	36.142917	105	8450	54	456	76
Catolobus_pendulus	94.92	29.58	31	5857	74	430	19
Catolobus_pendulus	56.1651	57.9492	26	11215	30	203	82
Catolobus_pendulus	87.187	54.5081	23	12033	28	200	81

Catolobus_pendulus	88.0717	53.689716	29	11732	23	284	132
Catolobus_pendulus	158.6812	57.7619	-10	10659	55	214	38
Catolobus_pendulus	44.547557	48.512319	92	11187	19	111	67
Catolobus_pendulus	124.729233	55.166228	-36	16225	88	337	16
Catolobus_pendulus	137.875	35.85375	103	8489	52	477	95
Catolobus_pendulus	127.03222	41.36611	38	12155	86	577	35
Catolobus_pendulus	53.777024	56.581117	35	11450	28	182	75
Catolobus_pendulus	93.849	55.8833	6	12307	50	220	43
Catolobus_pendulus	88.6433	53.979	7	11620	24	374	167
Catolobus_pendulus	135.530222	62.611636	-99	22454	66	166	23
Catolobus_pendulus	87.2491	53.2878	26	11799	32	256	90
Catolobus_pendulus	56.4674	58.3677	18	11085	31	229	91
Catolobus_pendulus	117.308128	36.036476	104	9612	106	794	34
Catolobus_pendulus	141.790185	39.48207711	96	7873	43	570	143
Catolobus_pendulus	137.2992	58.6584	-75	18328	64	204	29
Catolobus_pendulus	103.203553	52.821971	12	13157	83	228	22
Catolobus_pendulus	103.260892	52.2405	12	12639	92	283	23
Catolobus_pendulus	131.71939	42.965808	67	10757	70	411	44
Catolobus_pendulus	54.575955	58.09169	24	11131	36	220	69
Catolobus_pendulus	128.105069	38.653681	52	9046	68	1160	194
Catolobus_pendulus	87.8467	55.7484	17	12012	42	225	58
Catolobus_pendulus	94.8375	55.23	11	12094	69	233	30
Catolobus_pendulus	138.708333	36.084722	105	8404	58	529	78
Catolobus_pendulus	137.875	36.187083	117	8652	46	384	100
Catolobus_pendulus	133.0734	49.0286	11	15492	83	453	31
Catolobus_pendulus	84.3574	51.5125	2	11234	63	292	42
Catolobus_pendulus	137.75	36.26875	54	8371	52	649	156
Catolobus_pendulus	90.6987	56.4938	10	12292	47	203	45
Catolobus_pendulus	89.896455	55.027297	6	12014	62	244	37
Catolobus_pendulus	138.125	35.727917	56	8178	41	666	170
Catolobus_pendulus	88.441111	52.823611	26	11402	39	275	78
Catolobus_pendulus	139.293611	36.342222	145	8352	59	662	90
Catolobus_pendulus	44.9989	62.2346	21	10503	28	199	80
Catolobus_pendulus	96.2809	58.4979	-13	14212	43	170	42
Catolobus_pendulus	87.641875	51.690685	36	11057	62	251	34

Catolobus_pendulus	86.9547	54.6503	15	12029	32	220	76
Catolobus_pendulus	137.75	35.727917	63	8243	42	801	211
Catolobus_pendulus	129.311228	41.810028	-16	10800	87	583	41
Catolobus_pendulus	132.0836	61.6591	-93	21273	68	135	17
Catolobus_pendulus	86.079615	51.267673	30	10925	72	236	25
Catolobus_pendulus	138.56235	35.959265	78	8310	51	534	98
Catolobus_pendulus	85.935137	51.949222	31	11382	54	316	61
Catolobus_pendulus	88.1	54.733333	2	11689	25	420	190
Catolobus_pendulus	138.991276	35.924856	115	8257	51	535	101
Catolobus_pendulus	104.880646	51.863846	2	12995	74	368	42
Capsella_grandiflora	8.99037	47.23297	101	6549	20	527	307
Capsella_grandiflora	9.11956	47.14119	79	6502	17	575	366
Capsella_grandiflora	21.45	39.666667	104	7014	50	404	78
Capsella_grandiflora	10.024492	45.651371	130	7196	23	323	163
Capsella_grandiflora	20.416587	39.447553	152	6350	65	531	39
Capsella_grandiflora	19.91722	39.59167	173	6036	65	546	34
Capsella_grandiflora	19.8975	39.61306	173	6005	65	540	34
Capsella_grandiflora	21.933333	38.783333	73	6734	57	403	53
Capsella_grandiflora	9.05502	47.1871	88	6519	26	714	373
Capsella_grandiflora	20.718917	38.799055	174	6154	69	432	27
Capsella_grandiflora	20.712782	38.788432	172	6110	74	481	22
Capsella_grandiflora	9.715017	45.701266	127	7192	27	356	156
Capsella_grandiflora	22.32444	39.7425	161	7333	38	189	52
Capsella_grandiflora	9.05366	47.14213	91	6539	26	596	308
Capsella_grandiflora	13.80833	45.60278	139	7041	16	373	227
Capsella_grandiflora	21.216667	39.533333	77	6723	60	523	65
Capsella_grandiflora	8.85953	47.27964	97	6514	19	501	295
Capsella_grandiflora	20.600233	38.632717	164	5885	74	526	22
Capsella_grandiflora	8.98906	47.18801	105	6575	30	500	230
Capsella_grandiflora	20.633333	38.733333	123	5891	72	651	32
Capsella_grandiflora	9.05638	47.23206	74	6462	21	766	446
Capsella_grandiflora	8.6521	46.87719	75	6501	19	565	331
Capsella_grandiflora	20.838198	39.613223	138	6764	58	447	58
Capsella_grandiflora	6.926734	53.353991	97	5331	19	233	142
Capsella_grandiflora	9.18546	47.14021	63	6453	15	614	415

Capsella_grandiflora	21.47889	39.64028	108	7030	54	405	70
Capsella_grandiflora	21.833333	38.95	82	6787	57	427	62
Capsella_grandiflora	19.87216	39.74812	141	5982	67	743	44
Capsella_grandiflora	16.2994	60.64	48	7704	31	242	95
Capsella_grandiflora	10.17499	45.558143	132	7214	25	310	144
Capsella_grandiflora	20.696416	38.825049	166	6094	71	513	30
Capsella_grandiflora	21.65	39.033333	94	6790	60	509	59
Capsella_grandiflora	21.4	38.133333	176	6426	77	418	16
Capsella_grandiflora	9.06048	47.36695	88	6493	19	405	251
Capsella_grandiflora	23.65	38.333333	173	6068	63	217	22
Capsella_grandiflora	9.908174	45.715244	112	7102	27	390	178
Capsella_grandiflora	8.66027	47.23695	98	6500	28	438	217
Capsella_grandiflora	9.155109	47.136239	78	6503	15	544	374
Capsella_grandiflora	10.233702	45.583282	120	7147	26	325	147
Capsella_grandiflora	20.678164	38.756048	155	6036	74	554	27
Capsella_grandiflora	20.61219	38.37117	153	5661	76	575	22
Capsella_grandiflora	21.383333	39.5	87	6853	54	447	67
Capsella_grandiflora	10.099411	45.746567	118	7109	29	368	152
Capsella_grandiflora	20.733333	39.966667	87	6695	48	515	96
Capsella_grandiflora	8.7946	47.3254	96	6497	23	479	261
Capsella_grandiflora	10.671	63.434	57	6469	19	275	149
Capsella_grandiflora	21.15	39.416667	51	6529	57	623	77
Capsella_grandiflora	10.130286	45.709993	80	6946	32	464	175
Capsella_grandiflora	139.478049	35.490757	155	7714	40	571	160
Capsella_grandiflora	10.041648	45.654601	121	7154	24	383	182
Capsella_grandiflora	21.1	39.459999	90	6677	55	553	76
Capsella_grandiflora	20.704566	38.720757	152	5997	76	573	24
Capsella_grandiflora	20.522	38.28686	134	5509	73	595	27
Capsella_grandiflora	9.05231	47.09717	97	6570	24	446	243
Capsella_grandiflora	21.233333	39.483333	72	6682	61	576	67
Capsella_grandiflora	9.758418	45.715191	119	7140	29	386	161
Capsella_grandiflora	8.9231	47.18888	103	6561	31	518	234
Capsella_grandiflora	19.74722	39.70667	162	5874	65	637	40
Capsella_grandiflora	21.2	39.4	79	6642	66	648	58
Capsella_grandiflora	19.879999	39.470001	171	5909	67	573	28

Capsella_grandiflora	10.01937	45.683082	113	7110	26	396	184
Capsella_grandiflora	8.92435	47.23384	102	6546	22	517	283
Capsella_grandiflora	10.080044	45.698499	128	7167	23	338	170
Capsella_grandiflora	9.887974	45.443508	138	7315	20	302	165
Capsella_grandiflora	9.990195	45.691955	110	7100	29	399	165
Capsella_grandiflora	20.983333	38.816667	104	6203	68	639	46
Capsella_grandiflora	23.821484	38.021521	164	5866	65	201	14
Capsella_grandiflora	20.87806	40.03917	111	6903	50	388	71
Capsella_grandiflora	8.65109	46.83222	80	6547	17	369	228
Capsella_grandiflora	20.682129	38.83884	175	6144	68	439	29
Capsella_grandiflora	20.648468	38.627699	165	5925	73	524	24
Capsella_grandiflora	21.433333	38.366667	175	6600	73	412	22
Capsella_grandiflora	8.7263	47.23623	104	6534	23	418	229
Capsella_grandiflora	8.64907	46.74228	47	6453	13	429	301
Capsella_grandiflora	10.05	45.41	138	7312	20	301	162
Capsella_grandiflora	8.53098	47.37317	102	6482	25	341	186
Capsella_orientalis	29.943056	50.052778	79	8794	35	278	109
Capsella_orientalis	92.222013	49.91071	10	14166	97	112	7
Capsella_orientalis	36.0878	45.3337	119	7941	17	120	79
Capsella_orientalis	66.807983	55.277847	25	12716	54	177	33
Capsella_orientalis	83.393186	53.109262	25	12715	33	167	59
Capsella_orientalis	85.1991	54.8994	18	12546	42	184	47
Capsella_orientalis	43.3963	52.4324	56	10737	22	192	93
Capsella_orientalis	85.080177	56.552895	8	12582	37	227	65
Capsella_orientalis	47.426996	54.700393	44	10852	39	213	65
Capsella_orientalis	43.3252	52.4508	57	10733	23	192	90
Capsella_orientalis	64.13528	52.36381	40	13299	33	108	38
Capsella_orientalis	103.290253	53.044792	4	13404	83	226	21
Capsella_orientalis	51.370537	51.204019	66	12474	20	102	55
Capsella_orientalis	45.232958	49.996497	75	11417	17	112	68
Capsella_orientalis	43.6461	49.5031	83	11007	18	124	77
Capsella_orientalis	85.958	55.4706	18	12360	38	179	54
Capsella_orientalis	38.004722	50.088333	81	9945	18	177	97
Capsella_orientalis	93.874053	49.59124	-35	14637	92	169	9
Capsella_orientalis	76.57	52.16	35	13591	45	116	29

Capsella_orientalis	42.870794	52.481885	59	10680	23	190	90
Capsella_orientalis	83.66242	53.24251	26	12722	31	168	61
Capsella_orientalis	58.21719	51.863733	41	12475	31	140	59
Capsella_orientalis	65.8396	57.113598	19	12449	53	231	44
Capsella_orientalis	46.5194	50.0206	79	11760	17	105	61
Capsella_orientalis	79.41226	51.92589	36	13287	38	134	44
Capsella_orientalis	82.95477	51.84568	30	12143	27	194	87
Capsella_orientalis	98.641663	48.723558	-21	12453	108	161	6
Capsella_orientalis	46.0167	49.4214	84	11642	17	106	63
Capsella_orientalis	59.86456	57.00638	19	11161	48	216	52
Capsella_orientalis	70.189	64.009167	-36	12934	47	237	61
Capsella_orientalis	48.345982	53.190933	55	11429	22	175	83
Capsella_orientalis	59.190472	51.271589	48	12816	25	113	54
Capsella_orientalis	86.089722	55.354167	19	12318	38	189	57
Capsella_orientalis	46.708889	50.701389	73	11773	19	107	56
Capsella_orientalis	43.5369	52.6204	54	10718	21	193	93
Capsella_orientalis	96.739125	47.47375	-12	12767	103	116	5
Capsella_orientalis	39.2179	47.0111	104	9630	21	182	107
Capsella_orientalis	85.039753	56.523677	8	12578	36	230	68
Capsella_orientalis	45.4022	50.0853	78	11495	16	110	68
Capsella_orientalis	34.60331	49.608388	85	9503	17	172	96
Capsella_orientalis	82.33885	53.07453	24	12833	38	163	52
Capsella_orientalis	101.43659	47.476665	-10	12185	105	182	6
Capsella_orientalis	83.759189	53.349994	26	12749	31	174	64
Capsella_orientalis	89.195252	48.087253	-45	11554	74	85	10
Capsella_orientalis	70.215854	41.689001	13	8575	55	468	49
Capsella_orientalis	81.228528	53.853162	22	13097	50	148	32
Capsella_orientalis	43.8253	49.0108	79	10947	17	136	88
Capsella_orientalis	31.495212	48.428903	87	8996	30	209	96
Capsella_orientalis	60.620663	56.907552	23	11254	52	236	52
Capsella_orientalis	32.766799	46.122467	113	8493	13	110	80
Capsella_orientalis	43.7639	49.1856	85	11025	19	113	67
Capsella_orientalis	45.5322	48.6994	90	11428	17	107	68
Capsella_orientalis	43.950743	49.149838	84	11052	16	118	77
Capsella_orientalis	61.18979	52.18391	40	12763	43	136	37

Capsella_orientalis	91.008081	46.137679	37	13227	51	43	8
Capsella_orientalis	88.260002	48.349998	-57	11234	63	161	27
Capsella_orientalis	83.412454	53.234259	26	12772	30	159	60
Capsella_orientalis	47.743933	53.158026	56	11319	23	171	80
Capsella_orientalis	30.821556	48.18415	93	8949	29	199	91
Capsella_orientalis	31.496705	48.382087	88	9000	30	204	94
Capsella_orientalis	75.410004	50.470001	29	12316	53	176	40
Capsella_orientalis	80.269997	45.66	82	10681	31	205	84
Capsella_orientalis	32.544034	46.066833	114	8402	15	113	79
Capsella_orientalis	46.345606	53.264131	48	10918	26	208	89
Capsella_orientalis	85.224896	54.921223	17	12533	40	187	50
Capsella_orientalis	103.187599	52.88329	10	13232	82	224	23
Capsella_orientalis	63.63207	53.21448	35	13068	42	140	36
Capsella_orientalis	65.528315	57.130528	19	12385	56	232	41
Capsella_rubella	3.24231	47.7859	109	5791	9	211	157
Capsella_rubella	4.76422	47.9537	106	5981	11	228	157
Capsella_rubella	3.48459	47.0625	110	5909	12	249	177
Capsella_rubella	4.77152	47.3623	94	6017	13	256	177
Capsella_rubella	8.56667	42.36667	161	5060	47	368	56
Capsella_rubella	-1.816	42.981998	115	5092	26	414	182
Capsella_rubella	-3.05	43.01	119	4867	19	292	167
Capsella_rubella	-0.72	39.95	120	5840	33	163	64
Capsella_rubella	22.725528	37.881556	156	6858	60	244	28
Capsella_rubella	-1.796	43.377998	146	4758	19	579	309
Capsella_rubella	8.230583	50.037467	105	6314	16	180	113
Capsella_rubella	4.70237	47.9025	105	5972	11	257	181
Capsella_rubella	2.09254	48.9419	111	5369	8	171	137
Capsella_rubella	2.16574	47.9348	114	5616	9	185	144
Capsella_rubella	22.016306	38.049972	125	6828	72	336	17
Capsella_rubella	4.82092	46.4213	116	6194	14	283	184
Capsella_rubella	-6.7	38.4	162	5942	57	290	13
Capsella_rubella	-2.860472	37.375972	89	6259	48	233	25
Capsella_rubella	4.94043	46.5631	122	6222	13	248	160
Capsella_rubella	8.31702	47.0358	102	6540	20	445	270
Capsella_rubella	11.149001	43.924799	127	6850	22	348	161

Capsella_rubella	3.44715	45.37351	102	6031	22	284	146
Capsella_rubella	3.31418	46.9018	115	5900	11	255	192
Capsella_rubella	11.168726	43.894235	139	6878	24	334	147
Capsella_rubella	5.20129	47.0932	118	6185	14	229	153
Capsella_rubella	0.243576	47.1741	119	5352	12	198	140
Capsella_rubella	4.79289	46.948	118	6152	12	209	143
Capsella_rubella	2.41911	48.7651	114	5485	9	161	128
Capsella_rubella	2.35817	48.93	113	5421	8	175	139
Capsella_rubella	3.06	41.78	165	5482	34	240	74
Capsella_rubella	2.49119	48.6228	113	5535	9	166	130
Capsella_rubella	9.38333	42.5	153	5242	45	283	53
Capsella_rubella	-1.726	43.188	127	4970	20	478	254
Capsella_rubella	11.374771	42.397728	166	5999	39	204	47
Capsella_rubella	3.59407	48.0986	104	5784	9	217	164
Capsella_rubella	0.464918	48.111	114	5307	13	199	135
Capsella_rubella	4.04183	47.7893	108	5918	10	222	159
Capsella_rubella	2.25333	48.0719	112	5609	8	172	137
Capsella_rubella	1.40518	47.2864	120	5577	11	180	134
Capsella_rubella	-7.65555	37.12103	183	4093	73	288	5
Capsella_rubella	4.03267	46.4901	117	6064	12	246	181
Capsella_rubella	1.29277	46.4315	120	5562	11	226	164
Capsella_rubella	-0.052871	48.0733	113	5191	17	236	144
Capsella_rubella	5.24755	47.259	115	6164	14	231	161
Capsella_rubella	2.75424	47.3976	108	5757	11	234	173
Capsella_rubella	22.171472	38.092028	107	6847	68	384	27
Capsella_rubella	-0.020546	48.0793	112	5199	17	253	156
Capsella_rubella	14.723063	36.923768	156	5268	69	202	8
Capsella_rubella	4.48799	47.1362	105	6041	11	238	167
Capsella_rubella	3.29686	48.0013	114	5781	9	185	137
Capsella_rubella	2.40589	47.426	113	5708	10	227	166
Capsella_rubella	2.306694	41.750667	103	5707	19	341	203
Capsella_rubella	-2.228	42.426998	138	5481	27	202	75
Capsella_rubella	4.2386	47.7831	106	5933	12	262	180
Capsella_rubella	11.070076	43.930648	136	6862	23	342	163
Capsella_rubella	3.75535	47.0911	109	5954	14	341	227

Capsella_rubella	-8.60623	41.24116	155	3810	52	538	67
Capsella_rubella	3.44153	47.9943	114	5807	11	212	154
Capsella_rubella	2.91223	46.8237	117	5828	11	206	157
Capsella_rubella	-8.31005	39.44477	164	4869	54	321	22
Capsella_rubella	1.38927	47.321	119	5571	11	198	148
Capsella_rubella	29.316667	41.05	138	6590	43	357	91
Capsella_rubella	4.892222	50.241944	99	5576	9	241	182
Capsella_rubella	12.0819	53.6485	88	6240	20	198	110
Capsella_rubella	2.82181	46.7577	116	5811	11	224	169
Capsella_rubella	7.86861	47.33256	102	6436	11	310	229
Capsella_rubella	2.15264	47.184	120	5702	9	194	149
Capsella_rubella	-0.349551	47.9075	117	5161	17	222	136
Capsella_rubella	10.487928	43.749476	153	6500	34	335	103
Capsella_rubella	5.1784	46.8599	120	6219	13	250	169
Capsella_rubella	0.423934	47.1187	120	5403	12	181	131
Capsella_rubella	11.034756	43.808382	147	6808	31	373	122
Capsella_rubella	2.55	48.9098	110	5460	9	191	148
Capsella_rubella	0.422019	47.9412	113	5329	11	201	147
Capsella_rubella	0.175292	48.0626	115	5257	14	205	135
Capsella_rubella	9.22732	39.44072	151	5114	50	285	36
Capsella_rubella	5.062684	51.565982	103	5352	13	227	156
Capsella_rubella	-5.109	36.672	124	5244	71	383	13
Capsella_rubella	-4.908	36.693	164	5406	72	305	10
Capsella_rubella	4.30642	47.149	100	6000	12	269	181
Capsella_rubella	4.10528	46.8256	112	6039	13	264	180
Capsella_rubella	22.840306	38.331833	101	6610	59	296	33
Capsella_rubella	8.25927	46.92566	99	6554	14	392	271
Capsella_rubella	5.05392	46.5804	122	6241	12	275	188
Capsella_rubella	11.533203	42.588773	144	6256	32	255	79
Capsella_rubella	-0.292269	47.9892	115	5149	18	229	137
Capsella_rubella	2.41458	48.6806	114	5502	8	159	126
Capsella_rubella	5.061666	50.980833	104	5454	13	212	146
Capsella_rubella	-3.5615	36.7726	189	6035	60	207	8
Capsella_rubella	4.69094	46.5703	119	6172	11	241	167
Capsella_rubella	15.98	41.82	120	5794	19	197	115

Capsella_rubella	2.76	42.15	150	5761	21	275	154
Capsella_rubella	11.157338	43.96659	129	6881	23	327	148
Capsella_rubella	4.03709	46.2759	118	6083	13	235	158
Capsella_rubella	-2.62	42.91	120	4967	32	373	130
Capsella_rubella	1.93473	47.3008	119	5661	9	195	150
Capsella_rubella	-8.75089	41.35147	158	3476	51	568	75
Capsella_rubella	1.36	42.49	46	5892	18	272	152
Capsella_rubella	4.24	39.9	173	4698	56	249	23
Capsella_rubella	22.215417	38.637639	85	6789	53	292	46
Capsella_rubella	2.46861	47.9472	114	5665	9	182	140
Capsella_rubella	8.709001	40.328471	138	5148	52	342	43
Capsella_rubella	8.13145	47.19636	91	6441	17	467	309
Capsella_rubella	4.19616	46.759	107	6035	10	312	222
Capsella_rubella	8.463111	39.413528	154	4909	59	292	22
Capsella_rubella	-7.82058	37.30717	157	4327	69	326	8
Capsella_rubella	23.69861	37.96	176	5985	64	173	14
Capsella_rubella	8.39375	47.10453	96	6499	31	617	272
Capsella_rubella	5.09189	47.3393	113	6129	13	209	147
Capsella_rubella	-0.014895	47.7718	115	5260	13	218	149
Capsella_rubella	4.04835	46.5963	116	6060	12	222	166
Capsella_rubella	-0.194944	48.0174	113	5168	19	242	142
Capsella_rubella	13.539199	43.474448	155	6463	14	225	151
Capsella_rubella	-0.133267	47.9645	114	5193	18	239	145
Capsella_rubella	-0.23	40.39	113	5713	37	208	75
Capsella_rubella	-2.63	42.85	123	5011	26	287	125
Capsella_rubella	4.30721	46.4007	112	6102	11	258	187
Capsella_rubella	11.65472	43.65639	84	6658	24	378	165
Capsella_rubella	1.5777	49.0503	111	5189	12	177	123
Capsella_rubella	4.52624	46.5605	116	6136	12	215	150
Capsella_rubella	0.369075	48.2517	113	5230	15	218	140
Capsella_rubella	11.87639	45.39806	143	7259	17	280	164
Capsella_rubella	2.29441	48.7771	112	5460	9	158	127
Capsella_rubella	0.293994	48.3289	110	5200	16	230	146
Capsella_rubella	-5.22	40.849998	126	6491	31	154	41
Capsella_rubella	-0.17639	47.6865	118	5234	16	211	132

Capsella_rubella	0.19535	47.8703	117	5298	13	199	138
Capsella_rubella	0.280529	48.031	115	5283	15	214	139
Capsella_rubella	2.72931	47.945	115	5703	9	187	143
Capsella_rubella	3.03056	47.8872	110	5741	9	206	155
Capsella_rubella	4.62985	46.709	110	6119	11	258	181
Capsella_rubella	7.5705	47.559	111	6406	20	254	141
Capsella_rubella	0.082784	48.3976	108	5089	15	232	150
Capsella_rubella	1.2591	46.8612	121	5559	13	206	148
Capsella_rubella	4.21727	47.1253	100	5988	15	290	187
Capsella_rubella	8.831113	40.311192	128	5166	52	372	47
Capsella_rubella	2.73336	48.4145	112	5615	10	171	130
Capsella_rubella	0.896643	47.4114	119	5480	11	199	145
Capsella_rubella	26.396944	39.991944	146	6803	61	305	31
Capsella_rubella	9.5981	45.0258	140	7349	20	292	164
Capsella_rubella	-2.796	43.330002	144	4571	23	394	182
Capsella_rubella	3.73302	47.1318	109	5944	13	317	215
Capsella_rubella	3.294444	50.828611	106	5154	12	208	151
Capsella_rubella	2.28939	48.8222	110	5437	8	164	128
Capsella_rubella	-1.007	42.988998	69	5336	27	643	265
Capsella_rubella	4.46052	47.4293	101	5995	11	245	175
Capsella_rubella	2.95	39.17	182	4675	53	230	28
Capsella_rubella	139.47379	35.49127	155	7714	40	571	160
Capsella_rubella	6.637778	43.443333	157	5868	43	403	84
Capsella_rubella	0.58136	47.6817	115	5388	11	200	145
Capsella_rubella	-1.619	43.051998	102	5112	18	526	301
Capsella_rubella	-0.84	39.78	141	5948	30	146	67
Capsella_rubella	-2.11	43.27	144	4685	19	538	292
Capsella_rubella	3.08277	48.1454	110	5706	9	197	149
Capsella_rubella	-5.56	36.23	180	4584	73	335	7
Capsella_rubella	4.44855	46.3981	108	6106	10	262	194
Capsella_rubella	4.85709	46.7932	120	6177	13	232	153
Capsella_rubella	13.842773	44.902578	150	6489	25	284	131
Capsella_rubella	2.18879	48.5933	110	5481	9	180	142
Capsella_rubella	-3.068	43.34	146	4568	23	401	188
Capsella_rubella	3.56514	46.7497	117	5964	13	224	164

Capsella_rubella	4.38589	48.9699	109	5777	10	215	152
Capsella_rubella	5.707022	50.891796	104	5583	10	198	147
Capsella_rubella	0.200615	47.9885	116	5276	15	214	138
Capsella_rubella	4.52735	46.6954	110	6102	10	240	175
Capsella_rubella	3.78155	46.5497	115	6006	13	272	196
Capsella_rubella	8.588134	39.341496	149	4935	57	233	18
Capsella_rubella	0.343558	47.1733	117	5373	14	214	143
Capsella_rubella	-0.044322	47.9801	115	5215	17	223	138
Capsella_rubella	3.71403	46.6834	115	5994	12	265	199
Capsella_rubella	3.8733	47.4935	112	5939	11	238	171
Capsella_rubella	-0.031551	47.8678	116	5251	15	222	144
Capsella_rubella	0.21	40.84	129	5847	38	255	78
Capsella_rubella	0.702505	47.4001	119	5440	13	201	138
Capsella_rubella	-2.243	42.211	105	5565	26	194	74
Capsella_rubella	3.34548	47.7046	108	5818	9	216	163
Capsella_rubella	-0.089651	48.357	107	5054	19	253	147
Capsella_rubella	3.17724	48.8253	109	5585	13	182	125
Capsella_rubella	14.54278	40.755	170	5990	44	345	72
Capsella_rubella	-1.473	42.969002	100	5229	30	502	197
Capsella_rubella	8.19679	47.15098	95	6472	21	445	269
Capsella_rubella	14.08417	42.04667	12	5859	28	353	163
Capsella_rubella	2.86082	47.0583	116	5811	10	218	169
Capsella_rubella	1.51	38.98	179	4642	50	181	27
Capsella_rubella	3.38884	48.1116	109	5764	8	214	161
Capsella_rubella	136.6578	34.9521	164	8049	48	695	156
Capsella_rubella	4.42812	47.7816	105	5958	11	228	163
Capsella_rubella	-3.0126	37.9007	138	6749	55	334	20
Capsella_rubella	0.370462	48.357	109	5191	17	227	141
Capsella_rubella	3.63439	47.3612	113	5925	12	251	177
Capsella_rubella	2.81962	48.3871	113	5630	10	183	136
Capsella_rubella	8.872123	39.130988	146	4902	54	219	19
Capsella_rubella	7.969362	44.394651	126	6522	26	240	115
Capsella_rubella	3.16042	46.9879	117	5870	11	217	165
Capsella_rubella	-6.8	38.4	148	5821	56	329	16
Capsella_rubella	4.58553	46.596	111	6127	12	289	202

Capsella_rubella	14.53182	44.96329	143	6627	27	501	219
Capsella_rubella	6.95236	46.25288	104	6696	11	260	192
Capsella_rubella	5.00217	46.5266	122	6241	12	268	184
Capsella_rubella	4.54025	46.4237	114	6139	11	218	159
Capsella_rubella	136.538666	34.920968	160	8048	54	746	147
Capsella_rubella	8.254012	41.045394	171	4900	57	243	22
Capsella_rubella	-0.38	39.5	180	5474	45	212	44
Capsella_rubella	4.37446	47.1467	104	6021	12	240	160
Capsella_rubella	5.006667	50.469722	99	5541	10	238	178
Capsella_rubella	2.82314	47.3276	111	5776	13	236	164
Capsella_rubella	22.95	39.35	168	6759	42	168	38
Capsella_rubella	2.51523	47.0663	118	5761	9	204	158
Capsella_rubella	4.899722	50.299722	99	5555	9	256	193
Capsella_rubella	2.46	41.6	143	5588	23	309	141
Capsella_rubella	7.6047	47.5581	112	6413	20	261	147
Capsella_rubella	4.77211	47.0585	102	6078	14	237	155
Capsella_rubella	4.82857	46.5584	112	6166	12	286	197
Capsella_rubella	-5.39	42.83	93	5048	38	345	82
Capsella_rubella	8.288395	45.23694	137	7213	26	276	121
Capsella_rubella	11.719567	43.790837	125	6865	26	273	114
Capsella_rubella	9.258025	44.452039	121	6471	30	399	139
Capsella_rubella	-8.36624	41.45441	150	4287	50	537	70
Capsella_rubella	4.07156	47.5162	111	5961	11	227	161
Capsella_rubella	-0.009056	48.1236	112	5186	17	255	158
Capsella_rubella	3.39863	47.0431	107	5882	13	290	204
Capsella_rubella	4.95861	47.2593	107	6100	13	204	141
Capsella_rubella	-7.52851	37.97116	173	5327	56	234	9
Capsella_rubella	-3.48	43.25	135	4629	29	390	149
Capsella_rubella	5.35421	47.4397	113	6151	13	234	168
Capsella_rubella	-0.5748	44.8375	138	5152	19	269	147
Capsella_rubella	0.513519	47.1141	120	5419	13	201	140
Capsella_rubella	2.598611	51.095555	107	4809	17	232	142
Capsella_rubella	139.65317	35.58918	157	7735	40	540	152
Capsella_rubella	2.1105	47.1706	119	5694	9	195	150
Capsella_rubella	2.39117	47.7957	114	5671	10	206	156

Capsella_rubella	3.40895	47.2448	107	5871	10	264	194
Capsella_rubella	4.67152	47.3149	94	6011	12	270	190
Capsella_rubella	9.18333	42.55	123	5166	38	299	70
Capsella_rubella	5.57762	48.2018	99	6042	13	250	158
Capsella_rubella	-0.366815	47.8442	117	5169	17	213	131
Capsella_rubella	-2.859	43.096001	128	4779	25	372	172
Capsella_rubella	1.1935	46.7172	122	5546	11	206	156
Capsella_rubella	-0.103469	48.299	111	5082	18	232	138
Capsella_rubella	3.83803	46.7771	112	6001	11	294	215
Capsella_rubella	3.45541	47.4755	111	5870	11	222	161
Capsella_rubella	4.59525	47.4379	102	6014	11	215	153
Capsella_rubella	8.991668	39.537109	169	5162	50	288	37
Capsella_rubella	0.729546	47.9185	112	5383	12	184	130
Capsella_rubella	-4.12	41.59	126	6193	29	139	45
Capsella_rubella	5.37721	47.162	118	6206	14	248	171
Capsella_rubella	5.1788	43.91227	140	6230	37	232	67
Capsella_rubella	5.29552	46.6895	120	6260	11	312	218
Capsella_rubella	3.6428	47.5615	114	5903	10	217	160
Capsella_rubella	-6.37	42.92	81	4724	37	390	97
Capsella_rubella	3.17	42.26	156	5447	32	258	87
Capsella_rubella	-0.66	38.15	188	5302	48	135	16
Capsella_rubella	1.70475	47.9654	113	5539	10	179	138
Capsella_rubella	-9.14435	39.25481	164	3646	58	274	18
Capsella_rubella	-2.51	43.09	123	4755	23	431	213
Capsella_rubella	0.484495	48.0161	112	5321	11	210	152
Capsella_rubella	1.06622	46.8539	120	5520	15	215	147
Capsella_rubella	8.91667	42.26667	71	5078	46	435	74
Capsella_rubella	1.67152	49.0799	111	5223	13	199	137
Capsella_rubella	4.1233	47.5732	107	5949	13	281	193
Capsella_rubella	1.70164	47.8433	115	5563	11	179	135
Capsella_rubella	10.603056	45.5	139	7224	19	301	164
Capsella_rubella	5.1434	46.9687	119	6199	13	234	158
Capsella_rubella	-8.62968	38.02481	162	3820	65	285	8
Capsella_rubella	4.25821	47.3257	99	5975	11	242	168
Capsella_rubella	2.5227	47.3653	106	5714	11	260	187

Capsella_rubella	15.41208	48.36483	92	7225	37	238	89
Capsella_rubella	-0.103279	47.7569	116	5241	13	218	149
Capsella_rubella	4.26329	47.7536	106	5939	11	246	170
Capsella_rubella	0.637686	48.1877	113	5319	14	194	130
Capsella_rubella	1.61786	47.6077	116	5581	10	199	152
Capsella_rubella	-0.131029	48.0433	111	5165	19	240	140
Capsella_rubella	0.302062	47.9832	115	5301	13	211	143
Capsella_rubella	20.16	40.296967	126	6570	60	643	70
Capsella_rubella	-2.4	43.16	128	4722	26	511	216
Capsella_rubella	4.72639	48.1593	103	5937	11	225	157
Capsella_rubella	-0.266086	47.8165	117	5199	16	221	138
Capsella_rubella	0.776823	47.7815	116	5422	11	188	135
Capsella_rubella	8.60754	39.532541	162	5023	57	237	20
Capsella_rubella	8.4579	47.01402	102	6550	30	560	251
Capsella_rubella	4.74988	46.3528	115	6191	14	292	190
Capsella_rubella	-8.53185	39.38874	169	4615	58	306	17
Capsella_rubella	4.02304	47.2233	92	5926	13	352	239
Capsella_rubella	0.071399	47.6219	117	5297	13	200	135
Capsella_rubella	1.21509	46.5445	121	5549	12	233	166
Capsella_rubella	8.26373	47.0165	86	6479	24	538	290
Capsella_rubella	28.139999	36.23	190	5577	100	440	0
Capsella_rubella	0.035754	48.0539	112	5209	17	251	154
Capsella_rubella	4.44649	46.7684	111	6089	14	250	170
Capsella_rubella	4.81437	46.9674	117	6144	13	217	143
Capsella_rubella	4.84759	46.4461	120	6216	14	253	162
Capsella_rubella	3.99407	47.3659	101	5935	11	298	212
Capsella_rubella	5.24759	46.9902	118	6207	13	254	172
Capsella_rubella	3.38283	46.9256	112	5906	11	263	193
Capsella_rubella	2.29961	45.19212	100	5793	9	381	286
Capsella_rubella	0.273181	47.1449	121	5367	13	177	124
Capsella_rubella	6.17139	48.585	102	6112	10	235	170
Capsella_rubella	17.66611	44.3025	37	7037	14	360	229
Capsella_rubella	4.54115	46.7746	112	6105	11	224	160
Capsella_rubella	11.23167	43.88222	123	6830	23	372	163
Capsella_rubella	1.2761	48.0658	111	5456	10	181	136

Capsella_rubella	4.54465	47.4017	101	6012	11	246	176
Capsella_rubella	3.77713	48.7327	109	5711	11	189	131
Capsella_rubella	0.5326	47.4173	117	5399	13	202	140
Capsella_rubella	-1.407	43.166	109	5167	24	494	230
Capsella_rubella	2.35716	48.7951	111	5454	8	171	136
Capsella_rubella	4.16621	47.0481	101	5987	14	281	182
Capsella_rubella	-4.516	42.015	124	5889	28	130	44
Capsella_rubella	-6.19	39.990002	159	6375	51	399	36
Capsella_rubella	0.484874	51.610961	105	4466	12	166	115
Capsella_rubella	2.5256	46.7261	118	5763	11	221	170
Capsella_rubella	3.42428	47.8029	111	5823	9	208	157
Capsella_rubella	0.070543	47.8977	117	5266	15	213	137
Capsella_rubella	4.63166	47.1049	104	6062	11	223	160
Capsella_rubella	4.87601	47.094	109	6115	15	243	154
Capsella_rubella	0.21632	48.0755	113	5262	13	234	159
Capsella_rubella	0.533181	48.0688	114	5325	11	195	142
Capsella_rubella	0.087614	47.9298	116	5266	16	217	137
Capsella_rubella	2.30433	47.508	114	5692	10	221	163
Capsella_rubella	-0.408414	47.8297	118	5166	17	203	124
Capsella_rubella	-5.57444	31.57194	170	7242	48	101	15
Capsella_rubella	10.258253	44.778504	140	7300	23	316	142
Capsella_rubella	4.52762	46.4955	115	6139	11	216	155
Capsella_rubella	3.84337	48.0138	113	5863	10	211	154
Capsella_rubella	8.987392	44.701969	119	6700	30	371	125
Capsella_rubella	4.308333	50.348888	98	5428	10	261	199
Capsella_rubella	11.52545	42.82074	104	6307	30	304	105
Capsella_rubella	4.08284	47.7038	105	5919	10	261	189
Capsella_rubella	3.66584	47.3326	114	5932	11	230	165
Capsella_rubella	0.453769	47.3514	118	5389	15	200	131
Capsella_rubella	-0.83	39.96	98	5857	33	176	73
Capsella_rubella	1.99541	47.2516	117	5664	10	221	168
Capsella_rubella	3.62186	47.773	115	5869	10	197	145
Capsella_rubella	2.33756	46.8721	119	5737	10	210	166
Capsella_rubella	5.227777	50.155	95	5657	9	282	216
Capsella_rubella	3.029999	51.276111	107	4848	17	238	144

Capsella_rubella	22.15	38.683333	52	6668	53	338	56
Capsella_rubella	9.22596	40.240022	134	5266	51	370	52
Capsella_rubella	4.56793	46.8238	114	6113	12	210	148
Capsella_rubella	3.82772	45.19125	84	6070	19	264	156
Capsella_rubella	3.45101	47.293	111	5885	11	219	159
Capsella_rubella	4.22999	47.3623	99	5965	11	248	177
Capsella_rubella	3.10162	47.2345	114	5844	10	237	178
Capsella_rubella	4.89885	45.82043	124	6317	18	288	180
Capsella_rubella	4.02257	48.7198	108	5755	10	214	152
Capsella_rubella	24.466667	38.85	171	5719	71	220	16
Capsella_rubella	2.81027	48.2552	113	5655	10	175	135
Capsella_rubella	-0.06	38.72	156	4872	50	265	38
Capsella_rubella	4.46059	47.2209	100	6016	13	259	174
Capsella_rubella	13.039945	43.125523	126	6686	16	253	154
Capsella_rubella	-0.043274	48.0383	115	5205	17	220	134
Capsella_rubella	4.02268	47.9069	105	5882	11	257	180
Capsella_rubella	1.03402	47.3373	118	5502	13	216	151
Capsella_rubella	-2.638	42.997002	118	4881	29	351	139
Capsella_rubella	7.01685	46.29812	74	6588	11	381	283
Capsella_rubella	4.69461	46.5375	119	6175	11	230	161
Capsella_rubella	5.809167	45.910833	120	6530	15	299	196
Capsella_rubella	4.96177	46.5778	121	6225	13	277	182
Capsella_rubella	3.48122	47.2327	108	5891	10	258	193
Capsella_rubella	-3.43	43.42	147	4454	32	488	169
Capsella_rubella	23.9	35.35	120	4827	83	516	6
Capsella_rubella	4.08934	47.7722	111	5932	10	198	142
Capsella_rubella	20.71944	38.36639	170	5806	76	525	21
Capsella_rubella	19.916667	39.616667	173	6036	65	546	34
Capsella_rubella	8.00128	43.992	122	5836	33	370	108
Capsella_rubella	0.387197	47.1157	120	5392	11	187	137
Capsella_rubella	10.428853	43.431197	147	6146	39	415	98
Capsella_rubella	4.49653	47.4071	101	6001	11	264	186
Capsella_rubella	3.74465	47.1991	111	5947	11	277	197
Capsella_rubella	2.08911	46.8812	119	5695	9	197	158
Capsella_rubella	4.65216	46.4686	113	6150	11	250	179

Capsella_rubella	-3.388	43.266998	128	4637	28	466	184
Capsella_rubella	8.83138	46.20038	95	6843	38	565	168
Capsella_rubella	-9.36426	39.08302	165	3275	58	277	18
Capsella_rubella	11.255868	43.783279	150	6871	26	298	117
Capsella_rubella	8.258121	44.669654	128	6822	28	292	121
Capsella_rubella	9.13333	42.26667	99	5161	49	393	62
Capsella_rubella	3.61605	47.3494	113	5917	11	214	155
Capsella_rubella	4.73564	46.5552	118	6176	11	246	171
Capsella_rubella	10.477512	43.943494	138	6675	34	336	107
Capsella_rubella	-9.00032	39.23496	156	3853	56	327	22
Capsella_rubella	4.63006	46.5857	115	6148	14	250	163
Capsella_rubella	-0.014986	48.3759	109	5065	17	225	138
Capsella_rubella	3.93948	47.3508	101	5930	11	293	209
Capsella_rubella	6.46991	48.14266	97	6168	12	368	255
Capsella_rubella	9.392171	39.341762	149	5063	52	219	22
Capsella_rubella	3.53779	46.8052	117	5952	12	238	176
Capsella_rubella	1.74865	47.2986	119	5629	10	201	152
Capsella_rubella	-0.106401	47.8616	117	5233	15	217	139
Capsella_rubella	4.76474	46.6236	120	6183	12	234	161
Capsella_rubella	4.07964	47.4929	107	5953	10	249	180
Capsella_rubella	4.22514	47.6993	110	5957	10	199	143
Capsella_rubella	4.17188	47.8442	107	5916	11	247	170
Capsella_rubella	4.05009	47.6189	105	5929	12	281	196
Capsella_rubella	0.128845	47.2124	121	5336	12	182	129
Capsella_rubella	13.208614	42.723192	89	6482	23	286	145
Capsella_rubella	9.107989	44.57928	92	6446	28	416	155
Capsella_rubella	9.18333	42.38333	142	5219	43	284	56
Capsella_rubella	-5.85	43.349998	140	4419	29	371	147
Capsella_rubella	3.61627	47.1493	110	5927	12	271	190
Capsella_rubella	-2.271	41.851	91	5773	23	204	90
Capsella_rubella	-7.77556	41.14066	152	5156	47	330	49
Capsella_rubella	1.85945	47.8819	115	5582	10	183	141
Capsella_rubella	4.15753	47.2768	91	5932	12	298	203
Capsella_rubella	1.66505	47.2564	120	5619	11	196	148
Capsella_rubella	6.9068	46.4263	109	6638	11	336	244

Capsella_rubella	5.0091	46.9455	119	6185	13	234	156
Capsella_rubella	2.39542	48.8351	111	5449	8	183	146
Capsella_rubella	3.96503	47.0051	94	5943	11	341	238
Capsella_rubella	3.2511	47.4149	106	5832	9	258	195
Capsella_rubella	0.909644	48.0634	109	5385	11	199	143
Capsella_rubella	2.61524	46.9053	118	5776	10	214	168
Capsella_rubella	4.61425	46.8909	107	6087	14	250	167
Capsella_rubella	8.83023	46.1554	123	6970	33	496	186
Capsella_rubella	136.6041	34.8691	165	7989	49	702	146
Capsella_rubella	4.98164	47.5822	101	6041	13	248	164
Capsella_rubella	5.16615	47.1315	117	6171	14	222	149
Capsella_rubella	-0.219694	47.8819	117	5194	17	218	135
Capsella_rubella	2.88083	49.0176	108	5475	11	189	136
Capsella_rubella	-6.71104	41.34089	128	5648	44	295	44
Capsella_rubella	3.99818	48.4076	112	5819	10	179	132
Capsella_rubella	3.91837	47.5726	107	5924	13	277	190
Capsella_rubella	1.85095	48.9643	112	5302	11	174	128
Capsella_rubella	0.60375	47.3432	119	5425	10	191	143
Capsella_rubella	139.653034	35.474095	159	7618	40	537	153
Capsella_rubella	8.85	42.51667	153	5141	43	299	55
Capsella_rubella	1.86142	48.5335	107	5441	8	169	135
Capsella_rubella	8.025636	44.691542	138	6902	27	236	108
Capsella_rubella	4.55272	47.3033	96	6003	11	272	194
Capsella_rubella	2.25928	47.9364	113	5632	9	191	149
Capsella_rubella	0.316928	48.2573	112	5218	16	231	147
Capsella_rubella	2.00081	47.197	119	5682	10	185	141
Capsella_rubella	2.38768	48.887	111	5437	8	181	145
Capsella_rubella	0.926792	46.7333	122	5504	11	199	148
Capsella_rubella	-2.36	38.98	134	6705	33	156	44
Capsella_rubella	-6.24	43	64	4661	38	466	116
Capsella_rubella	0.433878	48.1288	111	5281	14	230	157
Capsella_rubella	0.182757	48.1531	115	5234	14	206	136
Capsella_rubella	11.4153	53.5485	88	6173	18	212	128
Capsella_rubella	4.57145	46.6523	114	6130	13	231	154
Capsella_rubella	-8.79332	39.02937	170	4138	59	298	16

Capsella_rubella	4.9494	47.1378	109	6117	14	224	147
Capsella_rubella	3.04048	47.8336	110	5747	9	211	159
Capsella_rubella	4.42361	46.8094	107	6072	16	264	172
Capsella_rubella	0.552613	47.0384	122	5434	13	179	126
Capsella_rubella	4.75383	47.4852	96	6008	12	233	164
Capsella_rubella	-5.27	43.02	82	4808	39	416	97
Capsella_rubella	-3.41	43.41	143	4508	27	478	195
Capsella_rubella	-0.41	38.69	146	5214	49	239	28
Capsella_rubella	1.68882	48.9977	110	5257	11	165	122
Capsella_rubella	4.08081	47.2359	92	5930	13	327	223
Capsella_rubella	22.42111	36.65917	146	5678	85	597	14
Capsella_rubella	4.52978	47.0235	105	6056	11	267	187
Capsella_rubella	1.70122	46.8057	120	5625	10	206	164
Capsella_rubella	4.13559	48.8455	109	5754	11	187	133
Capsella_rubella	2.28588	48.7902	108	5444	9	168	134
Capsella_rubella	2.21365	46.5597	115	5706	9	219	170
Capsella_rubella	8.65	42.25	164	5109	49	350	50
Capsella_rubella	3.02419	47.3996	112	5809	11	256	185
Capsella_rubella	5.23476	46.5503	121	6267	12	306	213
Capsella_rubella	11.145667	42.411119	150	5793	38	232	54
Capsella_rubella	2.07451	48.7762	106	5402	8	177	140
Capsella_rubella	0.934218	46.6373	122	5502	13	218	153
Capsella_rubella	2.28967	48.912	113	5419	8	165	134
Capsella_rubella	10.29	47.72	78	6580	21	371	220
Capsella_rubella	-5.2	40.86	125	6501	31	160	42
Capsella_rubella	6.44472	48.22722	101	6174	12	307	215
Capsella_rubella	0.9525	46.9925	118	5494	14	216	151
Capsella_rubella	1.86859	46.9082	119	5652	10	199	158
Capsella_rubella	2.78962	47.5957	114	5754	11	205	149
Capsella_rubella	4.99523	47.1407	115	6142	13	211	141
Capsella_rubella	3.57847	47.2312	111	5916	10	241	177
Capsella_rubella	-4.327	36.719	184	5717	64	280	15
Capsella_rubella	8.6572	47.10204	79	6457	22	586	322
Capsella_rubella	4.74071	47.0935	96	6043	12	266	191
Capsella_rubella	4.68088	47.3847	98	6014	12	242	174

Capsella_rubella	-1.592	43.141998	133	5089	25	474	205
Capsella_rubella	4.90588	46.6019	120	6205	13	232	153
Capsella_rubella	5.329444	51.162222	102	5468	12	232	166
Capsella_rubella	4.50207	47.0842	104	6046	11	262	184
Capsella_rubella	4.356667	50.851666	105	5334	11	224	163
Capsella_rubella	5.16276	46.6551	121	6243	13	282	188
Capsella_rubella	-2.074	42.993999	88	4944	30	623	235
Capsella_rubella	3.82243	46.7994	115	5999	11	279	202
Capsella_rubella	0.039452	47.6688	118	5283	13	199	136
Capsella_rubella	0.584953	48.0398	112	5344	10	199	148
Capsella_rubella	11.26268	43.88151	125	6847	27	379	142
Capsella_rubella	3.23196	47.189	112	5860	11	232	168
Capsella_rubella	-5.63	42.92	82	4879	38	357	89
Capsella_rubella	7.997189	44.364893	119	6439	28	296	119
Capsella_rubella	-1.289	42.551998	131	5666	21	228	110
Capsella_rubella	2.71686	48.254	113	5642	10	183	142
Capsella_rubella	3.75494	47.2468	109	5943	11	312	221
Capsella_rubella	4.26985	47.4757	105	5977	10	243	179
Capsella_rubella	4.59319	47.4634	102	6007	11	231	165
Capsella_rubella	0.334944	47.5336	115	5359	12	211	148
Capsella_rubella	4.49715	46.8004	108	6081	15	260	172
Capsella_rubella	-0.086798	47.9602	114	5203	17	238	145
Capsella_rubella	2.53844	48.9585	110	5430	9	191	146
Capsella_rubella	5.829719	51.835258	102	5461	12	225	159
Capsella_rubella	3.86918	46.7037	113	6009	11	262	197
Capsella_rubella	4.48668	46.3857	108	6114	10	247	184
Capsella_rubella	4.35317	46.386	111	6104	10	262	192
Capsella_rubella	3.95957	47.096	95	5940	14	394	264
Capsella_rubella	4.365277	51.084444	105	5268	13	229	161
Capsella_rubella	9.00089	47.59267	100	6511	21	322	188
Capsella_rubella	4.72988	47.2915	99	6037	12	230	165
Capsella_rubella	-0.28	38.66	112	5000	49	276	38
Capsella_rubella	4.84962	46.5108	116	6190	14	275	175
Capsella_rubella	11.425273	44.149328	119	6960	23	304	136
Capsella_rubella	-0.181112	47.9818	114	5181	18	239	143

Capsella_rubella	4.371666	51.334722	105	5189	15	232	155
Capsella_rubella	-0.100362	48.0524	113	5179	19	233	137
Capsella_rubella	3.23812	47.0525	113	5873	11	252	185
Capsella_rubella	5.41016	47.4436	114	6158	13	239	172
Capsella_rubella	2.75015	48.319	114	5644	10	172	132
Capsella_rubella	5.798584	53.183483	98	5055	20	243	138
Capsella_rubella	10.26	50.04	95	6592	17	194	118
Capsella_rubella	3.5443	47.4475	113	5901	10	227	167
Capsella_rubella	7.99425	43.97305	136	5884	32	286	90
Capsella_rubella	3.35006	48.0166	110	5774	10	234	171
Capsella_rubella	9.745816	44.096139	142	6488	35	419	118
Capsella_rubella	0.36167	47.292	119	5374	15	195	127
Capsella_rubella	8.886871	44.717228	131	6800	27	326	126
Capsella_rubella	1.75153	47.6223	115	5605	11	206	154
Capsella_rubella	1.49328	47.6526	117	5561	10	184	140
Capsella_rubella	-0.6	40.04	119	5771	36	201	68
Capsella_rubella	0.075601	47.2035	118	5311	12	194	139
Capsella_rubella	-5.4	43.1	67	4715	33	494	161
Capsella_rubella	4.3152	46.7714	108	6059	15	267	175
Capsella_rubella	2.7	42.1	138	5783	18	302	182
Capsella_rubella	4.55656	46.4768	117	6152	10	226	165
Capsella_rubella	5.86811	49.15342	102	6005	11	236	165
Capsella_rubella	4.35056	47.4988	107	5993	10	222	161
Capsella_rubella	13.55	42.51667	89	6324	16	270	176
Capsella_rubella	3.39425	47.4551	110	5864	10	220	162
Capsella_rubella	8.18869	47.03322	86	6472	28	579	296
Capsella_rubella	8.041949	44.305592	118	6352	25	256	106
Capsella_rubella	1.62345	47.7116	117	5570	11	181	137
Capsella_rubella	4.45336	47.0627	105	6044	13	273	183
Capsella_rubella	3.94849	47.198	96	5929	15	391	256
Capsella_rubella	3.224444	51.225277	107	4921	16	234	146
Capsella_rubella	-7.4731	37.84642	171	5280	57	256	9
Capsella_rubella	-8.36009	39.8065	149	4723	56	428	29
Capsella_rubella	3.54184	48.097	105	5780	8	210	160
Capsella_rubella	14.461983	37.73385	141	5607	51	216	31

Capsella_rubella	-1.666	43.116001	122	5084	18	479	277
Capsella_rubella	4.54476	46.6171	117	6140	12	232	157
Capsella_rubella	4.52391	46.9364	104	6062	12	231	162
Capsella_rubella	-6.02	42.46	109	5257	39	267	61
Capsella_rubella	4.39258	46.7064	112	6092	10	254	186
Capsella_rubella	-6.24	42.91	70	4775	32	358	111
Capsella_rubella	4.07099	46.3701	117	6081	13	260	182
Capsella_rubella	2.70711	47.6125	112	5735	10	204	150
Capsella_rubella	5.33297	47.1464	118	6199	14	233	161
Capsella_rubella	4.47816	46.8999	102	6052	13	236	159
Capsella_rubella	4.62108	46.3386	109	6142	10	261	193
Capsella_rubella	5.22281	46.6666	121	6248	13	298	201
Capsella_rubella	1.33984	47.2528	119	5566	11	179	133
Capsella_rubella	1.36208	47.0695	117	5563	10	205	158
Capsella_rubella	-4.9691	40.398	98	6603	48	369	45
Capsella_rubella	4.78974	46.5556	119	6185	12	238	167
Capsella_rubella	11.018077	43.113089	123	6233	32	328	103
Capsella_rubella	139.61119	35.59716	157	7762	40	535	152
Capsella_rubella	1.93251	48.8444	109	5358	9	179	139
Capsella_rubella	0.003543	47.7135	116	5275	16	226	141
Capsella_rubella	4.59306	46.7643	107	6097	12	273	192
Capsella_rubella	0.009602	48.2586	113	5142	15	212	137
Capsella_rubella	11.546825	44.108831	111	6918	23	320	146
Capsella_rubella	2.89821	48.802	110	5536	12	174	127
Capsella_rubella	0.247303	48.3426	109	5158	18	234	142
Capsella_rubella	-1.531	43.132	133	5137	27	455	189
Capsella_rubella	2.57588	46.6337	119	5775	12	204	148
Capsella_rubella	4.88724	47.0276	117	6154	13	216	144
Capsella_rubella	0.296681	48.3555	108	5179	17	238	148
Capsella_rubella	2.05393	47.871	116	5621	9	185	145
Capsella_rubella	0.819227	47.0428	120	5474	15	207	141
Capsella_rubella	5.23792	46.6354	121	6257	12	292	201
Capsella_rubella	35.890617	32.277233	175	6256	115	257	0
Capsella_rubella	4.46	51.315277	104	5245	14	233	158
Capsella_rubella	11.189327	43.969887	120	6854	27	411	156

Capsella_rubella	0.071005	47.7085	116	5283	16	229	146
Capsella_rubella	0.0115	42.7314	24	5590	14	388	273
Capsella_rubella	1.2884	47.3098	118	5548	10	182	138
Capsella_rubella	2.25396	47.3073	116	5706	10	214	158
Capsella_rubella	-1.169	42.902	90	5378	22	452	225
Capsella_rubella	0.844099	47.3861	119	5474	12	196	141
Capsella_rubella	4.26143	47.8164	105	5929	11	236	164
Capsella_rubella	11.341209	43.636776	125	6738	24	354	150
Capsella_rubella	8.25995	46.97063	61	6398	15	561	374
Capsella_rubella	11.082728	42.656241	162	5995	33	217	63
Capsella_rubella	-6.6	38.5	153	5965	53	265	15
Capsella_rubella	2.7722	47.9767	114	5702	9	194	147
Capsella_rubella	1.69294	47.6662	117	5592	10	186	142
Capsella_rubella	5.26574	47.1998	117	6184	14	225	154
Capsella_rubella	1.41584	46.7499	121	5579	11	210	159
Capsella_rubella	10.26	49.96	96	6602	18	188	112
Capsella_rubella	5.832222	50.590277	92	5643	9	297	226
Capsella_rubella	2.69	39.58	179	4771	51	233	31
Capsella_rubella	0.045474	48.1025	111	5207	16	237	151
Capsella_rubella	4.06701	46.2484	115	6084	13	279	190
Capsella_rubella	4.71414	46.7582	115	6147	14	233	149
Capsella_rubella	4.03993	46.3485	118	6077	13	243	167
Capsella_rubella	4.61412	47.3489	97	6004	11	266	191
Capsella_rubella	-2.36	40.06	119	6807	34	240	58
Capsella_rubella	0.407084	47.1557	120	5392	14	192	129
Capsella_rubella	4.53366	47.3289	102	6018	11	239	168
Capsella_rubella	0.408659	48.0889	114	5290	14	217	146
Capsella_rubella	6.88661	46.34254	111	6677	12	266	194
Capsella_rubella	7.884748	44.71597	130	6922	35	298	95
Capsella_rubella	4.50764	47.4386	103	6005	11	238	168
Capsella_rubella	-2.4	43.12	128	4768	25	474	216
Capsella_rubella	3.42947	47.9197	115	5817	9	193	144
Capsella_rubella	6.22899	52.237554	100	5505	15	240	157
Capsella_rubella	2.12859	48.8267	107	5406	8	178	144
Capsella_rubella	3.83793	46.6374	110	6001	12	266	199

Capsella_rubella	3.39192	47.1996	110	5884	11	247	178
Capsella_rubella	4.01623	47.5182	110	5951	11	230	165
Capsella_rubella	4.25931	46.2109	113	6110	12	239	171
Capsella_rubella	7.919383	49.978433	104	6268	14	150	99
Capsella_rubella	5.26348	47.2382	116	6176	14	232	161
Capsella_rubella	12.66788	38.106331	176	4970	56	251	19
Capsella_rubella	2.72418	46.8101	117	5799	11	206	159
Capsella_rubella	0.363767	48.3793	107	5170	16	224	141
Capsella_rubella	8.111117	44.243399	106	6171	30	296	93
Capsella_rubella	4.332222	51.267222	105	5203	14	224	153
Capsella_rubella	1.22	42.44	54	5906	19	337	186
Capsella_rubella	4.07566	47.5808	108	5948	13	271	185
Capsella_rubella	9.590089	39.710189	160	5201	50	290	38
Capsella_rubella	4.09544	47.3987	102	5953	10	253	183
Capsella_rubella	8.782701	40.577411	149	5147	52	272	32
Capsella_rubella	4.478627	51.922463	103	5065	19	253	150
Capsella_rubella	8.294458	44.663663	134	6783	29	260	101
Capsella_rubella	2.26099	46.7199	118	5719	10	214	168
Capsella_rubella	10.557218	43.707474	152	6504	37	395	109
Capsella_rubella	-1.58	43.141998	130	5110	27	484	198
Capsella_rubella	4.3271	47.1688	99	5995	13	262	175
Capsella_rubella	-2.57	42.86	121	5012	28	329	133
Capsella_rubella	1.01734	46.597	122	5518	13	218	151
Capsella_rubella	3.83958	47.5513	107	5913	11	280	198
Capsella_rubella	-2.785	43.140999	141	4737	22	324	159
Capsella_rubella	-2.82	42.95	112	4921	28	326	134
Capsella_rubella	4.08554	46.2756	109	6065	13	315	217
Capsella_rubella	-0.263556	47.9525	115	5172	17	233	145
Capsella_rubella	2.13385	47.8543	115	5631	9	193	150
Capsella_rubella	0.334447	48.1427	113	5268	13	220	151
Capsella_rubella	-6.8	40	158	6045	55	358	27
Capsella_rubella	4.80807	46.8347	118	6162	13	229	150
Capsella_rubella	4.874444	50.325277	99	5548	8	222	172
Capsella_rubella	0.792312	48.1736	107	5338	12	220	156
Capsella_rubella	0.287602	47.8277	115	5318	14	219	148

Capsella_rubella	11.66666	42.76637	112	6389	32	306	98
Capsella_rubella	7.99949	47.19708	99	6460	16	309	207
Capsella_rubella	0.234051	48.3145	111	5180	16	237	150
Capsella_rubella	4.23688	47.6216	105	5952	11	251	178
Capsella_rubella	5.60036	48.1567	99	6050	13	300	190
Capsella_rubella	13.121514	43.023641	108	6603	12	238	175
Capsella_rubella	2.209	47.0662	120	5718	9	196	154
Capsella_rubella	14.25139	40.87083	170	5846	51	378	60
Capsella_rubella	5.13147	47.3012	115	6144	14	219	153
Capsella_rubella	2.7	41.78	160	5669	23	260	124
Capsella_rubella	9.69222	37.13806	188	5181	63	283	16
Capsella_rubella	3.64637	48.1505	106	5790	9	196	149
Capsella_rubella	4.56107	47.1029	102	6045	11	261	185
Capsella_rubella	-4.11	38.3	154	6929	46	194	22
Capsella_rubella	4.761182	52.508521	102	4948	23	263	138
Capsella_rubella	4.65714	47.5697	99	5993	11	257	183
Capsella_rubella	4.308888	50.321944	99	5439	10	250	190
Capsella_rubella	3.24547	48.2889	114	5725	9	162	121
Capsella_rubella	4.853679	50.351056	99	5536	9	225	171
Capsella_rubella	4.37489	46.2652	104	6096	11	272	196
Capsella_rubella	9.4	41.86667	165	5263	50	298	44
Capsella_rubella	4.55096	46.7245	108	6097	12	273	190
Capsella_rubella	-0.42	38.6	130	5216	43	200	34
Capsella_rubella	-0.277442	47.8433	116	5190	16	227	144
Capsella_rubella	-5	43.25	86	4422	28	560	222
Capsella_rubella	3.4191	47.5596	109	5854	11	239	172
Capsella_rubella	8.82081	39.113935	148	4874	57	241	18
Capsella_rubella	0.789645	47.2954	118	5457	13	209	148
Capsella_rubella	11.6197	44.838	146	7305	16	218	135
Capsella_rubella	0.014556	48.1835	113	5173	17	224	137
Capsella_rubella	-6.80894	41.09231	139	5738	45	253	32
Capsella_rubella	1.30722	48.432	108	5369	9	174	132
Capsella_rubella	-7.91844	37.44064	161	4422	67	318	7
Capsella_rubella	8.786415	39.246273	159	4942	54	252	23
Capsella_rubella	-4.373	36.773	167	5737	64	303	16

Capsella_rubella	9.2992	44.8075	85	6860	28	447	170
Capsella_rubella	2.38733	48.8143	113	5466	8	158	127
Capsella_rubella	1.30437	47.3972	118	5552	11	197	146
Capsella_rubella	5.29353	47.2352	115	6178	14	249	174
Capsella_rubella	2.43826	48.8037	113	5473	8	170	135
Capsella_rubella	1.66406	49.0685	112	5214	13	180	124
Capsella_rubella	4.5769	46.5793	117	6146	11	230	162
Capsella_rubella	4.78729	47.1773	92	6027	12	278	198
Capsella_rubella	4.39472	46.418	111	6109	10	251	189
Capsella_rubella	139.56657	35.61341	155	7786	41	570	157
Capsella_rubella	4.0925	47.4574	103	5952	10	272	196
Capsella_rubella	2.35892	48.7265	111	5476	8	174	141
Capsella_rubella	4.58903	47.3986	97	6000	11	252	182
Capsella_rubella	4.73341	47.0641	97	6053	12	261	182
Capsella_rubella	2.20676	46.6108	117	5703	9	215	172
Capsella_rubella	1.98	42.36	53	5893	24	323	152
Capsella_rubella	-6.70254	41.52738	142	5654	45	240	34
Capsella_rubella	-0.07	41.38	150	6301	27	141	71
Capsella_rubella	5.388611	50.483333	92	5595	9	276	215
Capsella_rubella	7.932446	44.781682	131	6960	34	280	93
Capsella_rubella	0.2763	40.6347	148	5702	36	206	74
Capsella_rubella	-0.227553	47.9844	114	5170	18	250	149
Capsella_rubella	12.464093	37.906265	186	4925	59	218	14
Capsella_rubella	4.944722	50.320555	95	5551	9	282	215
Capsella_rubella	2.53278	48.9394	112	5446	8	179	141
Capsella_rubella	-2.414	43.32	143	4557	15	441	271
Capsella_rubella	11.310509	42.675814	136	6163	32	270	84
Capsella_rubella	8.848034	39.104909	147	4881	55	229	18
Capsella_rubella	5.10978	46.7969	119	6210	13	245	167
Capsella_rubella	13.210392	38.025393	132	5100	57	299	26
Capsella_rubella	-0.313162	47.7684	118	5194	15	215	136
Capsella_rubella	4.83007	47.0312	113	6127	15	225	143
Capsella_rubella	1.16603	47.5212	117	5513	12	198	139
Capsella_rubella	-1.3333	46.6667	121	4814	26	298	138
Capsella_rubella	11.164831	44.001953	128	6899	25	312	130

Capsella_rubella	15.052664	38.137156	181	5477	51	235	41
Capsella_rubella	1.28745	47.3552	118	5547	11	193	142
Capsella_rubella	2.98278	47.1841	116	5828	10	216	161
Capsella_rubella	-2.83	38.71	136	6802	41	211	34
Capsella_rubella	-5.78	39.900002	169	6648	52	352	30
Capsella_rubella	0.194494	47.6958	116	5316	14	224	149
Capsella_rubella	0.221537	47.4403	117	5339	13	199	136
Capsella_rubella	4.48718	47.5337	107	6006	11	223	161
Capsella_rubella	-6.93	40.16	154	5974	56	346	25
Capsella_rubella	0.290842	47.6298	117	5339	11	200	143
Capsella_rubella	-7.2	38.7	171	5706	54	249	13
Capsella_rubella	1.33	38.98	180	4637	55	182	21
Capsella_rubella	4.61423	46.9617	101	6060	12	266	188
Capsella_rubella	13.339018	38.132388	180	5164	58	249	21
Capsella_rubella	-6.3575	41.47308	127	5767	42	244	38
Capsella_rubella	3.45671	47.2003	110	5892	11	256	186
Capsella_rubella	1.53291	47.6889	117	5563	11	181	136
Capsella_rubella	-1.628	42.629002	119	5444	25	283	123
Capsella_rubella	0.391131	48.1352	112	5275	14	226	153
Capsella_rubella	3.61085	48.0054	113	5824	13	215	152
Capsella_rubella	4.73269	47.5283	97	6003	12	245	175
Capsella_rubella	12.047593	43.64618	124	6838	27	264	113
Capsella_rubella	2.63645	46.8029	118	5786	11	205	157
Capsella_rubella	-7.70446	40.41664	137	5407	51	479	53
Capsella_rubella	1.88012	47.2848	119	5658	10	194	147
Capsella_rubella	3.69734	47.5356	110	5903	9	241	180
Capsella_rubella	8.305	36.49278	159	6049	53	250	23
Capsella_rubella	0.711407	48.1089	110	5349	11	197	145
Capsella_rubella	3.44437	48.7015	106	5652	11	206	145
Capsella_rubella	-0.055586	47.6975	118	5264	14	206	136
Capsella_rubella	19.8975	39.61306	173	6005	65	540	34
Capsella_rubella	7.976272	44.623746	129	6790	25	268	134
Capsella_rubella	-4.51	36.63	177	5458	73	315	13
Capsella_rubella	10.6243	44.0522	98	6696	24	310	162
Capsella_rubella	0.072614	48.0548	113	5222	16	226	140

Capsella_rubella	4.39664	48.7422	106	5810	11	199	143
Capsella_rubella	9.3694	44.6972	110	6846	31	380	128
Capsella_rubella	2.1789	44.74109	118	5854	20	308	164
Capsella_rubella	4.67665	46.4318	113	6159	11	269	197
Capsella_rubella	4.66083	47.7329	102	5980	11	240	168
Capsella_rubella	2.88701	47.4346	115	5795	11	204	146
Capsella_rubella	-5.245	36.723	149	5342	71	314	9
Capsella_rubella	0.726301	47.8844	113	5391	11	182	129
Capsella_rubella	6.1009	48.60579	103	6101	11	221	155
Capsella_rubella	-0.99	38.52	136	5797	38	176	38
Capsella_rubella	-9.33739	38.69986	167	3139	65	298	13
Capsella_rubella	4.95179	46.985	118	6167	13	219	149
Capsella_rubella	-2.79	43.06	108	4796	17	326	196
Capsella_rubella	10.826029	42.779376	164	5901	37	229	57
Capsella_rubella	4.3102	46.8315	105	6047	12	248	169
Capsella_rubella	-5.48	42.2	119	5679	30	164	51
Capsella_rubella	11.008753	43.752336	145	6771	28	394	141
Capsella_rubella	-2.74	43.1	129	4767	25	381	178
Capsella_rubella	3.64354	47.7107	112	5873	10	235	171
Capsella_rubella	11.4125	44.5094	146	7228	18	237	136
Capsella_rubella	-0.39	38.94	170	5303	51	256	34
Capsella_rubella	-3.46	43.46	149	4415	27	444	180
Capsella_rubella	24	35.3	63	4858	86	561	5
Capsella_rubella	-7.5341	38.42679	170	5449	57	246	11
Capsella_rubella	0.361756	47.3531	117	5369	13	204	139
Capsella_rubella	0.635501	47.3829	119	5432	13	199	136
Capsella_rubella	8.242182	44.610877	125	6723	26	279	120
Capsella_rubella	0.198468	47.8996	117	5292	13	211	144
Capsella_rubella	8.13205	44.621251	121	6727	28	255	117
Capsella_rubella	3.84618	46.5471	113	6018	13	258	193
Capsella_rubella	3.77568	47.4554	113	5934	11	217	156
Capsella_rubella	4.45637	46.7155	112	6095	11	246	175
Capsella_rubella	4.01059	46.2029	115	6079	14	266	176
Capsella_rubella	3.58171	47.7938	114	5854	9	200	150
Capsella_rubella	5.2215	47.6492	103	6070	13	233	154

Capsella_rubella	20.666667	38.133333	121	5544	76	597	23
Capsella_rubella	4.27631	46.5332	111	6082	10	276	211
Capsella_rubella	-0.295749	47.872	117	5181	16	226	141
Capsella_rubella	3.08686	46.9119	117	5866	11	221	168
Capsella_rubella	0.885607	47.7391	114	5444	8	182	144
Capsella_rubella	-6.3	39.4	159	6235	54	262	17
Capsella_rubella	-3.816	38.166	177	7036	50	210	18
Capsella_rubella	6.62414	46.52087	109	6536	9	316	246
Capsella_rubella	3.42606	48.7915	106	5633	11	189	132
Capsella_rubella	3.822	47.1181	108	5957	14	343	224
Capsella_rubella	1.11	42.48	69	5924	18	241	139
Capsella_rubella	5.296388	50.294166	94	5629	9	251	189
Capsella_rubella	6.632222	46.519722	103	6527	10	352	270
Capsella_rubella	1.62739	49.0387	113	5235	12	164	116
Capsella_rubella	0.227339	48.1101	115	5254	13	219	148
Capsella_rubella	3.42184	46.7293	116	5937	13	220	158
Capsella_rubella	2.79212	47.8144	113	5731	9	192	145
Capsella_rubella	3.65488	46.934	115	5962	12	265	186
Capsella_rubella	6.82013	46.47713	97	6558	9	389	301
Capsella_rubella	10.949922	43.161434	121	6198	25	286	119
Capsella_rubella	10.261707	43.821793	156	6481	38	355	90
Capsella_rubella	-9.20284	39.34293	164	3574	56	283	21
Capsella_rubella	10.117709	44.837156	141	7338	23	327	147
Capsella_rubella	5.87132	49.19833	102	5997	12	256	174
Capsella_rubella	-4.85187	36.59998	152	5227	74	357	12
Capsella_rubella	-7.48392	37.18432	184	4364	71	275	5
Capsella_rubella	2.74222	47.6477	114	5742	10	204	150
Capsella_rubella	4.14869	47.6728	108	5940	10	207	151
Capsella_rubella	3.59965	46.803	116	5964	12	259	190
Capsella_rubella	8.26479	47.28543	88	6422	21	492	299
Capsella_rubella	10.830005	43.878075	147	6796	26	325	139
Capsella_rubella	-4.51	36.72	186	5658	67	290	14
Capsella_rubella	7.898495	44.478956	133	6675	27	232	114
Capsella_rubella	4.42042	46.8334	101	6046	15	276	180
Capsella_rubella	8.003693	45.037797	133	7123	32	256	85

Capsella_rubella	0.438375	47.6385	116	5370	10	194	145
Capsella_rubella	9.5047	44.8253	123	7121	23	298	135
Capsella_rubella	0.513487	48.1218	113	5315	14	207	140
Capsella_rubella	-4.08	41.740002	117	6063	28	162	55
Capsella_rubella	8.52366	47.01342	62	6408	24	704	370
Capsella_rubella	2.31844	48.8162	111	5446	8	167	131
Capsella_rubella	2.680277	51.133333	107	4820	17	230	143
Capsella_rubella	26.75106	40.05661	134	6906	61	378	37
Capsella_rubella	15.94583	41.70806	136	5921	30	200	79
Capsella_rubella	0.144074	48.0711	114	5245	15	214	140
Capsella_rubella	3.8605	47.1981	105	5948	14	346	231
Capsella_rubella	2.15237	48.9521	113	5381	8	163	129
Capsella_rubella	-1.667	42.521	131	5551	24	211	89
Capsella_rubella	7.66977	47.24319	102	6441	14	338	236
Capsella_rubella	4.56375	46.6742	113	6120	12	229	157
Capsella_rubella	-1.691	41.963001	143	5956	22	130	73
Capsella_rubella	6.03651	48.65292	99	6072	10	239	175
Capsella_rubella	4.89794	47.1851	103	6083	13	240	163
Capsella_rubella	2.226667	45.113889	106	5799	16	393	233
Capsella_rubella	-6.75881	42.51674	128	5025	37	331	78
Capsella_rubella	11.49	48.17	91	6761	34	375	152
Capsella_rubella	3.85245	51.757367	105	4816	21	250	139
Capsella_rubella	5.23886	47.186	116	6175	14	238	163
Capsella_rubella	6.55532	46.74526	107	6450	10	231	169
Capsella_rubella	3.57403	46.9056	114	5946	12	269	192
Capsella_rubella	-0.72	39.86	112	5778	36	200	71
Capsella_rubella	-4.14	38.28	151	6895	47	237	26
Capsella_rubella	3.91001	47.8338	108	5879	10	221	162
Capsella_rubella	2.22	41.86	127	5850	20	209	123
Capsella_rubella	11.04467	43.82105	152	6838	29	330	115
Capsella_rubella	-6.73	38.58	162	5932	53	233	14
Capsella_rubella	4.43001	47.1602	104	6034	12	252	172
Capsella_rubella	4.67166	46.7073	115	6146	13	228	151
Capsella_rubella	4.78782	46.242	123	6232	14	259	166
Capsella_rubella	4.86129	46.4091	122	6225	13	254	164

Capsella_rubella	3.31939	45.37395	111	6034	23	207	106
Capsella_rubella	10.402068	43.59236	158	6319	40	354	83
Capsella_rubella	11.482653	42.514007	154	6176	30	218	71
Capsella_rubella	9.322307	39.31234	162	5065	52	210	21
Capsella_rubella	8.859319	39.193563	146	4924	54	271	25
Capsella_rubella	2.27266	48.2149	111	5579	9	171	136
Capsella_rubella	5.23636	47.0115	118	6202	14	242	166
Capsella_rubella	-2.49	43.1	124	4758	25	424	192
Capsella_rubella	9.01525	45.83807	116	7065	29	429	175
Capsella_rubella	3.06357	45.10426	81	5915	14	295	202
Capsella_rubella	0.191579	47.393	118	5333	13	189	131
Capsella_rubella	4.921111	50.245833	97	5580	9	269	204
Capsella_rubella	10.99472	43.780197	140	6741	26	390	156
Capsella_rubella	3.75455	47.5965	112	5912	10	232	168
Capsella_rubella	10.533516	43.751545	126	6488	36	427	122
Capsella_rubella	4.56691	46.8938	105	6074	13	244	165
Capsella_rubella	-4.037	36.785	180	5890	60	264	14
Capsella_rubella	4.60443	47.1044	104	6056	11	226	162
Capsella_rubella	0.258405	48.2091	114	5226	15	222	145
Capsella_rubella	0.56531	47.0777	122	5432	13	187	132
Capsella_rubella	0.802872	48.008	111	5382	11	187	135
Capsella_rubella	8.510866	45.148444	141	7228	25	264	132
Capsella_rubella	3.5145	47.8705	113	5840	9	194	145
Capsella_rubella	3.27937	47.5496	106	5816	11	229	166
Capsella_rubella	3.16614	47.8605	110	5767	9	220	163
Capsella_rubella	0.038346	48.3097	111	5121	18	245	146
Capsella_rubella	4.99598	45.807	128	6352	16	254	164
Capsella_rubella	3.45301	46.09388	117	5976	20	242	137
Capsella_rubella	2.01361	49.0615	109	5301	9	177	134
Capsella_rubella	4.03403	48.3974	111	5820	10	205	149
Capsella_rubella	8.91667	41.68333	162	5191	49	315	46
Capsella_rubella	1.57105	46.6582	120	5604	11	224	165
Capsella_rubella	10.075458	42.580947	167	5355	41	229	46
Capsella_rubella	4.15808	47.4606	108	5969	10	240	174
Capsella_rubella	2.0864	47.2414	117	5687	11	222	166

Capsella_rubella	8.85	42.21667	99	5099	45	427	76
Capsella_rubella	8.11113	44.72688	136	6927	25	252	122
Capsella_rubella	3.00307	47.2707	115	5828	11	245	179
Capsella_rubella	3.5349	47.5529	111	5872	12	227	162
Capsella_rubella	4.27773	47.1732	97	5979	15	308	199
Capsella_rubella	4.38162	46.8501	98	6028	13	314	211
Capsella_rubella	-1.48	43.175999	130	5124	25	468	204
Capsella_rubella	3.37965	46.56377	115	5931	15	248	165
Capsella_rubella	-7.68033	40.60995	133	5357	53	550	58
Capsella_rubella	3.44643	45.28347	109	6069	23	225	117
Capsella_rubella	4.57711	46.8645	109	6091	15	246	161
Capsella_rubella	-6.7	38.5	155	5928	53	249	15
Capsella_rubella	2.49592	48.802	113	5480	9	171	135
Capsella_rubella	4.0239	47.4663	106	5946	10	258	188
Capsella_rubella	0.352419	47.0142	121	5388	13	191	134
Capsella_rubella	8.58333	42.35	156	5071	46	357	56
Capsella_rubella	7.1488	45.89374	-8	6443	10	431	309
Capsella_rubella	0.088941	48.1379	113	5210	14	207	137
Capsella_rubella	3.4156	47.2612	107	5871	10	245	180
Capsella_rubella	8.456842	39.76946	178	5042	59	283	22
Capsella_rubella	1.19732	47.4679	118	5526	9	188	145
Capsella_rubella	8.773221	40.297975	135	5162	52	343	43
Capsella_rubella	2.9568	48.98	111	5503	11	176	126
Capsella_rubella	0.045542	48.1599	111	5194	14	238	159
Capsella_rubella	-1.302	42.966999	94	5289	24	467	219
Capsella_rubella	1.92924	47.2551	118	5660	10	215	164
Capsella_rubella	-1.738484	43.002829	111	5093	28	470	192
Capsella_rubella	4.4025	51.219444	105	5242	13	224	156
Capsella_rubella	3.99465	46.2852	115	6067	13	263	180
Capsella_rubella	0.740793	48.0533	110	5358	10	183	137
Capsella_rubella	20.88083	37.78083	180	5565	78	481	15
Capsella_rubella	3.54518	47.2877	111	5910	10	258	187
Capsella_rubella	0.122689	47.8579	116	5278	12	218	151
Capsella_rubella	-8.02222	41.12165	149	4882	52	422	49
Capsella_rubella	5.783707	53.19252	99	5037	20	244	137

Capsella_rubella	3.19289	45.73443	114	5954	23	247	129
Capsella_rubella	4.21	40.01	176	4712	56	239	20
Capsella_rubella	3.21777	47.0909	112	5866	11	255	188
Capsella_rubella	4.65288	46.903	112	6111	14	227	151
Capsella_rubella	11.482252	43.824284	140	6907	25	291	120
Capsella_rubella	4.418611	51.208888	105	5256	13	220	154
Capsella_rubella	139.37383	35.57101	152	7867	46	574	139
Capsella_rubella	4.12724	47.5052	109	5967	11	253	179
Capsella_rubella	11.495823	42.822505	112	6309	26	268	106
Capsella_rubella	0.773906	47.3918	120	5459	13	193	135
Capsella_rubella	0.569676	48.2078	110	5295	13	206	141
Capsella_rubella	17.665833	44.226111	92	7281	15	277	180
Capsella_rubella	4.80963	46.6222	115	6173	12	271	191
Capsella_rubella	0.446705	48.0228	113	5314	11	216	155
Capsella_rubella	-4.921	36.738	140	5401	72	351	11
Capsella_rubella	5.33851	46.4768	119	6292	9	340	262
Capsella_rubella	4.8815	47.1411	107	6102	13	225	152
Capsella_rubella	11.103552	43.942612	133	6861	24	366	161
Capsella_rubella	0.911089	47.8453	112	5424	11	190	139
Capsella_rubella	4.51162	46.8456	105	6071	15	270	179
Capsella_rubella	3.8281	40.0377	174	4748	55	276	28
Capsella_rubella	2.48764	48.563	113	5542	10	172	133
Capsella_rubella	8.340083	41.09412	169	4925	58	214	18
Capsella_rubella	9.931761	44.555861	121	7084	31	373	129
Capsella_rubella	-9.06675	38.92496	156	3697	63	324	15
Capsella_rubella	2.02652	46.5551	116	5671	8	215	175
Capsella_rubella	1.46166	46.9444	119	5590	10	213	166
Capsella_rubella	1.31475	47.2784	117	5554	11	188	139
Capsella_rubella	1.68377	48.1404	112	5507	8	169	133
Capsella_rubella	-4.09	41.77	120	6045	29	143	46
Capsella_rubella	4.28385	47.0519	103	6015	16	317	199
Capsella_rubella	0.463504	47.132	119	5407	14	205	138
Capsella_rubella	0.57	40.76	169	5693	37	238	71
Capsella_rubella	3.45438	47.1174	107	5888	11	250	180
Capsella_rubella	11.059971	43.838938	151	6866	27	310	120

Capsella_rubella	2.42971	44.92274	100	5847	15	442	263
Capsella_rubella	9.39795	47.54165	103	6574	27	338	163
Capsella_rubella	6.025556	47.241111	111	6255	9	303	229
Capsella_rubella	2.8656	47.3151	113	5791	12	223	160
Capsella_rubella	6.216667	46.183333	110	6542	12	274	202
Capsella_rubella	2.22226	47.2745	117	5704	11	201	147
Capsella_rubella	9.7464	44.9677	140	7352	21	311	162
Capsella_rubella	4.26347	47.4226	104	5977	11	256	183
Capsella_rubella	0.970148	47.7899	115	5456	8	181	140
Capsella_rubella	4.24669	46.8388	110	6047	11	288	200
Capsella_rubella	-8.45958	38.67612	167	4485	58	303	15
Capsella_rubella	0.64164	48.0529	111	5343	10	211	156
Capsella_rubella	2.38304	47.3122	111	5710	10	223	167
Capsella_rubella	3.92352	46.7595	110	6008	12	287	206
Capsella_rubella	11.186093	42.545933	164	6017	38	214	51
Capsella_rubella	11.39169	42.889722	147	6415	22	212	96
Capsella_rubella	12.071918	43.668775	110	6791	24	281	135
Capsella_rubella	-3.06	43.31	141	4600	22	384	185
Capsella_rubella	-0.149926	47.8836	116	5214	16	233	145
Capsella_rubella	-9.26349	39.0393	163	3397	58	275	18
Capsella_rubella	-9.18706	38.7121	162	3392	64	325	14
Capsella_rubella	0.88	41.31	122	5866	34	210	71
Capsella_rubella	0.020716	47.972	114	5233	17	246	153
Capsella_rubella	11.00408	43.12633	122	6261	31	314	108
Capsella_rubella	4.30129	48.8571	109	5785	11	186	134
Capsella_rubella	4.63182	47.0083	98	6049	11	275	197
Capsella_rubella	2.22714	48.8053	109	5426	8	167	133
Capsella_rubella	2.71368	48.3003	114	5638	10	172	131
Capsella_rubella	-9.09384	39.23394	162	3715	57	293	19
Capsella_rubella	4.1426	47.5614	105	5947	13	293	201
Capsella_rubella	8.717313	38.913678	183	4807	60	194	12
Capsella_rubella	10.871624	42.768721	159	5922	36	254	66
Capsella_rubella	25.28897	43.48386	126	8393	18	178	108
Capsella_rubella	4.24269	47.4025	102	5971	10	247	179
Capsella_rubella	4.0091	47.5625	110	5948	12	236	164

Capsella_rubella	4.37906	46.7564	110	6078	15	264	176
Capsella_rubella	1.98376	48.8191	106	5374	8	178	143
Capsella_rubella	35.33	31.84	184	5860	111	307	0
Capsella_rubella	9.05	42.41667	96	5122	46	359	61
Capsella_rubella	1.71334	47.5444	117	5608	11	197	148
Capsella_rubella	2.43383	44.65195	115	5913	15	325	201
Capsella_rubella	4.71028	46.335	115	6184	13	260	174
Capsella_rubella	4.46028	46.8522	104	6064	16	257	168
Capsella_rubella	1.40209	46.448	117	5566	10	252	185
Capsella_rubella	3.42374	46.9116	113	5921	12	256	185
Capsella_rubella	4.46	51.9	104	5078	19	253	150
Capsella_rubella	11.54	48.07	88	6749	35	405	159
Capsella_rubella	11.695246	42.663948	140	6385	25	226	91
Capsella_rubella	4.66632	46.3116	102	6123	11	283	209
Capsella_rubella	-1.23	42.66	113	5576	22	341	167
Capsella_rubella	4.836111	50.196111	96	5569	10	305	226
Capsella_rubella	20.7049	37.73992	180	5380	77	509	18
Capsella_rubella	3.058611	36.7575	193	5403	59	280	18
Capsella_rubella	9.060784	39.598137	160	5199	49	326	47
Capsella_rubella	2.42905	48.3208	112	5588	10	177	138
Capsella_rubella	3.1386	48.3085	113	5698	9	172	130
Capsella_rubella	4.63705	46.4166	114	6160	12	239	164
Capsella_rubella	3.09524	45.0367	90	5963	14	224	154
Capsella_rubella	4.51182	46.7313	109	6096	11	261	189
Capsella_rubella	1.49	39.05	172	4657	53	211	26
Capsella_rubella	2.54218	46.8987	118	5770	10	208	163
Capsella_rubella	-7.53943	41.1706	151	5343	48	289	36
Capsella_rubella	8.714066	39.820462	153	5158	55	366	38
Capsella_rubella	0.712279	47.4569	117	5440	13	214	148
Capsella_rubella	2.51757	46.641	118	5765	12	211	154
Capsella_rubella	2.93612	45.37438	60	5789	12	354	253
Capsella_rubella	3.27519	47.0815	111	5873	11	268	198
Capsella_thracica	27.42	42.259998	126	7369	21	219	109
Capsella_thracica	27.42	42.25	126	7369	21	219	109
Capsella_thracica	24.299999	42.049999	120	7842	19	221	132

Capsella_thracica	26.0817869	41.8302608	138	7732	20	190	100
Capsella_thracica	24.4242264	42.1192947	131	7905	19	211	128
Capsella_thracica	23.9376425	42.2452753	115	7831	23	211	103
Capsella_thracica	24.9226892	42.1387767	135	7939	19	213	130
Capsella_thracica	24.4718281	42.1435981	134	7933	19	201	121
Capsella_thracica	24.4828572	42.1387931	134	7933	19	201	121
Capsella_thracica	25.61499219	42.43958165	116	7884	20	195	113
Capsella_thracica	27.59657318	43.22250003	127	7822	17	136	89
Capsella_thracica	24.4768153	42.0458175	133	7910	19	201	122
Capsella_thracica	22.3664253	40.0870478	39	6982	30	238	88
Capsella_thracica	24.5653381	40.6306175	154	6956	51	249	42

Table S6. Prediction of ploidy level based on Hyb-Seq data for all *Catolobus* accessions.

nQuire statistical parameters were designated for each ploidy model (M): 2*x*, 3*x*, and 4*x* (logLfree, logL, delta logL, SSR, y-y slope, Std.err, and R2).
Median allelic ratios and histograms of allele frequency distribution were assigned for all accessions.
Chromosome numbers are indicated when available, while "na" indicates that no information is available for this parameter.

sample	Chr nbr	logL free	M	LogL	delta logL	SSR	Y-yslope	std.err	R2	Media-allele-freq	histo
1	30	5286,2	2x	983	4304	0,080	-0,316	0,067	0,272	2,4	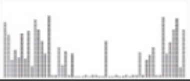
			3x	4209	1077	0,023	0,266	0,109	0,091		
			4x	4672	614	0,019	0,217	0,176	0,025		
4	30	10370,9	2x	428	9943	0,084	-0,372	0,059	0,403	2,8	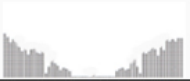
			3x	5341	5030	0,032	0,005	0,111	0,000		
			4x	9493	878	0,011	0,686	0,148	0,267		
6	30	12662,5	2x	1143	11519	0,079	-0,361	0,047	0,502	2,7	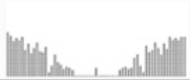
			3x	7710	4953	0,025	0,119	0,095	0,026		
			4x	10649	2014	0,011	0,435	0,139	0,142		
7	30	150853,7	2x	948	149906	0,075	-0,253	0,073	0,171	2,4	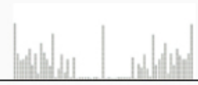
			3x	3283	147570	0,028	0,156	0,114	0,031		
			4x	3942	146912	0,019	0,214	0,178	0,024		
8	30	4082,1	2x	720	3362	0,083	-0,352	0,064	0,335	2,4	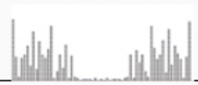
			3x	2994	1088	0,022	0,298	0,108	0,114		
			4x	2759	1323	0,022	0,045	0,179	0,001		
9	30	5177,7	2x	1546	3632	0,076	-0,308	0,058	0,324	2,3	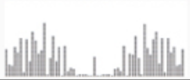
			3x	4741	437	0,016	0,384	0,090	0,237		
			4x	3483	1695	0,020	-0,050	0,160	0,002		
11	30	40108,2	2x	1288	38820	0,080	-0,324	0,064	0,306	2,3	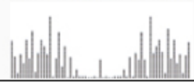
			3x	4358	35751	0,017	0,413	0,097	0,234		
			4x	3336	36772	0,022	-0,057	0,173	0,002		
13	30	4937,8	2x	1022	3916	0,080	-0,308	0,070	0,249	2,4	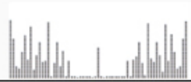
			3x	4029	908	0,026	0,228	0,113	0,064		
			4x	4291	647	0,021	0,151	0,181	0,012		
16	30	4862,4	2x	689	4174	0,086	-0,305	0,088	0,171	2,5	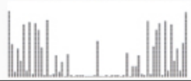
			3x	3264	1598	0,034	0,182	0,138	0,029		
			4x	4234	628	0,024	0,344	0,213	0,042		
19	30	61563,5	2x	683	60881	0,084	-0,340	0,071	0,280	2,7	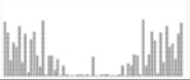
			3x	4108	57455	0,028	0,188	0,119	0,040		
			4x	5435	56129	0,019	0,358	0,184	0,060		
20	30	10322,8	2x	1274	9049	0,076	-0,334	0,046	0,473	2,7	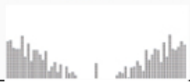
			3x	6846	3476	0,024	0,118	0,091	0,028		
			4x	9130	1192	0,011	0,419	0,132	0,145		
21	30	163458	2x	1130	162328	0,080	-0,273	0,080	0,166	2,4	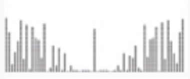
			3x	4177	159281	0,028	0,236	0,123	0,058		
			4x	4754	158703	0,022	0,230	0,195	0,023		
22	30	5636,1	2x	970	4666	0,079	-0,287	0,074	0,203	2,5	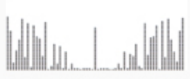
			3x	3979	1657	0,029	0,168	0,119	0,033		
			4x	4951	685	0,019	0,334	0,183	0,054		
23	30	5945,1	2x	1422	4523	0,078	-0,317	0,062	0,309	2,4	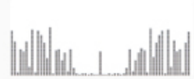
			3x	5120	825	0,020	0,321	0,100	0,149		
			4x	4627	1318	0,020	0,039	0,168	0,001		

24	30	11935,7	2x	1301	10635	0,078	-0,348	0,046	0,494	2,6	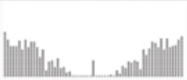
			3x	7743	4193	0,023	0,148	0,092	0,042		
			4x	10331	1605	0,011	0,429	0,135	0,147		
25	30	135048,8	2x	1103	133946	0,082	-0,284	0,083	0,167	2,4	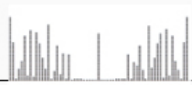
			3x	3912	131137	0,029	0,240	0,128	0,056		
			4x	4123	130926	0,025	0,121	0,205	0,006		
30	na	6091,5	2x	822	5270	0,080	-0,303	0,072	0,229	2,7	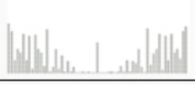
			3x	4033	2059	0,031	0,115	0,119	0,016		
			4x	5496	596	0,018	0,352	0,181	0,061		
35	na	6546,3	2x	685	5861	0,079	-0,315	0,065	0,285	2,7	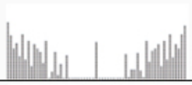
			3x	4039	2508	0,030	0,083	0,111	0,009		
			4x	5990	557	0,015	0,425	0,165	0,101		
38	na	12547,4	2x	1195	11353	0,079	-0,369	0,042	0,564	2,7	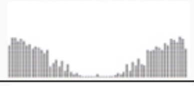
			3x	7795	4752	0,023	0,150	0,091	0,044		
			4x	10269	2278	0,010	0,453	0,132	0,166		
40	na	12591,2	2x	2828	9763	0,071	-0,318	0,023	0,764	2,4	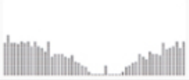
			3x	10050	2541	0,017	0,178	0,065	0,114		
			4x	9813	2778	0,011	0,153	0,105	0,034		
41	na	13178,8	2x	2077	11102	0,075	-0,351	0,031	0,679	2,5	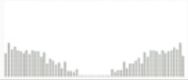
			3x	9490	3689	0,018	0,205	0,076	0,110		
			4x	10173	3006	0,011	0,242	0,122	0,063		
42	na	12385,0	2x	2063	10322	0,074	-0,331	0,036	0,584	2,5	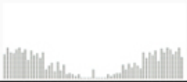
			3x	9108	3277	0,020	0,181	0,079	0,082		
			4x	10369	2016	0,010	0,327	0,120	0,111		
43	na	12944,4	2x	1946	10999	0,075	-0,343	0,033	0,641	2,6	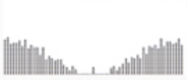
			3x	9203	3741	0,020	0,168	0,078	0,072		
			4x	10625	2319	0,010	0,312	0,120	0,103		
45	na	97731,5	2x	723	97008	0,080	-0,324	0,065	0,299	2,7	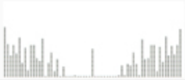
			3x	4016	93715	0,027	0,156	0,110	0,033		
			4x	5398	92333	0,016	0,348	0,169	0,067		
48	na	11381,2	2x	3102	8279	0,069	-0,295	0,027	0,668	2,4	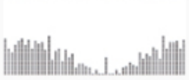
			3x	9755	1626	0,015	0,232	0,061	0,195		
			4x	8794	2587	0,011	0,113	0,105	0,019		
49	na	11467,6	2x	2796	8671	0,070	-0,308	0,025	0,714	2,4	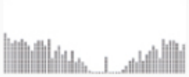
			3x	9355	2113	0,017	0,200	0,064	0,143		
			4x	8790	2678	0,012	0,090	0,107	0,012		
50	na	13205,7	2x	3012	10193	0,071	-0,320	0,023	0,763	2,4	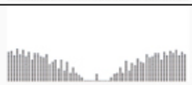
			3x	10618	2588	0,017	0,198	0,064	0,139		
			4x	10060	3145	0,011	0,129	0,107	0,024		
51	na	11060,2	2x	1045	10015	0,080	-0,351	0,053	0,430	2,7	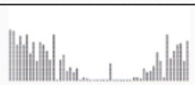
			3x	6913	4147	0,025	0,140	0,100	0,032		
			4x	9576	1484	0,013	0,425	0,148	0,123		

Table S7. Statistics on Hyb-Seq data for all Camelineae accessions studied using Angiosperm-353 and Brassicaceae-specific baits.

Species	Accession-code	ReadsMapped		PctOnTarget		GenesMapped		GenesWithContigs		GenesWithSeqs		GenesAt75pct	
		Brassicaceae baits	Angio-353	Brassicaceae baits	Angio-353	Brassicaceae baits	Angio-353	Brassicaceae baits	Angio-353	Brassicaceae baits	Angio-353	Brassicaceae baits	Angio-353
Catolobus pendulus	1	956894	337959	0.615	0.217	1827	349	1745	333	1739	333	1579	309
	4	11681239	4434317	0.489	0.185	1827	351	1826	349	1823	347	1690	337
	6	3546228	1442832	0.483	0.196	1827	350	1815	347	1812	347	1695	333
	7	1139026	408187	0.608	0.218	1827	349	1779	339	1774	337	1593	317
	8	1598709	539486	0.642	0.216	1827	350	1801	335	1795	335	1625	322
	9	1601980	531448	0.659	0.219	1827	349	1809	343	1805	341	1657	322
	11	1746747	597593	0.655	0.224	1827	349	1807	341	1802	339	1658	324
	13	1169186	449896	0.566	0.218	1827	350	1771	340	1760	339	1577	326
	16	598242	177775	0.63	0.187	1826	349	1561	313	1545	309	1406	259
	19	1136579	438552	0.563	0.217	1826	349	1771	339	1764	338	1571	319
	20	3559976	1207759	0.483	0.164	1827	350	1811	348	1809	347	1678	332
	21	784196	307389	0.556	0.218	1826	349	1716	333	1710	332	1519	311
	22	1000894	389696	0.561	0.219	1827	349	1753	333	1746	333	1583	314
	23	1355479	481811	0.618	0.22	1827	350	1792	338	1786	338	1624	321
	24	4406953	1802819	0.463	0.189	1827	350	1816	350	1812	348	1679	332
	25	671355	254915	0.58	0.22	1826	348	1697	329	1682	327	1543	297
	30	1186989	486343	0.478	0.196	1827	349	1749	341	1747	341	1626	329
	35	1392713	538113	0.567	0.218	1827	351	1792	346	1792	341	1690	317
	38	8753518	3428369	0.538	0.21	1827	353	1826	350	1823	348	1700	336
	40	8211504	3505058	0.553	0.236	1827	350	1827	350	1826	349	1715	326
	41	8324238	3107546	0.488	0.182	1827	350	1826	349	1823	348	1691	339
	42	6839235	2976728	0.478	0.208	1827	350	1826	350	1824	349	1703	334
	43	5521132	2248042	0.483	0.197	1827	350	1824	348	1823	346	1707	333
	45	1261328	526181	0.319	0.133	1827	353	1725	344	1721	339	1618	305
	48	2305539	984248	0.505	0.216	1827	352	1818	349	1814	348	1722	325
	49	3791032	1714685	0.486	0.22	1827	351	1823	350	1818	348	1716	333
	50	6362746	2790242	0.506	0.222	1827	353	1826	350	1824	349	1718	326
	51	5641873	2237370	0.432	0.171	1827	353	1826	349	1824	347	1739	331
Capsella grandiflora	C.grandiflora1	457423	163223	0.518	0.185	1824	350	1568	328	1566	327	1493	309
	C.grandiflora2	397793	156034	0.46	0.18	1825	351	1512	324	1505	323	1434	307
Capsella orientalis	C.orientalis1	319192	123640	0.473	0.183	1823	349	1375	316	1367	316	1313	305
	C.orientalis2	486244	182514	0.529	0.199	1824	350	1584	325	1581	324	1506	312
Capsella rubella	C.rubella1	404498	160506	0.478	0.19	1824	351	1493	320	1483	320	1428	308
	C.rubella2	356056	142698	0.473	0.189	1822	350	1429	321	1416	320	1361	312
Capsella thracica	C.thracica	619987	233612	0.528	0.199	1825	350	1661	330	1655	330	1543	310
Camelina neglecta	C.neglecta	512271	207655	0.526	0.213	1825	348	1572	324	1562	323	1495	315
Camelina hispida	C.hispida	905729	361672	0.545	0.218	1824	349	1712	335	1707	334	1580	317
Camelina laxa	C.laxa	493411	189328	0.534	0.205	1825	348	1542	324	1530	323	1456	315
Draba nuda	D.nuda	2013337	1447017	0.379	0.272	1803	350	1783	350	1714	347	1553	334

Table S8. Likelihood scores for 10 genetic groups (K) and 500 iterations indicating the best-fitting K value based on the method published by Evanno et al. (2005).

K	Reps	Mean LnP(K)	Stdev LnP(K)	Ln'(K)	|Ln''(K)|	Delta K
1	500	-26971.8002	908.5794	NA	NA	NA
2	500	-25154.1292	881.0806	1817.671	1530.4398	1.737003
3	500	-24866.898	895.332	287.2312	118.9668	0.132875
4	500	-24698.6336	962.8574	168.2644	167.0326	0.173476
5	500	-24697.4018	1162.3101	1.2318	94.4166	0.081232
6	500	-24601.7534	1204.5344	95.6484	270.1392	0.224269
7	500	-24776.2442	1953.6342	-174.4908	1471.052	0.752982
8	500	-26421.787	5734.2936	-1645.5428	1846.9756	0.322093
9	500	-29914.3054	11508.4384	-3492.5184	47.7106	0.004146
10	500	-33454.5344	16547.7283	-3540.229	NA	NA

Table S9. ***Catolobus*** **accessions from clusters I and II selected for the SNP count analysis.**

Cluster	Accession-code
I	9
	16
	22
	25
	11
	13
II	4
	38
	41
	42
	43
	50

Table S10. List of genes harboring specific SNPs from *Catolobus* cluster I and II, along with their chromosomal position based on ancestral genomic blocks within the *A. thaliana* genome.

AK genomic block + gene	Chromosome number	Location on Chromosome (ruff estimation)	Cluster number
AK1A+AT1G01220	chr1	91342	I
AK1A+AT1G01290	chr1	114202	I
AK1A+AT1G01770	chr1	278600	I
AK1A+AT1G01910	chr1	313101	I
AK1A+AT1G02410	chr1	490860	I
AK1A+AT1G02680	chr1	580625	II
AK1A+AT1G02970	chr1	673306	I
AK1A+AT1G03030	chr1	701365	I
AK1A+AT1G03090	chr1	739544	I
AK1A+AT1G03100	chr1	743885	I
AK1A+AT1G03360	chr1	824517	I
AK1A+AT1G03390	chr1	841033	I
AK1A+AT1G04110	chr1	1061297	I
AK1A+AT1G04730	chr1	1325099	I
AK1A+AT1G04910	chr1	1387871	I
AK1A+AT1G04970	chr1	1411088	I
AK1A+AT1G05910	chr1	1790216	I
AK1A+AT1G06240	chr1	1911095	I
AK1A+AT1G06260	chr1	1916430	I
AK1A+AT1G06270	chr1	1917991	I
AK1A+AT1G06560	chr1	2007615	I
AK1A+AT1G06950	chr1	2130053	I
AK1A+AT1G07010	chr1	2152798	I
AK1A+AT1G07320	chr1	2248967	I
AK1A+AT1G08460	chr1	2672198	I
AK1A+AT1G09010	chr1	2895166	I
AK1A+AT1G09800	chr1	3176883	II
AK1A+AT1G09830	chr1	3192568	I
AK1A+AT1G09870	chr1	3205579	II
AK1A+AT1G09900	chr1	3218133	I
AK1A+AT1G10240	chr1	3356627	I
AK1A+AT1G11000	chr1	3671756	I

AK1A+AT1G11915	chr1	4021750	I
AK1A+AT1G12244	chr1	4157654	I
AK1A+AT1G13330	chr1	4567935	I
AK1A+AT1G15980	chr1	5489145	I
AK1A+AT1G16070	chr1	5510301	I
AK1A+AT1G16570	chr1	5670577	I
AK1A+AT1G16770	chr1	5737839	I
AK1A+AT1G16870	chr1	5770732	I
AK1A+AT1G18340	chr1	6311443	II
AK1A+AT1G19025	chr1	6568674	I
AK1B+AT1G19860	chr1	6891382	I
AK1B+AT1G20080	chr1	6962030	II
AK1B+AT1G20540	chr1	7112611	II
AK1B+AT1G21640	chr1	7588414	I
AK1B+AT1G22770	chr1	8061751	I
AK1B+AT1G24610	chr1	8720651	I
AK1B+AT1G24706	chr1	8741836	I
AK1B+AT1G25570	chr1	8991708	I
AK1B+AT1G26220	chr1	9071140	I
AK1B+AT1G26940	chr1	9343112	I
AK1B+AT1G27460	chr1	9534488	I
AK1B+AT1G28140	chr1	9831876	I
AK1B+AT1G29690	chr1	10379020	I
AK1B+AT1G30000	chr1	10509156	I
AK1B+AT1G30130	chr1	10587471	I
AK1C+AT1G47670	chr1	17536625	I
AK1C+AT1G48090	chr1	17732010	I
AK1C+AT1G49040	chr1	18139174	I
AK1C+AT1G49380	chr1	18276643	I
AK1C+AT1G49970	chr1	18501570	I
AK1C+AT1G50120	chr1	18561728	II
AK1C+AT1G50575	chr1	18728634	II
AK1C+AT1G50670	chr1	18774805	I
AK1C+AT1G50900	chr1	18866197	I
AK1C+AT1G50940	chr1	18877812	I

AK1C+AT1G51550	chr1	19117032	I
AK1C+AT1G52590	chr1	19589009	I
AK1C+AT1G52760	chr1	19651172	I
AK1C+AT1G53270	chr1	19862821	I
AK1C+AT1G55150	chr1	20574474	II
AK1C+AT1G55250	chr1	20606953	I
AK1C+AT1G55480	chr1	20713499	I
AK1C+AT1G55510	chr1	20723279	I
AK1C+AT1G55670	chr1	20802670	I
AK1C+AT1G55760	chr1	20846872	I
AK1C+AT1G55910	chr1	20905914	I
AK2D-AT1G57770	chr3	100000	I
AK2D-AT1G60060	chr3	844074	I
AK2D-AT1G60560	chr3	1013382	I
AK2D-AT1G60995	chr3	1170475	I
AK2D-AT1G61850	chr3	1560655	I
AK2D-AT1G63160	chr3	2126738	II
AK2D-AT1G63660	chr3	2308695	I
AK2D-AT1G63970	chr3	2443577	I
AK2E+AT1G65030	chr3	3098084	I
AK2E+AT1G65320	chr3	3201292	I
AK2E+AT1G66520	chr3	3757540	II
AK2E+AT1G66830	chr3	3871310	I
AK2E+AT1G67620	chr3	4281765	I
AK2E+AT1G68740	chr3	4753861	I
AK2E+AT1G69680	chr3	5146217	I
AK2E+AT1G69860	chr3	5251040	I
AK2E+AT1G71220	chr3	5782962	I
AK2E+AT1G72280	chr3	6153233	I
AK2E+AT1G72500	chr3	6236559	I
AK2E+AT1G73180	chr3	6459303	I
AK2E+AT1G73930	chr3	6736452	I
AK2E+AT1G73960	chr3	6746291	I
AK2E+AT1G73990	chr3	6765767	I
AK2E+AT1G74460	chr3	6929302	I

AK2E+AT1G74530	chr3	6950877	I
AK2E+AT1G74640	chr3	6973974	I
AK2E+AT1G75330	chr3	7207671	I
AK2E+AT1G76080	chr3	7489198	II
AK2E+AT1G76400	chr3	7599966	I
AK2E+AT1G76450	chr3	7625963	I
AK2E+AT1G78010	chr3	8274824	I
AK2E+AT1G79150	chr3	8713922	II
AK2E+AT1G79560	chr3	8868036	I
AK2E+AT1G80460	chr3	9188095	I
AK3F+AT3G01100	chr5	34658	I
AK3F+AT3G01150	chr5	51496	II
AK3F+AT3G01670	chr5	247192	I
AK3F+AT3G02130	chr5	380350	I
AK3F+AT3G02260	chr5	430877	I
AK3F+AT3G02300	chr5	461072	I
AK3F+AT3G02570	chr5	543346	I
AK3F+AT3G02660	chr5	570134	I
AK3F+AT3G02710	chr5	583113	I
AK3F+AT3G03100	chr5	705381	II
AK3F+AT3G03380	chr5	799500	I
AK3F+AT3G03710	chr5	919362	I
AK3F+AT3G03790	chr5	961750	II
AK3F+AT3G04340	chr5	1146568	I
AK3F+AT3G04480	chr5	1193540	I
AK3F+AT3G04970	chr5	1376154	I
AK3F+AT3G06270	chr5	1895858	II
AK3F+AT3G06530	chr5	2022433	I
AK3F+AT3G06880	chr5	2169768	I
AK3F+AT3G06920	chr5	2181346	I
AK3F+AT3G06950	chr5	2192685	I
AK3F+AT3G07050	chr5	2229355	I
AK3F+AT3G07080	chr5	2241002	I
AK3F+AT3G07140	chr5	2261104	I
AK3F+AT3G07750	chr5	2473194	I

AK3F+AT3G08670	chr5	2633663	I
AK3F+AT3G08760	chr5	2657838	I
AK3F+AT3G08960	chr5	2729743	I
AK3F+AT3G09650	chr5	2958676	I
AK3F+AT3G09720	chr5	2979772	I
AK3F+AT3G10030	chr5	3092016	I
AK3F+AT3G10110	chr5	3116110	I
AK3F+AT3G10130	chr5	3130063	I
AK3F+AT3G10400	chr5	3232608	I
AK3F+AT3G10700	chr5	3346509	II
AK3F+AT3G11540	chr5	3631887	I
AK3F+AT3G11945	chr5	3779839	I
AK3F+AT3G11960	chr5	3786314	I
AK3F+AT3G11964	chr5	3793731	I
AK3F+AT3G12210	chr5	3894753	I
AK3F+AT3G12280	chr5	3913349	I
AK3F+AT3G12610	chr5	4006346	I
AK3F+AT3G14120	chr5	4677699	I
AK3F+AT3G16270	chr5	5513267	I
AK3F+AT3G17170	chr5	5853059	I
AK3F+AT3G17465	chr5	5977722	I
AK3F+AT3G17470	chr5	5979814	I
AK3F+AT3G17810	chr5	6093990	I
AK3F+AT3G17830	chr5	6101753	II
AK3F+AT3G17880	chr5	6123341	I
AK3F+AT3G17900	chr5	6128506	I
AK3F+AT3G18524	chr5	6367941	I
AK3F+AT3G18730	chr5	6445852	I
AK3F+AT3G19210	chr5	6652695	II
AK3F+AT3G19553	chr5	6790731	I
AK3F+AT3G19970	chr5	6958509	I
AK3F+AT3G19990	chr5	6965595	I
AK3F+AT3G20240	chr5	7056903	I
AK3F+AT3G20260	chr5	7063910	I
AK3F+AT3G20320	chr5	7087445	I

AK3F+AT3G20630	chr5	7202748	I
AK3F+AT3G20970	chr5	7348157	II
AK3F+AT3G21110	chr5	7402477	I
AK3F+AT3G21540	chr5	7585886	I
AK3F+AT3G21720	chr5	7652487	I
AK3F+AT3G22425	chr5	7951023	II
AK3F+AT3G25430	chr5	9221135	I
AK3F+AT3G25470	chr5	9232821	I
AK3G+AT2G07340	chr5	9732821	II
AK3H+AT2G13540	chr5	11927503	I
AK3H+AT2G15240	chr5	12906075	I
AK3H+AT2G16440	chr5	13416898	I
AK3H+AT2G17510	chr5	13899855	I
AK3H+AT2G17760	chr5	14003853	I
AK3H+AT2G18710	chr5	14402703	II
AK3H+AT2G18760	chr5	14419325	I
AK3H+AT2G19870	chr5	14866404	I
AK3H+AT2G20050	chr5	14940053	I
AK3H+AT2G20190	chr5	15002198	I
AK4I+AT2G21470	chr7	100000	I
AK4I+AT2G21610	chr7	146469	I
AK4I+AT2G22120	chr7	295222	I
AK4I+AT2G23140	chr7	746841	I
AK4I+AT2G24230	chr7	1203248	I
AK4I+AT2G25280	chr7	1663786	I
AK4I+AT2G25710	chr7	1853978	II
AK4I+AT2G25800	chr7	1907206	I
AK4I+AT2G26690	chr7	2248578	I
AK4I+AT2G26780	chr7	2311259	I
AK4I+AT2G26800	chr7	2330450	I
AK4I+AT2G26930	chr7	2393138	I
AK4I+AT2G27090	chr7	2468867	I
AK4I+AT2G27450	chr7	2638805	I
AK4I+AT2G27500	chr7	2653495	I
AK4I+AT2G27590	chr7	2678985	I

AK4I+AT2G28070	chr7	2857304	II
AK4J+AT2G31040	chr7	4110055	I
AK4J+AT2G31740	chr7	4392225	I
AK4J+AT2G31955	chr7	4485416	I
AK4J+AT2G32290	chr7	4615947	I
AK4J+AT2G32590	chr7	4730721	I
AK4J+AT2G32900	chr7	4855096	II
AK4J+AT2G33770	chr7	5178989	I
AK4J+AT2G34640	chr7	5483266	I
AK4J+AT2G34860	chr7	5609674	I
AK4J+AT2G34980	chr7	5650308	I
AK4J+AT2G35360	chr7	5790479	II
AK4J+AT2G35450	chr7	5803875	I
AK4J+AT2G35610	chr7	5848774	I
AK4J+AT2G35920	chr7	5976774	I
AK4J+AT2G36740	chr7	6308053	I
AK4J+AT2G36840	chr7	6352951	I
AK4J+AT2G36895	chr7	6389966	I
AK4J+AT2G37230	chr7	6538328	II
AK4J+AT2G37370	chr7	6580538	II
AK4J+AT2G38000	chr7	6804556	I
AK4J+AT2G38500	chr7	7018826	I
AK4J+AT2G38510	chr7	7025416	I
AK4J+AT2G38770	chr7	7104373	I
AK4J+AT2G39260	chr7	7293355	I
AK4J+AT2G39730	chr7	7472177	I
AK4J+AT2G39830	chr7	7521174	I
AK4J+AT2G40070	chr7	7629624	I
AK4J+AT2G40190	chr7	7686604	II
AK4J+AT2G40570	chr7	7842253	I
AK4J+AT2G40760	chr7	7911382	I
AK4J+AT2G40860	chr7	7954929	I
AK4J+AT2G40890	chr7	7959484	I
AK4J+AT2G41190	chr7	8068710	I
AK4J+AT2G41760	chr7	8323120	II

AK4J+AT2G42490	chr7	8592838	I
AK4J+AT2G42700	chr7	8679561	I
AK4J+AT2G42750	chr7	8694696	II
AK4J+AT2G42850	chr7	8732839	I
AK4J+AT2G43030	chr7	8796227	I
AK4J+AT2G43235	chr7	8870129	II
AK4J+AT2G43890	chr7	9078262	I
AK4J+AT2G45500	chr7	9650783	I
AK4J+AT2G45770	chr7	9752519	I
AK4J+AT2G46060	chr7	9842530	I
AK4J+AT2G46370	chr7	9935017	I
AK4J+AT2G46580	chr7	10030661	II
AK4J+AT2G46890	chr7	10168095	I
AK4J+AT2G47020	chr7	10221116	I
AK4J+AT2G47420	chr7	10358805	I
AK4J+AT2G47790	chr7	10472395	I
AK4J+AT2G48070	chr7	10564146	I
AK5KL+AT2G03420	chr9	100000	II
AK5KL+AT2G04270	chr9	541122	I
AK5KL+AT2G04660	chr9	691073	I
AK5KL+AT2G04842	chr9	764345	II
AK5KL+AT3G25660	chr9	1105785	I
AK5KL+AT3G26085	chr9	1297038	I
AK5KL+AT3G26090	chr9	1299056	I
AK5KL+AT3G26410	chr9	1435858	I
AK5KL+AT3G26700	chr9	1576025	I
AK5KL+AT3G26710	chr9	1579666	I
AK5KL+AT3G27730	chr9	2039541	I
AK5KL+AT3G27870	chr9	2096990	I
AK5KL+AT3G28040	chr9	2201367	I
AK5KL+AT3G29320	chr9	3019250	I
AK5MN+AT3G44330	chr9	5226080	I
AK5MN+AT3G44880	chr9	5603167	I
AK5MN+AT3G46220	chr9	6198620	I
AK5MN+AT3G47400	chr9	6685082	I

AK5MN+AT3G47700	chr9	6803173	I
AK5MN+AT3G47860	chr9	6876174	I
AK5MN+AT3G48110	chr9	6982542	I
AK5MN+AT3G48425	chr9	7151277	I
AK5MN+AT3G48540	chr9	7209370	II
AK5MN+AT3G48610	chr9	7231047	I
AK5MN+AT3G49400	chr9	7538632	I
AK5MN+AT3G52200	chr9	8579701	I
AK5MN+AT3G52390	chr9	8642560	I
AK5MN+AT3G53100	chr9	8904258	I
AK5MN+AT3G53180	chr9	8926392	I
AK5MN+AT3G54860	chr9	9543660	I
AK5MN+AT3G55070	chr9	9627846	I
AK5MN+AT3G55160	chr9	9664074	I
AK5MN+AT3G55580	chr9	9832265	I
AK5MN+AT3G56120	chr9	10042718	I
AK5MN+AT3G56370	chr9	10118671	I
AK5MN+AT3G56650	chr9	10204207	I
AK5MN+AT3G56940	chr9	10296077	I
AK5MN+AT3G57180	chr9	10383117	I
AK5MN+AT3G58520	chr9	10863342	I
AK5MN+AT3G60660	chr9	11640916	II
AK5MN+AT3G60830	chr9	11693555	I
AK5MN+AT3G61960	chr9	12161337	II
AK5MN+AT3G62130	chr9	12224360	I
AK5MN+AT3G62810	chr9	12447194	I
AK5MN+AT3G63140	chr9	12546439	I
AK6O+AT4G00026	chr11	100	II
AK6O+AT4G00560	chr11	229919	I
AK6O+AT4G00740	chr11	296154	I
AK6O+AT4G00910	chr11	376673	I
AK6O+AT4G01037	chr11	440479	I
AK6O+AT4G01570	chr11	668757	I
AK6O+AT4G01730	chr11	738592	I
AK6O+AT4G01880	chr11	799942	I

AK6O+AT4G02030	chr11	881367	I
AK6O+AT4G02680	chr11	1170208	I
AK6O+AT4G02780	chr11	1226956	I
AK6O+AT4G02790	chr11	1236612	I
AK6O+AT4G02990	chr11	1311043	I
AK6O+AT4G03020	chr11	1320684	I
AK6O+AT4G03220	chr11	1406757	II
AK6O+AT4G03240	chr11	1412523	II
AK6O+AT4G04970	chr11	2526210	I
AK6O+AT4G05090	chr11	2598351	I
AK6P-AT4G07410	chr11	4189791	II
AK6P-AT4G08150	chr11	5136984	I
AK6P-AT4G08280	chr11	5219530	II
AK6P-AT4G08790	chr11	5597433	I
AK6P-AT4G08920	chr11	5712936	I
AK6P-AT4G09750	chr11	6135908	I
AK6P-AT4G10050	chr11	6273593	II
AK6P-AT4G10180	chr11	6335696	II
AK6P-AT4G11120	chr11	6767037	I
AK6Q-AT5G23050	chr11	7720506	I
AK6Q-AT5G23300	chr11	7836872	I
AK6Q-AT5G23395	chr11	7863857	II
AK6Q-AT5G24240	chr11	8220201	I
AK6Q-AT5G24840	chr11	8523072	I
AK6Q-AT5G25230	chr11	8728830	I
AK6Q-AT5G26570	chr11	9250764	I
AK6Q-AT5G30510	chr11	11608285	I
AK6R-AT5G01230	chr11	13020506	II
AK6R-AT5G01360	chr11	13076084	I
AK6R-AT5G01590	chr11	13152802	II
AK6R-AT5G02810	chr11	13566349	I
AK6R-AT5G02820	chr11	13571222	I
AK6R-AT5G03070	chr11	13646804	I
AK6R-AT5G03280	chr11	13716096	I
AK6R-AT5G04480	chr11	14200173	I

AK6R-AT5G04520	chr11	14218553	II
AK6R-AT5G04910	chr11	14366231	I
AK6R-AT5G04930	chr11	14373306	I
AK6R-AT5G05200	chr11	14472675	I
AK6R-AT5G06360	chr11	14873422	I
AK6R-AT5G06410	chr11	14888035	II
AK6R-AT5G06970	chr11	15086638	I
AK6R-AT5G08280	chr11	15592167	I
AK6R-AT5G08320	chr11	15605705	II
AK6R-AT5G08415	chr11	15639449	I
AK6R-AT5G08490	chr11	15671998	I
AK6R-AT5G08660	chr11	15742538	I
AK6R-AT5G09680	chr11	15927863	I
AK6R-AT5G10700	chr11	16306721	I
AK6R-AT5G10900	chr11	16364617	I
AK6R-AT5G11040	chr11	16423863	II
AK6R-AT5G11330	chr11	16545806	I
AK6R-AT5G11380	chr11	16558656	I
AK6R-AT5G11390	chr11	16562317	I
AK6R-AT5G12470	chr11	16973288	II
AK6R-AT5G13020	chr11	17057822	I
AK6R-AT5G13030	chr11	17061802	I
AK6R-AT5G13420	chr11	17230460	I
AK6R-AT5G13530	chr11	17273870	I
AK6R-AT5G13630	chr11	17315975	I
AK6R-AT5G13640	chr11	17321605	I
AK6R-AT5G13680	chr11	17338982	I
AK6R-AT5G14140	chr11	17490117	I
AK6R-AT5G14210	chr11	17507046	I
AK6R-AT5G14230	chr11	17520358	I
AK6R-AT5G14660	chr11	17655309	I
AK6R-AT5G14700	chr11	17668906	I
AK6R-AT5G14760	chr11	17696931	I
AK6R-AT5G14950	chr11	17765739	I
AK6R-AT5G15300	chr11	17896757	I

AK6R-AT5G15400	chr11	17926157	I
AK6R-AT5G15540	chr11	17976183	I
AK6R-AT5G16300	chr11	18266563	I
AK6R-AT5G17070	chr11	18541975	I
AK6R-AT5G17170	chr11	18577725	I
AK6R-AT5G17230	chr11	18588222	I
AK6R-AT5G17290	chr11	18614803	I
AK6R-AT5G17530	chr11	18706137	II
AK6R-AT5G18525	chr11	19075393	I
AK6R-AT5G18670	chr11	19154624	I
AK6R-AT5G19180	chr11	19381916	I
AK6R-AT5G19610	chr11	19546333	I
AK6R-AT5G19640	chr11	19564465	II
AK6R-AT5G19680	chr11	19578242	II
AK6R-AT5G19690	chr11	19580974	I
AK6R-AT5G20170	chr11	19736043	II
AK6R-AT5G20350	chr11	19805109	II
AK6R-AT5G20890	chr11	20015321	I
AK6R-AT5G21930	chr11	20171670	I
AK6R-AT5G22030	chr11	20218371	I
AK6R-AT5G22350	chr11	20326054	I
AK6R-AT5G22450	chr11	20365573	I
AK6R-AT5G22510	chr11	20403303	II
AK6R-AT5G22750	chr11	20493705	I
AK6R-AT5G22800	chr11	20544415	I
AK6R-AT5G22850	chr11	20562041	I
AK7S-AT5G35460	chr13	100	I
AK7S-AT5G36880	chr13	562222	II
AK7S-AT5G36890	chr13	568926	II
AK7S-AT5G38520	chr13	1048926	I
AK7S-AT5G38530	chr13	1051617	I
AK7S-AT5G39040	chr13	1253097	I
AK7S-AT5G39500	chr13	1442196	I
AK7S-AT5G39710	chr13	1523319	II
AK7S-AT5G40405	chr13	1696586	I

AK7S-AT5G40440	chr13	1709265	I
AK7S-AT5G40530	chr13	1762098	I
AK7S-AT5G40740	chr13	1829483	I
AK7S-AT5G40820	chr13	1870365	I
AK7S-AT5G41800	chr13	2161300	I
AK7S-AT5G41880	chr13	2188483	II
AK7T+AT4G14147	chr13	2292672	II
AK7T+AT4G15420	chr13	2962267	I
AK7T+AT4G16144	chr13	3276654	II
AK7U+AT4G16340	chr13	3367050	I
AK7U+AT4G16510	chr13	3441467	II
AK7U+AT4G16570	chr13	3475561	II
AK7U+AT4G16660	chr13	3515564	I
AK7U+AT4G17090	chr13	3743827	I
AK7U+AT4G18010	chr13	4129853	I
AK7U+AT4G18340	chr13	4268915	I
AK7U+AT4G20050	chr13	4988625	I
AK7U+AT4G20070	chr13	5000247	I
AK7U+AT4G20850	chr13	5299586	I
AK7U+AT4G21680	chr13	5656218	II
AK7U+AT4G21770	chr13	5702382	I
AK7U+AT4G22720	chr13	6075807	I
AK7U+AT4G23540	chr13	6419278	I
AK7U+AT4G24610	chr13	6839182	I
AK7U+AT4G24790	chr13	6916305	I
AK7U+AT4G24830	chr13	6931692	I
AK7U+AT4G24880	chr13	6947207	I
AK7U+AT4G24930	chr13	6960043	II
AK7U+AT4G25080	chr13	7015687	I
AK7U+AT4G26310	chr13	7452282	I
AK7U+AT4G26750	chr13	7614482	I
AK7U+AT4G26980	chr13	7687411	I
AK7U+AT4G27640	chr13	7936481	I
AK7U+AT4G28080	chr13	8087468	I
AK7U+AT4G28220	chr13	8131075	II

AK7U+AT4G28660	chr13	8288714	I
AK7U+AT4G29010	chr13	8435909	I
AK7U+AT4G29380	chr13	8597513	I
AK7U+AT4G29810	chr13	8731828	I
AK7U+AT4G30510	chr13	9043962	I
AK7U+AT4G30790	chr13	9131261	I
AK7U+AT4G31200	chr13	9300465	II
AK7U+AT4G31770	chr13	9508926	I
AK7U+AT4G31790	chr13	9516023	I
AK7U+AT4G32910	chr13	10020110	II
AK7U+AT4G33030	chr13	10074238	I
AK7U+AT4G33210	chr13	10154505	I
AK7U+AT4G33330	chr13	10198619	I
AK7U+AT4G33410	chr13	10219625	I
AK7U+AT4G33440	chr13	10230400	I
AK7U+AT4G33460	chr13	10236935	II
AK7U+AT4G33760	chr13	10328009	I
AK7U+AT4G34260	chr13	10536860	II
AK7U+AT4G34310	chr13	10552839	I
AK7U+AT4G34350	chr13	10567272	I
AK7U+AT4G34730	chr13	10708233	II
AK7U+AT4G34850	chr13	10747069	I
AK7U+AT4G35250	chr13	10909938	I
AK7U+AT4G35560	chr13	11020201	II
AK7U+AT4G35850	chr13	11122390	II
AK7U+AT4G36180	chr13	11258838	I
AK7U+AT4G36530	chr13	11378776	I
AK7U+AT4G36790	chr13	11474988	I
AK7U+AT4G37020	chr13	11584486	II
AK7U+AT4G39470	chr13	12498063	II
AK7U+AT4G39850	chr13	12627299	I
AK8V-AT5G43920	chr14	1000	I
AK8V-AT5G44000	chr14	30382	II
AK8V-AT5G44370	chr14	203106	I
AK8V-AT5G45780	chr14	895170	I

AK8V-AT5G46100	chr14	1022688	I
AK8V-AT5G46220	chr14	1067069	I
AK8V-AT5G46580	chr14	1225817	I
AK8V-AT5G47010	chr14	1400196	I
AK8V-AT5G47040	chr14	1421230	I
AK8V-AT5G47090	chr14	1455559	I
AK8V-AT5G47690	chr14	1646040	I
AK8V-AT5G47780	chr14	1676246	I
AK8W+AT5G48385	chr14	4009365	I
AK8W+AT5G48470	chr14	4042619	II
AK8W+AT5G48520	chr14	4062078	I
AK8W+AT5G48800	chr14	4187340	I
AK8W+AT5G49430	chr14	4337863	II
AK8W+AT5G49810	chr14	4440168	I
AK8W+AT5G50160	chr14	4616639	I
AK8W+AT5G50170	chr14	4621826	I
AK8W+AT5G50210	chr14	4643488	I
AK8W+AT5G50840	chr14	4786647	I
AK8W+AT5G50960	chr14	4835011	I
AK8W+AT5G51070	chr14	4864971	I
AK8W+AT5G51150	chr14	4889788	I
AK8W+AT5G51170	chr14	4894669	II
AK8W+AT5G51200	chr14	4905590	I
AK8W+AT5G51340	chr14	4963777	I
AK8W+AT5G51540	chr14	5032906	I
AK8W+AT5G52980	chr14	5483015	I
AK8W+AT5G53000	chr14	5486192	I
AK8W+AT5G53320	chr14	5637116	I
AK8W+AT5G53580	chr14	5765812	II
AK8W+AT5G53800	chr14	5848910	I
AK8W+AT5G54260	chr14	6032913	II
AK8W+AT5G55220	chr14	6398332	II
AK8W+AT5G55540	chr14	6497071	I
AK8W+AT5G55960	chr14	6663407	I
AK8W+AT5G56220	chr14	6754763	I

AK8W+AT5G56290	chr14	6787371	I
AK8W+AT5G57040	chr14	7084773	I
AK8W+AT5G57450	chr14	7273942	I
AK8W+AT5G58230	chr14	7556887	II
AK8W+AT5G58470	chr14	7638290	I
AK8W+AT5G58480	chr14	7641848	I
AK8W+AT5G58750	chr14	7729463	I
AK8W+AT5G60020	chr14	8068871	I
AK8W+AT5G60540	chr14	8237498	I
AK8W+AT5G60750	chr14	8331705	I
AK8X+AT5G61560	chr14	8553658	II
AK8X+AT5G61865	chr14	8648185	I
AK8X+AT5G62030	chr14	8719354	I
AK8X+AT5G62130	chr14	8751139	I
AK8X+AT5G63010	chr14	8982551	I
AK8X+AT5G63050	chr14	8993279	I
AK8X+AT5G63420	chr14	9101192	I
AK8X+AT5G63770	chr14	9219279	I
AK8X+AT5G63840	chr14	9245782	I
AK8X+AT5G63920	chr14	9275294	II
AK8X+AT5G64370	chr14	9440006	II
AK8X+AT5G64730	chr14	9573976	I
AK8X+AT5G65500	chr14	9881968	I
AK8X+AT5G65950	chr14	10080998	I
AK8X+AT5G66470	chr14	10242437	I
AK8X+AT5G66960	chr14	10437084	I

Table S11. ABBA-BABA related statistics on trios including *Capsella* and *Catolobus* taxa.

P1	P2	P3	D-statistic	Z-score	p-value	f4-ratio	BBAA	ABBA	BABA
Capsella_orientalis	*Capsella_thracica*	*Capsella_grandiflora*	0.577022	15.7287	2.30E-16	0.256511	3456	2759	740
Capsella_orientalis	*Capsella_thracica*	*Capsella_rubella*	0.64811	15.4976	2.30E-16	0.320941	3227	3227	689
Capsella_grandiflora	*Capsella_rubella*	*Capsella_thracica*	0.315242	6.07544	1.24E-09	0.122754	3283	1454	757
Capsella_grandiflora	*Capsella_rubella*	*Capsella_orientalis*	0.10545	2.6349	0.008416	0.023311	5300	933	755
Capsella_thracica	*Capsella_orientalis*	*Catolobus_pendulus*	0.087761	2.11738	0.034227	0.017106	16398.8	926.5	777
Capsella_grandiflora	*Capsella_orientalis*	*Catolobus_pendulus*	0.040365	1.84429	0.06514	0.012445	14151.2	1395	1286.75
Capsella_rubella	*Capsella_orientalis*	*Catolobus_pendulus*	0.042557	1.73711	0.082368	0.012702	14287.8	1353.5	1243
Capsella_thracica	*Capsella_grandiflora*	*Catolobus_pendulus*	0.020393	0.75042	0.453002	0.00472	15915.5	1032	990.75
Capsella_thracica	*Capsella_rubella*	*Catolobus_pendulus*	0.021643	0.692274	0.488765	0.004463	16503.2	920.5	881.5
Capsella_rubella	*Capsella_grandiflora*	*Catolobus_pendulus*	0.001809	0.072739	0.942014	0.000259	18032.5	623	620.75

Table S12. Results of maximum entropy modeling for *Catolobus and Capsella* species performed at present time using cross-validation.

Features used in Maxent models include: L – linear, Q – quadratic, H – hinge, P – product, T – threshold. 'Training AUC' refers to the AUC value calculated based on training data, while 'Testing AUC' is the mean AUC value calculated based on the 10% of samples (25% for *C. thracica*) excluded from the training dataset. Overfitting is the difference between training and testing AUC value. CBI is the Continuous Boyce Index (Hirzel et al. 2006)*. AICc is the Akaike information criterion corrected for small sample sizes. For details see Muscarella et al. (2014)*.
The contributions (%) of the environmental variables are given at the end of the table: bio_1 = Annual Mean Temperature, bio_4 = Temperature Seasonality, bio_15 = Precipitation Seasonality, bio_16 = Precipitation of Wettest Quarter, and bio_17 = Precipitation of Driest Quarter.

**Species**	*Catolobus pendulus*	*Capsella orientalis*	*Capsella rubella*	*Capsella grandiflora*	*Capsella thracica*
**No. of samples**	882	68	1033	76	12
**Features**	LQHPT	LQHP	LQHPT	LQHPT	LQ
**Regularization multiplier**	1	2	1	2	1
**Cross-validation folds**	10	10	10	10	4
**Training AUC**	0.939	0.947	0.992	0.991	0.983
**Training CBI**	0.994	0.946	0.991	0.925	0.887
**Overfitting mean**	0.009	0.023	0.001	0.01	0.031
**Overfitting std. dev.**	0.008	0.021	0.001	0.008	0.012
**Testing AUC mean**	0.933	0.939	0.991	0.985	0.972
**Testing AUC std. dev.**	0.009	0.028	0.001	0.012	0.029
**Testing CBI mean**	0.946	0.7579	0.9219	0.7553	0.808
**Testing CBI std. dev.**	0.025	0.098	0.046	0.165	0.254
**AICc**	15089.6	1119.8	15591.5	1054.4	215.8
**bio_1**	48.4	35.8	21.9	25	7.4
**bio_4**	20.2	27.9	29.8	37.9	18.7
**bio_15**	4.9	9.4	22.3	15.3	67.9
**bio_16**	20.6	11.1	2.7	20.4	0.1
**bio_17**	5.9	15.9	23.2	1.4	5.9

* Hirzel, A.H., Le Lay, G., Helfer, V., Randin, C. and Guisan, A. (2006). Evaluating the ability of habitat suitability models to predict species presences. *Ecol Modell.* 199, 142–152.
* Muscarella, R., Galante, P. J., Soley-Guardia, M., Boria, R. A., Kass, J. M., Uriarte, M., et al. (2014). ENMeval: An R package for conducting spatially independent evaluations and estimating optimal model complexity for Maxent ecological niche models. *Methods Ecol. Evol.* 5: 1198-1205. doi: 10.1111/2041-210X.12261
